# Mining SOM expression portraits: feature selection and integrating concepts of molecular function

**DOI:** 10.1186/1756-0381-5-18

**Published:** 2012-10-08

**Authors:** Henry Wirth, Martin von Bergen, Hans Binder

**Affiliations:** 1 Interdisciplinary Centre for Bioinformatics of Leipzig University, Härtelstr. 16-18, D-4107, Leipzig, Germany; 2 Helmholtz Centre for Environmental Research, Department of Proteomics, Permoserstr 15, D-04318, Leipzig, Germany; 3 Leipzig Interdisciplinary Research Cluster of Genetic Factors, Clinical Phenotypes and Environment (LIFE), Universität Leipzig, Philipp-Rosenthalstr. 27, D-4103, Leipzig, Germany; 4 Helmholtz Centre for Environmental Research, Department of Metabolomics, Permoserstr. 15, D-04318, Leipzig, Germany

## Abstract

**Background:**

Self organizing maps (SOM) enable the straightforward portraying of high-dimensional data of large sample collections in terms of sample-specific images. The analysis of their texture provides so-called spot-clusters of co-expressed genes which require subsequent significance filtering and functional interpretation. We address feature selection in terms of the gene ranking problem and the interpretation of the obtained spot-related lists using concepts of molecular function.

**Results:**

Different expression scores based either on simple fold change-measures or on regularized Student’s t-statistics are applied to spot-related gene lists and compared with special emphasis on the error characteristics of microarray expression data. The spot-clusters are analyzed using different methods of gene set enrichment analysis with the focus on overexpression and/or overrepresentation of predefined sets of genes. Metagene-related overrepresentation of selected gene sets was mapped into the SOM images to assign gene function to different regions. Alternatively we estimated set-related overexpression profiles over all samples studied using a gene set enrichment score. It was also applied to the spot-clusters to generate lists of enriched gene sets. We used the tissue body index data set, a collection of expression data of human tissues as an illustrative example. We found that tissue related spots typically contain enriched populations of gene sets well corresponding to molecular processes in the respective tissues. In addition, we display special sets of housekeeping and of consistently weak and high expressed genes using SOM data filtering.

**Conclusions:**

The presented methods allow the comprehensive downstream analysis of SOM-transformed expression data in terms of cluster-related gene lists and enriched gene sets for functional interpretation. SOM clustering implies the ability to define either new gene sets using selected SOM spots or to verify and/or to amend existing ones.

## Introduction

High-throughput genome-scale sequencing and microarray technologies generate huge amounts of data which challenge tasks such as dimension reduction, data compression, visual perception, data integration and extraction of biological information. A natural basis for organizing gene expression data is to group together genes with similar patterns of expression, e.g. of highly correlated expression values. A series of different similarity measures and clustering algorithms have been developed in the last decade for this purpose. Another important task in extracting reliable information is to examine the extremes, e.g., genes with significant differential expression in two individual samples or in a series of measurements and to judge the degree of significance. To interpret the extracted genes in terms of biological function gene set enrichment methods have been developed. They link previous biological knowledge about groups of functionally related genes with the results of differential expression analysis.

This study addresses the question how to combine self organizing maps (SOM) machine learning with differential expression and gene set enrichment analysis. SOMs describe a family of nonlinear, topology preserving mapping methods with attributes clustering and strong visualization through the use of neural networks. They are applied in many fields like bioinformatics for dimension reduction and the grouping and visualization of high dimensional data. Thus, SOMs accomplish two goals: they reduce dimensions and display similarities. Moreover, SOMs are very intuitive and easy to understand and therefore used in decision-making. SOMs were devised by Kohonen [1], and first applied by Tamayo et al. [2] and Törönen et al. [3] to analyze gene expression data.

Our approach follows that of Nikkilä et al. [4] and of Eichler et al. [5] who configured the SOM method in such a way that it combines sample- and feature-centered perspectives to portrait the expression landscapes of individual samples. This method transforms large and heterogeneous sets of expression data into colored images which can be directly compared in terms of similarities and dissimilarities of their textures. These images represent two-dimensional views on high-dimensional data, akin to multidimensional scaling with the following benefits: Firstly, they provide individual visual ‘portraits’ for each sample which serve as new, complex objects for next level analysis in terms of visual recognition and statistical analysis. Secondly, they strongly reduce the dimension of the original data while preserving their information richness (because original data are not removed but remain ‘hidden’ behind the transformed data).

The SOM method is relatively infrequently applied to high-dimensional molecular data compared with alternative approaches such as hierarchical clustering despite these convincing advantages. One reason might be seen in the fact that downstream data mining tasks require the availability of appropriate algorithms and of suited program tools to generate the desired information. The sample ‘portraits’ represent mosaic-images where each tile represents a ‘minicluster’ of single-genes of similar expression profiles. It is characterized by one prototypic expression profile, called metagene, subsuming the mean expression profile of the associated genes. Metagenes of similar profiles usually cluster together into so-called spots due to the specifics of the machine learning algorithm. These spot clusters provide lists of candidate genes co-expressed in the samples studied.

Our previous publication addresses methodical aspects of the machine learning step and details of data structure [6]. SOM machine learning alone is however insufficient to extract important features and biological information from the data. The obtained spot-clusters need further filtering and association with previous knowledge for this purpose. Here we address these data mining tasks with special emphasis on the structure of SOM-transformed data to enable their downstream analysis and biological interpretation.

The first focus of this publication addresses the gene ranking problem in SOM-transformed data. SOM training typically uses a simple fold-change (FC) scale with respect to the mean expression of each gene in the pool of all samples to detect genes of interest. The FC-score however does not provide explicit information about statistical significance for the observed expression changes and thus it might have disadvantages in generating false signals, e.g., if large expression changes are paralleled with high uncertainties of the respective signals or, vice versa, if relatively small changes refer to accurate signals. SOM mapping must therefore be supplemented with appropriate algorithms to assess significance of the features selected. In this publication we apply significance analysis to the spot-clusters of genes identified by the SOM method using three alternative test statistics based either on FC-measures or on regularized Student’s t-statistics with special emphasis on the error characteristics of microarray expression data. Such local, cluster-related lists of genes are expected to improve the resolution of the method to identify sample-specific features with a common functional impact.

The second focus of this publication addresses gene set enrichment analysis under special consideration of the spot-clusters generated by SOM machine learning. It is based on the fact that the importance of genes in terms of their relation to a particular molecular function is not necessarily associated with strongest or most significant changes of expression provided by their rank in the obtained lists. Instead, it can also involve weak but consistent alterations of transcript abundance. Therefore gene set based methods have been developed to investigate phenotypic changes at the level of biological function considering, for example, the involvement of genes into signalling pathways, their relation to cellular components or their chromosome location [7-13]. These methods essentially assess the enrichment of a set of several genes in the list of differentially expressed genes compared with the total reservoir of genes studied. The members of the set are defined a priori by some biological commonality for certain phenotypes. The main advantage of such methods over single gene based methods is that they directly link the ranked gene list with biological knowledge and therefore provide better functional insight into the cause of the phenotypic differences under study.

Our work thus aims at refining the avenues for feature mapping and data reduction offered by SOM machine learning. We use the microarray expression data of a series of 67 different human tissues taken from ten tissue categories such as nervous, immune system, epithelial and muscle tissues as an illustrative example to demonstrate the strengths of the SOM method in disentangling large heterogeneous data sets.

The paper is organized as follows: In the Results-section we present and discuss our approach of significance and enrichment analysis of SOM-transformed data if applied to the tissue body-index data set. In the methodical part we provide details of the applied methods and algorithms and of relevant characteristics of microarray data. In the additional material we address aspects of SOM data mining which supplement our main results. Finally, we complemented our R-package ‘oposSOM’ [6] with appropriate add-on functions enabling the differential expression and gene set enrichment analysis of SOM-transformed microarray data.

## Results

### Mining SOM expression portraits - an overview

**Figure 1:**
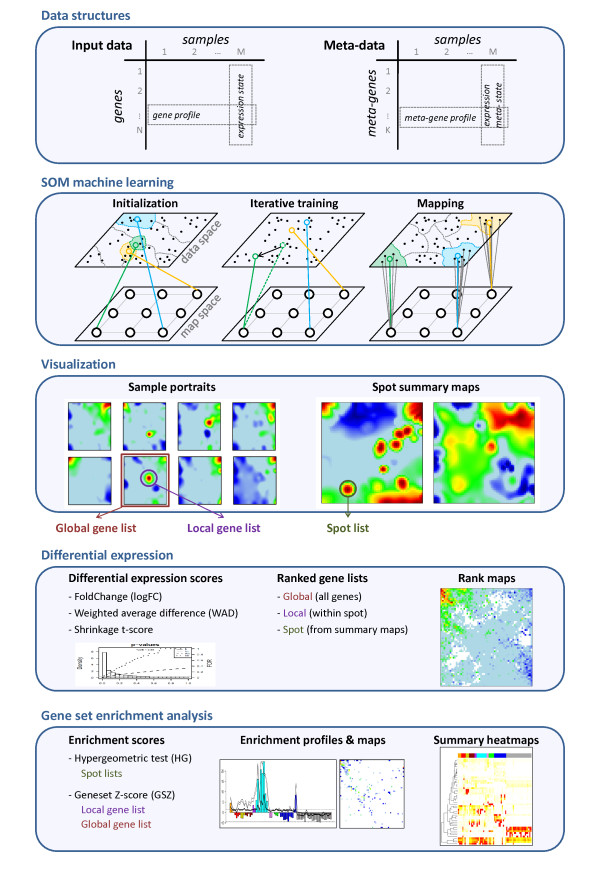
Schematic overview about our SOM expression analysis pipeline.

Figure 1 summarizes the main ingredients of our SOM analysis pipeline. Details of the method and of the different analysis algorithms are provided in the Methods section below and, partly, in our previous publication [6]. In short: SOM is a neuronal network algorithm which transforms high-dimensional input data into ‘meta-data’ of lower dimension. Both data types are given as matrixes where the rows are the feature values (expression levels of a number of genes/metagenes here) and the columns are the samples measured typically under different conditions (see the first box in Figure 1). Each row-vector (called expression profile) thus characterizes the expression of one gene/metagene in the series of samples whereas each column (called expression state) characterizes the expression ‘landscape’ of all genes in one sample. As a rule of thumb, the number of genes typically exceeds the number of metagenes by, at minimum, one order of magnitude. 

SOM machine learning iteratively adjusts the metadata (map space) to the input data (data space) using the *Euclidean distance* as criterion after appropriate initialization (see the second box in Figure 1). This training of the map ensures that the obtained metagene profiles cover the diversity of expression profiles inherent in the data. Finally, the input data are mapped to the metadata such that each metagene profile serves as representative of a minicluster of single genes with similar expression profiles as indicated by lines connecting selected metagenes in the map space with the associated single genes in the input space in the second box in Figure 1.

Intuitive visualization is a third strength of the SOM method besides dimensionality reduction and clustering (third box in Figure 1). Particularly, the metagene expression of each sample is transformed into one image which ‘portrays’ its expression state in terms of a color texture. It allows identifying clusters of co-regulated *and* over- or underexpressed genes as red and blue spots, respectively. Spot summary maps provide an overview of all spot clusters observed (see [6] for details).

Expression analysis then aims at extracting local (i.e. including genes selected into one spot-cluster) and also global (including all genes studied) lists of differentially expressed genes for each of the conditions studied (fourth box in Figure 1). Global lists can be visualized in the SOM-map space using rank maps. Details of the gene-ranking problem and of multiple test adjustment are addressed below using different significance scores and false discovery estimates, respectively.

The second focus of this publication deals with the ‘function-mining’ problem using gene set enrichment techniques (fifth box in Figure 1). Particularly we aim at extracting information about the functional context of the genes clustered in a selected spot or, alternatively, we map genes of common function (so-called gene sets) into the map space of the SOM. The following pseudocode summarizes the machine learning steps and downstream analyses:

***# SOM Training & Mapping (see, e.g.,***[1]***for detailed descriptions)***

**Input:** input-data, SOM-size

Step 1: Initialization

eigenv1, eigenv2 ← CalculateEigenVectors( input-data )

for( x, y in 1… SOM-size _x,y_ ):

{

coeff_x_ ← 2 · ( (x-1) / (SOM-size_x_ -1) ) – 1

coeff_y_ ← 2 · ( (y-1) / (SOM-size_y_ -1) ) − 1

meta-data_x,y_ ← coeff_x_ · eigenv1 + coeff_y_ · eigenv2

}

Step 2: Iterative training

for( i in 1…max-iterations ):

{

gene-profile ← SelectTrainingProfile( input-data )

BMU ← FindBestMatchingMetagene( meta-data, gene-profile )

learning-rate ← CalculateLearningRate( i, max-learning-rate, max-iterations )

for( metagene-profile in meta-data ):

{

neighborhood-factor ← CalculateNeighborhoodFactor( BMU, neighborhood-function, i )

metagene-profile ← metagene-profile + learning-rate · neighborhood-factor · ( gene-profile − metagene-profile )

}

}

Step 3: Final mapping

for( gene in input-data ):

{

gene-profile ← SelectGeneProfile( input-data, gene )

BMU ← FindBestMatchingMetagene( meta-data, gene-profile )

gene-to-metagene-mapping_gene_ ← BMU

}

for( metagene in meta-data ):

{

for( gene in input-data ):

{

if( gene-to-metagene-mapping_gene_ = metagene ): metagene-cluster_metagene_ ← metagene-cluster_metagene_ ∪ gene

}

}

**Output:** meta-data, gene-to-metagene-mapping, metagene-cluster

***# Visualization of the meta-data (see***[6]***for detailed descriptions)***

**Input:** meta-data

Step 1: Generate sample portraits

for( expression-state in meta-data ):

{

CreateImage( expression-state, colorcode: minimum ← blue, mean ← green, maximum ← maroon )

}

Step 2: Generate spot summary maps

for( metagene in meta-data ):

{

metagene-profile ← SelectGeneProfile( meta-data, metagene )

overexpression-summary_metagene_ ← GetMaximum( metagene-profile )

underexpression-summary_metagene_ ← GetMinimum( metagene-profile )

}

CreateImage( overexpression-summary, colorcode: minimum ← blue, mean ← green, maximum ← maroon )

CreateImage( underexpression-summary, colorcode: minimum ← blue, mean ← green, maximum ← maroon )

**Output:** Images: Sample expression portraits, spot summary maps ; Objects: overexpression-summary, underexpression-summary

*# Spot detection and gene lists*

**Input:** meta-data, overexpression-summary, underexpression-summary, expression-threshold

Step 1: Sample portrait spot detection

for( sample in meta-data ):

{

expression-state ← SelectExpressionState( meta-data, sample )

spot-metagenes ← { metagenes | expression-state > expression-threshold }

local-gene-lists_sample_ ← SplitSeparatedSpots( spot-metagenes )

}

Step 2: Summary map spot detection

spot-metagenes ← { metagenes | overexpression-summary > expression-threshold }

spot-gene-lists_overexpression_ ← SplitSeparatedSpots( spot-metagenes )

spot-metagenes ← { metagenes | underexpression-summary < -expression-threshold }

spot-gene-lists_underexpression_ ← SplitSeparatedSpots( spot-metagenes )

**Output:** local-gene-lists, spot-gene-lists>

*# Differential expression analysis:*

**Input:** input-data, meta-data, local-gene-lists, spot-gene-lists, metagene-cluster

Step 1: Ranked gene lists

for( gene,sample in input-data ):

{

WAD_gene,sample_ ← CalculateWAD( input-data ) *# see equation (*1*)*

t-score_gene,sample_ ← CalculateTScore( input-data ) *# see equation (*2*)*

}

global-gene-lists_logFC, WAD, t-score_ ← RankGenes( input-data, WAD, t-score )

local-gene-lists_logFC, WAD, t-score_ ← RankGenes( local-gene-lists, input-data, WAD, t-score )

spot-gene-lists_logFC, WAD, t-score_ ← RankGenes( spot-gene-lists, input-data, WAD, t-score )

Step 2: Rank maps

for( sample in meta-data ):

{

for( metagene in meta-data ):

{

logFC-map_metagene_ ← GetAverage( global-gene-list_logFC_, metagene-cluster_metagene_ )

WAD-map_metagene_ ← GetAverage( global-gene-list_WAD_, metagene-cluster_metagene_ )

t-score-map_metagene_ ← GetAverage( global-gene-list_t-score_, metagene-cluster_metagene_ )

}

CreateImage( logFC-map, colorcode: minimum ← blue, mean ← green, maximum ← maroon )

CreateImage( WAD-map, colorcode: minimum ← blue, mean ← green, maximum ← maroon )

CreateImage( t-score-map, colorcode: minimum ← blue, mean ← green, maximum ← maroon )

}

**Output:** Images: Sample rank maps ; Objects: global-gene-lists, local-gene-lists, spot-gene-lists

*# Gene set enrichment analyses:*

**Input:** input-data, meta-data, local-gene-lists, spot-gene-lists, GO-gene-set-collection

Step 1: Spot-related and global gene set enrichment analysis

for( spot in spot-gene-lists ):

{

for( gene-set in GO-gene-set-collection ):

{

spot-HG_spot,gene-set_ ← PerformHGtest( spot-gene-lists_spot_, gene-set ) *# see equation (*8*)*

}

}

for( sample,spot in local-gene-lists ):

{

for( gene-set in GO-gene-set-collection ):

{

local-GSZ_sample,spot,gene-set_ ← PerformGSZtest( input-data_sample_, local-gene-lists_sample,spot_, gene-set ) *# see equation (*10*)*

}

}

for( sample in global-gene-lists ):

{

for( gene-set in GO-gene-set-collection ):

{

global-GSZ_sample,gene-set_ ← PerformGSZtest( input-data_sample_, gene-set ) *# see equation (*10*)*

}

}

Step 2: Gene set enrichment summary heatmap

for( sample,spot in local-gene-lists ):

{

top-three-gene-sets_sample,spot_ ← GetTopRankedGeneSets( local-GSZ_sample,spot_ )

}

top-three-gene-sets ← RemoveDuplicates( top-three-gene-sets )

CreateClusteringHeatmap( local-GSZ_top-three-gene-sets_, colorcode: minimum←white, mean←yellow, maximum←red )

Step 3: Gene set enrichment profiles

for( gene-set in GO-gene-set-collection ):

{

CreateBarplot( global-GSZ_gene-set_ )

}

Step 4: Gene set enrichment maps

for( gene-set in GO-gene-set-collection ):

{

for( metagene in meta-data ):

{

metagene-HG_metagene,gene-set_ ← PerformHGtest( metagene-cluster_metagene_ , gene-set ) *# see equation (*8*)*

}

CreateImage( -log_10_( metagene-HG_gene-set_ ), colorcode: minimum←blue, mean←green, maximum←maroon )

}

**Output:** Images: GSZ summary heatmap, enrichment profiles & maps; Objects: spot-HG, metagene-HG, local-GSZ, global-GSZ

### SOM-portraits and rank maps

**Figure 2:**
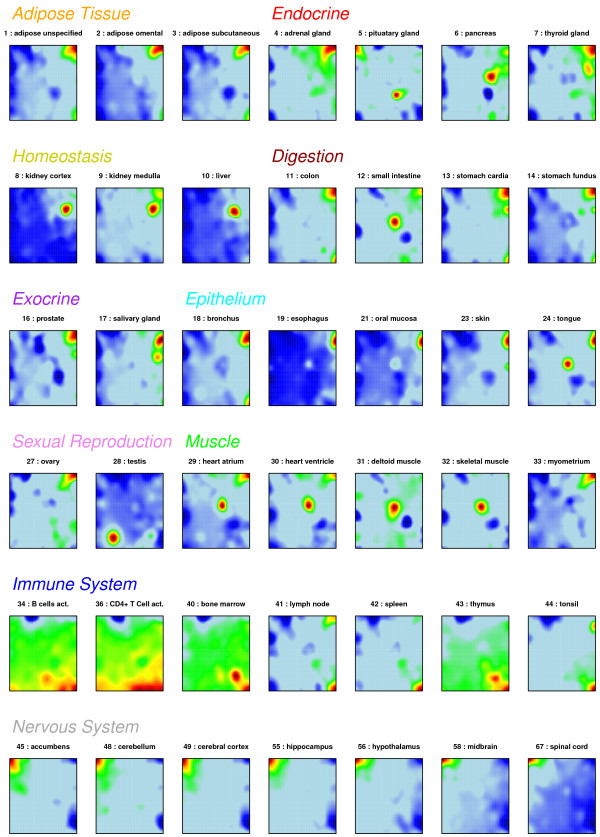
**Gallery of SOM portraits of 42 selected tissues of different tissue categories such as adipose, endocrine tissues.** The colors of the respective headings are used below to assign the respective tissue categories (e.g., in Figure 11 below).

Genome-wide gene expression data of 67 selected tissues taken from 10 tissue categories were pre-processed and subsequently used to train a SOM as described in the methodical part. Figure 2 shows the obtained SOM-portraits of selected tissues using a 60x60 mosaic grid. The method identifies coherent tissue-specific texture patterns of gene expression readily discernable in the obtained gallery of SOM images. Particularly, our SOM machine learning method partitions the more than twenty thousand ‘single’ genes probed by each microarray into 3600 miniclusters arranged in a two-dimensional mosaic map. Each minicluster refers to one metagene. Its expression profile serves as representative of the respective minicluster of co-regulated single genes. Their number typically varies from tile to tile.

The color gradient of the map was chosen to visualize over- and underexpression of the metagenes compared with the mean expression level in the pool of all tissues studied. The obtained images visualize the specific expression pattern of each sample in terms of a color-coded texture indicating regions of over- and underexpression by red and blue spots, respectively. Most of the spots are tissue specific features which are found only in one or a very few tissue categories such as nervous, immune system or muscle tissues.

Note that the color textures of the individual portraits visualizes the ‘expression landscape’ of human tissues which is governed by different, partly tissue-specific expression modules of co-regulated genes evident as spots in the respective images. The observed pattern is far from a random one: In the supplementary text (Additional file 1) we compare properties of the textures observed in the tissue data with that of a randomized expression landscape of equal size. The latter one is characterized by much more numerous, SOM-size dependent and mutually independent expression modules when compared with the tissue data.

**Figure 3:**
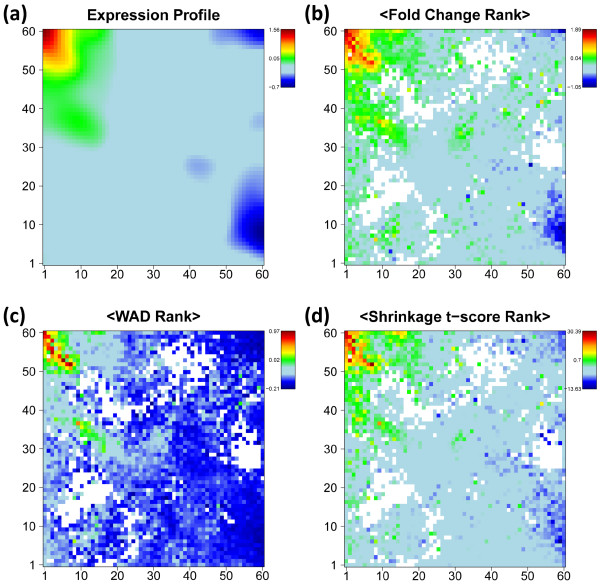
**Expression image of *nucleus accumbens *(‘standard’ SOM profile, panel a) and the average-rank maps for FC, WAD and shrinkage t-score statistic (b-d).** The numberings of the tiles k=1…60 are given at the vertical and horizontal borders of the SOM. White areas indicate empty metagenes.

Gene expression analysis aims at extracting lists of genes ranked with decreasing ‘importance’ in the actual context. The ‘importance’ can be judged using different criteria such as the log-expression difference with respect to a reference state or its significance which takes into account in addition also the error level of the measurement. In the next step we therefore map ranked lists of genes using the SOM-grid. Figure 3 shows such SOM expression images of one particular tissue example, nucleus accumbens, taken from the category of nervous tissues in log FC units (panel a) together with the respective average-rank maps for three different expression scores described in the Methods section (panels b-d), namely the FC-, weighted average difference (WAD)- and shrinkage t-score, respectively (see (Eqs. (1) and (2) in the methodical part). The rankings of genes refer to total gene lists which contain all genes studied. These maps color-code the mean rank of each metagene which was calculated as the arithmetic average over the individual rankings of the associated single genes in the total list. In general, genes on top of the list accumulate in the red overexpression spot of the standard SOM-profile however with a few exceptions, e.g. in the range of the green spot below the red one.

The three alternative scores provide very similar pattern, however with subtle differences: The contrast, i.e. the gradient between areas of under- and overexpression is largest for the WAD-ranking and smallest for FC-ranking with t-shrinkage in-between. Similar trends are observed for the SOM expression profiles which are color-coded according to the FC- and WAD-scores of their metagenes. Note also that the rank maps reveal subtle details within the SOM-spots such as the chain-like cluster of metagenes of small rank within the overexpression spot (compare panel a with b-d in Figure 3). The analysis of such fine-structures might help to refine the subsequent selection of relevant genes within the spots.

**Figure 4:**
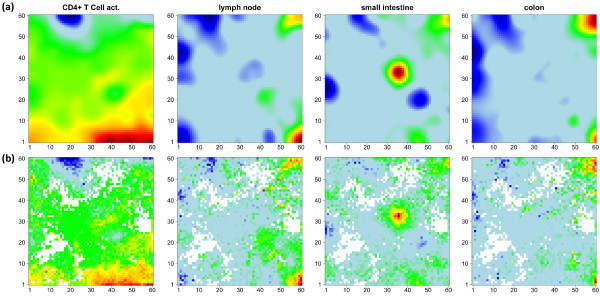
**Comparison of standard SOM in log FC-scale (panel a) with rank maps based on global gene lists according to the t-shrinkage statistics (panel b).** Metagenes of high overexpression and of small average rank of the associated single genes are coded in red. Both options show essentially similar textures. The rank maps partly reveal more detailed spot pattern or a low overall rank level (blue, e.g. colon). The atlas of rank maps of all tissues studied is shown in Additional file 2.

The examples shown in Figure 4 further support this result: The t-shrinkage rank-map of small intestine, T-cells and lymph node show a partly better resolved fine structure of highly ranked genes in different regions of the map than the standard SOM mosaics which use the log FC expression scale. On the other hand, the rank map of colon is dominated by blue areas which reveal an average level of relatively low rankings. This effect presumably reflects the relatively small expression level of the genes in the overexpression spot in the top right corner of the map which give rise to relatively large rank numbers. The whole atlas of the rank maps of all tissues studied is shown in Additional file 2.

### Global gene lists

The alternative scores generate ordered global lists of genes for each tissue with characteristic differences between the methods as illustrated in the rank-map shown in Figure 3. The WAD-score, for example, strongly weights highly expressed genes which concentrate in a few metagene-tiles in the top left corner of the map. As a consequence, these metagenes occupy smaller ranks in the WAD-list than in the respective FC- or shrinkage-t lists with consequences for the textures of the respective rank maps. The present study does not aim at comparing the performance of different expression scores in absolute units, an objective which is problematic in the absence of a suited gold standard. Previous work makes use either of synthetic simulation data, of correlation measures in real-world chip applications or of special calibration data sets to judge the quality of different expression scores [14-19]. It turned out that t-shrinkage and different FC-based scores such as the WAD-score are generally suited measures to generate lists of regulated genes. Here we apply the three scores as three complementary alternatives with a specific focus on different expression properties: Particularly, WAD-lists heavily weight strongly expressed genes. In consequence, subtle expression changes of weakly expressed genes potentially get lost in WAD-lists. FC-lists directly rank the genes according to their differential expression and thus represent a simple and intuitive measure related to the change of mRNA abundance. FC-lists are however prone to generate false positives because the FC-score equally weights strongly and weakly expressed genes with usually smaller and larger noise levels, respectively. The t-shrinkage score explicitly considers the noise level of the genes which however might raise problems due to the uncertainty of the error estimates as discussed in the methodical section. Because of their specific advantages and disadvantages we consider the different scores rather as complementary measures than as competitive ones providing information which mutually supplement each other.

**Figure 5:**
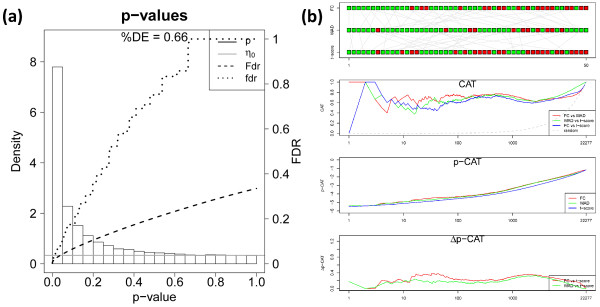
Global significance analysis of accumbens sample: p-value distribution and fdr- and FDR-curves of the t-shrinkage statistics (panel a) and comparison of gene rankings for FC-, WAD and t-shrinkage scores using the RC-, CAT-, p- and Δp-CAT plots (panel b).

Figure 5a shows the p-value distribution of differential expression of nucleus accumbens based on the t-shrinkage score (the atlas of the p-value distributions of all tissues studied is given in Additional file 3). It well separates into a constant noise floor and the left-skewed subpopulation of differentially expressed genes constituting a percentage of about 66% of all genes available. We compare the global lists ranked with increasing t-shrinkage, FC- and WAD-scores using four plots, namely (i) the rank comparison (RC), (ii) the correspondence at the top (CAT)- ,(iii) the p-CAT and (iv) the Δp-CAT plots (Figure 5b, see Methods section for description). The RC-plot compares the individual positions on top of the lists by appropriate color-coding. It reveals moderate disordering between the three lists where most ranks agree within ±20 positions up to rank r=50 (see green symbols). The CAT-plot presents the cumulative fraction of common genes on top of the list for positions below a running threshold. In our example it shows that best agreement is achieved in FC/WAD-comparisons for ranks r ~ 10…100. However, also the other combinations provide acceptable agreement between the lists with CAT(r)≥ 0.5 for positions r<100, meaning that at minimum 50% of the same genes are included in pairs of lists up to rank one hundred.

The p-CAT plot estimates the agreement between the lists in units of the cumulative log p-value of the t-shrinkage statistics. It enables to differentiate whether a given CAT-value refers to more similar or very different p-values and thus it estimates the importance of rank differences. The respective Δp-CAT plot shows the difference between the p-CAT value of the FC- or WAD-score and that of the t-shrinkage statistics which provides the lower margin per definition. The Δp-CAT values of the global lists of the FC- and WAD-scores initially increase for ranks below 5-20 indicating that the different rankings are associated with clearly different p-values. For positions r> 20 the Δp-CAT values remain virtually constant indicating that the alternative lists provide consistent results where rank differences reflect rather the noise inherent in the data than systematic biases between the scores used.

### Local, spot-related gene lists

The spot-texture of the SOM portraits of individual tissues implies to generate spot-related gene lists by taking into account only the single genes which are associated with the metagenes forming a particular spot. Recall that a spot clusters genes of similar and thus co-variant expression profiles in the series of samples studied. Our spot-based significance analysis therefore shares similarity with methods which exploit the correlation between genes in significance testing of differential expression [20,21] because it selectively applies to sub-ensembles of genes of highly correlated expression profiles.

**Figure 6:**
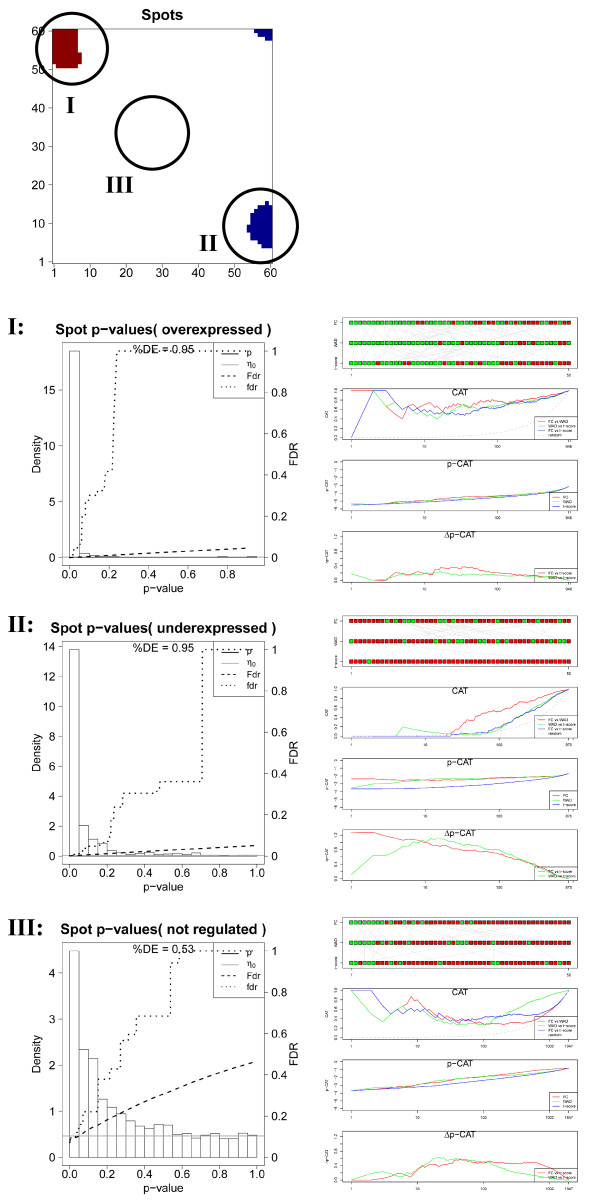
Local significance analysis of selected spots of the accumbens sample (see part above): p-value distribution and fdr- and FDR-curves of the t-shrinkage statistics (left part) and comparison of gene rankings for FC-, WAD and t-shrinkage scores using the RC-, CAT-, p- and Δp-CAT plots (right part).

In the next step we therefore analyzed the p-value distribution and the mutual list characteristics for three selected spots referring to over- (spot I), under- (spot II) and indifferent (spot III) expression (see Figure 6) which contain different numbers of single genes (I: 980, II: 745, III: 1,947). Spots of regulated metagenes are detected for each tissue using the 98% / 2% quantile criterion for over- / underexpressed metagenes, respectively. The fraction of differentially expressed genes in the spots either markedly exceeds (I,II: %DE=0.95) or falls below (III, %DE=0.53) the global value (%DE=0.66). The ranking characteristics of the overexpression spot I closely resembles that of the global lists indicating that this spot contains most of the ‘leading’ genes of the global list (compare Figures 5 and 6). Note that the overexpression spot selects strongly differentially expressed genes. Therefore the level of agreement between the alternative lists is slightly better especially for FC/WAD-comparison (CAT(r<100) ~ 0.6) compared with the respective comparisons between the global lists. Note that the spot-filtering effectively combines the scoring of differential expression with the selection of co-expressed and correlated genes. It has been previously shown that ‘correlation-sharing’ for the detection of differentially expressed genes improves the performance of the analysis in terms of the false discovery rate [20]. Effectively, the consideration of additional information about co-expression in other samples obviously removes false positives and thus improves the lists of differentially expressed genes. For spot I we indeed obtain a much smaller total cumulative FDR value of Fdr(p=1)≈0.05 (Figure 5a) compared with the total list (Fdr(p=1)≈0.35; Figure 6). 

Contrarily, the alternative gene lists taken from the underexpression spot II largely diverge revealing the lack of agreement among the top 10 – 50 features. The CAT-plot shows best agreement for FC/WAD-comparisons with CAT(r≈100)< 0.6 and worst for FC/t-shrinkage (CAT(r≈100)<0.2). These rank comparisons are paralleled by relatively large differences of the p-CAT and Δp-CAT characteristics revealing systematic and significant rank differences due to the specific biases of the used scores. Particularly, FC/t-shrinkage comparisons shows largest dissimilarity in the CAT- and p-CAT-plots for r<50 followed by WAD/t-shrinkage comparisons. These discrepancies can be rationalized by the large uncertainty of low expression genes which accumulate in the underexpression spot.

Interestingly, also spot III contains a large fraction of differentially expressed genes (%DE=0.53) despite the fact that the metagene expression is virtually on the moderate level. The comparisons between the alternative lists provide less agreement when compared with spot I but almost similar trends. The spot of ‘mean expression’ obviously still contains residual amounts of significantly differently expressed genes which appear as green and grey tiles in the region of spot III in the rank-map (Figure 3).

To generalize these results we calculated mean global and local CAT(r) and Δp-CAT(r) values for lists of length r=10 and 100 of all tissue samples studied considering either all genes or the genes of the strongest overexpression spot, respectively (see Additional file 1 for details). The results of these global and local rank comparisons confirm the trends discussed above: global FC- and WAD-lists of length r=10 – 100 agree to about 70% on the average whereas global FC/t-shrinkage and WAD/t-shrinkage lists are identical to about 50%. Local lists are slightly more similar by a few percent than global ones due to the pre-filtering of the genes in the SOM-spots. The respective Δp-CAT values reveal that the significance level of the alternative scores is virtually identical for all considered lists.

In summary, the different scoring methods typically provide similar and virtually equivalent gene lists for overexpression spots but diverging lists for underexpression spots. The rank-maps of the respective methods clearly express these differences: The regions of overexpression are essentially similar in the different rank-maps (see red areas in Figure 3). Contrarily, the regions of underexpression largely differ in their texture. They appear either as relatively localized spots in the FC-rank and, to a less degree, in the WAD-rank maps or they ‘smear’ over larger regions in the shrinkage-t rank map due to the large uncertainty of low expression values.

In conclusion, overexpression rankings provide robust lists of differentially expressed genes which are relatively independent of the scoring method used thus allowing the quantitative analysis in terms of the obtained rank and expression level. In contrast, underexpression lists are highly uncertain providing essentially qualitative information, namely that the respective genes are weakly expressed. Discrimination analysis between the different samples and especially GO-enrichment analysis to identify overrepresented gene sets should therefore focus on overexpression spots. The t-shrinkage score will be applied as the default criterion for gene ranking in the remainder of this study.

### Global overrepresentation analysis

The correlation and co-expression of the gene profiles in each spot can be utilized as a simple heuristic with implications for tentative gene function because biological processes are governed by coordinated modules of interacting molecules [22]. Application of gene set enrichment analysis to the series of about one dozen stable over- and underexpression spots detected in the SOM of human tissues will make explicitly use of this ‘guilt-by-association’ principle which assumes that co-expressed genes are likely to be functionally associated [22,23]. Enrichment analysis is expected to assign putative gene function(s) to the selected spots. Below we compare several options of enrichment analysis estimating either ‘overrepresentation’ of the members of a priori functional gene sets in the spot list, their ‘overexpression’ in terms of differences of the average expression levels in the set and the list and the combination of both options.

Nine overexpression spots are identified in the SOM-images of all tissues studied using the 98-percentile criterion of maximum expression. These spots are collected into one, so-called overexpression summary map as described in [6]. Subsequently GO-gene set overrepresentation analysis using the hypergeometric (HG-) test is applied to the lists of genes contained in each of the overexpression spots (see the Methods section below). Particularly, the genes associated with each spot are analyzed for overrepresentation of genes taken from the collection of 1454 gene sets downloaded from the GSEA-homepage according to the GO-categories molecular function, biological process and cellular component. The HG-test then provides an ordered list of gene sets ranked with decreasing significance of overrepresentation with respect to the random appearance of genes from the set in each of the spots.

**Figure 7:**
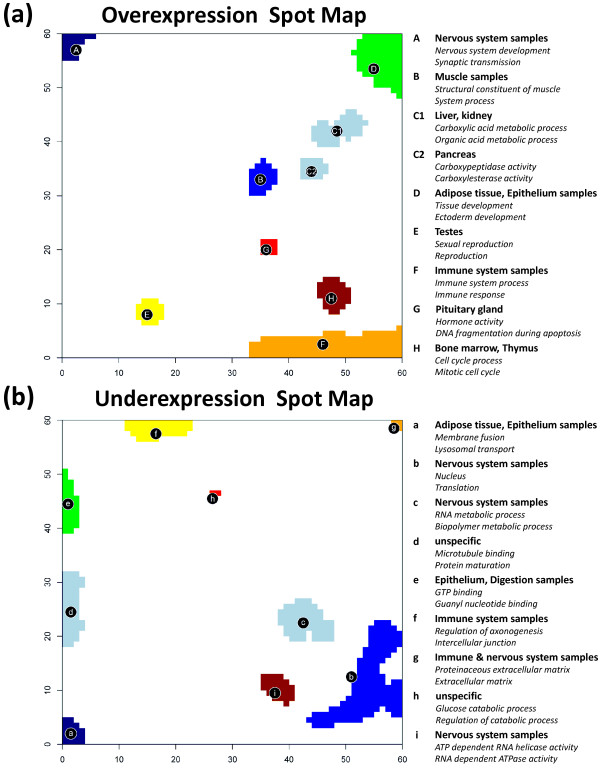
**The over- and underexpression summary spot maps show nine spots each which are strongly over-/underexpressed in different tissues (part a and b, respectively).** Overrepresentation of a collection of 1454 gene sets is estimated for each spot using the hypergeometric distribution. The right legend assigns the two most significantly overrepresented gene sets to the respective spots.

Figure 7a shows the overexpression summary map with the nine spots of strongly overexpressed metagenes. The legend assigns the two leading overrepresented gene sets in the list of each of the spots to get a first idea about the possible biological context of the genes in the spots. For example, spot A in the top left corner of the SOM is clearly related to molecular processes in nervous cells according to the two leading gene sets. The more detailed inspection of the lists reveals that ten out of the top-twenty gene sets of spot A are related to nervous system (see Additional file 1). Also other tissue-specific spots can be associated with distinct molecular functions such as immune system processes (immune systems samples, spot F), sexual reproduction (testis, spot E) or muscle contraction (muscle tissues, spot B). Hence, the functional context of the different spots according to previous knowledge is clearly related to the tissues showing the respective overexpression spot.

The analogous overrepresentation analysis was performed for the underexpression spots related to local minima of the metagene expression profiles (Figure 7b). The functional context of these spots thus refers to genes which are strongly underexpressed in the tissues showing this spot (see also the respective spot expression heatmap shown in Additional file 1). For example, spot b, c and g related to processes in the nucleus, RNA processing and the extracellular region, respectively, are underexpressed in most nervous tissues. Spot g and also spot f (related to neurogenesis) are underexpressed in immune system tissues. The latter spot, in turn, shows clear overexpression in nervous tissues, which is however not detected in the overexpression map selecting only the regions of strongest overexpression.

Thus, overrepresentation analysis of both, over- and underexpression spots provide complementary information: On one hand, they allow to assign antagonistic gene activities in the same tissue and in different tissues. On the other hand, parts of the underexpression spots occupy different regions of the map than the overexpression spots. In consequence, combination of both maps extends the range of relevant gene sets and thus also the functional context studied. For example, spots a and d related to biopolymer metabolism and microtubules, respectively, are not detected in the overexpression map. Spots e and f are both overexpressed in nervous tissues. They occupy regions near the spot A also overexpressed in nervous tissues. The respective functional context of all three different spots allows to disentangle subtle details of gene activity in nervous tissues. A similar relation exists for overexpression spot F and underexpression spot b, where the former one overrepresents gene sets related to cell cycle and the latter one gene sets related to nucleus activity.

### Alternative spots selections

In the previous subsection we have shown that over- and underexpression spots partly occupy different regions of the map with complementary information about their functional context. One can apply also alternative methods of spot selection using hierarchical clustering of the metagenes based on the Euclidean distance between them or determining correlation cluster based on Pearson correlation coefficients between the metagenes [6]. The former method provides an area-filling fragmentation of the map into different spots which typically occupy larger areas than the spots from the over-/underexpression summary maps. In the Additional file 1 we demonstrate that the cluster-spots detect, for example, different groups of genes related to the functioning of nervous tissues. The correlation clusters provide almost similar results however also with subtle specifics of their functional context (Additional file 1). This method preferentially selects areas of highly variable metagenes along the border of the map with subtle differences between the functional context of adjacent clusters.

In summary, different spot selection algorithms and criteria fragment the expression landscape of the map in partly different ways with complementary information about the functional context of the associated genes. The suitability of the different methods depends on the particular aims of the issues studied and is not in the focus of this methodical publication. In the remainder of the paper we will use the overexpression spots to extract further functional information from the maps. Note however that over- and underexpression spot selection can be applied to the individual portraits of each sample and thus they provide specific enrichment characteristics as described below. In contrast, the k-means and the correlation clusters are based on the similarities between the metagene profiles and thus they refer to all samples in terms of the global overrepresentation of the associated genes. Application of the GSZ-score allows however to study also sample-specific enrichment of the respective genes (see below).

### HG-enrichment analysis

Gene set overrepresentation analysis as described in the previous subsection applies to global spots of adjacent metagenes taken from the overexpression summary map. The real genes associated with each spot are the same in all tissues studied because the overexpression spot map summarizes the maximum size of each spot sizes observed in any of the tissues and thus it neglects sample-specific alteration of the spot size. This global approach applies to the whole series of tissue samples. It consequently lacks sample-specificity. Thus, overrepresentation of a selected gene set is independent of the individual expression level of the genes in the different samples. In the following we present and discuss two approaches to take into account sample-specific gene expression. We will use the term gene set overexpression analysis if the mean expression of the set-members is compared with the mean expression of all genes in the list without considering the number of set members in the list in contrast to gene set overrepresentation analysis which is based solely on the latter criterion. The term enrichment analysis will be used if both criteria, overrepresentation and overexpression, are combined which enables the refinement of gene set analysis in terms of sample-specificity.

**Figure 8:**
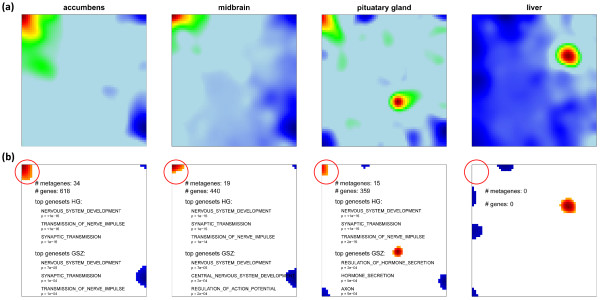
**Local spot characteristics of the ‘nervous’ spot A in different tissues.** Panel **a** shows the original expression profile of selected tissues and panel **b** the selected overexpression spot(s) by applying the 98% quantile criterion to the metagenes (red color). Note that the spot size (# of metagenes) and consequently also the number of associated genes with spot A (red circle) changes from tissue to tissue affecting the results of enrichment analysis using either the HG- or the GSZ-scores: The top three gene sets are given for each of the examples.

The first option of HG-enrichment analysis simply substitutes the global spots by tissue specific ones. These local spots are determined individually for each tissue-specific SOM by applying the 98-percentile threshold. The size of one particular spot usually varies from tissue to tissue and it can even disappear if the expression values of the respective metagenes do not meet the threshold criterion as illustrated in Figure 8 for the ‘nervous tissue’-spot A. In consequence, the spot-related lists of single genes and the derived list of overrepresented gene sets vary between the different samples. Subsequent application of overrepresentation analysis based on the HG-distribution (Eq. (8)) to these local spots provides tissue-specific p-values and thus one list of overrepresented gene sets for each of the spots in each of the samples.

**Figure 9:**
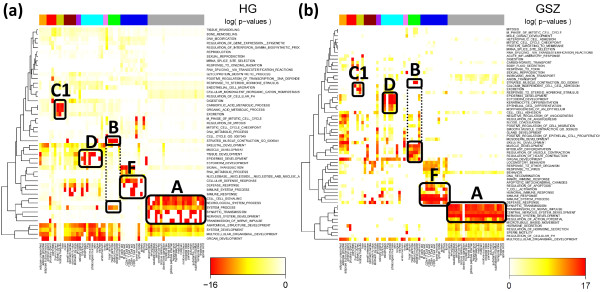
**One-way hierarchical clustering heatmap of significantly enriched gene sets (rows) versus tissues (columns) using the HG- (a) and the GSZ- (b) statistics.** The three-top gen sets per overexpression spot are selected in each of the maps. The heatmap color-codes the p-values of the respective score in log-scale (see the legends in the figure). The tissue categories are color-coded in the bar above the heatmap. The gene sets are clustered in vertical direction. The capital letters approximately assign clusters of enriched gene sets in correspondence with the spots selected in Figure 7a. The GSZ-score provides a larger number of gene sets (factor 1.8) and thus a more diverse pattern.

We selected the top-three gene sets per spot in each tissue and merged them into one global list of most enriched gene sets in all spots. Finally, this global list was converted into the HG-enrichment heatmap shown in Figure 9a. We applied hierarchical clustering to group similarly expressed gene sets in vertical direction. It reveals five to six gene sets associated with the ‘nervous tissue’-spot A in a tissue-specific fashion. Other groups of enriched gene sets can be associated with immune systems tissues (F), muscle tissues (B), epithelial (D) and homeostasis tissues (C1). The selected gene sets are listed in Table 1. Please note that we chose the same capital letters as labels as were used for the spot assignments discussed above for sake of comparison (see Figure 7a).

**Table 1:** **Molecular characteristics of selected overexpression spots as obtained by HG- and GSZ-enrichment analysis**^**a**^

**Spot**	**GSZ**	**HG**
**A**	Synaptic Transmission	Cell-Cell Signaling
	Transmission of Nerve Impulse	Neurological System Process
	Central Nervous System Development	Synaptic Transmission
	Nervous System Development	Transmission of Nerve Impulse
	Regulation of Action Potential	Nervous System Development
**B**	Muscle Development	Striated Muscle Contraction
	Myoblast Differentiation	System Process
	Regulation of Muscle Contraction	
	Regulation of Heart Contraction	
	Striated Muscle Contraction	
**C1**	Carboxylic Acid Metabolic Process	Calcium Independent Cell-Cell Adhesion
	Organic Acid Metabolic Process	Excretion
	Excretion	Response to Steroid Hormone Stimulus
**D**	Epidermis Development	Tissue Development
	Ectodermis Development	Epidermis Development
	Keratinocyte Differentiation	Ectodermis Development
	Epithelial Cell Differentiation	
	Morphogenesis of an Epithelium	
**F**	Regulation of Apoptosis	Cellular Defense Response
	T-Cell Activation	Defense Response
	Humoral Immune Resonse	Immune System Process
	Immune System Process	Immune Response
	Immune Response	
	Defense Response	

^a^ Gene sets enriched in both approaches are printed in bold letters.

### GSZ-enrichment analysis

HG-enrichment analysis applies a binary ‘included-or-not included’ criterion to assess the positive membership of the genes from a gene set in a selected spot-cluster. The gene set Z (GSZ)-score (Eq. (10), see the Methods section below) provides an alternative, second option for enrichment analysis which explicitly considers the individual expression values of the genes included in the list. The algorithm of GSZ-enrichment analysis is largely identical with that of HG-enrichment analysis; namely it starts with the tissue-specific identification of overexpression spots in the respective SOM-images followed by the identification of spot- and tissue-specific lists of gene sets and their aggregation into one global lists using the top-three gene sets from each individual list. The only difference refers to the expression-dependent GSZ-score (Eq. (10)) which is used instead of the expression-independent HG-score (Eq. (8)).

Figure 9b shows the GSZ-enrichment heatmap obtained from the aggregated list of all relevant spots. The obtained number of 64 gene sets exceeds the 48 gene sets in the HG-enrichment map in Figure 9a indicating the increased diversity of the GSZ approach. It can be adjusted by using stricter or more lax thresholds in the GSZ- and/or HG-mappings for the number of selected top-gene sets per spot, respectively. Both heatmaps reveal clusters of molecular characteristics which can be clearly assigned to selected tissue types, e.g. nervous processes to nervous tissues (cluster A in Figure 9) and muscle-related function to muscle tissues (cluster B). Table 1 lists the HG- and GSZ-enriched gene sets associated with the main spots.

In Additional file 1 we further disentangle the obtained GSZ-lists for the three spots selected in the bar plots in Figure 7b to illustrate the specifics of GSZ-enrichment analysis. Our standard algorithm applies the ‘top-three’ criterion, i.e. it selects the three top gene sets of each local spot list and merges them into the global list of gene sets which is further used to characterize the functional context of gene expression in the different tissues. This approach equally weights each spot in terms of the number of selected gene sets and thus it ensures that each spot-feature is equally represented in the resulting global list. Alternatively one can generate a global list of gene sets ranked according to their significance of enrichment in each of the tissues and cut this list using appropriate criteria. Results of this approach are presented in Additional file 1 and 6. The enrichment lists are very similar compared with those obtained from the ‘top-three’ selection criterion.

In summary, HG- and GSZ-enrichment maps based on the ‘top-three’ selection criterion provide an overview about the most important gene sets in the experimental series studied. For the more detailed analysis we recommend using full lists of gene sets for each spot which are provided as additional material in the spot-reports as described below.

### Overexpression maps and profiles of selected gene sets

**Figure 10:**
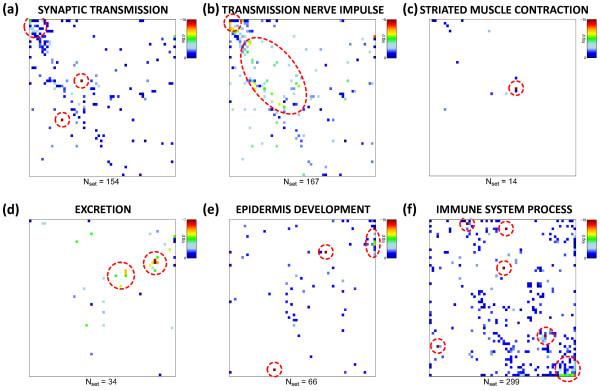
**Overrepresentation maps of six selected gene sets containing between N**_**set**_**= 157 and 472 genes.** Overrepresentation in each tile of the mosaic is calculated in units of log(p_HG_) using the hypergeometric distribution and color-coded (significance: maroon>red>yellow>green>blue). White areas indicate metagenes not containing genes from the respective set). Strongest overrepresentation of the different gene sets is found in different regions of the SOM (see red circles). Overrepresentation can be concentrated within one or a few adjacent metagenes (e.g. muscle contraction, panel c) or spread over different disjunct regions of the map (immune system, panel f).

In the previous subsections we applied ‘spot-centered’ gene set enrichment analysis to extract the most relevant functional gene sets in each tissue sample. One can also pursue a ‘gene set-centred’ approach and map the overrepresentation of one selected gene set in each tissue-specific mosaic image. Particularly, we estimate the degree of overrepresentation of this gene set in each metagene minicluster using the hypergeometric (HG-) distribution. It provides an overrepresentation p-value for each metagene and each gene set considered. Then the distribution of p-values is visualized in the same two-dimensional mosaic which was used for the original expression images. Figure 10 shows overrepresentation maps of gene sets selected from each spot in Table 1. Overrepresentation is observed in different regions of the map, for example in the top left and bottom right corner for genes related to ‘synaptic transmission’ and to ‘immune system process’, respectively. The examples also show that overrepresentation is either strongly localized in one region of the map (e.g. for ‘striated muscle contraction’ or, to a less degree, for ‘synaptic transmission’ and ‘immune system process’) or it spreads over wider areas of the SOM (e.g. for ‘transmission of nerve impulse’). Note that this overrepresentation map applies to all samples studied owing to the fixed gene composition of the metagene clusters.

**Figure 11:**
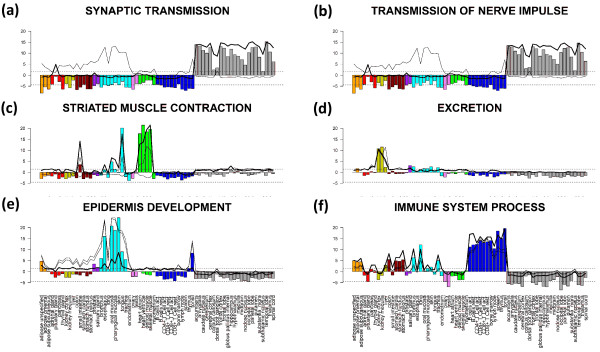
**Overexpression profiles of selected gene sets (bar plots, compare with Figure **10**).** The bars are colored in accordance to the color-codes of the different tissue categories. They are scaled in units of the GSZ-score (left axis). The horizontal dotted lines mark the fdr=0.2 significance threshold estimated from the p-value distribution of the GSZ-score. The inserted curves show the log FC-expression profiles of the top-three metagenes of strongest enrichment of the respective gene set.

One can also apply an orthogonal approach to characterize the ‘enrichment’ profile of a selected gene set in all tissues studied. Our approach makes use of the full list of genes and calculates the GSZ-score for the gene set of interest in all tissues. In this special case the GSZ-score estimates overexpression in terms of the normalized difference between the mean expression averaged either over the gene set of interest and over the full list of genes (see Eq. (15)). The bar plots in Figure 11 show overexpression profiles of the selected gene sets. The gene sets are strongly and consistently overexpressed in different tissue categories. For example, the profiles of ‘synaptic transmission’ and ‘transmission of nerve impulse’ are strongly overexpressed in nervous tissues and underexpressed in virtually all non-nervous tissues. Contrarily, ‘immune system process’-genes show a more heterogeneous expression pattern in the non-nervous tissues with ‘local’ over- (especially in immune systems tissues) and underexpression characteristics while remaining strongly underexpressed in the nervous tissues. Genes related to muscle contraction are naturally overexpressed in muscle tissues but also in tongue which also contains muscle tissue. Note also that the gene set ‘epidermis development’ is overexpressed in epidermal tissues and in tonsil assigned to tissues of the immune system.

The curve plots inserted in all panels of Figure 11 show the expression profiles of the topmost three enriched metagenes containing the respective gene set. Most of these metagene expression profiles are very similar compared with the respective GSZ-overexpression profiles. Hence, representative profiles of the selected metagene miniclusters of co-regulated real genes well agree with the expression profiles of functionally related sets of genes which have been collected independently. This result supports the ‘guilt-by-association’ principle which states that coexpressed genes are likely to be functionally associated because biological processes are governed by coordinated modules of interacting molecules [22].

The ‘guilt-by-association’ principle, in turn, implies the ability to define either new gene sets using selected metagene-miniclusters or to verify and/or to amend existing ones. Such verification can address the distribution of the single genes of a selected gene set over different regions of the SOM (see, e.g. Figure 10) to prove their set membership by independent methods. On the other hand, spot-members not assigned to any gene set constitute potential new candidates for those gene sets which are highly enriched in the respective spot. For example, the tissue specific spots A (nervous system tissues), B (muscle tissues) and F (immune system tissues) contain about 30% - 40% genes which are not assigned to any of the gene sets tested and about 50% genes which are members of gene sets not listed at the top of the list (details are given in Additional file 1). These genes constitute potential candidates for further verification of their functional context.

Based on our spot analysis we define tissue-specific gene sets from spots that are clearly assigned to selected tissue categories. The single genes of each spot are filtered using a correlation threshold for mutual correlations between the single gene and metagene profiles: Only genes are considered with Pearson correlation coefficient larger than 0.8. The defined gene sets are available in Additional file 4.

### Zoom-in analysis

We applied so-called, zoom-in‘ SOM analysis to study the expression profiles of subgroups of samples such as nervous and immune system tissues with enlarged resolution as described previously [6]. The zoom-in maps were trained using reduced sets of tissue samples but the same number of tiles of the SOM-mosaic. They show ‘new’ textures of characteristic over- and underexpression spots which reflect the expression profiles of the tissues of interest more in detail than the original SOM. In the supplementary material (Additional file 1) we present the results of global overrepresentation and of local GSZ-enrichment analysis applied to the respective subgroups of tissues. The zoom-in analysis of nervous tissues, for example, provides clusters of genes related to signal transduction and replication which are not clearly detected in the original maps. Both approaches, global overrepresentation and local GSZ-enrichment analysis, provide consistent results. In the additional material we provide also overrepresentation maps and overexpression profiles of the same gene sets shown in Figures 10 and 11, respectively, to illustrate re-distribution of gene sets after zoom-in.

### SOM-mapping of strongly expressed, absent and housekeeping genes

The gene sets studied in the previous subsections are chosen from GO-categories. They are subsequently processed to estimate their enrichment in overexpressed spot-clusters of co-regulated genes taken from the SOM mosaics. Gene sets can also be collected by applying alternative criteria such as the consistent high or weak expression of the selected genes in all samples. The population mapping of these sets into the SOM mosaic then specifies the activity of the respective genes in different areas of the map. Gene function of these sets can be specified using GO-overrepresentation analysis as described above. However, such global expression criteria itself lend to define groups of genes related to specific functions such as housekeeping gene activity. Housekeeping genes are thought to be by nature significantly expressed in all somatic cells under all circumstances because their gene products are required for the maintenance of basal cellular function (see, e.g., [24,25] and references cited therein). In addition to housekeepers we select special sets of highly expressed (using differential expression and ranking criteria) and of absent (i.e. consistently not or weakly expressed genes) to obtain information about additional aspects of genome-wide transcriptional activity which complements the functional analysis of tissue-specific overexpressed and co-regulated gene sets discussed above (see Table 2 for an overview; the genes of these sets are given in Additional file 5). 

**Table 2:** Special gene sets

	**Gene set**^**a**^	**Selection criterion**	**# of genes**	**Top three overrepresented GO-sets**^**b**^
**a**	Highly expressed	Top ranked expression in the global overexpression list	2,227 (10%)	Cation homeostasis, chemical homeostasis, multicellular organism development
**b**	Highly ranked	Top ranked in the global rank product list ^c^	2,227 (10%)	Anatomical structure morphogenesis, axiogenesis, cell migration
**c**	Inactive (consistently not or weakly expressed)	Member of the N-range of the hook curve, absent in all tissues	688	Receptor activity, signal transduction, plasma membrane
**d**		Present call parameter pc = 0 in all tissues	1,156	Receptor-protein signaling pathway, neurological system process, signal transduction
**e**	Housekeepers (consistently expressed)	Not member of the N-range of the hook curve, present in all tissues	3,561	Anti-apoptosis, apoptosis, cell development, RNA processing, DNA/RNA binding, DNA metabolic process, metabolic process, transcription, translation ^d^… .
**f**		Present call parameter pc = 1 in all tissues	3,167	see e
**g**		Top ranked in mean expression list averaged over all tissues	2,227 (10%)	Macromolecular complex assembly, nucleic acid metabolic process, regulation of cellular metabolic process
**h**		Taken from ref. [31], criterion analogous to g	852	Cellular macromolecule metabolic process, cellular protein metabolic process, protein metabolic process

^a^ gene lists are given in Additional file 5.

^b^ HG-enrichment, lists are given in Additional file 5.

^c^ details are given in Additional file 1.

^d^ about 150 gene sets (see Additional file 5 and Table 2).

**Figure 12:**
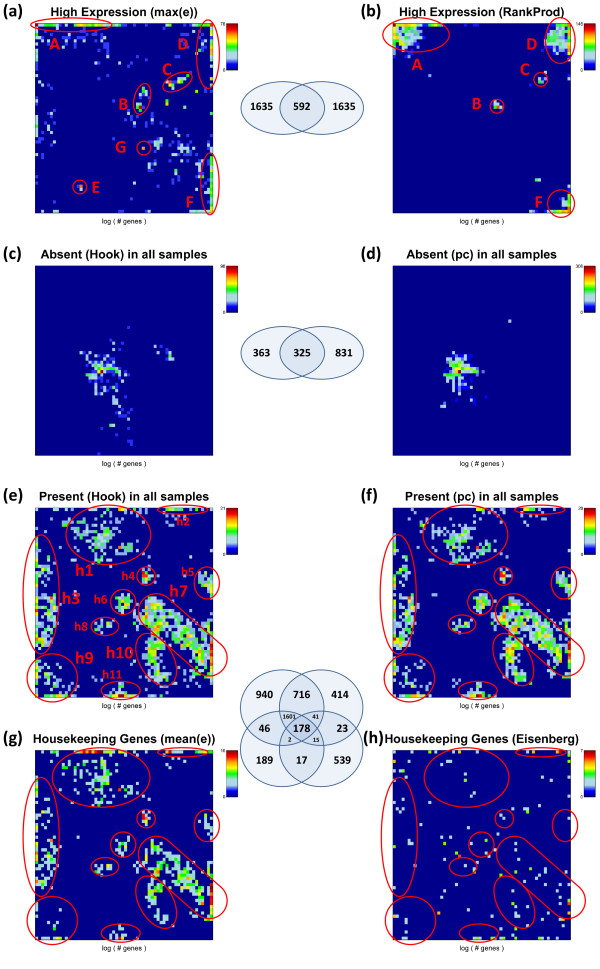
**Population maps of special gene sets: Genes of highest expression (top 10%) preferentially accumulate in a few metagenes in spots A – F (spots are assigned in agreement with Figure **10**) whereas the consistently absent genes (~3-5% of all genes) are found in the area of minimum variability (see variability map in **[6]**).** Housekeeping genes selected as consistently present in all tissues (not-absent, ~15% of all genes) and as the top 10% most stable expressed genes are compared with the set of housekeeping genes taken from ref. [31]. The gene sets enriched in selected highly populated spots (h1 – h11) are given in Table 2. The Venn diagrams show the overlap between different gene sets as illustrated.

**Figure 13:**
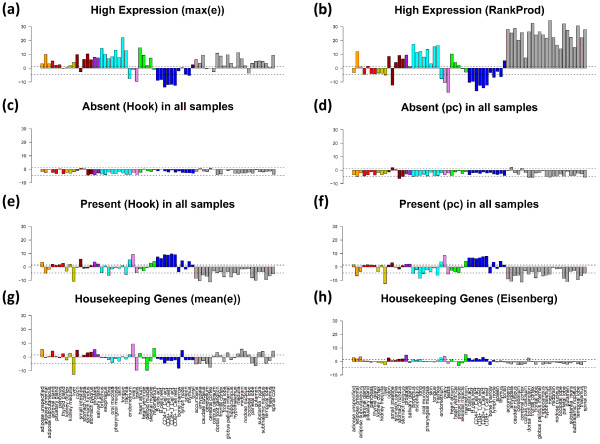
**GSZ-overexpression profiles of the special gene sets defined in Table **1**.**

We analyze the SOM population patterns, the tissue-wide overexpression profiles and also GO-set overrepresentation of these special gene sets. Figures 12 and 13 show the population maps of these gene sets and their GSZ-overexpression profiles, respectively. Highly expressed genes were selected by taking the top-10% genes either from the global overexpression list (panel a) or from the global rank product list (b, see Additional file 1 for details and also [26]). These criteria select genes either from a larger number of overexpression spots (e.g. spots A, C, D, H; compare Figure 7 and Figure 12a and b) or from only a few ones (Figure 12b). Note that only about one fourth of the genes in each of the sets are commonly found in both sets due to the different criteria which select either maximum expressed genes or consistently top ranked genes. The overexpression profiles in Figure 13 (panel a and b) reveal that the rank criterion (b) more strongly weights highly expressed genes from nervous tissues than the alternative high expression criterion (a). On top of the HG-overrepresentation lists one finds gene sets related to homeostasis for high expression (a) and to morphogenesis and cell migration for consistently highly ranked genes (b) (see Table 2). Note that the ranking criterion weights the effect of tissues according to the number of samples of the respective tissue category. The relatively large number of nervous tissues obviously biases the particular genes selected using the ranking criterion towards genes involved in nervous function. 

The expression of ‘absent’ genes per definition falls below the detection threshold for specifically hybridized probes in the microarray measurement. One can detect the respective genes using two different but closely related criteria (see rows c and d in Table 2). The first one extracts these genes directly after single-array intensity calibration using the hook method [27,28] whereas the second one is based on the present-call parameter of each gene which was obtained after applying background correction and chip-to-chip normalization to all arrays of the series (see the methods section in [6] for details). The latter criterion selects about twice as much genes as the former one with only moderate overlap between both groups (Table 2 and Figure 12). Both criteria however provide very similar characteristics of absent and weakly expressed genes despite these differences (see panels c and d in Figures 12 and 13): the genes selected strongly accumulate within one localized area near the centre of the SOM which has been assigned to virtually invariant genes. The GSZ-profiles support this result: They show relatively constant profiles for these sets which contain enriched populations from GO-sets related to receptor activity and signal transduction (Table 2).

The criteria e and f (Table 2) essentially invert the previous selection of absent genes. They select genes which are significantly expressed in all tissues studied. These genes widely distribute over different regions of the SOM mosaics forming several highly populated ‘hot spots’ (see panel e and f in Figure 12). Spots of high tissue specificity are virtually not selected by these criteria as expected (compare with Figure 7). Interestingly, these consistently present genes are overexpressed in immune system tissues and underexpressed in nervous tissues, a pattern which basically inverts the respective profiles of the highly expressed genes in these two tissue categories (compare e and f with a and b in Figure 13).

Criteria e and f essentially meet the conditions for housekeeping genes (see above). We applied an alternative criterion which chooses 10% of the genes of highest mean expression log-averaged over all tissues. Most of the genes selected are common members also in the sets e and f. These three sets consequently possess very similar characteristics (see Figures 12 and 13). For comparison we included a list of housekeepers taken from a previous microarray study [24]. The respective selection condition essentially agrees with our criterion d. However it was applied to an alternative tissue data set which was studied using a previous generation of HGU95a- GeneChip arrays [29,30]. We reanalyzed this data set and found that it contains a much higher fraction of absent genes in most of the tissues (data not shown). This difference presumably explains the relatively small number of housekeepers detected in this data set. Despite this difference it reveals a similar overexpression profile compared with our alternative sets.

HG-overrepresentation analysis of the housekeepers provides functional gene sets related to basal cell activity such as ‘metabolic process’, ‘transcription’, ‘translation’ and ‘RNA processing’. Note that the housekeepers distribute over several separated spot-like areas in the SOM mosaic which partly contain enriched fractions of the same gene sets such as ‘cytoplasm’ found on top of gene set lists in the spots h1-3, 5, 11 (see Table 3). Other gene sets accumulate in single or only a few spots only, for example ‘nucleus’ in h3, h9 and h10; ‘mitochondrion’ in h4 and ‘lipid binding’ in h5. The SOM approach thus enables to further disentangle larger groups of genes such as housekeepers into subgroups of more specific function. For example, housekeepers related to nucleic acid processing accumulate in spots h7, h9 and h10 whereas genes related to actin functioning in h2. Note also that the spots of housekeepers discussed are still located in regions of relatively highly variable and thus specific metagene profiles.

**Table 3:** GO-overrepresented gene sets in SOM-spots of highly populated housekeeping metagenes

**Spot**^**a**^	**# of genes**	**Top overrepresented gene sets**
**h1**	333	Cytoplasm, enzyme regulator activity, vesicle mediated transport, establishment of localization
**h2**	74	Cytoplasm, oxidoreductase activity, actin binding, endoplasmic reticulum, cytosol
**h3**	418	Cytoplasm, macromolecular complex, nucleus, protein metabolic process, protein complex
**h4**	89	Oxidoreductase activity, cytoplasm, mitochondrion, envelope, organelle
**h5**	91	Cytoplasm, Golgi apparatus, cofactor catabolic process, lipid binding, microsome
**h6**	101	Protein complex, macromolecular complex, cytoplasm, protein catabolic process
**h7**	775	Biopolymer metabolic process, biosynthetic process, nucleic acid, RNA processing
**h8**	50	Protein metabolic process, endosome, cellular metabolic process, phosphatase activity
**h9**	176	Nucleus, biopolymer metabolic process, nucleic acid / RNA metabolic process
**h10**	253	Biopolymer metabolic process, mRNA metabolic process, RNA processing, nucleus
**h11**	118	Cytoplasm, proteasome complex, cellular protein metabolic process, protein metabolic process

^a^ spots are defined in Figure 12e.

In conclusion, global expression criteria represent an alternative option for selecting metagenes and spots of metagenes with functional impact. These criteria complement the overexpression criteria discussed above. Note for completeness that both options can be combined, for example, to mask absent genes in the overexpression SOM to exclude noisy and thus presumably irrelevant genes.

### Reports

Our SOM approach enables views from different perspectives on large sets of high dimensional data. They include overview characteristics which address similarity relations between different samples and the detailed description of the expression pattern in each of the samples studied as well. Moreover, differential expression analysis identifies ordered lists of over- and underexpressed genes taken either from the full ensemble of all genes available or from subensembles selected from metagene clusters of co-regulated genes. Information about the functional context is extracted by applying enrichment analysis to the different gene lists.

We designed a set of standard PDF-reports which allows the systematic browsing in the full set of results. Details are given in the supporting text (Additional file 1). The whole report is organized into several main topics each of them contains a series of documents. The reports of this tissue-study can be downloaded from our website (http://som.izbi.uni-leipzig.de).

## Summary and conclusions

SOM machine learning transforms large and heterogeneous sets of expression data into mosaic images which visualize sample-specific over- and underexpression in terms of characteristic textures. This view is very intuitive to identify modules of correlated and differentially expressed genes in terms of well defined colored spots. SOM analysis basically rearranges and condenses the primary information of gene expression without filtering. It thus preserves the whole information content of the original data set despite the dimension reduction used to visualize the most essential expression profiles inherent in the data.

This primary information together with the respective gene annotations is further processed in differential expression analysis using three alternative scores which place emphasis either exclusively on the fold change of gene expression or, in addition, on the precision of the measurement.

SOM analysis provides special advantage to generate local lists of genes taken from selected spots of the map. Thus, the impact of differential expression can be studied not only in a sample-specific fashion but also for selected subgroups of co-regulated genes. The alternative scores studied provide slightly different but mostly consistent rankings for lists containing up to a few dozen genes. The FC-, WAD- and shrinkage t-scores tested are rather complementary measures than competitive ones providing information which mutually supplements each other with specific advantages and disadvantages.

To extract the functional context of spot- and metagene-related lists of single genes we applied overrepresentation- and overexpression analysis, and a combination of both with respect to pre-defined gene sets of known functional impact. Overrepresentation analysis combines the criterion membership in a gene set with that of co-(i.e. correlated-) expression in a series of samples whereas overexpression analysis compares the mean expression of genes from the set with that of all genes. The mapping of overrepresentation of a selected gene set into the SOM mosaic provides a ‘functional’ map showing areas which are potentially relevant in this context. Alternatively, one can screen the degree of overrepresentation of a large number of gene sets in a selected metagene spot to discover its potential function. Both complementary views provide a link between the tiles and/or spots of the SOM mosaic and their potential molecular function.

Overexpression analysis of a selected gene set, on the other hand, profiles a selected molecular function across the different samples studied, for example, to identify tissues with highly active or inactive genes from the set of interest. The gene set enrichment approach was applied to discover the functional context of the metagene overexpression spots in a sample specific fashion by estimating significance using either the hypergeometric statistics or the gene set enrichment Z-score with similar results in both cases. GSZ-enrichment however tends to select more diverse lists of gene sets because it explicitly takes into account the expression profile of the associated genes. The use of multiple options of ranking scores for differential expression and for gene set functional analysis enable to test the robustness of single gene and gene set rankings with potential consequences for their biological interpretation.

The tissue related spots of the SOM typically contain enriched populations of gene sets corresponding to known molecular processes in the respective tissues in the actual case study. SOM spot-clustering implies the ability to define either new gene sets using selected SOM spots or to verify and/or to refine existing ones. In addition to overexpression criteria for selecting SOM spots (given in units of expression differences) we study absolute ones (given in units of expression values) which allow identification of alternative sets of housekeeping genes and of consistently-high or -low expressed genes.

The present paper thus extends our previous study and adapts these methods for feature selection and for mining the functional context to the SOM-data. Beyond these methodical issues our case study provides insights into tissue specificity of gene expression. For example, genes involved into nervous function show an antagonistic expression patterns with high expression in nervous tissues and low expression in nearly all non-nervous tissues studied. In contrast, genes related to immune system response are specifically upregulated not only in immune system tissues but also in other tissues (e.g. adipose and digestion) thus reflecting commonly activated immune processes. Also specific combinations of different gene functions can be easily detected by our methods such as the combined activation of genes related to immune response and to epidermis development in tonsils. Using our spot-selection method we provide a series of tissue specific gene sets which can be applied, for example, to study tissue-specific factors in different diseases. In addition to the detailed profiling of functional gene sets in human tissues our SOM-analysis enables diversification of general categories of genes such as highly expressed and permanently expressed ones. Highest expression levels are observed in epithelial, digestion, exocrine and partly muscle tissues. Permanently expressed ‘housekeepers’ can be split into different subgroups related, e.g., to protein metabolism, mitochondrial or transcriptional activity due to tissue-specific modulation of their expression levels.

Application of SOM-based analysis to the full set of 67 tissues thus provides the comprehensive and detailed characterization of the transcriptome of human tissues as seen by GeneChip microarrays. Our study produced an extensive collection of results which are provided as supplementary reports to illustrate the potency of the method and also as data base for further studies in the context of gene regulation in different tissues and its dysfunction. The methods of differential gene expression and enrichment analysis are implemented in the R-program ‘oposSOM’ available as CRAN package.

## Data and methods

### Microarray data and SOM-cartography

The raw microarray data and their primary and secondary analysis in terms of calibration, normalization and SOM-cartography was described in [6]. In short: Gene expression profiles were downloaded from Gene Expression Omnibus under accession number GSE7307 (http://www.ncbi.nlm.nih.gov/geo/query/acc.cgi?acc=GSE7307). The data set consists of 677 human tissue samples measured with the Affymetrix HG-U133 plus 2.0 array. We selected 187 of these samples derived from 67 different tissues for further analysis.

Microarray intensities were transformed into expression values, E_g,m,r_, using hook calibration [27,28] and quantile normalization. The indices assign the gene (g = 1…N), the tissue (m = 1…M) and the replicate (r = 1…R_m_) where the number of replicates can vary between the tissues. The logged expression values of each gene, $e_{g,m,r}=\log_{10}E_{g,m,r}$, are averaged over the replicates, $e_{g,m}\equiv {〈e_{r,g,m}〉}_{r}$ (angular brackets denote arithmetic averaging), and transformed into differential expression values, $\Delta e_{g,m}\equiv e_{g,m}-e_{g}$, with respect to the mean expression of each gene averaged over all tissues studied, $e_{g}\equiv {〈e_{g,m}〉}_{m}$.

Subsequently self organizing maps (SOM) machine learning was applied to all differential expression data. The algorithm initializes K weight vectors of dimensionality M given by the number of conditions studied. The elements of the weight vectors can be interpreted as expression profiles of prototypic genes which are called metagenes in our application.

The metagenes are arranged in a rectangular grid (K = 60 × 60 tiles) and initialized using linear initialization [31,32]. Here, the metagene profiles are determined along the linear subspace spanned by the two eigenvectors with largest eigenvalues of the input data. This approach is similar to *principal component analysis (PCA)*, attempting to cover the greatest variability of the data. Due to linear scale used, adjacent metagene profiles in the grid are more similar than more distant ones and the most distant metagenes roughly cover the whole range of input data. Linear initialization is effective because it reduces the number of required iterations required for training (see below) compared, e.g. with random initialization. It largely avoids topological defects and it is deterministic, i.e., repeated training runs of the same data with slightly varied grid dimensions provide reproducible and comparable results.

After initialization the SOM is trained using an iterative algorithm. In each iterative step a gene is picked from the gene list and its vector of differential expression $\Delta e_{g,m}$ is compared with the metagene profiles using the Euclidean distance as similarity measure. The metagene profile of closest similarity is then modified, so that it more closely resembles the expression profile of the selected gene. In addition, the neighboring metagene vectors in the two-dimensional grid closest to this metagene are also modified, so that they also resemble the gene's expression vector a little more closely. This process is applied to all genes and repeated about 250,000 times. The radius of considered neighbors is decreased with progressive iteration which modifies fewer metagene vectors by smaller amounts, so that the metagene vectors asymptotically settle down. The resulting map becomes organized because the similarity of neighboring metagenes decreases with increasing distance in the map. Finally each gene is associated to its best matching metagene, building up miniclusters of similar gene profiles each represented by the metagene profile.

In the final SOM each ‘single’ gene is assigned to the metagene vector of closest similarity. It consists of regions of similar metagene profiles each of them represents a minicluster of single genes with correlated expression profiles. High-variable metagene profiles arrange near the edges of the map about a central region of less variable metagenes. In the next step, the SOM is ‘stained’ using an appropriate color code: Particularly, each sample studied provides one SOM-image which ‘portrays’ its expression landscape. Each tile of the mosaic is colored according the value of the respective metagene in the sample chosen. The distance similarity metrics and the training algorithm used gives rise to characteristic sample-specific spot patterns where each spot includes several adjacent metagenes. Sample-specific over- and underexpression spots are selected among all metagenes using a 98% and 2% quantile criterion, respectively.

### Differential expression scores

A large multitude of various methods have been developed in the last decade to assess statistical significance of differential expression in microarray data analysis (see, e.g., the overview given in [33] and the references cited therein). Most statistical methods aim at generating ranked lists of single genes which are differentially expressed according to a certain level of significance. Microarray data are very noisy and prone to systematic errors [34-39]. The proper estimation of the level of precision constitutes therefore one basal problem in significance analysis, especially if only a few replicates are available. Another problem is raised by the highly multivariate character of the data which requires suited concepts to control significance in multiple testing.

In this study we estimated differential expression of individual genes using three alternative scores:

1. The fold change (FC) simply estimates the expression change in logarithmic scale, log ${}_{\log}FC_{g,m}\equiv \Delta e_{g,m}$.

2. The weighted average difference (WAD)-score,

(1) $$WAD_{g,m}=w_{g,m}\cdot \Delta e_{g,m}\;with\;w_{g,m}=\frac{\Delta e_{g,m}-\min\left(\Delta e_{g,m}\right)}{\max\left(\Delta e_{g,m}\right)-\min\left(\Delta e_{g,m}\right)}$$

is a fold-change based measure well performing in differential expression analysis [14,15]. The main idea behind the WAD method assumes that relevant marker genes tend to have high expression levels, i.e. ‘strong signals are better signals’ in the gene ranking problem [16,34,40]. This assumption accounts for the fact that the experimental error of expression values inflates at small expression levels in logarithmic scale [41-43]. Note that the weighting factor in Eq. (1) can be expressed as a function of the absolute expression values as in the original paper of Kadota et al. [14], $w_{g,m}=\left(e_{g,m}-\min\left(e_{g,m}\right)\right)/\left(\max\left(e_{g,m}\right)-\min\left(e_{g,m}\right)\right)$, showing that the weighting factor linearly scales with the expression level of the gene.

3. The shrinkage t-score,

(2) $$t_{g,m}=\frac{\Delta e_{g,m}}{SE_{g,m}^{\mathrm{diff}}}\;with\;SE_{g,m}^{\mathrm{diff}}=\sqrt{\frac{{\left(\sigma_{g,m}^{\mathrm{shr}}\right)}^{2}}{R_{m}}+\frac{{〈{\left(\sigma_{g,m}^{\mathrm{shr}}\right)}^{2}〉}_{m}}{\sum_{m-1}^{M}R_{m}}}\approx \frac{\sigma_{g,m}^{\mathrm{shr}}}{\sqrt{R_{m}}}$$

accounts for the standard error of the expression values of each gene in replicated measurements. Our shrinkage statistics was defined in Eq. (2) in analogy with previous approaches [44-46]. Here SE_g,m_^diff^ denotes the standard error of differential expression of gene g measured under condition m. To estimate the standard error in Eq. (2) we first calculate the standard deviation of the log-expression values using the available replicates, $\sigma_{g,m}\equiv \sqrt{{〈{\left(e_{r,g,m}-e_{g,m}\right)}^{2}〉}_{r}}$. These values are then plotted for each sample as a function of the logged expression degree, e_g,m_, and locally pooled over a moving window of a few hundred neighboring values. The obtained locally pooled error (LPE) estimates the mean standard deviation as a function of the expression, $\sigma_{\mathrm{LPE}}\left(e_{g,m}\right)$. It is combined with the individual standard deviation for each gene to provide the shrinkage error estimate used in Eq. (2)

(3) $$\sigma_{g,m}^{\mathrm{shr}}=\sqrt{\lambda \cdot {\sigma_{g,m}}^{2}+\left(1-\lambda \right)\cdot \sigma_{\mathrm{LPE}}{\left(e_{g,m}\right)}^{2}}$$

The parameter λ (0 ≤ λ ≤ 1) scales the degree of shrinking $\sigma_{g,m}$ towards $\sigma_{\mathrm{LPE}}$.

The shrinkage t-statistics was developed in the framework of James-Stein analytic shrinkage and applied in different modifications in gene expression analysis (see [44] and references cited therein). The basic idea behind Eq. (3) assumes that the error estimate based on σ_g,m_ alone might be very imprecise, e.g. if only a few replicates are available. The resulting large ‘error of the error’ leads to highly uncertain naive t-scores associated with large false positives rates (see Eqs. (2) and (3) with λ = 1).

It has been suggested previously that estimates of the variance from individual genes is questionable [25,38,46-49]. Yet accurately estimating variability of gene expression is essential for correctly identifying differentially expressed genes. Additional information may be gained by combining variance estimates across all or part of the experiment. Such information borrowing methods that exploit this information are able to improve the results [16,48,50]. Particularly, local-pooled-error estimates for evaluating significance of each gene’s differential expression have been shown to effectively identify significant differential expression patterns with a small number of replicated arrays [50].

To get more precise error estimates, the shrinkage t-score makes therefore use of the fact that the variability of microarray expression values is governed by methodical factors which allow to express the measurement error as a function of the expression level [43,51]. This error can be estimated with high precision using the LPE averaging approach. Finally, Eq. (3) combines the pooled and the gene-specific error to take into account both, individual and common factors. Shrinkage t-scores consistently lead to accurate gene rankings which might outperform simple t-statistics or FC-scores [44].

In the supplementary text (Additional file 1) we address details of the error characteristics of the different tissue samples studied in terms of their LPE-functions and mean LPE-values. It is shown that the error level depends on the tissue type. For example, adipose tissues and tissues related to digestion show nearly twice as large gene-related error levels than tissues of sexual reproduction, of exocrine function and partly of homeostasis.

### Significance analysis

The shrinkage t-statistics (Eq. (2)) transforms into p-values characterizing the significance of differential expression for each gene assuming Student’s t-distribution. The obtained density distribution for the p-values of all genes in one selected tissue, ρ(p), meets the normalization condition $\int_{0}^{1}\rho \left(p\right)\cdot dp=1$. Examples for selected tissues of different mean error level are shown in Additional file 1. Under the null hypothesis one expects a uniform distribution, ρ_0_(p) = 1, whereas the alternative hypothesis will produce a skewed distribution, ρ_DE_(p), decaying with increasing p because differentially expressed genes tend to cluster closer to p = 0 [52]. In the general case, the observed distribution can be interpreted as the superposition of two components due to differentially and not-differentially expressed genes, $\rho \left(p\right)=\rho_{\mathrm{DE}}\left(p\right)\left(1-\eta_{0}\right)+\rho_{0}\left(p\right)\eta_{0}$, where η_0_ is the fraction of non-informative ‘null’-genes among all genes considered [52,53]. It was derived using the “fdrtool” R-package [54] under the assumption of vanishing differential expression at p = 1, ρ_DE_(1) = 0, giving rise to $\rho \left(1\right)=\eta_{0}$[55]. “fdrtool” was further used to calculate false discovery rates (FDR) to control the number of false discoveries:

(4) $$fdr\left(p\right)=\frac{\eta_{0}}{\rho \left(p\right)}\;and\;Fdr\left(p\right)=\frac{\eta_{0}\cdot p}{\int_{0}^{p}\rho \left(p\right)\cdot dp}$$

Here fdr and FDR denote the local and tail area-based FDR estimates, respectively. The latter Fdr(p)-values provide a cumulative estimate of FDR referring to all genes on top of a list with p-values p’ ≤ p whereas fdr(p) estimates the FDR of a selected gene with p’ = p [56]. For a monotonically decaying total density ρ(p) both, fdr(p) and Fdr(p), are increasing functions which well correlate in the intermediate p range. The local FDR-estimate however systematically exceeds the tail-based one, fdr(p) ≥ Fdr(p), at intermediate and large values of argument (see the examples shown in Additional file 1). Their limiting values at p = 0 and 1 are given by the equations Fdr(0) = fdr(0), Fdr(1) = η_0_ and fdr(1) = 1, respectively.

The total fraction of differentially expressed and thus informative genes per sample can be estimated using the background level of the respective p-value distribution,

(5) $$\%DE=1-\eta_{0}$$

%DE decreases with increasing error level and with increasing FDR at a selected p-value (p = const). In the supplementary text (Additional file 1) we studied the relation between different error measures and the fraction of differentially expressed genes more in detail. The number of differently expressed genes meeting a given significance criterion is governed by the error level of the expression measures which, in turn, systematically varies between the different tissues and tissue types. On the other hand, we found that data transformation after preprocessing and normalization can mask mutual relations between the error measures including also the fraction of differentially expressed genes.

### Comparing alternative gene lists

Each of the alternative scores of differential expression provides an ordered list of differentially expressed genes per tissue which are ranked, for example, with decreasing absolute value of the score. The similarity between two lists of length r can be described using the ‘correspondence at the top’ (CAT(r)) plot. It shows the fraction of genes commonly found at the top of both lists up to rank r [57]. Note that ‘null-correspondence’ for randomly ranked genes can be estimated using the hypergeometric distribution and Eqs. (8) and (12) (see below). The respective CAT(r) value is given by the probability that a selection of N_set_ = r genes is found among the top N_list_ = r positions of a total list of length N, p_HG_ = r/N (see below).

The CAT-plot thus estimates the agreement between two lists irrespective of the particular score values of the genes in the lists. For example, two lists can agree with CAT = 0.5 but differ with respect to the significance level of the remaining 50% of genes. To assess this aspect of pairwise list comparisons we define the p-CAT(r) value as the cumulative logged p-values of the t-shrinkage score of the r genes at the top of the list obtained from the t-shrinkage or from the alternative scores. The p-CAT value of the t-shrinkage score provides the lower limit because it per definition is ranked with increasing p value. The corresponding p-CAT value of an alternative score such as the WAD-statistics consequently judges the degree of discordance with respect to the t-shrinkage statistics. It is given as the difference $\Delta p-CAT=p-CAT{\left(r\right)}_{alternative\;score}-p-CAT{\left(r\right)}_{t-shrinkage}$.

Finally, the rank-correspondence (RC) plot illustrates the agreement between two lists by color-coding each position either in red or in green: green symbols assign ranks which agree with ±20 positions in the alternative list whereas red ranks do not.

### Differential expression of metagenes

SOM machine learning identifies k = 1…K metagenes where each of them is representative for a minicluster of n_k_ real genes of correlated expression profiles. A simple natural approach of combining significance information for a group of genes is to calculate the mean characteristics averaged over the group members. Accordingly, we calculate the mean p- and fdr- (Fdr) values for each metagene via arithmetic averaging,

(6) $${〈S〉}_{k,m}=\frac{1}{n_{k}}\sum_{g=1}^{n_{k}}S_{g\in k,m}$$

where S_g,m_ = t_g,m_, log(p_g,m_), fdr_g,m_ are the single gene significance characteristics of gene g in metagene k and tissue m. Ranking of the averaged characteristics provides ordered lists of metagenes according to their differential expression.

Spots of adjacent metagenes are determined by applying different criteria, such as the mutual correlations between the metagene profiles or their differential expression beyond an appropriately chosen threshold value. For example, metagenes are classified as over- (or under-) expressed, if their expression value exceeds the 98% (or falls below the 2%) quantile-level of the expression range of all metagenes in the particular tissue studied. These spots are characterized by their mean significance characteristics as averages over all genes of the respective spot in analogy with Eq. (6)

(7) $${〈S_{m}〉}_{\mathrm{spot}}=\frac{1}{\sum_{k\in spot}n_{k}}\sum_{k\in spot}\sum_{g=1}^{n_{k}}S_{g\in k,m}$$

### Gene set overrepresentation analysis: integrating concepts of molecular function

Gene set analysis requires the knowledge of predefined gene sets to study their enrichment in gene lists which are obtained from independent differential expression analysis (see [7,8] for a critical review and references cited therein). A large and diverse collection of such sets can be downloaded from the ‘gene-set-enrichment-analysis’-website (http://www.broadinstitute.org/gsea). Particularly, we included in total 1454 gene sets in our analysis according to the GO terms ‘biological process’ (825 sets), ‘molecular function’ (396 sets) and ‘cellular component’ (233 sets). These sets can partly overlap in component genes, and some gene sets are subsets of others due to the hierarchical nature of the GO-systematics [47]. Rather than merge these sets we kept them all in order to maximize the functional annotation conveyed by the gene set names.

We will use the term ‘overrepresentation’ to assign the probability to find members of a given set in a list compared with their random appearance independent of the values of their expression scores. Contrarily, the term ‘overexpression’ will be used to characterize deviations between the mean expression score averaged over the set-members in a list compared with the mean score of all list members independent of their overrepresentation. The term ‘enrichment’ will be used for estimates which combine overrepresentation and overexpression (see below).

Particularly, in gene set overrepresentation analysis, each gene studied is classified according to two memberships leading to a 2 × 2 contingency table for further testing (Table 4): firstly, its membership in the particular set of functionally related genes of length N_set_ and, secondly, its membership in the respective list of differentially expressed genes of length N_list_. The intersection of the set and the list is given by the number of ‘positive’ genes, N_+_. Then, one can estimate overrepresentation of these positive genes using the hypergeometric distribution by calculating the cumulative probability that there is more overlap between the list and the set than would be expected by chance [58-60],

(8) $$p=P\left(n>N_{+}\right)=\sum_{n=N_{+}+1}^{N_{\mathrm{set}}}p_{\mathrm{HG}}\left(n\right)\;with\;p_{\mathrm{HG}}\left(n\right)=\frac{\left(\begin{array}{c} N_{\mathrm{set}}\\ n \end{array}\right)\left(\begin{array}{c} N-N_{\mathrm{set}}\\ N_{\mathrm{list}}-n \end{array}\right)}{\left(\begin{array}{c} N\\ N_{\mathrm{list}} \end{array}\right)}$$

**Table 4:** 2x2 contingency table of the number of genes in different classes for gene set overrepresentation in a list of differentially expressed genes

**# of genes**	**in list**	**not in list**	**total**
in set	N_+_	N_set_- N_+_	N_set_
not in set	N_list_- N_+_	N- (N_list_ + N_set_) + N_+_	N- N_set_
total	N_list_	N- N_list_	N

The obtained p-value estimates the probability to find a stronger overlap between the list and the set by chance than actually detected.

The gene set overrepresentation approach thus considers the joint membership of a gene in a gene set and in an independent list of genes without taking into account the rank and the particular values of the respective test statistics of the genes in the list. For example, it ignores whether a positive gene is found on top or on bottom of the list or whether a gene is strongly or weakly differentially expressed. In contrast, the so-called gene set overexpression approach compares the gene set statistics with the null given by the ensemble of all genes studied (see refs. [8] and [10] for a review). In this case however no enrichment of a set in a sub-ensemble of a gene list is taken into account.

### Gene set enrichment analysis: the GSZ-score

The so-called gene set Z-score (GSZ) merges both options provided by the gene set overrepresentation and the gene set overexpression approaches [10]. Namely, the GSZ method estimates overrepresentation of a gene set in a list using its score statistics, for example, $S_{g\in list}=t_{g\in list}$. It is designed in such a way that members of the list with high values on top of the list more heavily contribute than members with lower values down the list. Particularly, one first transforms the total sum of the score function over the gene list into two components containing members and non-members of the set, $S_{\mathrm{list}}=\sum_{all\;g\in list}S_{g}=S_{\mathrm{list}}^{+}+S_{\mathrm{list}}^{-}$ with

(9) $$S_{\mathrm{list}}^{+}=\sum_{g\in list\;AND\;g\in set}S_{g}\;and\;S_{\mathrm{list}}^{-}=\sum_{g\in list\;AND\;g\notin set}S_{g}$$

Secondly, one defines the regularized Z-value of the differential score, $\Delta S_{\mathrm{list}}=S_{\mathrm{list}}^{+}-S_{\mathrm{list}}^{-}$, of the form (see [10] for details)

(10) $$GSZ=\frac{\Delta S_{\mathrm{list}}-E\left(\Delta S_{\mathrm{list}}\right)}{\sqrt{\lambda \cdot SE{\left(\Delta S_{\mathrm{list}}\right)}^{2}+\left(1-\lambda \right)\cdot S{E_{0}}^{2}}}$$

Here,

(11) $$\begin{array}{c} E\left(\Delta S_{\mathrm{list}}\right)={〈S〉}_{\mathrm{list}}\cdot \left({〈N_{+}〉}_{\mathrm{HG}}-{〈N_{-}〉}_{\mathrm{HG}}\right)={〈S〉}_{\mathrm{list}}\cdot \left(2{〈N_{+}〉}_{\mathrm{HG}}-N_{\mathrm{list}}\right)\;and\;\\ SE{\left(\Delta S_{\mathrm{list}}\right)}^{2}=4\left(\frac{\mathrm{var}{\left(S\right)}_{\mathrm{list}}}{N_{\mathrm{list}}-1}\left({〈N_{+}〉}_{\mathrm{HG}}\cdot \left(N_{\mathrm{list}}-{〈N_{+}〉}_{\mathrm{HG}}\right)-\mathrm{var}\left(N_{+}\right)\right)+{{〈S〉}_{\mathrm{list}}}^{2}\cdot \mathrm{var}\left(N_{+}\right)\right) \end{array}$$

are the expected mean and the standard error of ΔS_list_ for the selected list under the null hypothesis. ${〈s〉}_{\mathrm{list}}=s_{\mathrm{list}}/N_{\mathrm{list}}$ and $\mathrm{var}{\left(S\right)}_{\mathrm{list}}=\frac{1}{N_{\mathrm{list}}}\sum_{g\in list}{\left(S_{g}-{〈S〉}_{\mathrm{list}}\right)}^{2}$ are the mean and the variance of the expression score in the list, respectively. SE_0_ and λ denote the regularization constant and a scaling factor (1 ≤ λ ≤1) which were chosen to stabilize the variance in the denominator of Eq. (10) especially for short lists (see below).

The mean and the variance of positive members of the hypergeometric distribution are

(12) $${〈N_{+}〉}_{\mathrm{HG}}=N_{\mathrm{set}}\frac{N_{\mathrm{list}}}{N}\;and\;\mathrm{var}\left(N_{+}\right)={〈N_{+}〉}_{\mathrm{HG}}\cdot \left(1-\frac{N_{\mathrm{set}}}{N}\right)\left(\frac{N-N_{\mathrm{list}}}{N-1}\right)$$

respectively. The respective mean number of negative members is ${〈N_{-}〉}_{\mathrm{HG}}=N_{\mathrm{list}}-{〈N_{+}〉}_{\mathrm{HG}}$. One gets after inserting Eq. (12) into Eq. (11) for the special case $N,N_{\mathrm{list}}>>1$

(13) $$\begin{array}{c} E\left(\Delta S_{\mathrm{list}}\right)={〈S〉}_{\mathrm{list}}\cdot N_{\mathrm{list}}\cdot \left(\frac{2\cdot N_{\mathrm{set}}}{N}-1\right)\;and\;\\ SE{\left(\Delta S_{\mathrm{list}}\right)}^{2}\approx 4\cdot N_{\mathrm{set}}\frac{N_{\mathrm{list}}}{N}\cdot \left(\mathrm{var}{\left(S\right)}_{\mathrm{list}}\cdot \left(1-\frac{N_{\mathrm{set}}}{N}\right)+{{〈S〉}_{\mathrm{list}}}^{2}\cdot \left(1-\frac{N_{\mathrm{set}}}{N}\right)\cdot \left(1-\frac{N_{\mathrm{list}}}{N}\right)\right) \end{array}$$

Eq. (13) indicates that the standard error in Eq. (10) vanishes for small sets and/or short lists (compared with the total number of genes, i.e. N_list_/N < <1) giving rise to instable estimates of the GSZ-score [10]. Making use of approximation Eq. (13) we chose the regularization constant according to

(14) $$\begin{array}{l} S{E_{0}}^{2}\approx 4\cdot N_{\mathrm{list}}^{\min}\frac{N_{\mathrm{set}}^{\min}}{N}\cdot \left(\mathrm{var}{\left(S\right)}_{\mathrm{list}}\cdot \left(1-\frac{N_{\mathrm{set}}^{\min}}{N}\right)+{{〈S〉}_{\mathrm{list}}}^{2}\cdot \left(1-\frac{N_{\mathrm{set}}^{\min}}{N}\right)\cdot \left(1-\frac{N_{\mathrm{list}}^{\min}}{N}\right)\right)\\ and\;\lambda =1-\min\left(1,\sqrt{\frac{N_{\mathrm{list}}^{\min}}{N_{\mathrm{list}}}\cdot \frac{N_{\mathrm{set}}^{\min}}{N_{\mathrm{set}}}}\right) \end{array}$$

to penalize small lists and sets. N_list_^min^ and N_set_^min^ are minimum settings (typically 5–10) and ${〈S〉}_{\mathrm{list}}\;and\;\mathrm{var}{\left(S\right)}_{\mathrm{list}}$ are the mean and the variance of the significance score in the ensemble of all genes of the list. The ad-hoc estimate of the scaling factor λ ensures that SE_0_ progressively increases with decreasing number of genes in the list and/or set. Obtained GSZ-values were transformed into p-values using a permutation approach which generates the respective null distribution by random rearrangement of genes in the collection of predefined gene sets. One and two tailed tests were applied to assess over- or underexpression and differential expression (i.e., under- *and* overexpression), respectively.

In the following we consider two special cases of the GSZ-score referring to overexpression and overrepresentation, respectively.

Firstly, the GSZ-score can be calculated for the whole gene list with N_list_ = N. Eq. (13) provides for this special case $E\left(\Delta S_{\mathrm{list}}\right){|}_{N_{\mathrm{list}}=N}={〈S〉}_{\mathrm{list}}\cdot \left(2\cdot N_{\mathrm{set}}-N\right)$ and $SE{\left(\Delta S_{\mathrm{list}}\right)}^{2}{|}_{N_{\mathrm{list}}-N}\approx 4\cdot N_{\mathrm{set}}\cdot \mathrm{var}{\left(S\right)}_{\mathrm{list}}$. The difference score becomes $\Delta S_{\mathrm{list}}{|}_{N_{\mathrm{list}}=N}=\left(2{〈S^{+}〉}_{\mathrm{list}}\cdot N_{\mathrm{set}}-{〈S〉}_{\mathrm{list}}\cdot N\right)$ where ${〈S^{+}〉}_{\mathrm{list}}=S_{\mathrm{list}}^{+}/N_{\mathrm{set}}$ is the mean expression score averaged over all members of the gene set. Insertion into Eq. (10) for the special case λ = 1 provides the GSZ-score of the full list

(15) $$GSZ{|}_{N_{\mathrm{list}}=N}=\frac{{〈S^{+}〉}_{\mathrm{list}}-{〈S〉}_{\mathrm{list}}}{\sqrt{\mathrm{var}{\left(S\right)}_{\mathrm{list}}/N_{\mathrm{set}}}}$$

It represents a Z-statistics estimating the overexpression in terms of the deviation of the set average of the expression score from its total average over the whole gene list where the standard error is estimated using the variance of S for sample size N_set_. The respective shrinkage statistics is obtained with the substitution $\mathrm{var}\left(S\right)\to \mathrm{var}\left(S\right)\cdot \left(\lambda +\left(1-\lambda \right)\cdot N_{\mathrm{set}}^{\min}\right)\approx \mathrm{var}\left(S\right)\cdot N_{\mathrm{set}}^{\min}$ in the denominator of Eq. (15).

The second special case assumes an identical value of the expression score for all genes, S_g_ = 1, after ranking. The difference score thus simply counts the difference of members and non-members of the set in the list, $\Delta S_{\mathrm{list}}{|}_{S=1}=N_{+}-N_{-}=2N_{+}-N_{\mathrm{list}}$. The expected mean value and the variance in Eqs. (11) and (13) are given by < S > _list_ = 1 and var(S)_list_ = 0, respectively. Insertion into Eq. (10) provides the GSZ-score with λ = 1

(16) $$GSZ{|}_{S=1}=\frac{\left(N_{+}-{〈N_{+}〉}_{\mathrm{HG}}\right)}{\sqrt{\mathrm{var}\left(N_{+}\right)}}\approx \frac{\left(N_{+}-{〈N_{+}〉}_{\mathrm{HG}}\right)}{\sqrt{\frac{N_{\mathrm{set}}\cdot N_{\mathrm{list}}}{N}\cdot \left(\left(1-\frac{N_{\mathrm{list}}}{N}\right)\cdot \left(1-\frac{N_{\mathrm{set}}}{N}\right)\right)}}$$

where the right hand approximation assumes $N,N_{\mathrm{list}}>>1$. It represents a Z-statistics estimating the overrepresentation in terms of the deviation of the actual number of positive members from the expected mean according to the hypergeometric distribution and the respective variance. Eq. (16) further simplifies for short lists and sets, N_list_,N_set_ < <N, into:

(17) $$GSZ{|}_{S=1}\approx \frac{\left(N_{\mathrm{list}}^{+}-{〈N_{+}〉}_{\mathrm{HG}}\right)}{\sqrt{\frac{N_{\mathrm{set}}\cdot N_{\mathrm{list}}}{N}}}$$

The denominator substitutes for the shrinkage statistics with $\frac{N_{\mathrm{set}}\cdot N_{\mathrm{list}}}{N}\to \frac{\max\left(N_{\mathrm{list}}\cdot N_{\mathrm{set}},N_{\mathrm{list}}^{\min}\cdot N_{\mathrm{set}}^{\min}\right)}{N}$.

Eqs. (15) and (16) thus illustrate that the GSZ-score in its general formulation in Eq. (10) estimates enrichment in terms of a combination of overexpression and overrepresentation Z-scores. It has been shown in ref. [10] that the GSZ-score is related to alternative scores, namely the Random Sets [61] and the max-mean gene set statistics [62] representing a unification between these relevant scoring functions. Another comparative study on different gene set enrichment methods showed that removing incoherent pathways prior to analysis improves specificity [47]. The GSZ-score implicitly accounts for coherency because inconsistent genes with positive and negative contributions to the sum in Eq. (9) virtually compensate each other.

### SOM-based metagene and spot enrichment

SOM analysis provides two-dimensional contour maps visualizing the expression pattern of k = 1…K metagenes in a series of m = 1…M tissues. Each tile of the SOM refers to a minicluster of n_k_ genes associated with the respective metagene. The overrepresentation and/or overexpression of a gene set can be estimated for these metagene-related lists of genes using the methods presented in the previous subsection. Importantly, the list of length N_list_ = n_k_ per tile is invariant in all SOMs independently of the chosen tissue sample. In consequence, overrepresentation analysis in terms of the hypergeometric distribution (Eq. (8)) provides p-values for each gene-set s and metagene, p_s,k_, which apply to all particular SOMs of the series of tissues studied. In other words, metagene-related overrepresentation is independent of the particular sample considered. We estimated overrepresentation of the whole collection of 1454 gene sets in terms of a ranked list of p-values to identify the most relevant gene sets for each metagene.

One can also pursue an orthogonal approach which calculates the significance of one selected gene set in all metagenes to identify those of them which contain an enriched population of the genes from the chosen set. The results are visualized in terms of the so-called overrepresentation map. It color-codes the p-values of a particular gene-set in the two-dimensional mosaic of the SOM. The overrepresentation map also allows to link overrepresentation of a particular gene set with overexpression of the respective metagene by comparison with the sample-specific SOM. Particularly, overrepresented and overexpressed genes can be simply identified if overrepresentation and overexpression spots overlap in both maps. Note that the metagenes are located at the same positions in both maps.

In contrast to these sample-independent overrepresentation maps based on the hypergeometric distribution one can use the GSZ-score (Eq. (10)) to study metagene-related gene set enrichment in a sample-specific fashion. Also in this case we calculated p-values for all 1454 gene sets as default. The null distribution of the GSZ-score was calculated for each list using randomly composed gene sets of equal length.

Gene set overrepresentation and enrichment analysis was also applied to gene lists which are extracted from spots of adjacent metagenes. In this case, the respective length of the list is given by the sum of the number of real genes belonging to all metagenes forming the spot, $N_{\mathrm{list}}=\sum_{k\in spot}n_{k}$. Spot-related overrepresentation analysis based on the HG-distribution is characterized by one p-value per gene set and spot. It is independent of the selected sample if the spot is invariant in all samples. We applied this approach by using the global spots taken from the overexpression summary map which apply to all samples of the series. In addition, sample-specific spots are determined using a common overexpression threshold criterion to the SOM of different tissues. In this case one gets sample-specific overrepresentation lists because the size and position of each spot can vary from sample to sample and it can even disappear if the expression of the metagene strongly drops in a particular tissue. The GSZ-score delivers sample specific lists of gene sets for global and local spots as well because it explicitly processed the expression values of the genes in each spot.

Complete sets of results for full tissue dataset as well as zooming-in analysis can be found on our website: http://som.izbi.uni-leipzig.de.

## Competing interests

The authors declare that they have no competing interests.

## Authors’ contributions

HW and HB: conceived and designed this study, performed data analysis and wrote the manuscript. HW: wrote the R-programs and performed the calculations. All authors read and approved the final manuscript.

## Supplementary Material

Additional file 1 **The supplementary text addresses different details of our study: the error characteristics in different tissues, the amount of non-informative genes, gene rankings of single genes, spot patterns of randomized expression data, gene set overrepresentation and alternative spot selection, GSZ-enrichment of selected spots in selected tissues and the selection of gene sets using global lists and gene set.** Furthermore, the results of gene set analysis of subsets of tissues (zoom-in) and different summary reports are provided (Additional file 6). Click here for file

Additional file 2 Atlas of the ranking maps of all tissues studied. Click here for file

Additional file 3 Atlas of errors and p-value distributions of all tissues studied. Click here for file

Additional file 6 Results of gene set averaging approach. Click here for file

Additional file 4 Tissue specific gene sets. Click here for file

Additional file 5 Special gene sets of highly and weakly expressed and of housekeeping genes. Click here for file
