# Supplementary material for: Mining SOM expression portraits: feature selection and integrating concepts of molecular function
Source: BioData Min. 2012 Oct 8;5:18. doi: 10.1186/1756-0381-5-18 (PMC3599960; doi:10.1186/1756-0381-5-18)
Supplement: Additional file 1 — The supplementary text addresses different details of our study: the error characteristics in different tissues, the amount of non-informative genes, gene rankings of single genes, spot patterns of randomized expression data, gene set overrepresentation and alternative spot selection, GSZ-enrichment of selected spots in selected tissues and the selection of gene sets using global lists and gene set. Furthermore, the results of gene set analysis of subsets of tissues (zoom-in) and different summary reports are provided (Additional file 6). [file 1756-0381-5-18-S1.pdf]

Supplementary Text

Mining SOM expression portraits: Feature selection and integrating concepts of molecular function

Henry Wirth[1,2,3]*, Martin von Bergen[2,4], Hans Binder[1,3]*

[1] Interdisciplinary Centre for Bioinformatics of Leipzig University, D-4107 Leipzig, Härtelstr. 16-18
[2] Helmholtz Centre for Environmental Research, Department of Proteomics, D-04318 Leipzig, Permoserstr. 15, Germany
[3] Leipzig Interdisciplinary Research Cluster of Genetic Factors, Clinical Phenotypes and Environment (LIFE); Universität Leipzig, D-4103 Leipzig, Philipp-Rosenthalstr. 27, Germany
[4] Helmholtz Centre for Environmental Research, Department of Metabolomics, D-04318 Leipzig, Permoserstr. 15, Germany

* to whom correspondence should be addressed

1. Error characteristics of different tissues 2
2. Locally pooled error functions of single tissues 3
3. Non-informative and absent-called genes 5
4. Single gene ranking characteristics 7
5. Randomized SOM 9
6. HG- and GSZ-enrichment of selected spots in selected tissues 11
7. Functional context of over- and underexpression spots 14
8. Functional context of alternative spots 15
9. Selecting gene sets from global lists 17
10. Genes with functional annotations in selected spots 19
11. Zoom-in: Nervous tissues 20
12. Zoom-in: Immune systems tissues 23
13. Zoom-in: Diverse tissues 26
14. Selecting special sets of genes using ranking and expression criteria 29
15. Reports 31
16. References 36

1. Error characteristics of different tissues

We calculated the standard deviation of the expression of each probed gene for each tissue using the replicate samples available. The level of variability markedly decreases as a function of the expression value (Figure S 1, dot plots). The locally pooled error function (LPE, see green curves in Figure S 1) was combined with the gene-specific error into the regularized t-shrinkage values which provide specific p-value distributions for each tissue (Figure S 1, bar plots). These distributions are used to estimate the false discovery rate (local and tail-based ones, fdr and FDR, respectively) and the fraction of differentially expressed genes, %DE. Details of the method are given in the Methods section of the main paper.

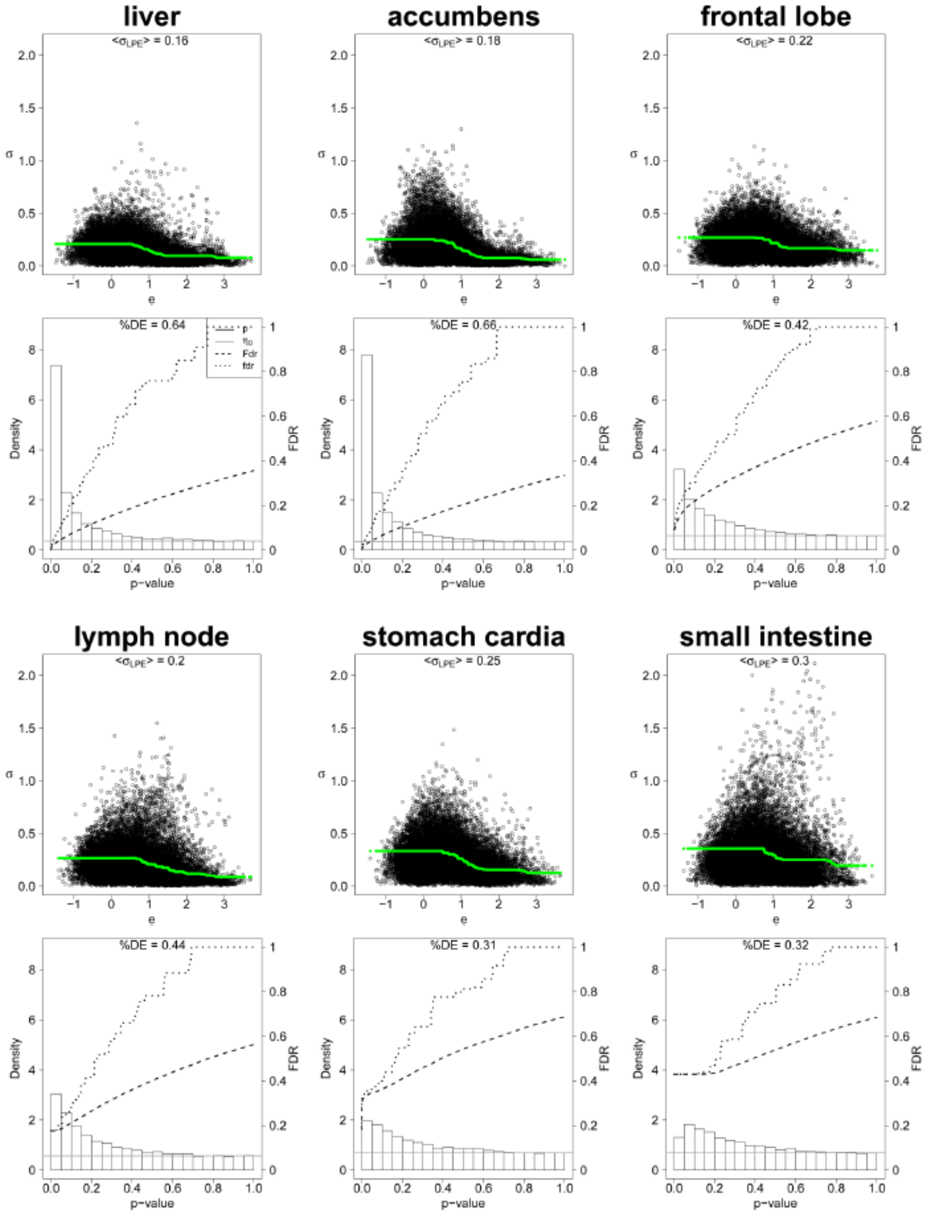

Figure S 1: Error characteristics of selected tissues: The first and third row of figures show error distributions (dots) and locally pooled estimates (green curves) of selected tissues as a function of the logged expression, e. The LPE-curves are calculated as moving average over 500 single probe values under the condition of non-positive slope which ensures that the LPE is maximal at small expression values. The second and fourth rows of figures show the respective p-value density distributions (bar histograms) together with the local FDR (dotted curves) and tail area-based FDR (dashed curves) obtained from the shrinkage t-statistics. The density-levels of null-genes, η_0, are shown by horizontal thin lines. The examples shown are ordered with increasing fraction of differentially expressed genes %DE.

2. Locally pooled error functions of single tissues

The locally pooled error (LPE) approach pools genes with similar expression values to estimate their variance with improved precision. It is justified by the observation that the experimental error of microarray expression values is governed by systematic factors caused by the physico-chemical principle of probe intensity detection. Particularly, the technical error of the measurement is a function of the expression degree [1–5] which can be derived using error propagation of the underlying hybridization isotherm [1], [6]. It predicts that the uncertainty of determining expression estimates inflates towards small expression levels due to the increasing contribution of the non-specific background and it progressively decreases towards large expression degrees due to saturation effects.

Indeed, the locally pooled error of the array data studied typically decreases with increasing expression degree (see Figure S 1 and Additional file 2 which shows the error functions of all tissues). The significance level of strongly expressed genes is consequently larger than that of weakly expressed genes for identical fold changes. In addition, the mean error level averaged over all probes can markedly vary between the different samples. We proved two summary measures to quantify the mean error level of each sample:

a) the mean standard deviation averaged over all probes,

$$\langle\sigma\rangle = \sqrt{\left\langle\left(\sigma_{g,m}\right)^2\right\rangle_{\text{all probes}}} \quad \text{with} \quad \left\langle\left(\sigma_{g,m}\right)^2\right\rangle \equiv \frac{1}{N \cdot M}\sum_{\text{all } g,m}\left(\sigma_{g,m}\right)^2 \approx \int_{e_{min}}^{e_{max}} P(e)\cdot\left(\sigma_{LPE}(e)\right)^2 \cdot de/\left(e_{max}-e_{min}\right),$$

and

b) the mean LPE-error,

$$\langle\sigma_{LPE}\rangle = \sqrt{\left\langle\left(\sigma_{LPE}(e)\right)^2\right\rangle} \quad \text{with} \quad \left\langle\left(\sigma_{LPE}(e)\right)^2\right\rangle \equiv \int_{e_{min}}^{e_{max}} \left(\sigma_{LPE}(e)\right)^2 \cdot de/\left(e_{max}-e_{min}\right),$$

where e_{max} and e_{min} are appropriate integration limits of maximum and minimum expression values. Note that also $<\sigma>$ can be obtained by integrating over the LPE-error function to a good approximation (see the right part of the equation above), where P(e) is the normalized probability density to find a probe with expression e. Accordingly, the mean LPE-error equally weights the error function whereas the mean standard deviation in addition considers the population of the expression values. In consequence, the value of $<\sigma>$ is closer to the standard deviation of strongly populated background probes whereas $<\sigma_{LPE}>$ better reflects the error of specifically hybridized, more strongly expressed but less populated probes.

Both error measures strongly correlate with r=0.99 (Figure S 2, panel a). In this study we use $<\sigma_{LPE}>$ as a characteristic measure of the mean level of scattering of the expression values between replicated samples. Group-averaging over the tissue categories (e.g., nervous tissues, immune systems tissues, muscle tissues etc.) reveals significant differences of their mean error level (Figure S 2, panel b). For example, adipose tissues and tissues related to digestion show nearly twice as large gene-related error levels than tissues of sexual reproduction, of exocrine function and partly of homeostasis.

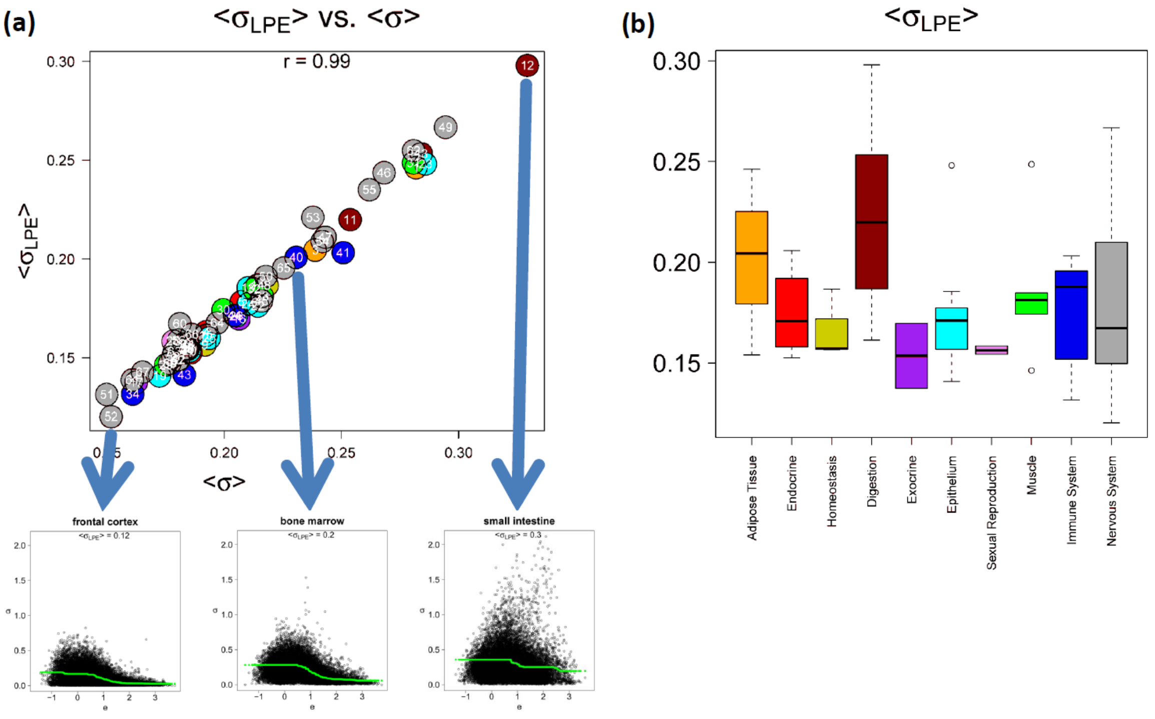

Figure S 2: Tissue specific error levels of microarray expression data: Panel a) Comparison of <σ> and $<\sigma_{LPE}>$ for all tissues studied reveals strong correlation with r=0.99. LPE error functions are shown for selected tissues referring to low, intermediate and high error levels: 'frontal cortex', 'bone marrow' and 'small intestine'. Panel b) Boxplot of $<\sigma_{LPE}>$ for different tissue categories defined in [7]. Note the different error levels. The color code is used also in the scatter plot (panel a). The error plots and $<\sigma_{LPE}>$ values of all tissues are given in Additional file 2.

3. Non-informative and absent-called genes

The total fraction of differentially expressed and thus informative genes per sample can be estimated using the background level of the respective p-value distribution, $\%DE = 1 - \eta_0$. %DE decreases with increasing mean error level $<\sigma_{LPE}>$ and with increasing FDR at a selected p-value (p=const). In analogy to %DE we define %fdr (and %FDR) as the fraction of genes, the FDR-value of which falls below a given threshold, e.g. $fdr(p)<fdr_{threshold}$ for the local FDR-value. We arbitrarily chose $fdr_{threshold}=0.5 > Fdr_{threshold}=0.2$ where the latter relation ensures similar values of %fdr and %Fdr (see previous subsection). Both, %fdr and %Fdr, strongly correlate with each other (r=0.97; data not shown) and with %DE (see the %fdr-vs-%DE plot in Figure S 3 r=0.98). The latter result indicates that %fdr is largely determined by the noise floor of non-informative probes whereas the slope of the decay of the p-value distribution near its left boundary has, if at all, an almost tiny effect. Note that both factors, the non-informative noise floor and the particular shape of the distribution of informative probes, can affect %fdr.

%DE (and %fdr) negatively correlates with the mean error level $<\sigma_{LPE}>$ (r= -0.79, Figure S 3), i.e., a higher uncertainty of the expression measures is accompanied by a smaller number of differentially expressed genes on the average. This result reflects the fact that a larger uncertainty of the expression estimates effectively increases the fraction of non-informative probes which contribute to the null distribution only. Note that %DE more than halves from values about 0.7 to 0.3 if $<\sigma_{LPE}>$ increases from ~0.1 to 0.3.

On the other hand, $<\sigma_{LPE}>$ is related to absolute expression values whereas %DE refers to differential expression relative to a reference level. %DE of a particular tissue is consequently affected by its expression profile and by the respective noise floor. The expression level is governed by biological factors, e.g. by the tissue specifics of gene activity, whereas the noise level mainly depends on the precision of the measurement, which is affected by biological and methodical effects as well. Hence, the obtained correlation between %DE and $<\sigma_{LPE}>$ indicates that the precision of the expression measurement largely affects the number of detected differentially expressed features.

To further analyze the noise-level inherent in the data we included the fraction of absent-called genes (%N) in our correlation plot where %N is defined as the fraction of genes the expression of which falls below the detection threshold of the microarray measurement. It is determined separately for each chip in the calibration step [7–9]. Interestingly, %N does virtually not correlate with %DE (r=-0.04), however it moderately correlates with $<\sigma_{LPE}>$ (r=0.38), which, in turn, correlates with %DE (r=-0.79, see previous paragraph). The quantile normalization and scaling algorithms used transform the individual sample-specific density distributions of expression values into one common average distribution [7]. As a result the potential relation between %N and %DE gets mostly lost in this step presumably because also absent-called genes can differentially express in different samples. In consequence, %N essentially does not affect the differential expression estimates whereas it is directly related to the mean error level of each array. In turn, $<\sigma_{LPE}>$ affects %DE because it determines the significance of the differential expression values and thus %DE.

This somewhat puzzling relation between the error measures considered shows that data transformation after preprocessing and normalization can mask mutual relations. Most importantly, the number of differently expressed genes meeting a given significance criterion is governed by the error level of the expression measures which, in turn, systematically varies between the different tissues and tissue types.

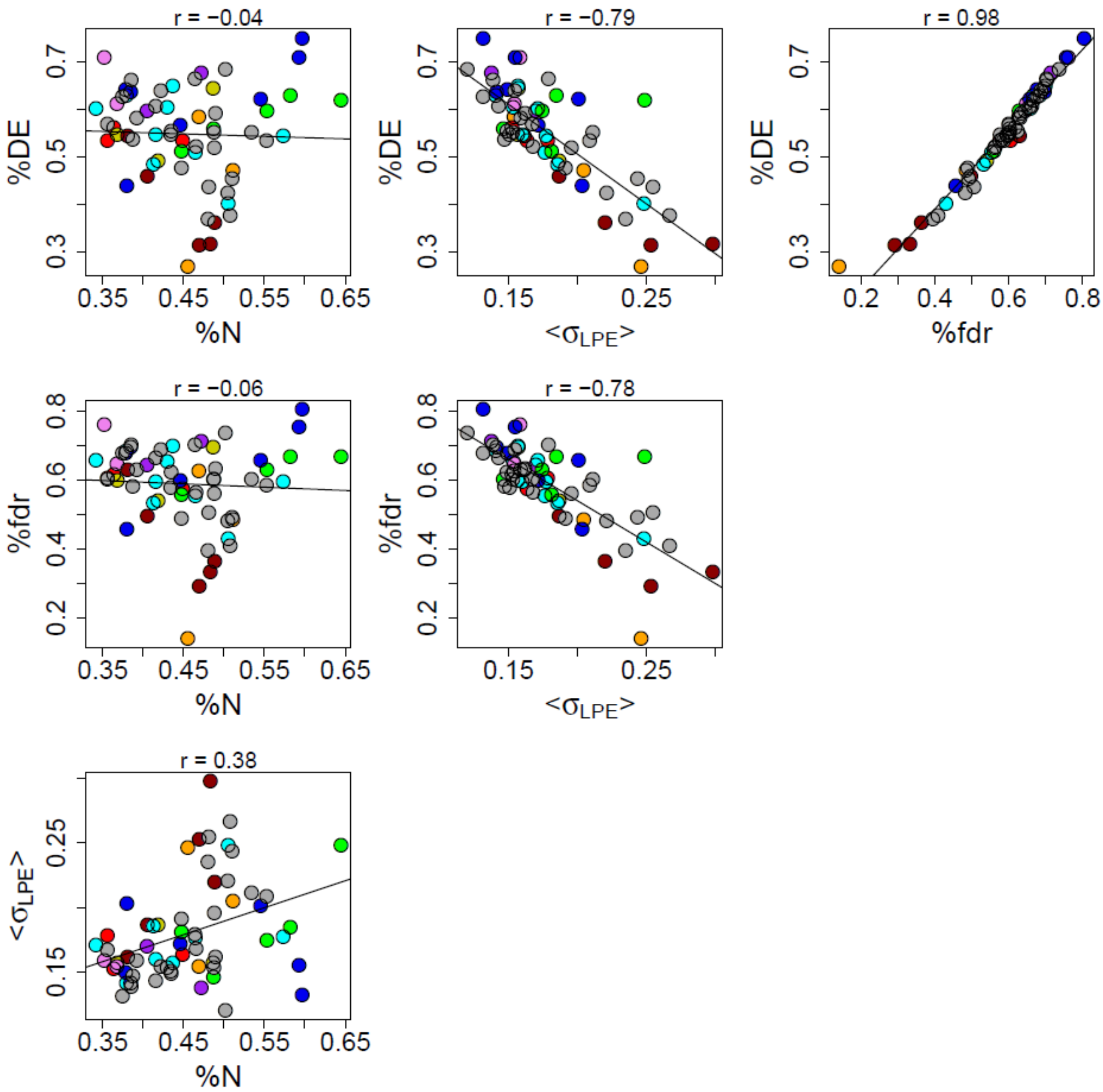

Figure S 3: Correlation plots of different error estimates, the fraction of differentially expressed genes (%DE), of genes meeting a minimum FDR-criterion (%fdr), absent-called genes (%N) and the mean error level (<σ_{LPE}>), of the tissues studied. Regresssion lines and the respective regression coefficients are given within the figure.

4. Single gene ranking characteristics

We calculated mean global and local CAT(r) and Δp-CAT(r) values for lists of length r=10 and 100 of all tissue samples studied considering either all genes or the genes taken from the strongest overexpression spot, respectively. Figure S 4 shows boxplots of the data. The results of these global and local rank comparisons at both rank positions consistently show that similarities between the different lists are maximum for FC/WAD, and worse but virtually similar for WAD/t-shrinkage and FC/t-shrinkage pairings. Lists agree to about 70% (FC/WAD) and 50% (WAD/t-shrinkage and FC/t-shrinkage) on both, the global and local level and for both considered lengths (r=10 and 100) on the average. Local lists are slightly more similar by a few percent than global ones due to the pre-filtering of the genes in the SOM-spots. The averaged Δp-CAT values show that the penalty of the WAD- and FC-lists in units of the cumulative p-value of the t-shrinkage statistics is very similar for the global lists at rank r=10 and 100 and for the local lists of the overexpression spots at r=10. The former results indicate that the global lists are virtually equivalent at r>10 for all scores applied. Interestingly, the penalty of the Δp-CAT score of the local list almost completely disappears at r=100. This result can be rationalized by the fact that genes which penalize the p-CAT score at r<10 are simply shift to ranks 10<r<100 in the alternative lists where they compensate the penalty on top of the list. This effect of compensation is not observed for the global lists. Overexpressed genes are obviously preselected within the respective overexpression spots by the SOM machine learning algorithm making the local rankings more stable in the considered r-range.

The spot-filtering effectively combines the scoring of differential expression with the selection of co-expressed and correlated genes. It has been previously shown that ‘correlation-sharing’ for the detection of differentially expressed genes improves the performance of the analysis in terms of the false discovery rate [10].

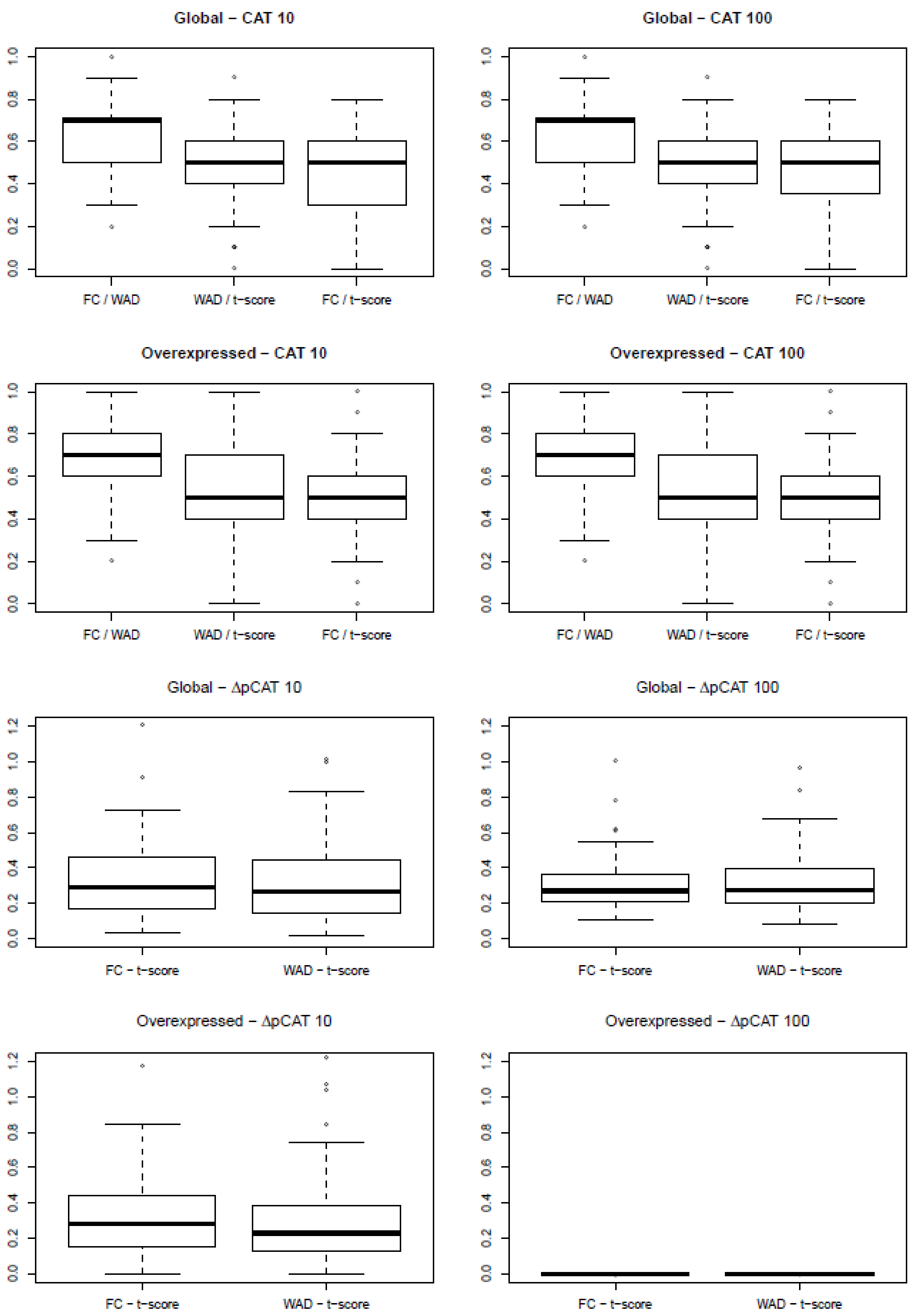

Figure S 4: Boxplots of rank-differences of ordered lists obtained from the different significance scores used at position r=10 (left) and r=100 (right) of all tissues studied: The differences are estimated using the CAT- and Δp-CAT scores for global and local lists considering all genes or genes of the strongest overexpression spot, respectively. The Δp-CAT(r) values are given as difference with respect to respective p-CAT(r) value of the t-shrinkage statistics.

5. Randomized SOM

The SOM images visualize the gene expression landscapes of different tissues in terms of spot patterns which can be understood as disjunct regulatory modes of gene expression. Each of them is represented by a cluster of co-regulated genes. Co-regulation is assumed to be caused by the involvement of the genes from a particular spot into common pathway activities according to the 'guilt-by-association'-principle. Alternatively, genes can be ostensibly co-regulated also by chance, for example, in an ensemble of genes with random expression profiles. The probability to find such random co-regulation patterns depends on the number of different conditions studied and on the resolution of the cluster algorithm used. We studied the effect of random expression for the tissue data set simply by permuting the expression values of each gene randomly among the samples. This way the tissue-specificity of each expression profile is completely destroyed. Then, a SOM was trained using the randomized data. We applied the same SOM size and grid-topology as for the unperturbed tissue SOM. Finally, the expression landscapes of both SOM's were compared in terms of the number and properties of spot clusters and metagene variance (Table S 1). In addition we trained SOM's of increasing size to the data and performed sample similarity analysis in terms of neighbor-joining trees (Figure S 5).
The number of overexpression spots and thus of correlation clusters increases nearly threefold after randomization (Table S 1, SOM size 30x30). Moreover, the observed number of spots in the randomized SOM monotonously increases with sample size whereas that of the tissue SOM levels off to limiting values always for small sample sizes (Figure S 5). Hence, the 'real' expression landscape of human tissues is considerably less fragmented than the respective random one. The more 'bush-like' similarity tree of the latter one reflects this fact and also the aspect that after randomization also the covariance structure between the different modules disappears. Hence, the random landscape is characterized by more and SOM-size dependent expression modules without mutual correlations. Their expression amplitude is however less variable than that that of the tissues. Hence, the randomized expression landscape is more flat than the real one. Note that our randomization algorithm does not change the variability of the expression profiles of the single genes. The decrease of the variability of the metagenes after randomization thus reflects a stronger 'smoothing' effect of the metagene profiles due to less concerted single gene profiles in each of the metagene miniclusters. This effect becomes also obvious in the larger covariance (cross correlation) and reduced mean value of the Euclidian distance between the single gene and metagene profiles in each of the miniclusters (Table S 1).

Table S 1: Comparison of the tissue SOM before and after randomization of the input data

	**Tissue SOM**	**…after randomization**
#of overexpression spots: mean / total [a]	1.4 / 10	3.3 / 50
# of correlation clusters [b]	121	311
median Gene per metagene G/M[c]	4±10; max=308	5±8; max=248
Variance [d]: var_k	0.01±0.05; max=0.57	0.001±0.002; max=0.008
Covariance [d]: r_k	0.61±0.14; max=0.94	0.36±0.06; max=0.88
Deviation [d]: d_k	0.15±0.10; max=0.59	0.20±0.09; max=0.53

[a] mean and total number of overexpression spots (>98% threshold)
[b] number of correlation clusters using the seed algorithm
[c] median number of genes per metagene± standard deviation and the maximum occupancy observed
[d] mean metagene variance, mean metagene-gene covariance and mean metagene-gene Euclidian distance (deviation) of all metagene profiles

Hence, the random SOM is characterized by a larger number of different expression states which are only partly captured by the particular SOM-size used. In consequence, the increase of the SOM-size gives rise to an increasing number of spots. In contrast, the number of expression modules of the tissue SOM asymptotically levels off.

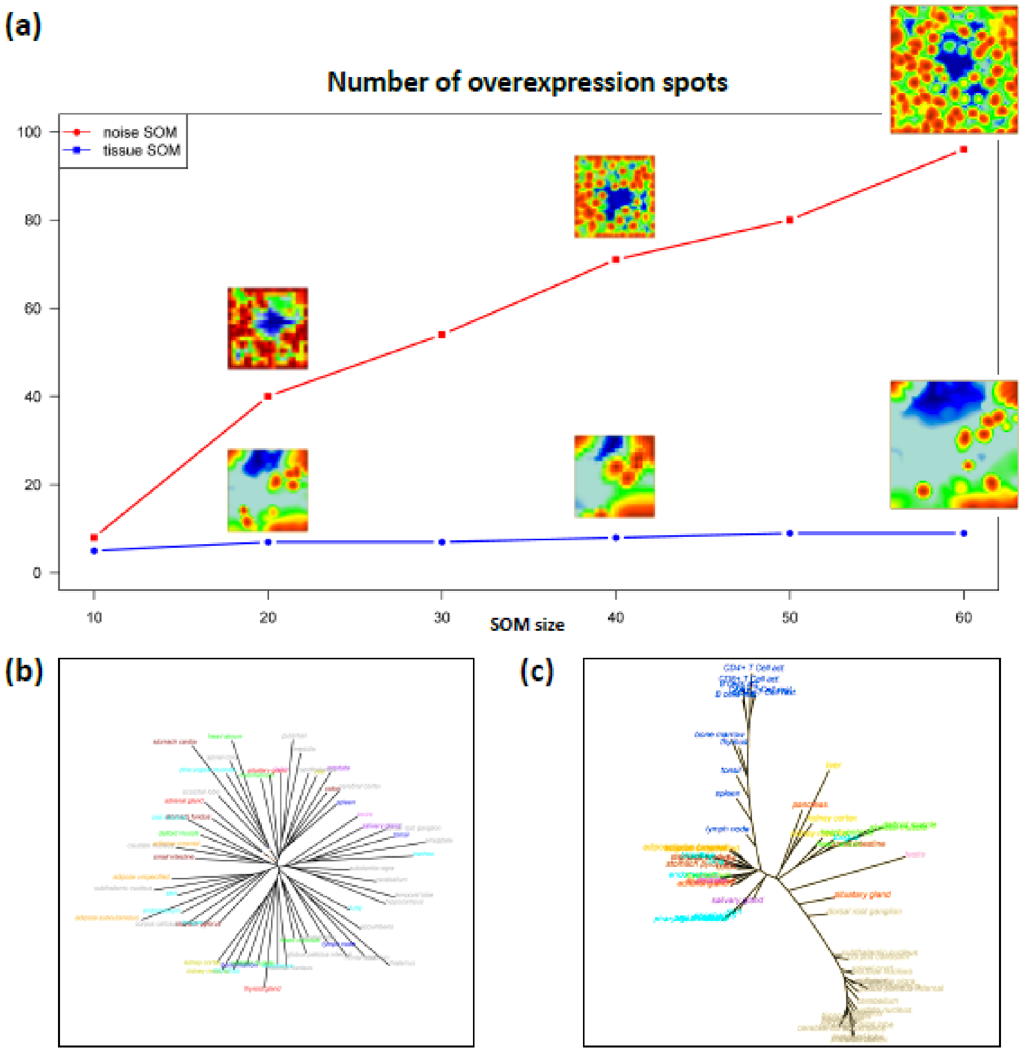

Figure S 5: Tissue-vs-randomized SOM: (a) Total number of overexpression spots as a function of the SOM size observed in the SOM portraits of human tissues before (blue curve) and after (red) randomization. The respective overexpression summary maps are shown for SOM sizes 20x20, 40x40 and 60x60. Sample similarity dendrograms for the set of human tissues after (b) and before (c) randomization. Tissues of the same category are colored identically (e.g., nervous tissues in grey, immune systems tissues in blue and muscle tissues in green).

6. HG- and GSZ-enrichment of selected spots in selected tissues

Figure S 6 shows bar plots of the top-twenty HG-overrepresented gene sets in the three spots A, B and F. Ten out of the top-twenty gene sets of spot A are related to nervous system and virtually all twenty gene sets overrepresented in spot B to muscle. Spot F overrepresents sets related to inflammation, leukocyte function etc. as expected for immune systems tissues. The annotation of the overrepresented gene sets clearly agrees with the tissues overexpressing the respective spot.

GSZ-enrichment analysis takes into account overrepresentation and overexpression of the genes of each set. It consequently provides sample-specific enrichment lists for constant spots due to the changing expression values of each gene in contrast to HG-overrepresentation which is sample independent. Figure S 7 shows bar plots of the top-ten GSZ-scored gene sets which are over- and underexpressed in the three spots A, B and F in three selected tissues (frontal lobe, skeletal muscle, lymph node).

The three arrows indicate the same three gene sets enriched in each of the spots for comparison. The GSZ-ranking provides very similar positions on top of the enrichment list in the tissues which overexpress a given set of genes in the respective spot (compare with Figure S 6): for example gene sets related to nervous processes are overexpressed in spot A of nervous tissue taken from the frontal lobe; gene sets related to muscle contraction are overexpressed in the muscle-related spot B of skeletal muscle tissue and gene sets related to immune system processes are overexpressed in the 'immune system spot' F of lymph node tissue. The expression of these spots can drop drastically in the other tissues considered. In consequence, part of the discussed gene sets occupy even leading position in the respective underexpression lists: for example, the gene sets addressing nervous processes (spot A) and muscle processes (spot B) are on leading positions in the underexpression list of lymph node tissue and the gene sets addressing immune system processes (spot F) and muscle processes (spot B) are found on top of the underexpression list in frontal lobe tissue. This result illustrates the property of the GSZ-score to combine gene set overrepresentation with over- (and under-) expression of the associated genes.

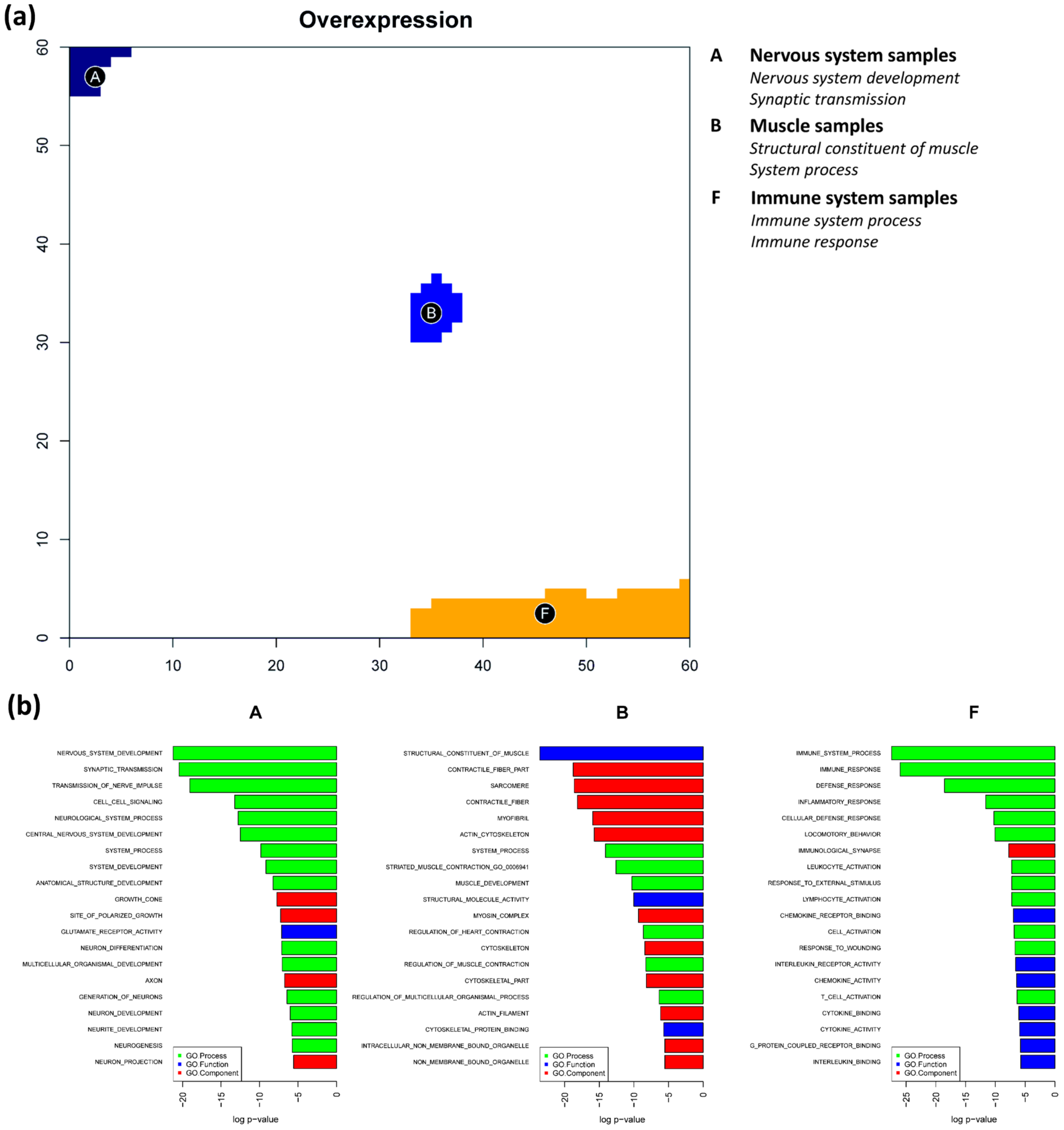

Figure S 6: The top-twenty gene sets of three selected spots. The length of the bars scales with the logged overrepresentation p-value of the sets. The color assigns the category of the gene sets according to the GO terms ‘molecular process’ (green), ‘molecular component’ (red) and ‘molecular process’ (blue).

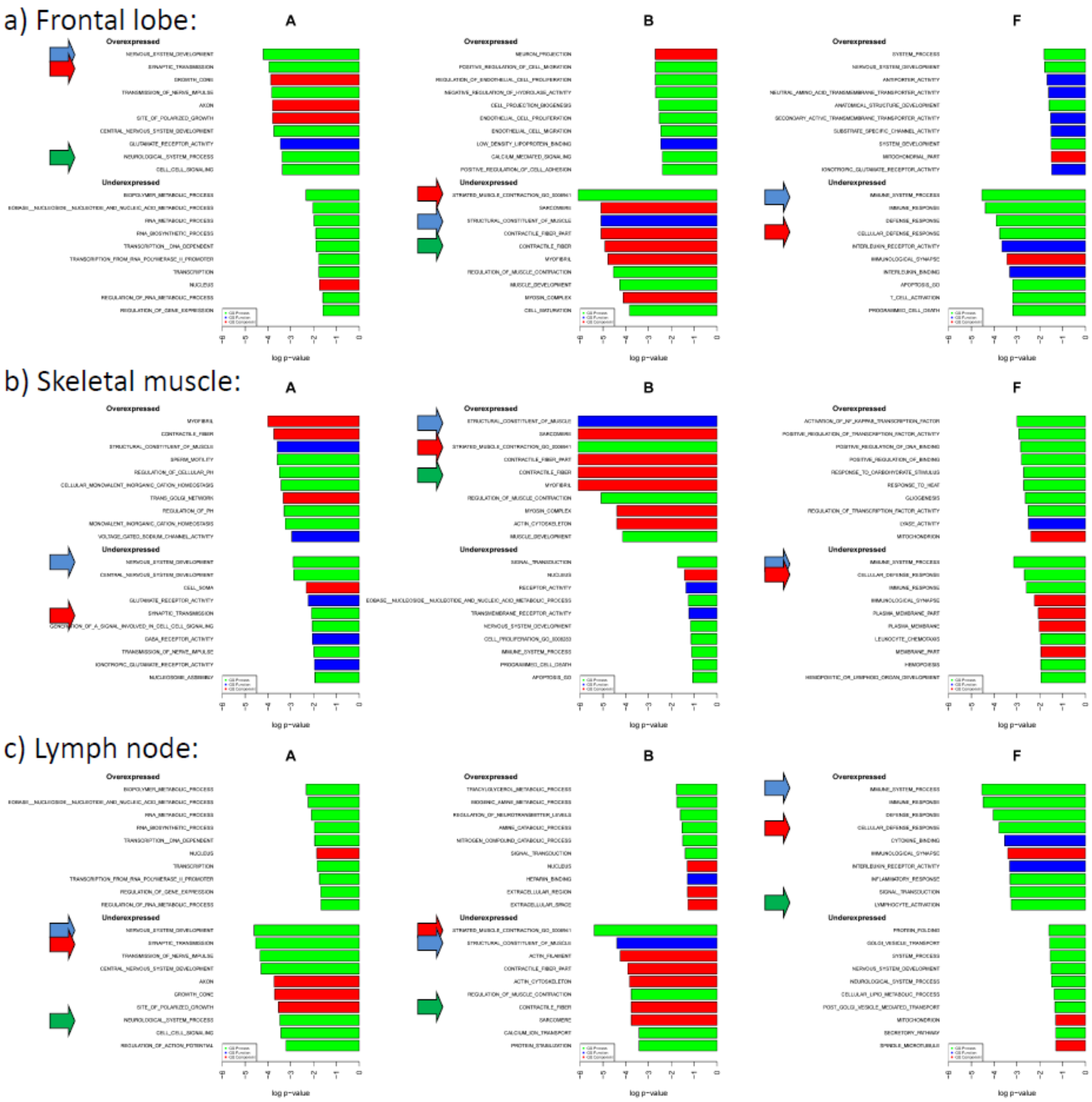

Figure S 7: Top-ten GSZ-over- and -underexpression lists of gene sets in the three spots for three tissues (the spot assignments are given in the main paper and in ref. [7]). The arrows indicate the same sets in each spot for direct comparison.

7. Functional context of over- and underexpression spots

The heatmaps in Figure S 8 show the mean expression of the overexpression and underexpression spots selected (see also the over- and underexpression maps in the right part of the figure for assignments of the spots). The three top overrepresented gene sets given in the figures allow assigning the functional context of each of the spots. Note that the terminus ‘over-/under-expression’ spot refers to the criterion of spot detection. Both types of spots show usually high expression in one and low expression in other tissues. A few of the over- and underexpression spots occupy the same (e.g. spots D and g) but mostly different positions in the maps. They consequently carry complementary information of high- and low-expression genes. For example, overexpression spot F can be assigned to ‘immune response’ whereas the nearby located underexpression spot b refers mainly to the translation machinery in the nucleus. The underexpression map detects also spots in regions without strongly overexpressed genes: For example, underexpression spot ‘a’ which can be assigned to endocytosis and membrane-related transport. Note also that the underexpression landscape is less sharp compared with the overexpression landscape as indicated by the contrast of the respective spot expression heatmaps shown in Figure S 8.

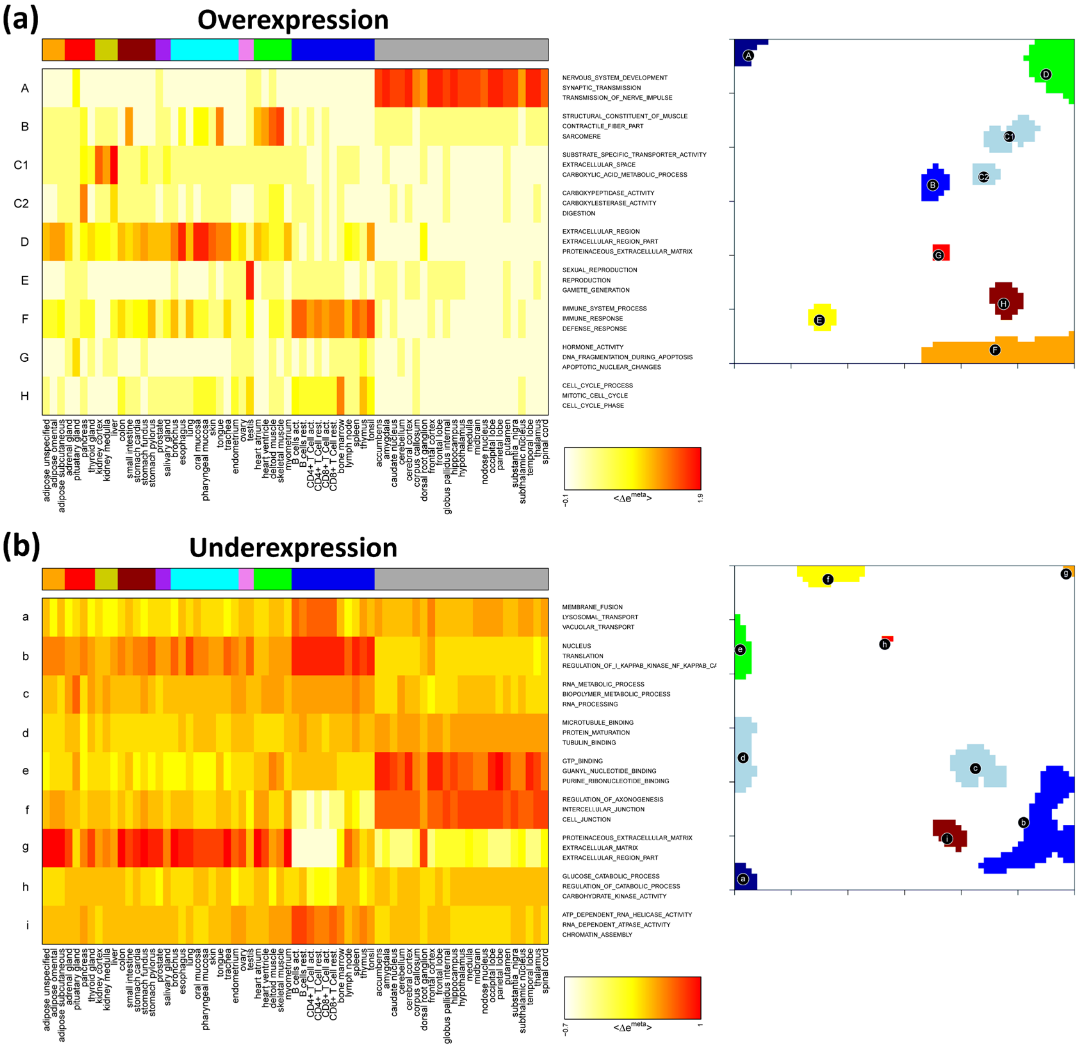

Figure S 8: Overrepresentation analysis of overexpression (panel a) and underexpression (panel b) spots. The heatmaps show the mean expression of the selected spots in all tissues studied. The top three overrepresented gene sets in each spot are given for each spot. The respective spot maps are redrawn from the main paper for direct assignment of the respective spot positions in the map.

8. Functional context of alternative spots

We applied alternative methods of spot selection partly described previously [7]. K-means clustering of the metagene profiles provides an area-filling spot pattern (Figure S 9). Here we arbitrarily set the number of clusters to fifteen to distribute the metagenes over a similar numbers of clusters as detected in the unsupervised spot selection based on the over- or underexpression of the metagegenes. Partly the position and size of the obtained spots agree with that of the overexpression and/or underexpression maps (e.g. the clustering spot C with overexpression spot A, and also H with F and J with f). Other spots occupy different areas of the map not selected by the over- or underexpression criteria (e.g. M, K). Moreover, most of the cluster-spots are larger than the typical over-/underexpression spots giving rise to a more coarse fragmentation of the map. On the other hand, the clustering spots enable the gapless sorting of genes into cluster-spots. Note that four of these cluster-spots are specifically overexpressed in nervous tissues (C, G, I, J, see Figure S 9b) with subtle differences in their functional context: Whereas spot C and G both overrepresent genes related to synaptic transmission, spots I and J collect genes associated with the pernuclear region and axiogenesis, respectively. The former ones are strongly underexpressed in most of immune system tissues.

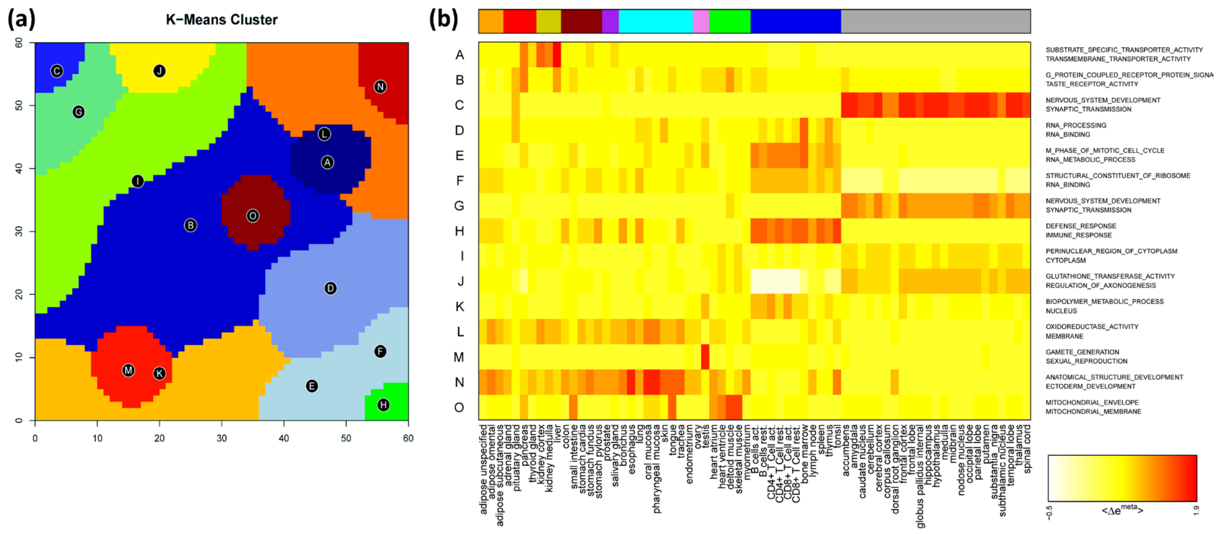

Figure S 9: Spot map based on k-means clustering of the metagene profiles (panel a). The heatmap shows the mean expression of the spots detected in all tissues studied. Spots are assigned using capital letters.

As an additonal option we selected spots of highly correlated metagenes (see ref. [7] for details of the spot selection algorithm). The obtained spot areas are again partly different compared with the spots obtained by the other methods discussed so far. The correlation spots tend to fragment the regions along the border of the map which refer to the metagenes of strongest variability of their expression profiles ([7]). The functional context reveals further details of SOM-mapping: For example, spots C, O, D, I, J and K are related to different aspects of nucleus function such as sexual reproduction (D), the ubiquitin ligase complex (O) and RNA metabolism (I).

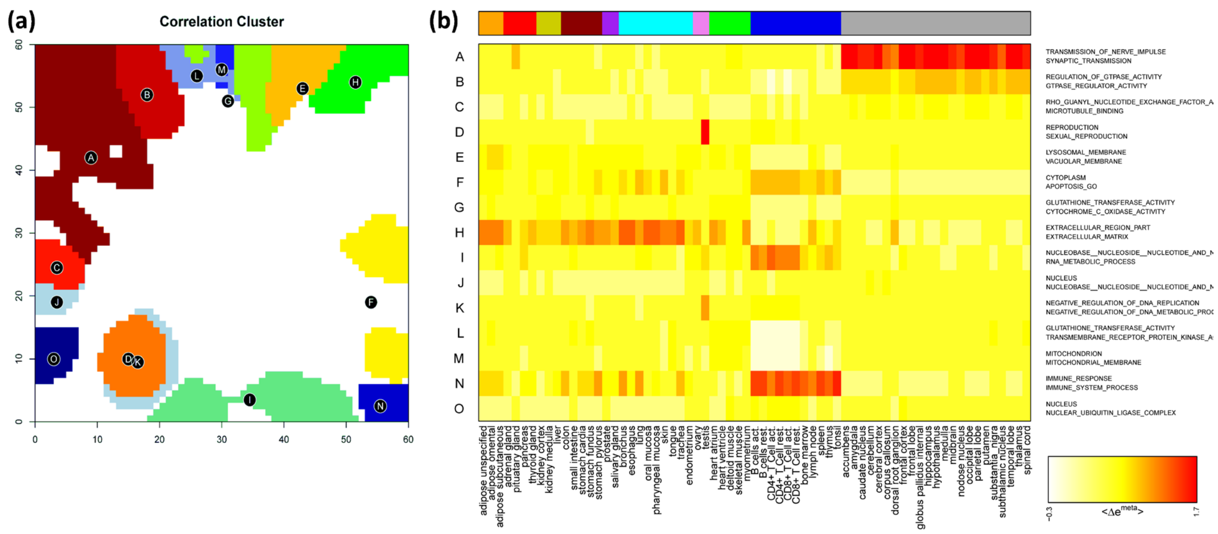

Figure S 10: Spot map based on Pearsons correlation coefficient between adjacent metagenes (panel a; see [7] for details). The heatmap shows the mean expression of the spots detected in all tissues studied. Spots are assigned using capital letters.

9. Selecting gene sets from global lists

Our basic algorithm applies the 'top-three' criterion to the local lists of gene sets and extracts the selected sets into one global list. Particularly, it selects the three top gene sets per spot and merges them into the global list of gene sets which is further used to characterize gene expression in the different tissues in a functional context. This approach equally weights each spot in terms of the number of selected gene sets. This way, it ensures that each spot-feature is equally represented in the resulting global list.

Alternatively one can merge the full local spot lists of gene sets (i.e. without selecting the top-three sets) into one global one, rank them with increasing p-value and finally cut the list either at a suited significance threshold or after a certain number of positions. In this case the spots contribute with different numbers of gene sets depending on the respective degree of enrichment. We applied this approach using the p-values of the hypergeometric distribution and of the GSZ-score which was calculated separately for over- and underexpression spots.

Figure S 11b shows the respective density distribution of the p-values (from the left to the right). These p-value distributions provide the total fraction of significantly enriched gene sets, %DE, and the respective local (fdr) and tail based (FDR) false discovery rates in analogy with the single gene analysis described in the methodical part of the main paper. The FDR- and fdr-functions increase much more steeply for the GSZ-lists compared with the HG-list. In consequence, application of a constant significance level (e.g. FDR<0.1) selects much less features from the GSZ-list than from the HG-list. Recall that the respective null distributions are given either analytically by the hypergeometric distribution or they are estimated empirically for the GSZ-distributions using random permutations. These different approaches presumably produce the different FDR-levels of both approaches. Note that the null distribution of a test statistic under permutation is not necessarily the same for equally and differentially expressed genes. Previously it was suggested to use suited subsets of the data to more accurately estimate true nulls and to substantially increase the power of significance testing [11], [12]. Moreover, methodical problems with the proper definition of the null hypothesis and the proper calculation of p-values which arise in the context of gene set enrichment analysis have been identified [13].

To compare the results of both approaches we select similar numbers of gene sets in each of the global lists referring to HG-enrichment (145 gene sets with fdr<0.0001), GSZ-overexpression (169 gene sets with fdr<0.1) and GSZ-underexpression (72 gene sets with fdr<0.2). The obtained gene set enrichment heatmaps in Figure S 11a reveal very similar spot pattern with essentially the same lists of enriched genes sets (Table S 2). Note that the underexpression GSZ-heatmap collects sets of virtually inactive genes whereas the overexpression heatmaps (HG and GSZ) refer to strongly overexpressed gene sets. The obtained sets refer consequently to different functions.

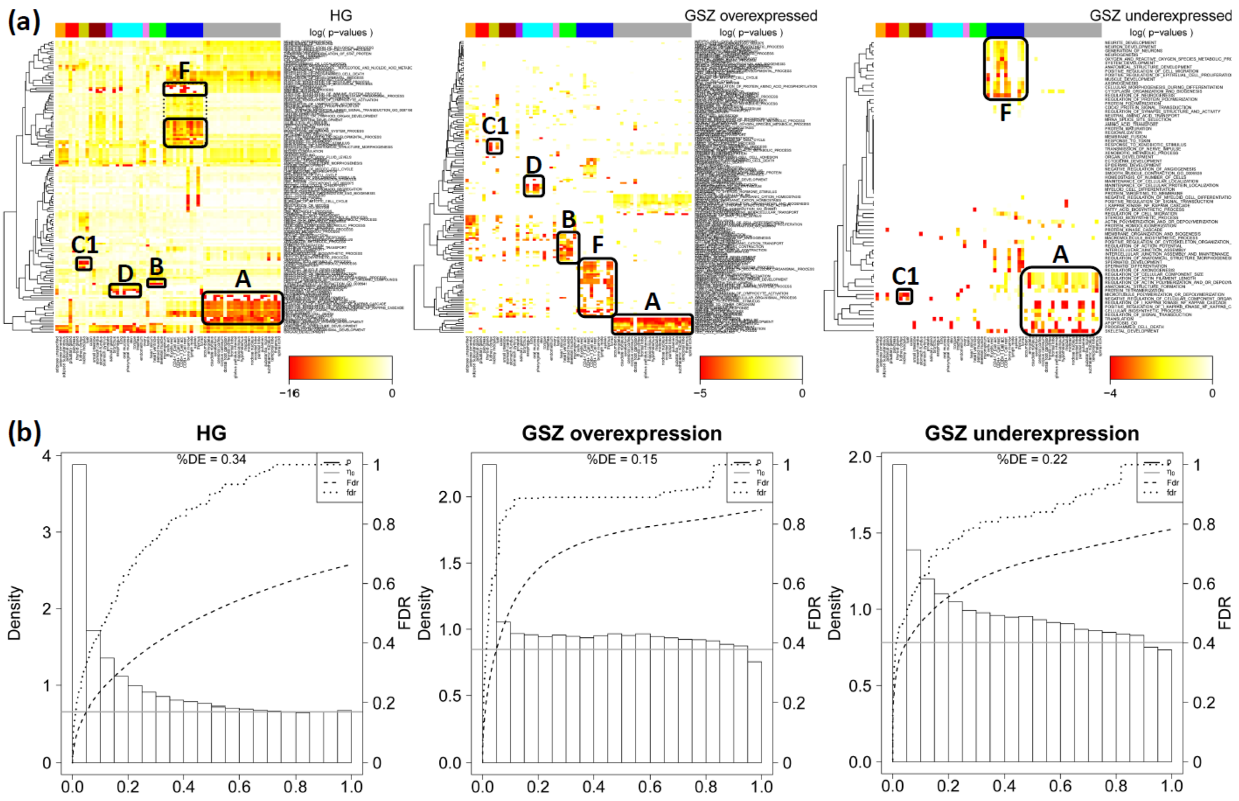

Figure S 11: One-way hierarchical clustering heatmap of significantly enriched gene sets (rows) versus tissues (columns) using the HG- and the GSZ-statistics using a fdr-threshold (panel a). The GSZ-statistics was applied separately to over- and underexpression spots. The heatmap color codes the p-values of the respective score in log-scale (see the legends in the figure). The tissue categories are color coded in the bar above the heatmap according to the assignments given in [7]. The gene sets are clustered in vertical direction. Panel b shows the respective p-value density distributions together with the local (dotted curves) and tail based (dashed curve) false discovery rates (see right ordinates).

Table S 2: Top gene sets from selected spots of the heatmaps shown in Figure S 11

spot	HG	GSZ-overexpression	GSZ-underexpression [a]
A	Cell-cell signaling	Cell-cell signaling	Skeletal development
	Neurological system process	Transmission of nerve impulse	Regulation of I-κB cascade
	Synaptic transmission	Synaptic transmission	Translation
	Nervous system development	Nervous system development	Apoptosis
F	Lymphocyte activation	Lymphocyte differentiation	Regulation of neurogenesis
	Regulation of immune system	Immune system development	Cytoplasm organization
	Immune response	Immune response	Axonogenesis
	Defense response	Defense response	Neuron development
C1	Organic acid metabolic process	Organic acid metabolic process	Microtubule polymerization
	Carboxylic acid metabolic process	Carboxylic acid metabolic process	Negative regulation of cellular component organization
	Excretion	Glutamine family metabolic process	

[a] assignment of underexpression spots to capital letter is arbitrary

10. Genes with functional annotations in selected spots

We count the percentage of genes in selected spots which can be assigned to any of the gene sets considered. ‘Primary’ gene sets are the top-enriched ones whereas ‘other’ gene sets are not necessarily enriched in the particular spots. The data show that typically only about 20% (or less) of the genes are members of the top enriched sets and that more than 30% are of unknown origin with respect to gene set membership.

Table S 3: Amount of genes assigned to functional gene sets in selected spots

**Spot** [a]	**Total** [b]<br># of genes	**Primary gene sets** [b]<br>% in enriched sets	**Other gene sets** [b]<br>% in other sets	**Not in gene sets** [b]<br>% not assigned
**A**<br>nervous system	445	22%	40%	38%
**B**<br>muscle	229	22%	49%	29%
**F**<br>immune system	1558	13%	52%	35%.

[a] Spots are assigned in correspondence with Figure 6a in the main paper

[b] total number of genes in the respective spots which decompose into genes with membership in the top-three HG-enriched gene sets, genes with membership in at least one of the remaining gene sets tested and genes without membership in any of the gene sets

11. Zoom-in: Nervous tissues

We applied a ‚zoom-in' step of SOM analysis to study the expression profiles of the subgroup of nervous immune systems and the remaining 'diverse' tissues with enlarged resolution as described in ref. [7]. They show 'new' textures of characteristic over- and underexpression spots which reflect the expression profiles of the tissues of interest more in detail than the original SOM. Figure S 12a shows the obtained overexpression spots and the three leading overexpressed gene sets after global overexpression analysis of nervous tissues. Spot H collects processes directly related to nervous system whereas spots G and H refer to nucleus-related and cell membrane-related processes, respectively. The zoom-in map amplifies subtle details of the expression profile of these genes in the reduced subset selected for zoom-in analysis. Also that the GSZ-overexpression profile of the gene set 'nervous system development' shows a heterogeneous fine structure which reflects modulation of the expression of this set in the nervous tissues. The GSZ-enrichment heatmap after zoom-in is shown in Figure S 12b. It provides a detailed picture of the gene set enrichment in nervous tissues.

Figure S 13 and Figure S 14 provide overrepresentation maps and overexpression profiles of the same gene sets selected in the respective plots in the main paper. The genes of the sets widely distribute over the maps. The gene sets related to synaptic transmission and to the transmission of nerve impulse indeed accumulate in the region of spot H and the gene set 'immune systems process' in the region of spot H, as expected. The overexpression level however can strongly vary in the different nervous systems tissues: For example, the former two gene sets are clearly underexpressed in corpus callosum and subthalamic nucleus, which, on the other hand, show relative overexpression of the gene set immune systems process. The overexpression of the remaining three gene sets considered is mostly invariant in nervous tissues.

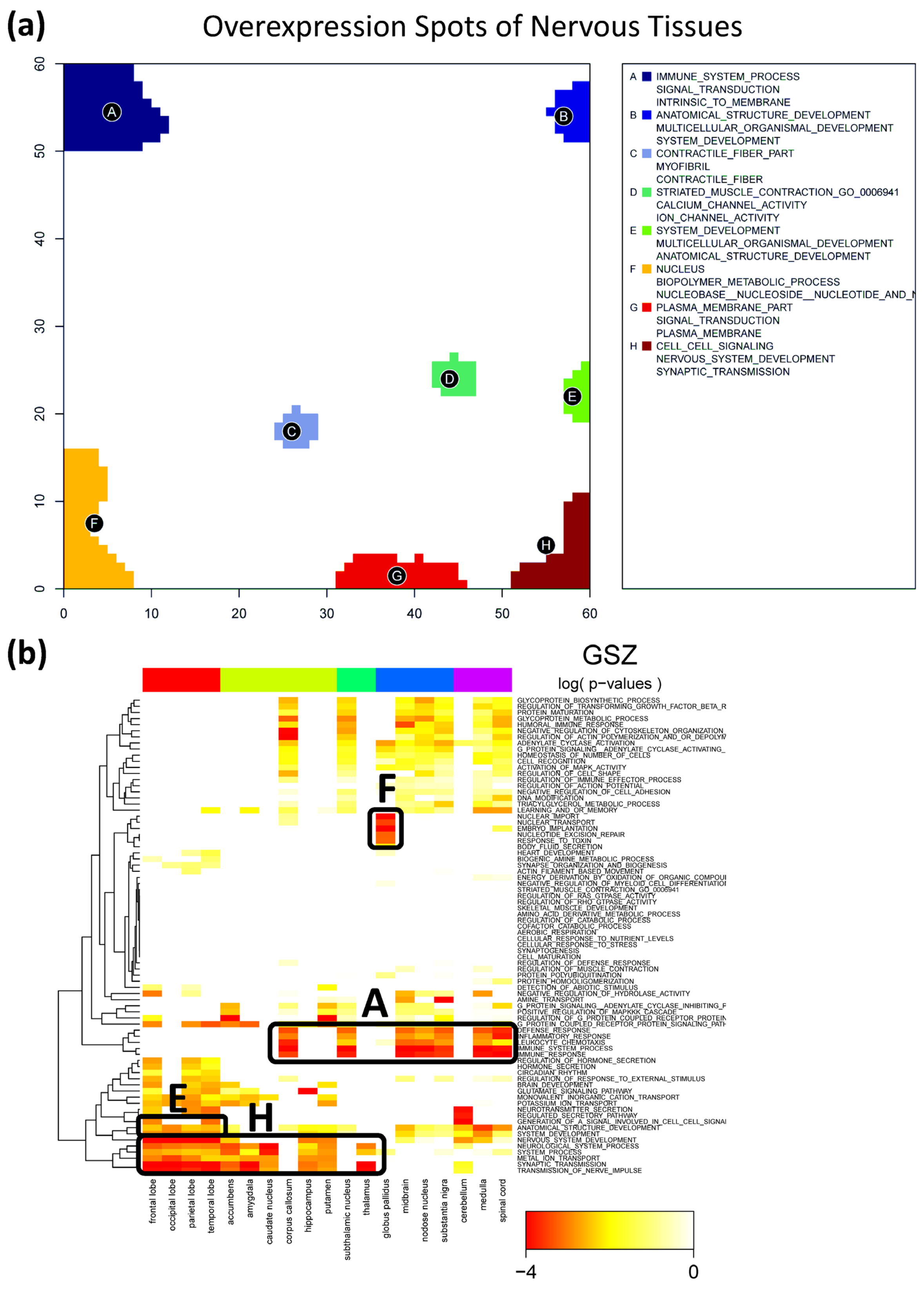

Figure S 12: The overexpression summary map of nervous tissues shows eight spots (A – H) which are strongly overexpressed in at least one of the 19 nervous tissues studied. Global overrepresentation analysis is estimated for each spot using the hypergeometric distribution. The right legend assigns the two most significantly overrepresented gene sets in each spot. Expression heatmaps of the spots are shown in the supplementary material of ref. [7].

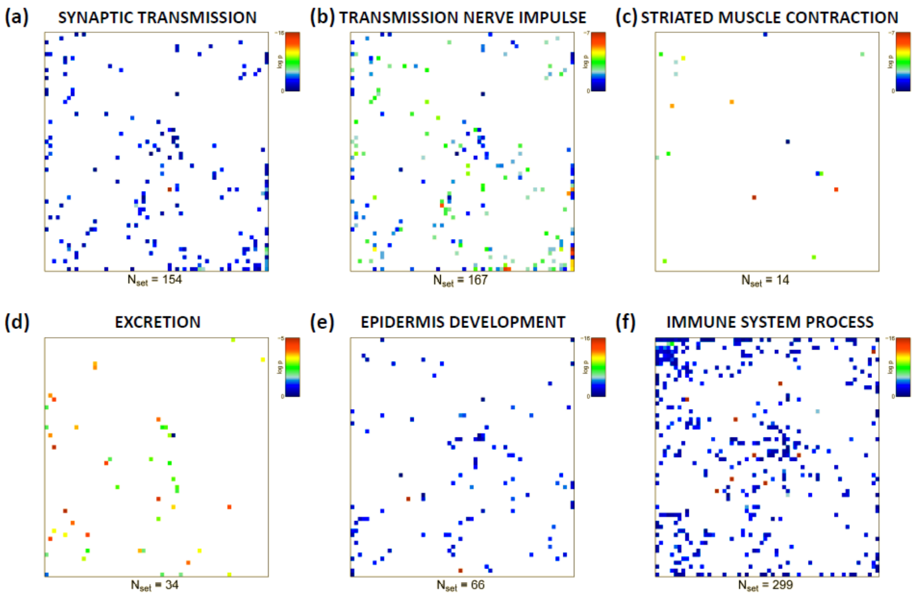

Figure S 13: Overrepresentation maps of six selected gene sets for the zoom-in SOM of nervous tissues. Overrepresentation in each tile of the mosaic is calculated in units of log(p_{HG}) using the hypergeometric distribution and color-coded (maroon>red>yellow>green>blue). White areas indicate metagenes not containing genes from the respective set).

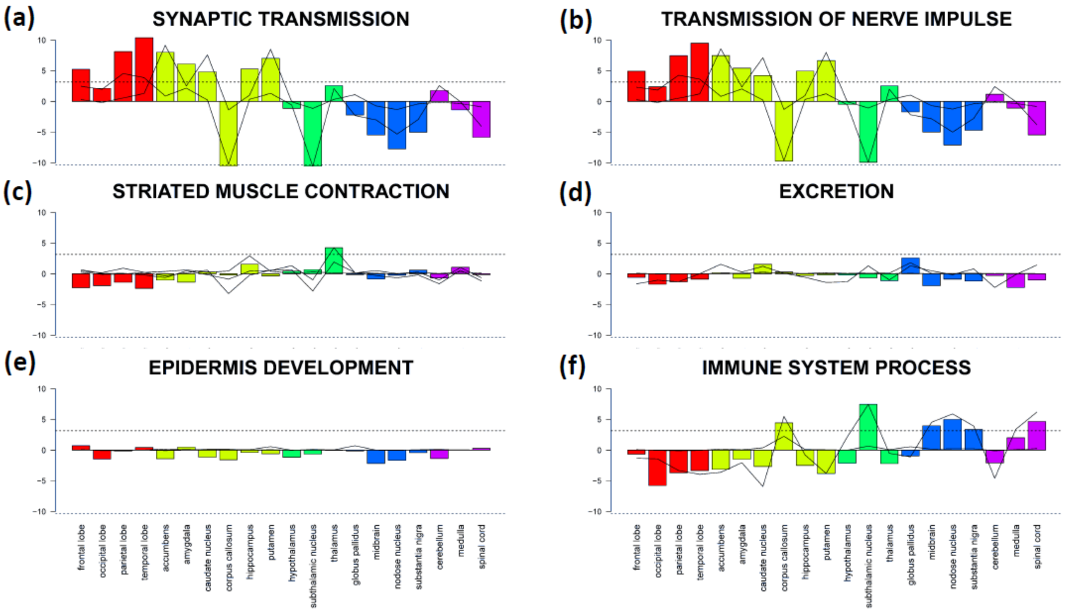

Figure S 14: Overexpression profiles of selected gene sets in nervous tissues. The bars are colored in accordance to the color-codes of the tissue categories. They are scaled in units of the GSZ-score (left axis). The horizontal dotted lines mark the fdr=0.2 significance threshold estimated from the p-value distribution of the GSZ-score. The inserted curves show the logged FC-expression profiles of the top-three metagenes of strongest enrichment of the respective gene set.

12. Zoom-in: Immune systems tissues

Figure S 15 shows the global spot overexpression maps after zoom-in of immune system tissues, and Figure S 16 below the respective GSZ-enrichment heatmap. Both approaches of gene set analysis provide consistent results where local GSZ-enrichment analysis shows a slightly more diverse pattern than global overrepresentation analysis. Note however, that the overexpression spot maps list only the three leading gene sets. Extended lists are available in the detailed reports described below.

The respective overrepresentation (Figure S 17) and overexpression profiles (Figure S 18) show that gene sets which are obviously not related to these tissue categories (e.g. 'synaptic transmission') are virtually invariant and accumulate around the centre of the map. On the other hand, gene sets related to selected tissues accumulate in special regions of the maps and show heterogeneous overexpression (see, for example, the gene sets 'striated muscle contraction' and 'epidermis development' in Figure S 21).

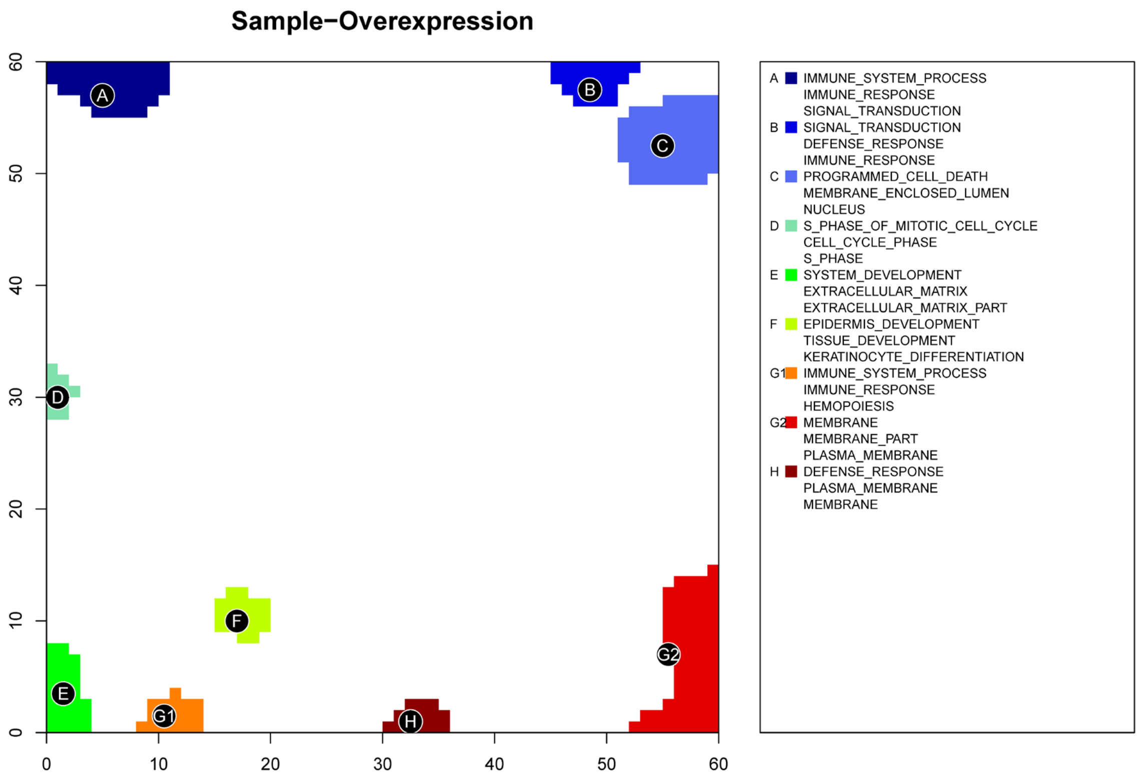

Figure S 15: The overexpression summary map of immune systems tissues shows nine spots (A – H) which are strongly overexpressed in at least of the 11 immune systems tissues studied. Global overrepresentation analysis is estimated for each spot using the hypergeometric distribution. The right legend assigns the most significantly overrepresented gene sets in each spot. Expression heatmaps of the spots are shown in the supplementary material of ref. [7].

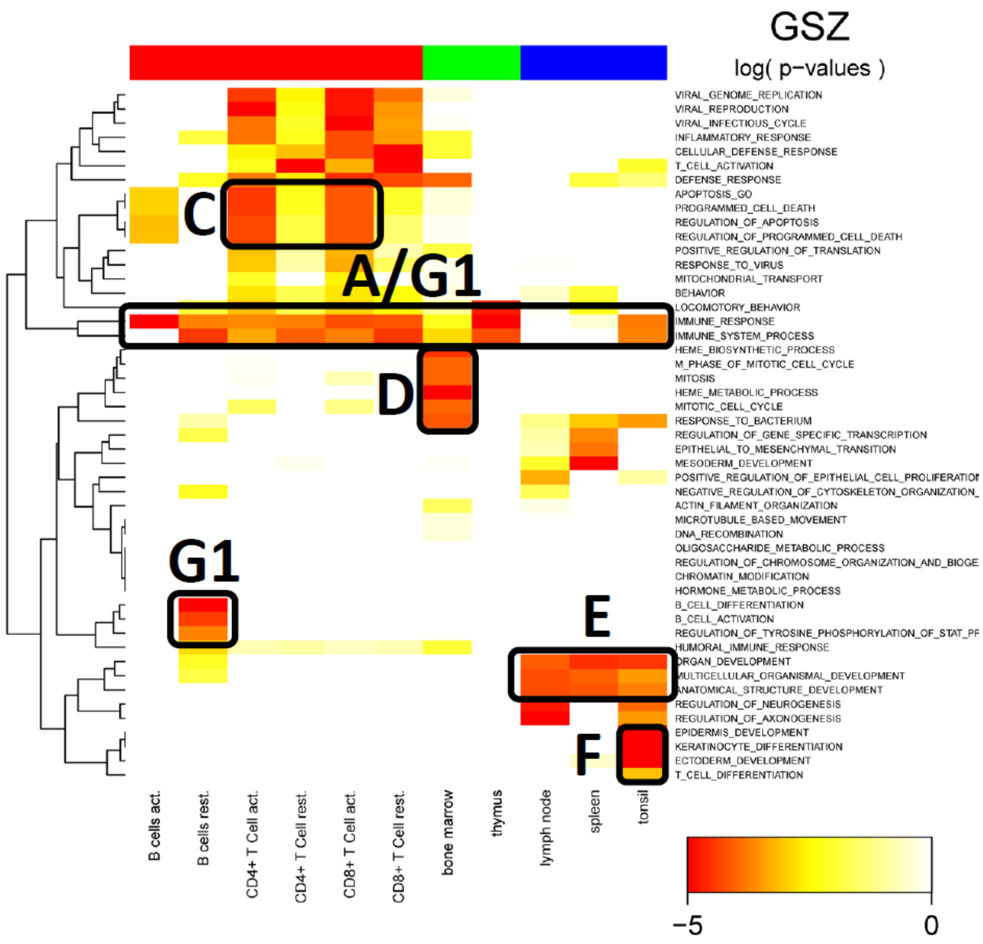

Figure S 16: One-way hierarchical clustering heatmap of significantly enriched gene sets (rows) in immune systems tissues (columns) using the GSZ-statistics. The top-three gen sets per overexpression spot are selected. The heatmap color codes the p-values of the respective score in log-scale (see the legends in the figure). The tissue categories are color coded in the bar above the heatmap according to the assignments given in [7]. The gene sets are clustered in vertical direction. The capital letters assign clusters of enriched gene sets in correspondence with the spots shown in Figure S 15.

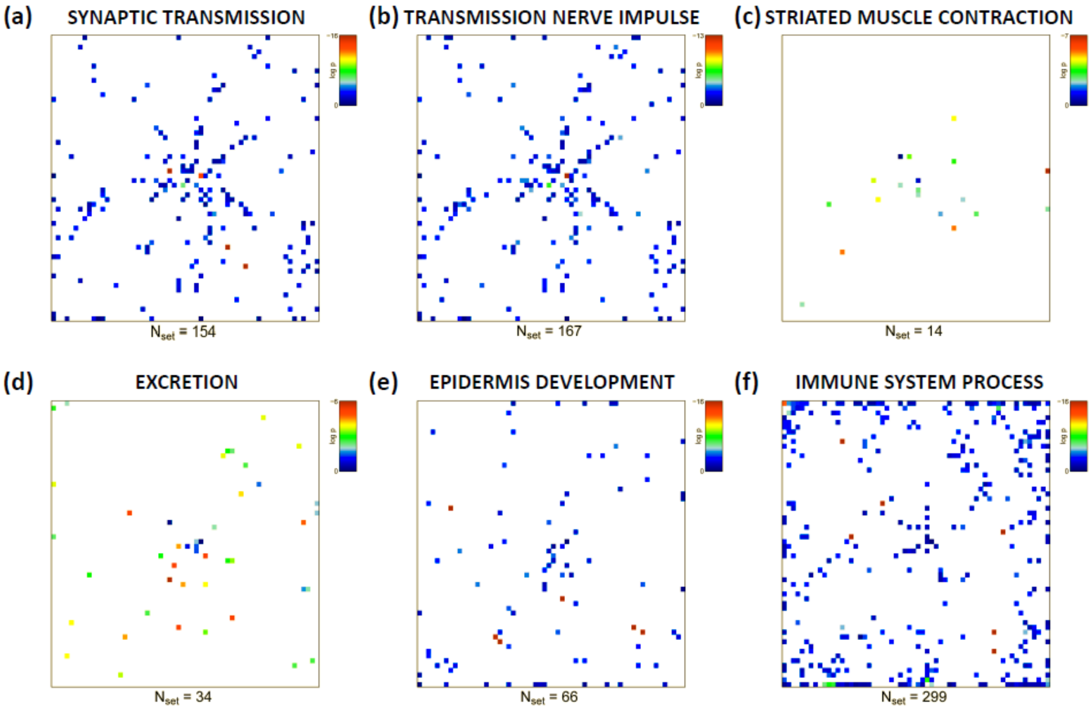

Figure S 17: Overrepresentation maps of six selected gene sets for the zoom-in SOM of immune system tissues.

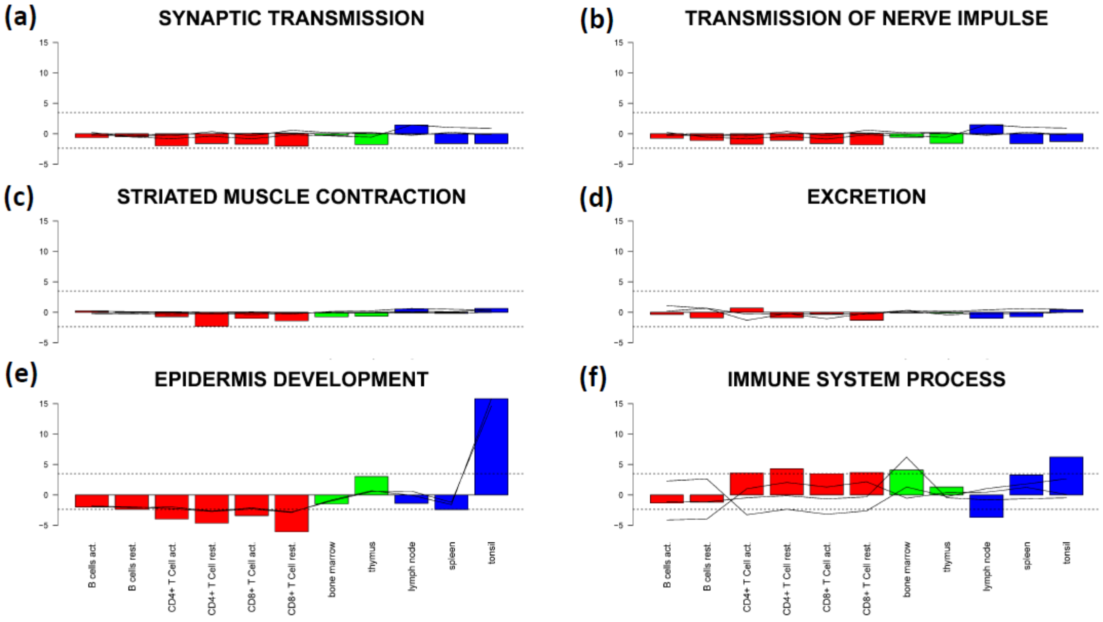

Figure S 18: Overexpression profiles of selected gene sets in immune system tissues.

13. Zoom-in: Diverse tissues

The collection of 'diverse' tissues subsumes the categories adipose, endocrine, homeostasis, digestion, exocrine, epithelium and muscle tissues which cluster relatively tightly together in the agglomerative analyses provided previously. Figure S 19 shows the global spot overexpression maps after zoom-in, and Figure S 20 below the respective GSZ-enrichment heatmap. Both approaches of gene set analysis show consistent results where local GSZ-enrichment analysis provides a slightly more diverse pattern than global overrepresentation analysis.

The respective overrepresentation (Figure S 21) and overexpression profiles (Figure S 22) show that gene sets which are obviously not related to these tissue categories (e.g. 'synaptic transmission') are virtually invariant and accumulate around the centre of the map. On the other hand, gene sets related to selected tissues accumulate in special regions of the maps and show heterogeneous overexpression (see, for example, the gene sets 'striated muscle contraction' and 'epidermis development' in Figure S 21 and Figure S 22).

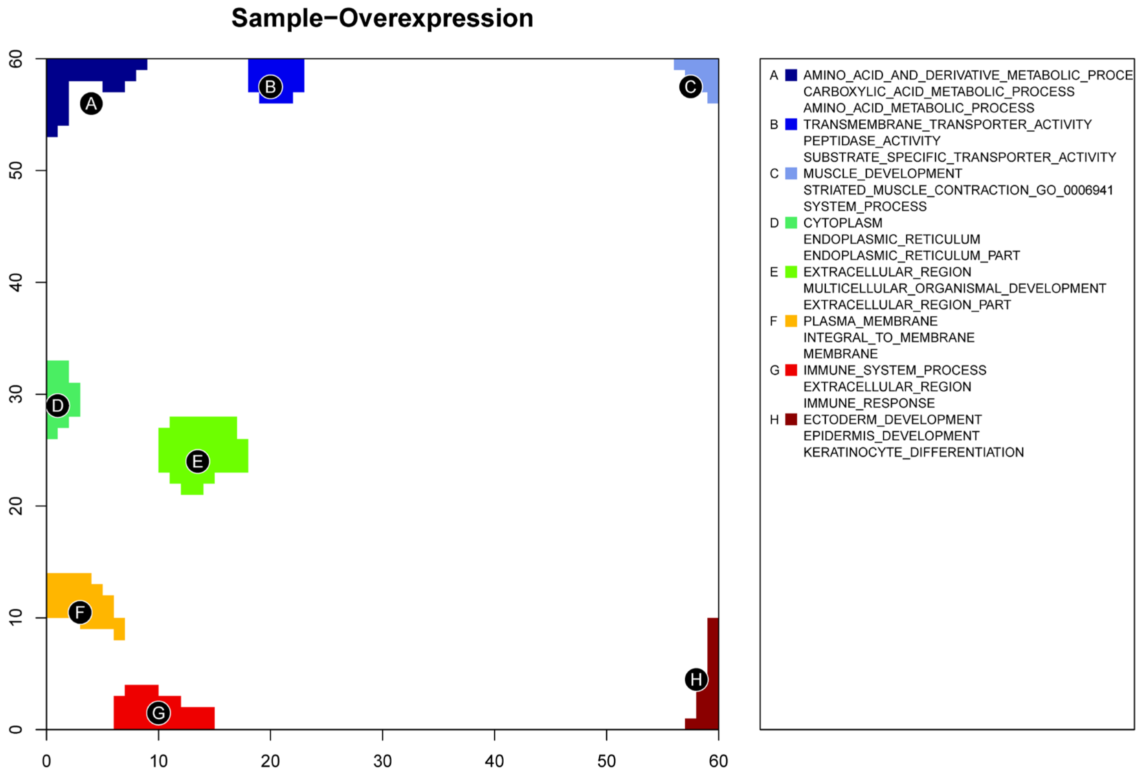

Figure S 19: The overexpression summary map of the group of 'diverse' tissues shows ten spots (A – J). The right legend assigns the two most significantly overrepresented gene sets in each spot. Expression heatmaps of the spots are shown in the supplementary material of ref. [7].

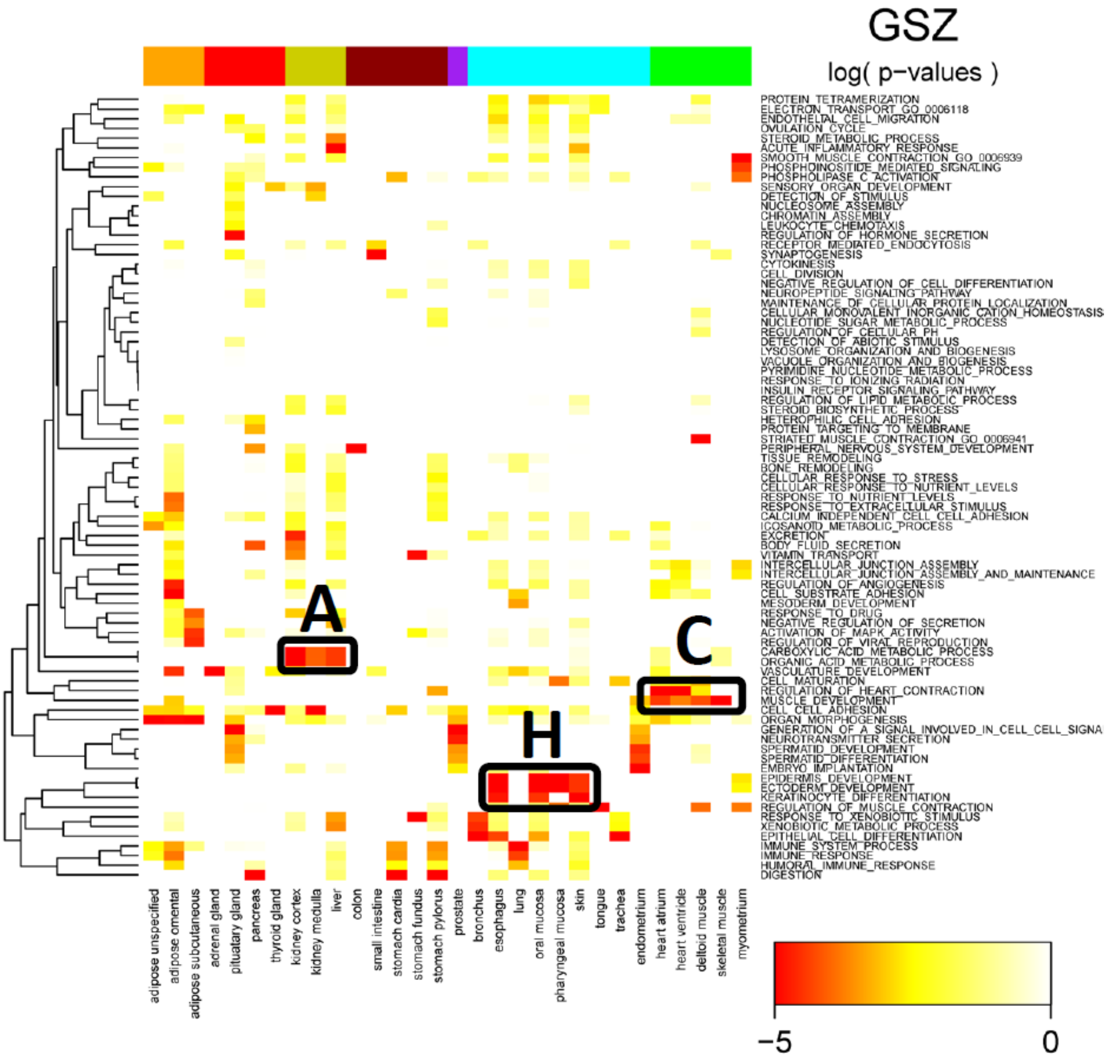

Figure S 20: One-way hierarchical clustering heatmap of significantly enriched gene sets (rows) in the selection of diverse tissues (columns) using the GSZ-statistics. The top-three gen sets per overexpression spot are selected. The heatmap color codes the p-values of the respective score in log-scale (see the legends in the figure). The tissue categories are color coded in the bar above the heatmap according to the assignments given in [7]. The gene sets are clustered in vertical direction.

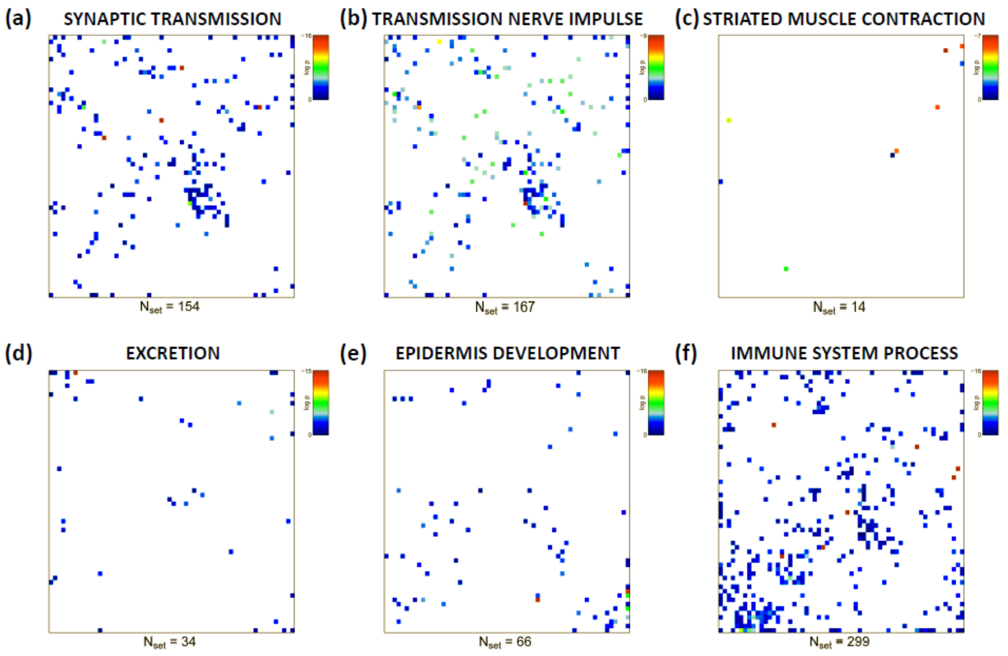

Figure S 21: Overrepresentation maps of six selected gene sets for the zoom-in SOM of the group of diverse tissues.

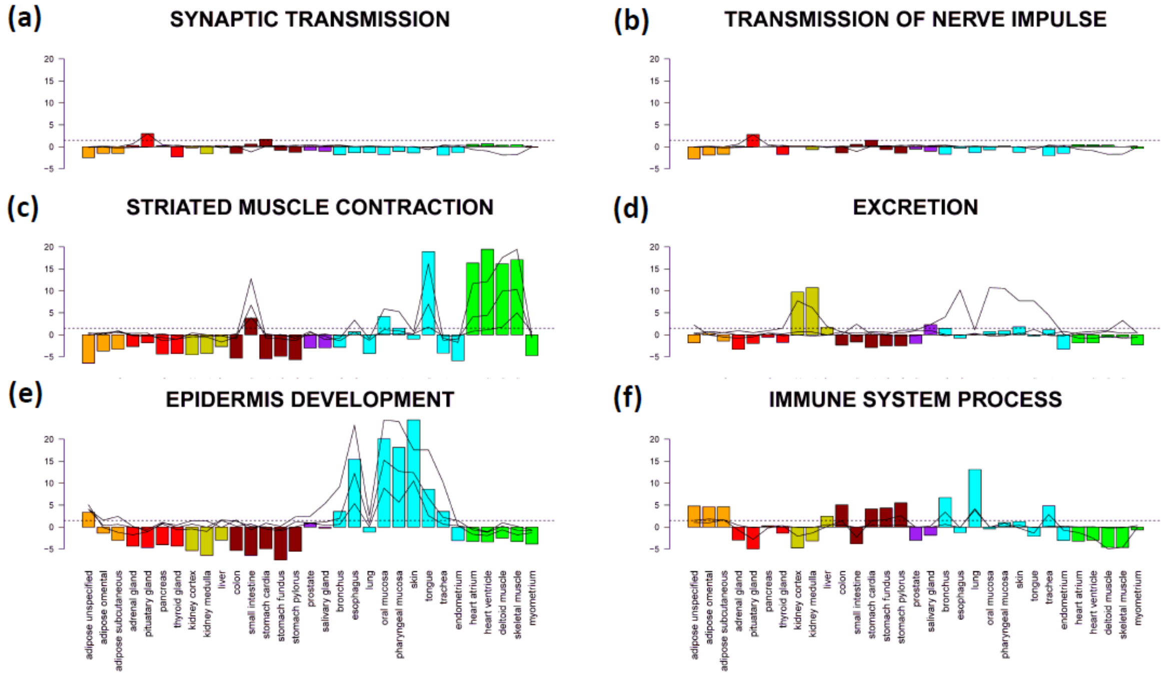

Figure S 22: Overexpression profiles of selected gene sets in the group of diverse tissues.

14. Selecting special sets of genes using ranking and expression criteria

We applied different criteria to select genes which are consistently expressed in all tissues studied. The first method uses the rank product approach [14]: The genes are ranked with decreasing expression score in each tissue. Then, the genes are re-ordered according the geometric mean of their ranks averaged over all tissue lists (i.e. by calculating the product of the tissue-specific ranks of each gene). Panel a of Figure S 23 shows the obtained logged average rank, $\log_{10} < r >= \frac{\log_{10} \text{RankProduct}_r}{M}$ as function of rank number r for the three alternative scores, FC, WAD and t-shrinkage. The initial part of the curves steeply increases. It collects the genes which are consistently ranked on top of the individual tissue lists with small rank numbers. The slope of the curves markedly drops and virtually levels off for rank numbers greater than 3,000 revealing a background floor of weakly expressed genes with almost high rankings in the individual tissue lists. The three alternative scores provide very similar curves showing a transition between both classes of consistently high and weak expressed genes near r= 1000- 3000. We arbitrarily select 10% of the genes on top of the lists as consistently expressed (2,227). The slightly smaller value of the mean rank of the FC-scores in the transition range indicates the slightly better consistency of the FC-score at ranks smaller than 3000 compared with the alternative scores (red curve in Figure S 23a).

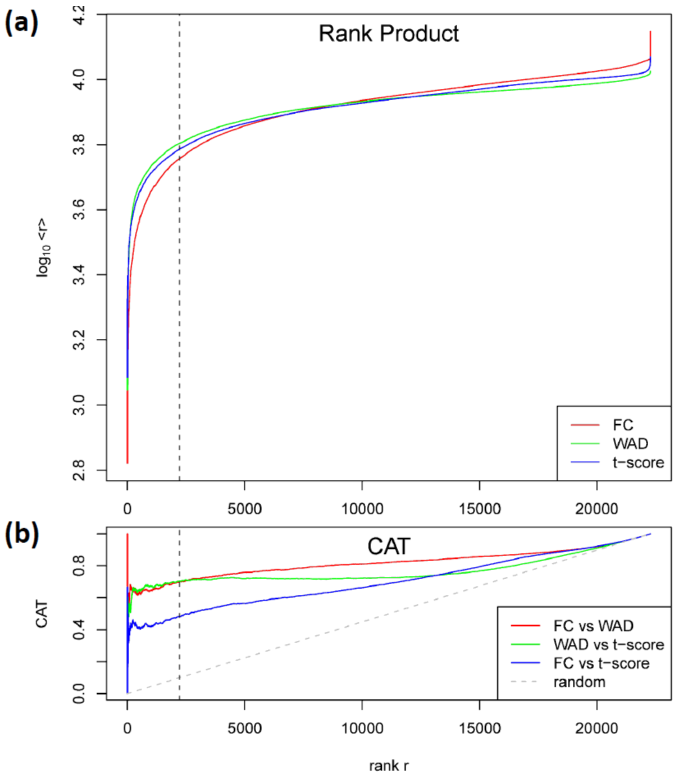

Figure S 23: Logged rank product of ranked gene lists of all 67 tissues as a function of the gene index. 2,227 genes are selected on top of the list over the range of steep slope as 'consistently high ranked' (see the vertical dashed line). Three alternative scores are applied for gene ranking, FC, WAD and t-shrinkage. Panel b of the figure shows pairwise comparisons of the ranked lists in terms of the CAT-measure. FC and WAD are most similar whereas FC and t-score deviate considerably.

Panel b of Figure S 23 compares the gene lists obtained from the different scores using the respective CAT-plots of all three pairwise combinations of the respective lists. For the FC/WAD- and WAD/t-pairings of lists we found overlap of about 70% of the genes for rankings r< 2000 whereas FC/t-pairings are common to only 50% in this range. This result is slightly different if compared with the CAT-values of the tissue specific lists (see Figure S 4 and the main paper). In these comparisons the FC/WAD-lists best agree to about 70% whereas WAD/t- and FC/t- lists overlap to 50% only. Hence, rank-averaging over all tissues slightly modifies the overlap between the different lists. Nevertheless,

the observed differences are relatively small confirming our conclusion that all scores considered provide reliable and partly complementary results.
In addition to the rank product approach we applied another one which makes use of the present call parameter, $0 \leq pc \leq 1$, estimated in the normalization step of gene expression data (see ref. [7] for details). Figure S 24 shows the distribution of the number of genes significantly expressed in a certain number of tissues. The histogram ranges from genes which are consistently absent in all tissues (with present calls $pc<1$) to genes consistently present ($pc=1$) in all tissues. Interestingly, the distribution shows maxima at their left and right borders. This result reveals that many genes tend to be expressed either in most of the tissues or in only a few ones. A number of about 200 – 300 genes forms a sort of constant background level which characterizes the incremental cumulative tissue specificity of gene expression. Eisenberg and Lavanon [15] obtained a similar histogram using an alternative tissue data set. We collect the genes which are strictly absent or strictly present in all tissues into two groups for further analysis (see also Figure S 24).

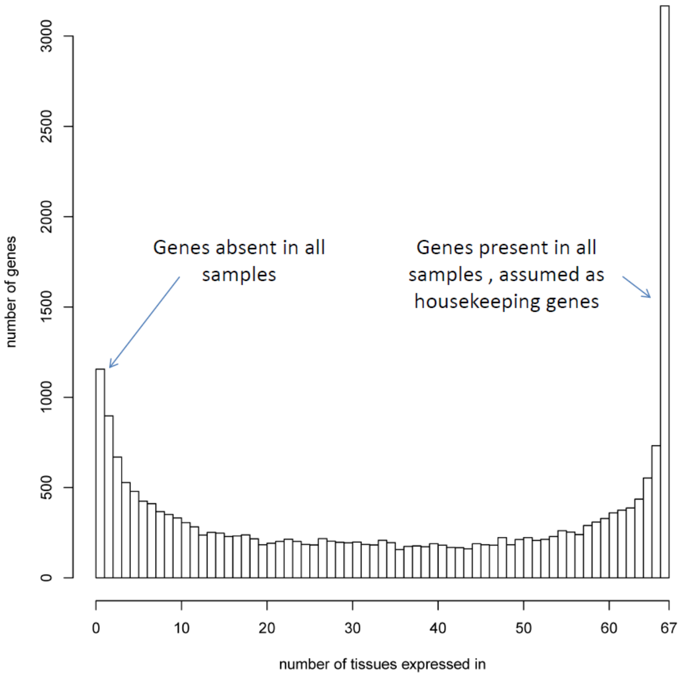

Figure S 24: Histogram of the number of genes expressed in different numbers of tissues using the present-call criterion. Absent genes (not expressed in any tissue) and housekeeping genes (expressed in all tissues) are found at the left and right positions, respectively. Please note the histogram-width equals one. The height of the bars consequently indicates the number of genes expressed in no, one, two…..all 67 tissues (from the left).

15. Reports

Our SOM analysis produces a series of reports which characterize gene expression of the samples studied:

(i) Maps (experiment atlas)

These reports show the collection of first level SOM of all tissue samples, supporting maps and the second level SOM as described in ref. [7]. First level SOM profiles are shown with different contrast (log FC-, WAD- and double log-scale) and also as rank-maps using the different scores as described above.

(ii) Metagene and enrichment analysis

Several agglomerative methods based either on distance or on correlation metrics are applied to the samples using filtered subsets of metagenes. The reports show the respective two-way hierarchical clustering heatmaps, pairwise correlation maps, minimum spanning trees and the results of independent component analysis [7]. In addition, GSZ- and HG-enrichment clustering heatmaps are available to associate the most relevant functional gene sets with the different samples. This information is supplemented by the respective p-value distributions to assess the quality of the data.

(iii) Sample summaries

For each sample we generated one PDF-report which summarizes the most relevant information using the global (i.e. sample-centered) and local (i.e. spot-centered) perspective as well. The collection of all spots detected in the respective sample is depicted in the right small map shown in the 'Global summary' sheet. The small map shown in the 'Local summary' sheet depicts the spot selected for analysis. Figure S 25 presents the PDF-report for one particular tissue (accumbens). Table S 4and Table S 5 provide glossaries of the data given in the sheets. The global summary shows the ranked list of differentially expressed genes together with the associated significance characteristics for the whole sample, the ranked list of over- and underexpressed gene sets after GSZ-overexpression analysis and the respective p-value distributions. The local summary sheets present the analogous information for each single spot which is selected using the 98%-quantile criterion. The two maps in the left part of the sheet show the respective first level SOM and the selected spot, respectively. The full global and local lists can be downloaded in excel format for detailed inspection and further processing.

(iv) Spot summaries

The sample summary reports collect analysis data for each sample either considering all genes or genes taken from a selected spot as described in the previous subsection. The spot summary reports pursue the orthogonal view: Each relevant over-/underexpression spot is characterized across all samples. Figure S 26 and Table S 6 show one example and the glossary of data listed in the sheet, respectively. These analyses apply different criteria of spot selection such as overexpression, underexpression, maximum and minimum of metagene expression and mutual correlations between the metagenes. GO-enrichment analysis provides the three leading genes in the respective HG-enrichment list of each of the spots considered. Spot-related heatmaps characterize the expression profiles of the selected features in the series of samples. Single spot summary sheets provide detailed information about each of the spots such as the ranked list of samples which overexpress this feature according to the mean t-shrinkage statistics of the spot and the ranked list of the top-twenty HG-overrepresented gene sets together with the histogram of the respective p-value distribution.

(v) GSZ-enrichment profiles

Enrichment maps and profiles of individual GO gene sets are shown as bar plots and provided as excel-files for download (Additional file 6). These cross-tissue characteristics are supplemented by the log FC-expression profiles of the leading metagenes of the respective gene set.

Table S 4: Glossary of data given in the global summary sheet shown in Figure S 25a

	**Description**
**1**	Sample name
**2**	General characteristics: number of differentially expressed genes (“#DE”); numbers of genes below particular fdr thresholds (“#genes with fdr < …”); number of genes covered by GO genesets (“#genes in genesets”); average scores (“<FC>”, “<shrinkage-t>”, “<p-value>”, “<fdr>”) over all genes
**3**	Expression profile (SOM) & map showing all spots selected using the 98%-quantile criterion
**4**	Ranking of differentially expressed genes: Affymetrix gene id (“Affy-ID”), gene symbol (“Symbol”), $\log_{10}$ fold-change (“log(FC)”), p-value(“p-value”), fdr (“fdr”), x-y-position of associated metagene in expression profile (“Metagene”, x-y are the tile numbers in horizontal and vertical directions, respectively) and GO-term (“GO-Term”)
**5**	Distribution of p-values from gene list (4) along with FDR-analysis: η_0 level is shown as horizontal line, fdr and Fdr are shown as dotted and dashed lines, respectively
**6**	GSZ enrichment list of top-20 over- and underexpressed genesets: GSZ score (“GSZ”) and p-value (“p-value”), number of genes in particular set (“#in”), GO-category and -term (“Geneset”)
**7**	Distribution of p-values from GSZ enrichment analysis (6), analogous to (5)

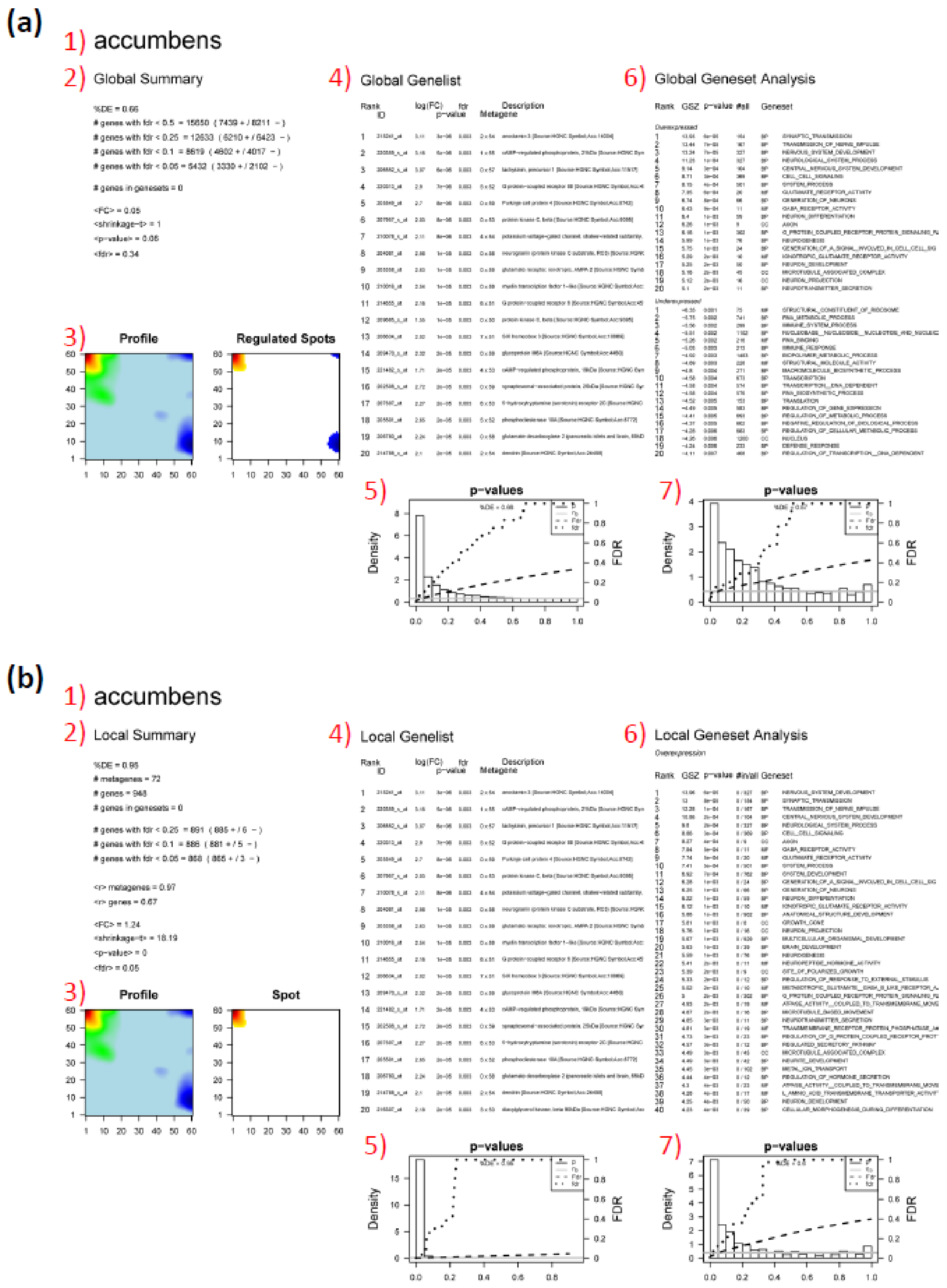

Figure S 25: Global (panel a) and local summary sheet (panel b) of accumbens. Note that only one out of three local summary sheets of this particular tissue is shown as example.

Table S 5: Glossary of data given in the local summary sheet shown in Figure S 25b

	**Description**
**1**	Sample name
**2**	General characteristics: number of differentially expressed genes (#DE); numbers of genes & metagenes in current spot; numbers of genes below particular fdr thresholds ("#genes with fdr < …"); number of genes covered by GO genesets ("#genes in genesets"); average scores (correlation coefficient r ("<r>"), "<FC>", "<shrinkage-t>", "<p-value>", "<fdr>") over spot genes
**3**	Expression profile (SOM map) & the local spot analyzed
**4**	Ranking of differentially expressed genes: Affymetrix gene id ("Affy-ID"), gene symbol ("Symbol"), $\log_{10}$ fold-change ("log(FC)"), p-value("p-value"), fdr ("fdr"), x-y-position of associated metagene in expression profile ("Metagene", x-y are the tile numbers in horizontal and vertical directions, respectively) and GO-term ("GO-Term")
**5**	Distribution of p-values from gene list (4) along with FDR-analysis: η_0 level is shown as horizontal line, fdr and Fdr are shown as dotted and dashed lines, respectively
**6**	GSZ enrichment list of top-40 over- resp. underexpressed genesets: GSZ score ("GSZ") and p-value ("p-value"), numbers of genes in particular geneset (#all) and associated genes found in current spot (#in), GO-category and -term ("Geneset")
**7**	Distribution of p-values from GSZ enrichment analysis (6), analogous to (5)

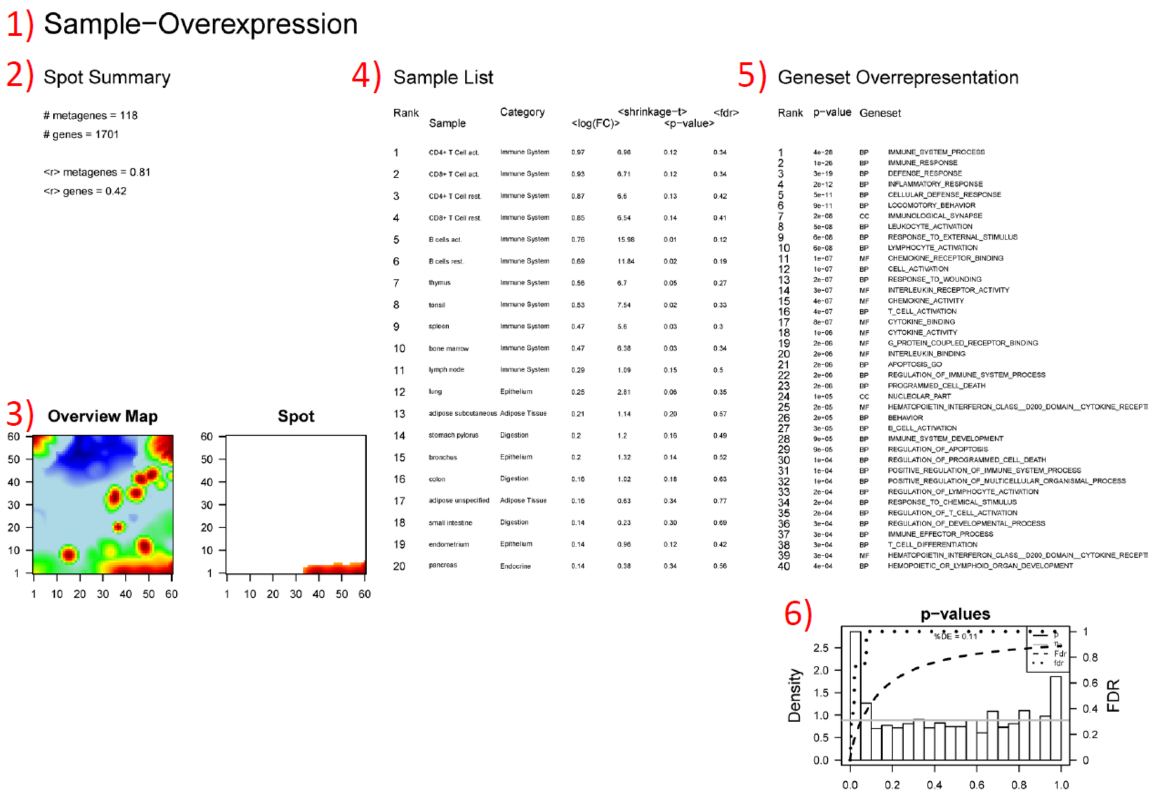

Figure S 26: Spot summary sheet for the ‘immune systems’ spot F.

Table S 6: Glossary of data given in the spot summary sheet shown in Figure S 26

	**Description**
**1**	Summarization criterion
**2**	General characteristics: numbers of genes (“#genes”) & metagenes (“#metagenes”) in current spot; average correlation coefficient (“<r>”) among spots genes & metagenes
**3**	Summary map & mal showing the analyzed spot
**4**	Ranking of samples according to average fold change of genes within current spot: sample name (“Sample”); average scores (“<FC>”, “<shrinkage-t>”, “<p-value>”, “<fdr>”) of respective sample
**5**	HG-overrepresentation list: p-value (“p-value”), GO-category and –term (“Geneset”) are given for top-40 genesets
**6**	Distribution of p-values from HG-overrepresentation list (5) along with FDR-analysis: η_0 level is shown as horizontal line, fdr and Fdr are shown as dotted and dashed lines, respectively

16. References

[1] H. Binder, T. Kirsten, M. Löffler, and P. F. Stadler, “Sensitivity of microarray oligonucleotide probes: variability and effect of base composition,” *The Journal of Physical …*, 2004.

[2] M. A. Sartor, C. R. Tomlinson, S. C. Wesselkamper, S. Sivaganesan, G. D. Leikauf, and M. Medvedovic, “Intensity-based hierarchical Bayes method improves testing for differentially expressed genes in microarray experiments.,” *BMC bioinformatics*, vol. 7, p. 538, Jan. 2006.

[3] A. Zeisel, A. Amir, W. J. Köstler, and E. Domany, “Intensity dependent estimation of noise in microarrays improves detection of differentially expressed genes.,” *BMC bioinformatics*, vol. 11, p. 400, Jan. 2010.

[4] A.-M. K. Hein, S. Richardson, H. C. Causton, G. K. Ambler, and P. J. Green, “BGX: a fully Bayesian integrated approach to the analysis of Affymetrix GeneChip data.,” *Biostatistics (Oxford, England)*, vol. 6, no. 3, pp. 349-73, Jul. 2005.

[5] N. Jain, J. Thatte, T. Braciale, K. Ley, M. O’Connell, and J. K. Lee, “Local-pooled-error test for identifying differentially expressed genes with a small number of replicated microarrays.,” *Bioinformatics (Oxford, England)*, vol. 19, no. 15, pp. 1945-51, Oct. 2003.

[6] H. Binder, S. Preibisch, and H. Berger, “Calibration of microarray gene-expression data.,” *Methods in molecular biology (Clifton, N.J.)*, vol. 576, pp. 375-407, Jan. 2010.

[7] H. Wirth, M. Loffler, M. von Bergen, and H. Binder, “Expression cartography of human tissues using self organizing maps,” *BMC Bioinformatics*, vol. 12, no. 1, p. 306, 2011.

[8] H. Binder, K. Krohn, and S. Preibisch, “‘Hook’-calibration of GeneChip-microarrays: chip characteristics and expression measures.,” *Algorithms for molecular biology : AMB*, vol. 3, p. 11, Jan. 2008.

[9] H. Binder and S. Preibisch, “‘Hook’-calibration of GeneChip-microarrays: theory and algorithm.,” *Algorithms for molecular biology : AMB*, vol. 3, p. 12, Jan. 2008.

[10] R. Tibshirani and L. Wasserman, “Correlation-sharing for detection of differential gene expression,” *Arxiv preprint math/0608061*, 2006.

[11] H. Yang and G. Churchill, “Estimating p-values in small microarray experiments.,” *Bioinformatics (Oxford, England)*, vol. 23, no. 1, pp. 38-43, Jan. 2007.

[12] Y. Xie, W. Pan, and A. B. Khodursky, “A note on using permutation-based false discovery rate estimates to compare different analysis methods for microarray data.,” *Bioinformatics (Oxford, England)*, vol. 21, no. 23, pp. 4280-8, Dec. 2005.

[13] J. J. Goeman and P. Bühlmann, “Analyzing gene expression data in terms of gene sets: methodological issues.,” *Bioinformatics (Oxford, England)*, vol. 23, no. 8, pp. 980-7, Apr. 2007.

[14] R. Breitling, P. Armengaud, A. Amtmann, and P. Herzyk, “Rank products: a simple, yet powerful, new method to detect differentially regulated genes in replicated microarray experiments.,” *FEBS letters*, vol. 573, no. 1-3, pp. 83-92, Aug. 2004.

[15] E. Eisenberg and E. Y. Levanon, “Human housekeeping genes are compact.,” *Trends in genetics : TIG*, vol. 19, no. 7, pp. 362-5, Jul. 2003.
