# Supplementary material for: Mining SOM expression portraits: feature selection and integrating concepts of molecular function
Source: BioData Min. 2012 Oct 8;5:18. doi: 10.1186/1756-0381-5-18 (PMC3599960; doi:10.1186/1756-0381-5-18)
Supplement: Additional file 2 — Atlas of the ranking maps of all tissues studied. [file 1756-0381-5-18-S2.pdf]

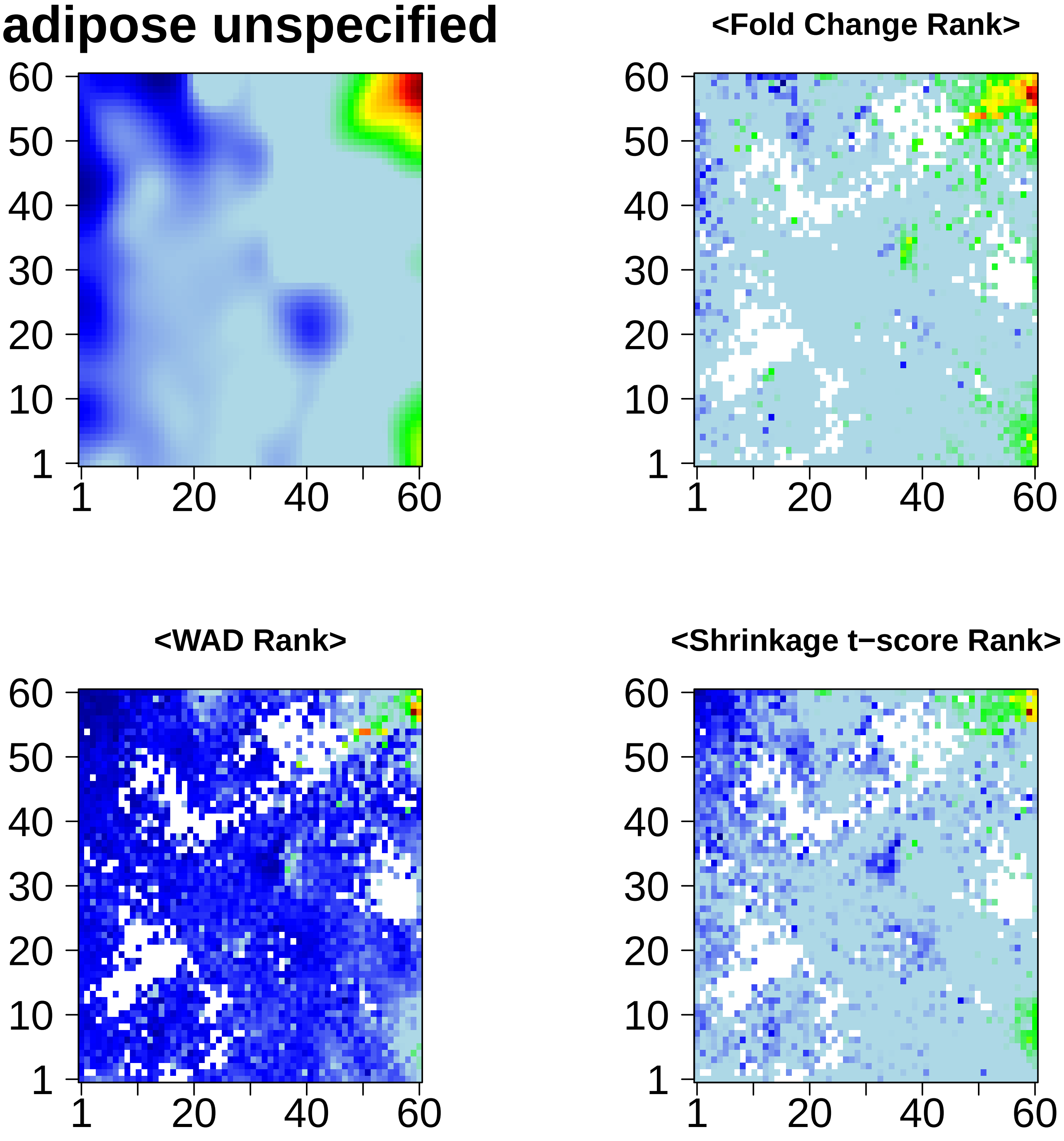
adipose unspecified
<Fold Change Rank>
<WAD Rank>
<Shrinkage t−score Rank>
60
50
40
30
20
10
1
1
20
40
60

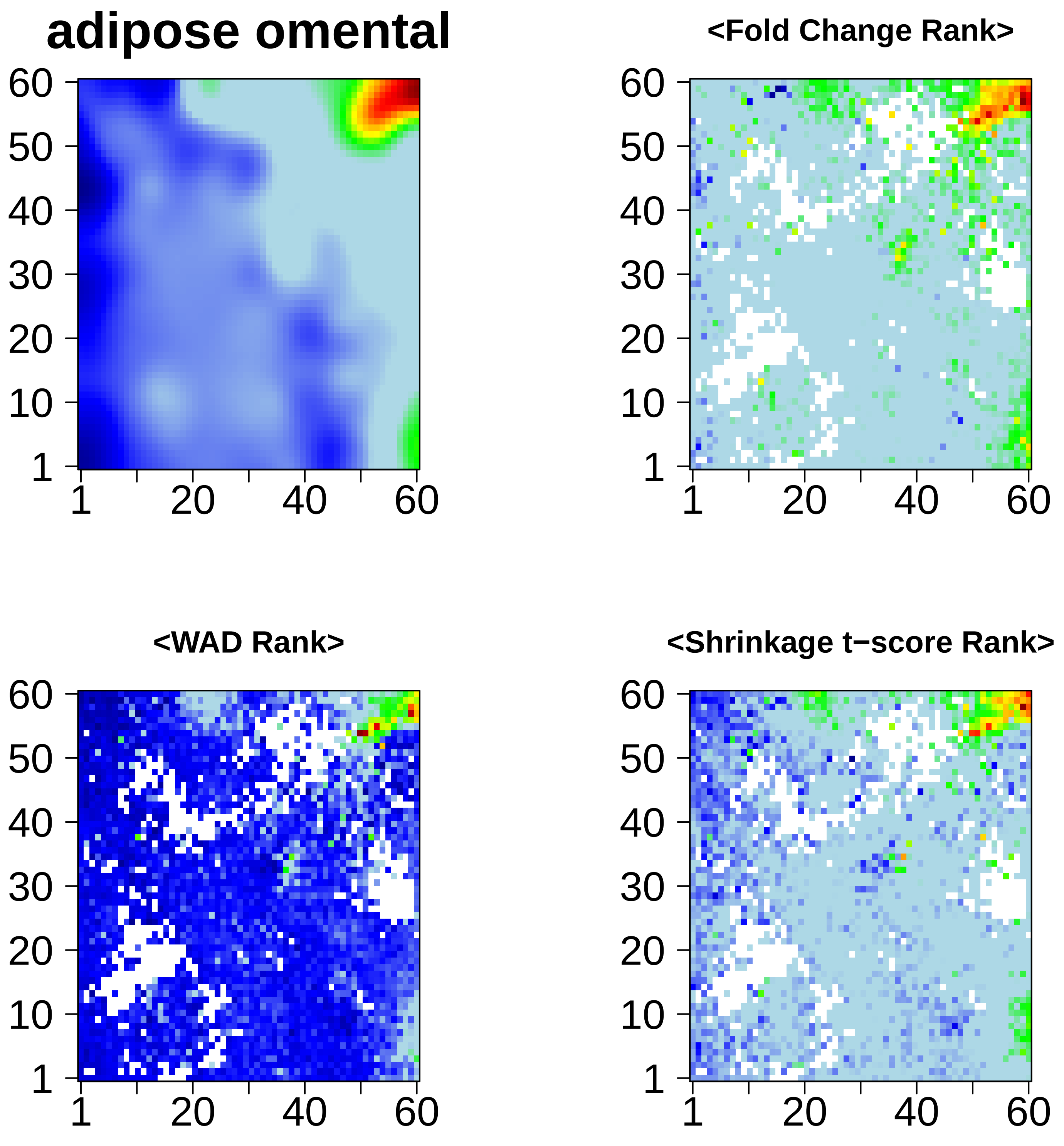
adipose omental
<Fold Change Rank>
<WAD Rank>
<Shrinkage t−score Rank>
60
50
40
30
20
10
1
1
20
40
60

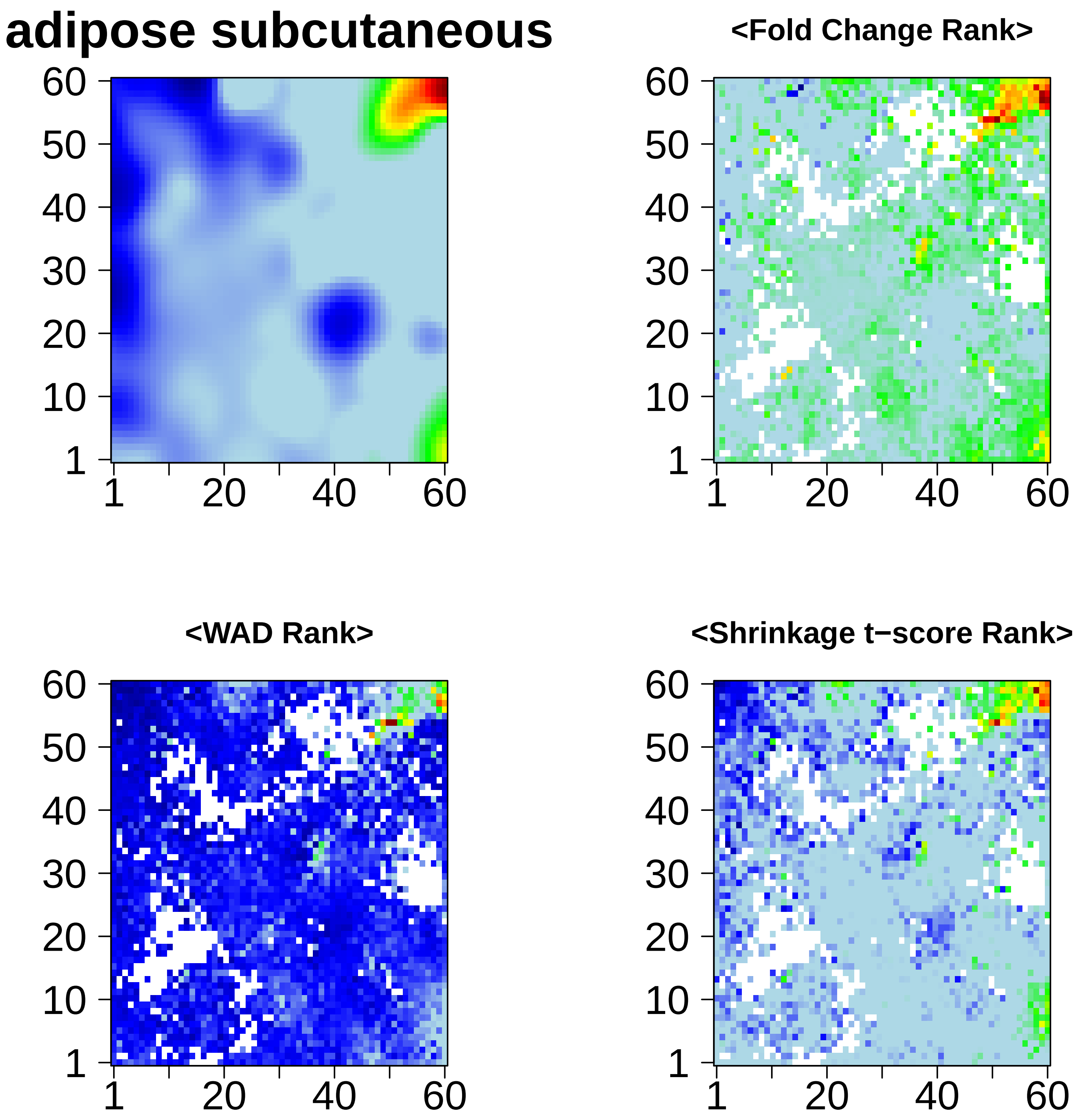
adipose subcutaneous
<Fold Change Rank>
<WAD Rank>
<Shrinkage t−score Rank>
1
10
20
30
40
50
60

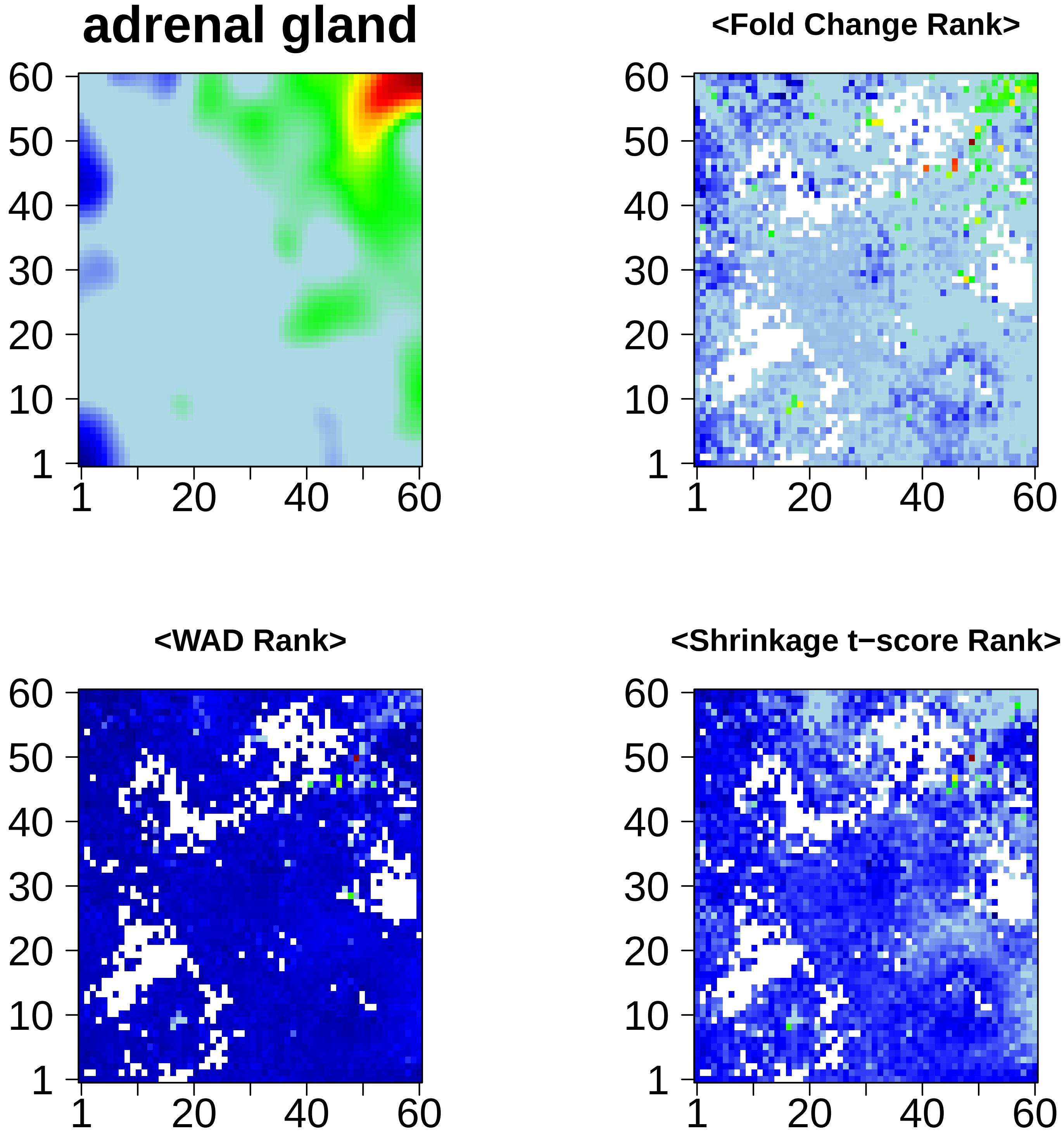
adrenal gland
<Fold Change Rank>
<WAD Rank>
<Shrinkage t−score Rank>
1
10
20
30
40
50
60

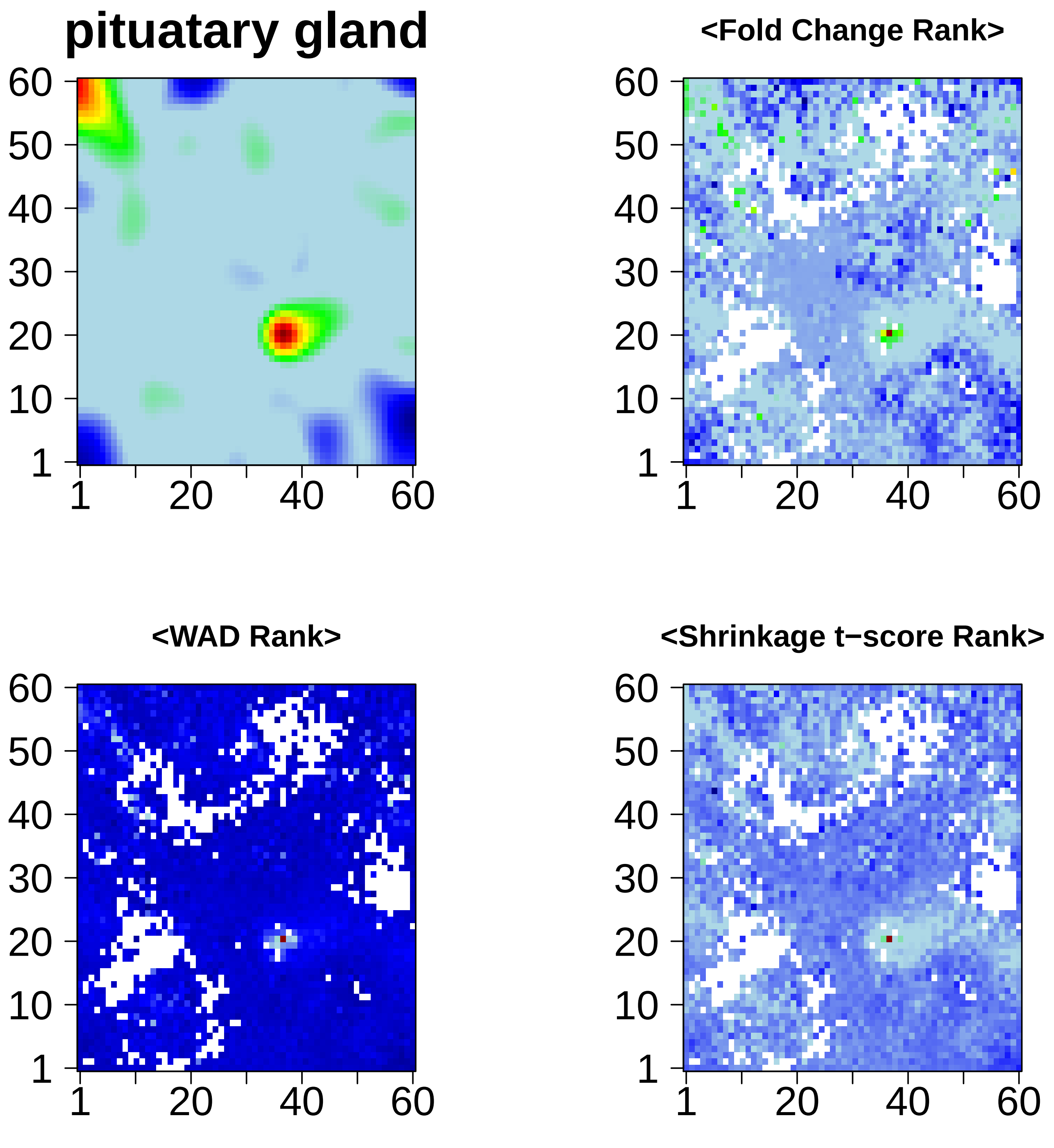
pituatary gland
<Fold Change Rank>
<WAD Rank>
<Shrinkage t−score Rank>
60
50
40
30
20
10
1
1
20
40
60

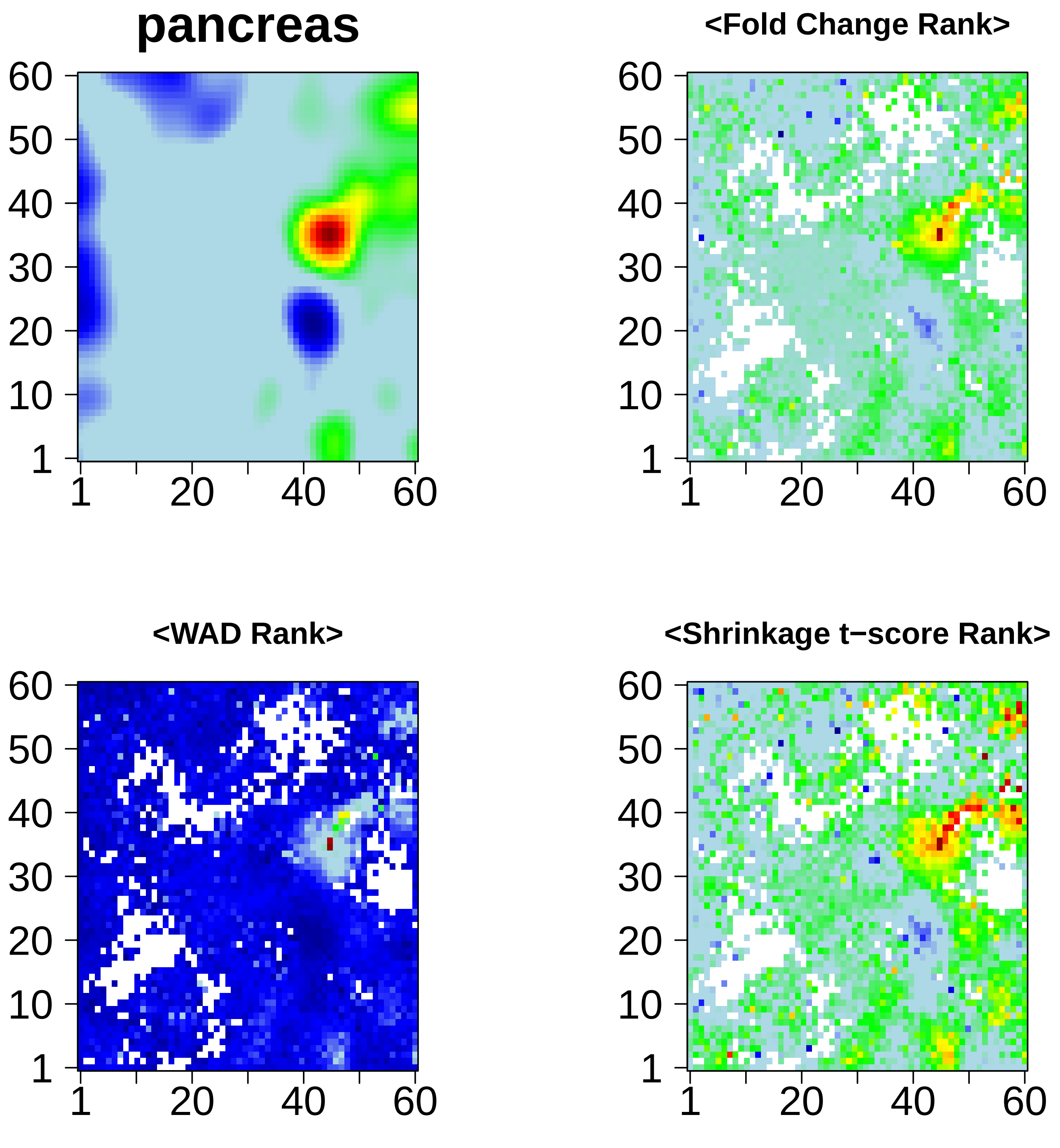
pancreas
<Fold Change Rank>
<WAD Rank>
<Shrinkage t−score Rank>
60
50
40
30
20
10
1
1
20
40
60

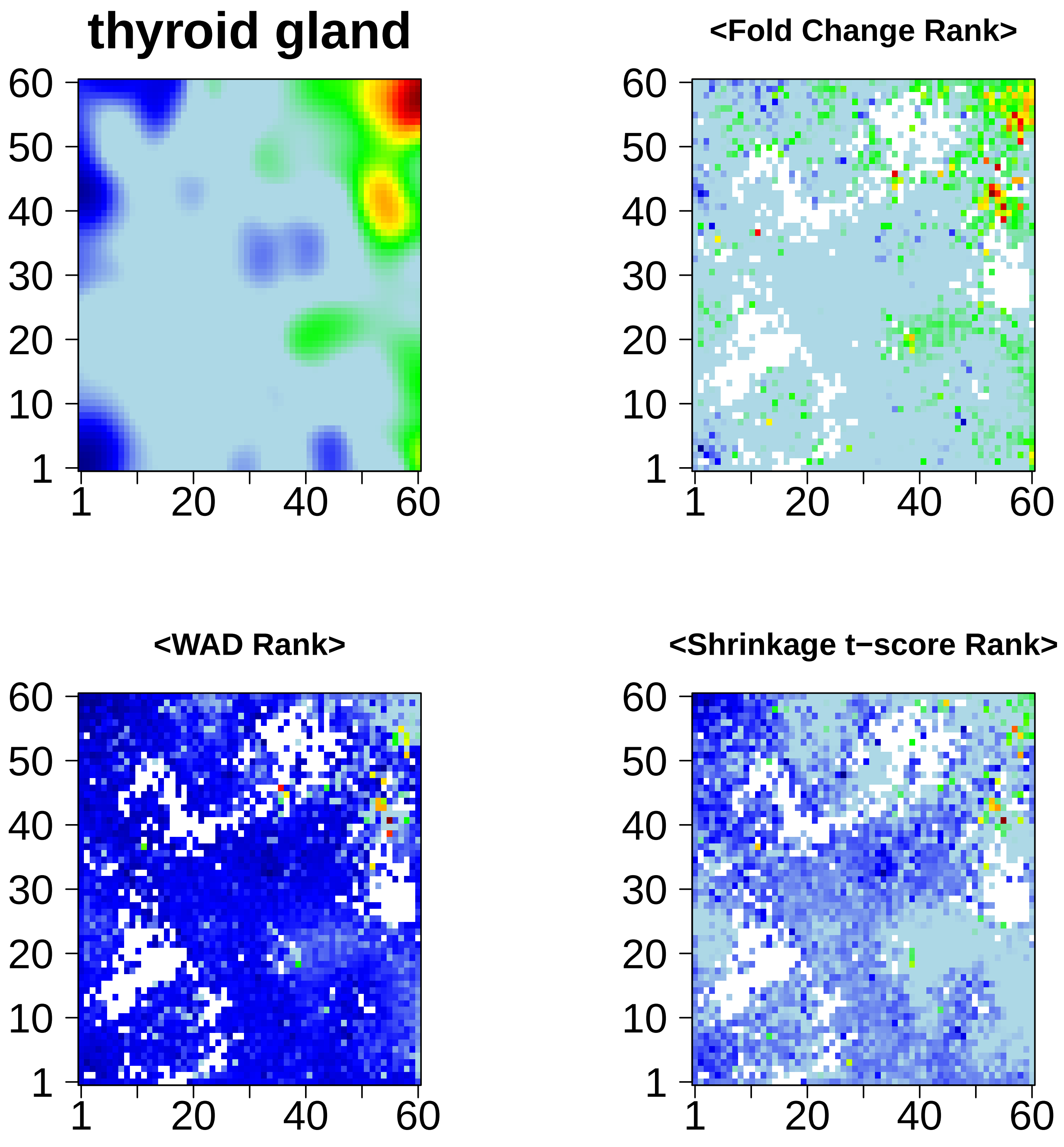
thyroid gland
<Fold Change Rank>
<WAD Rank>
<Shrinkage t−score Rank>
60
50
40
30
20
10
1
1
20
40
60

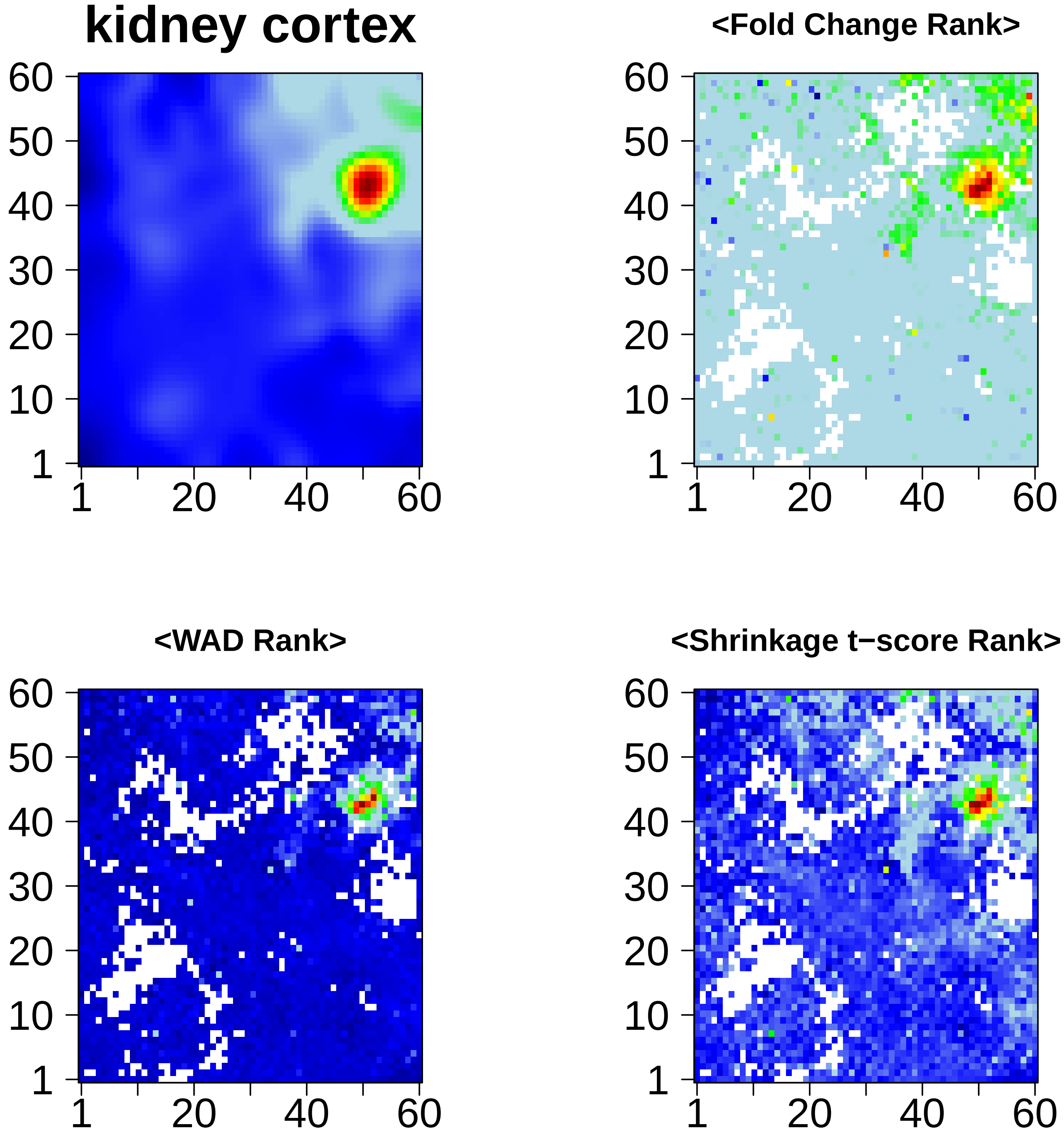

kidney cortex
<Fold Change Rank>
<WAD Rank>
<Shrinkage t−score Rank>
60
50
40
30
20
10
1
1
20
40
60

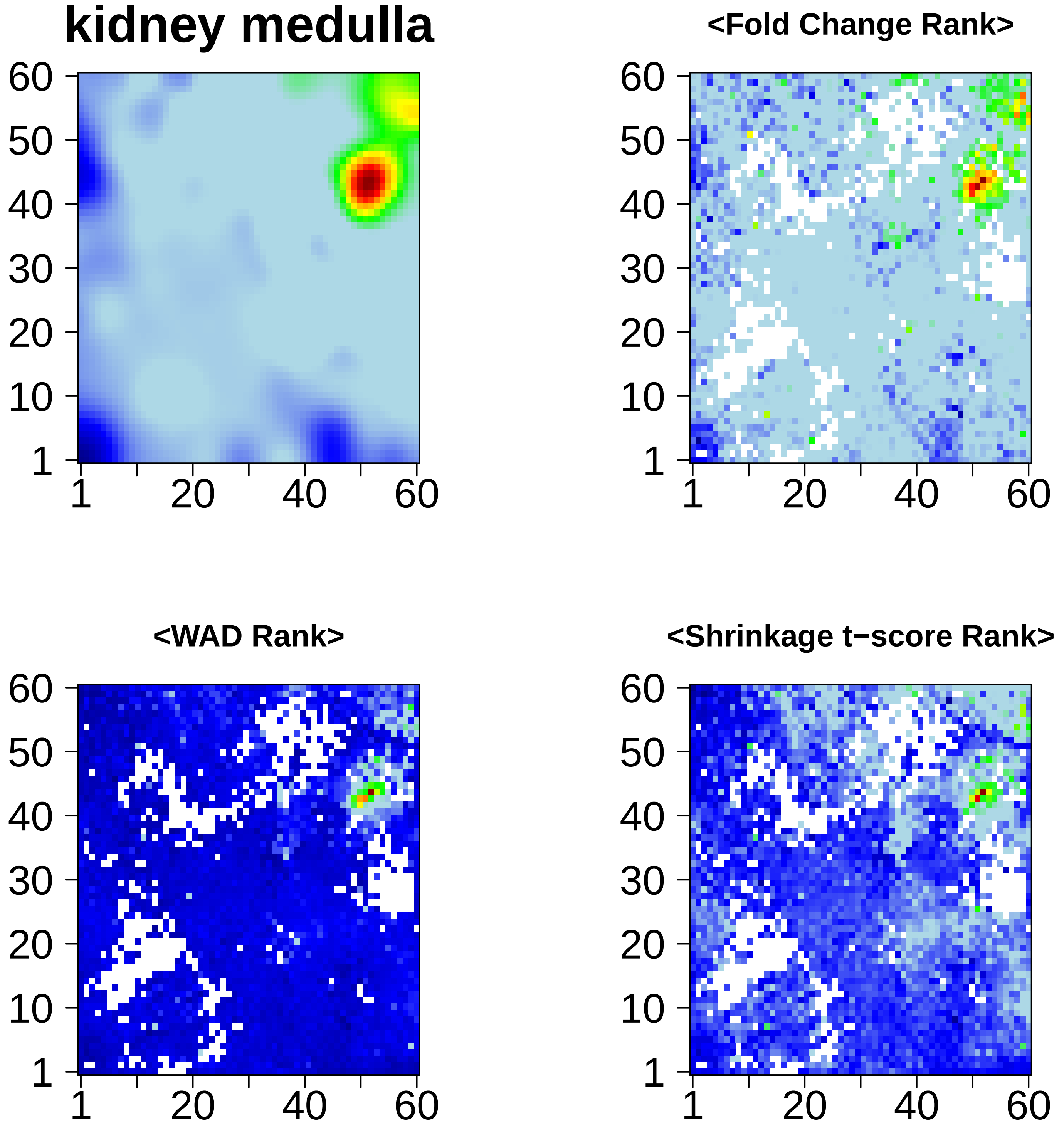

kidney medulla
<Fold Change Rank>
<WAD Rank>
<Shrinkage t−score Rank>

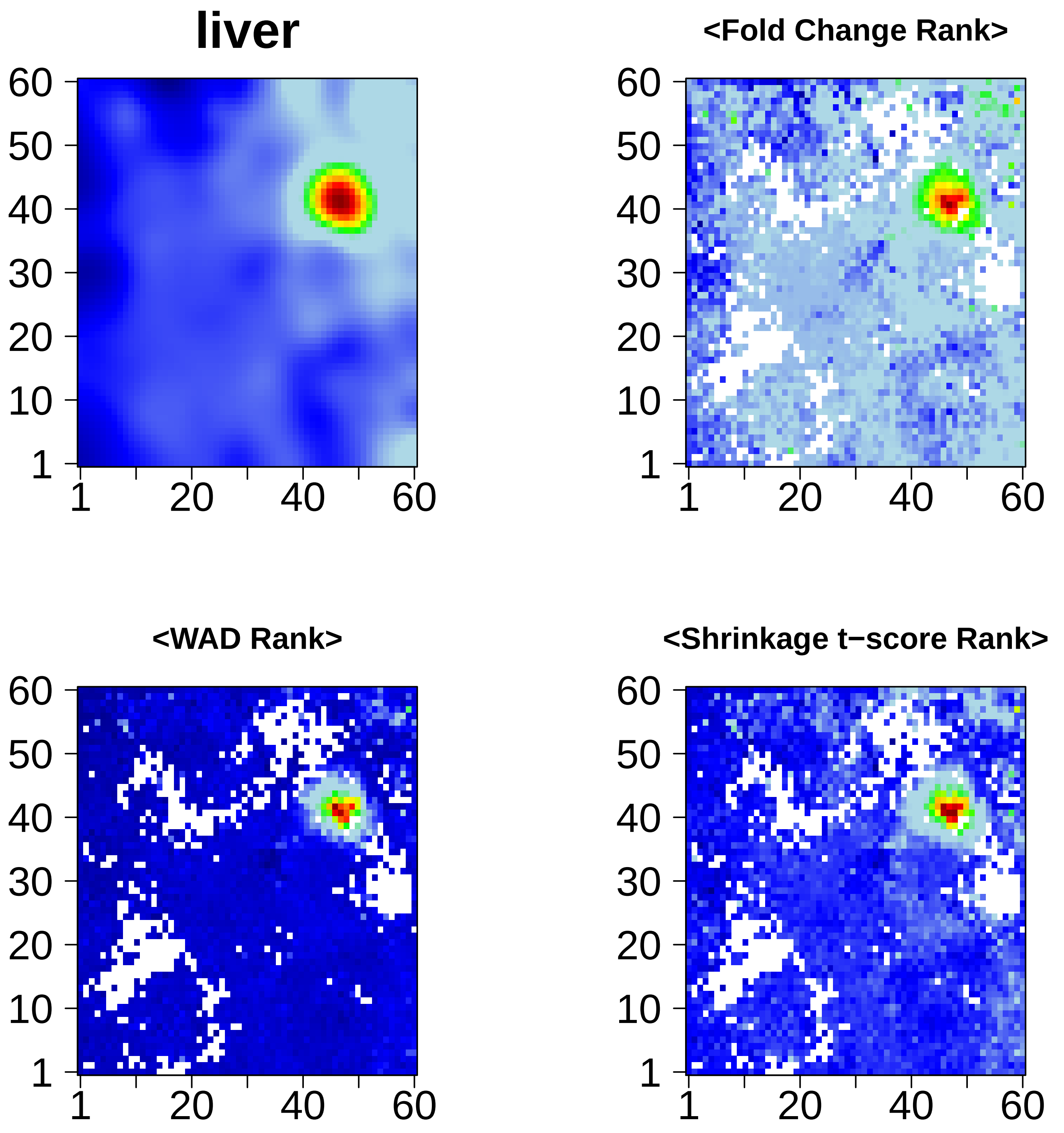

liver
<Fold Change Rank>
<WAD Rank>
<Shrinkage t−score Rank>
60
50
40
30
20
10
1
1
20
40
60

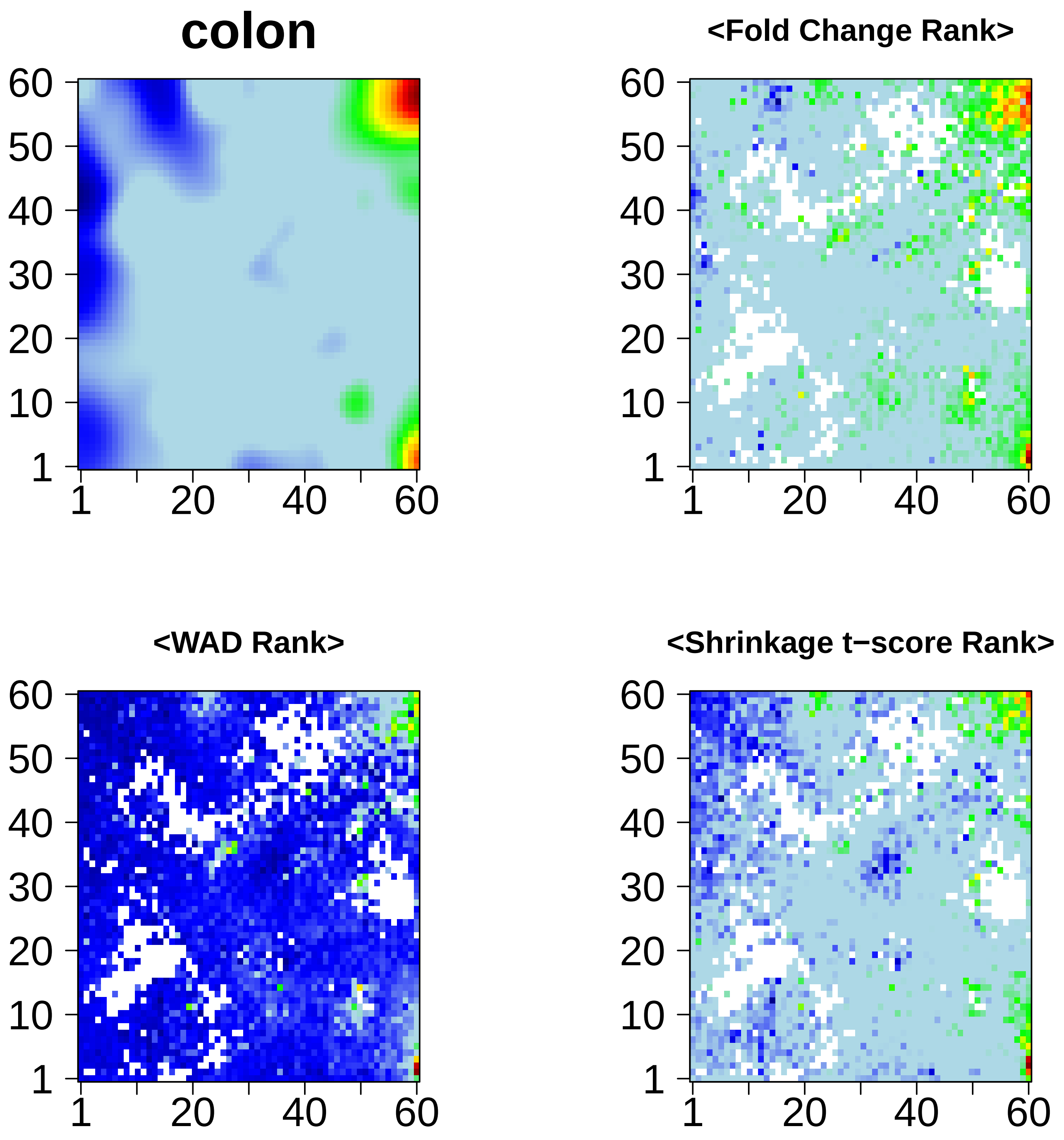

colon
<Fold Change Rank>
<WAD Rank>
<Shrinkage t−score Rank>
1
10
20
30
40
50
60

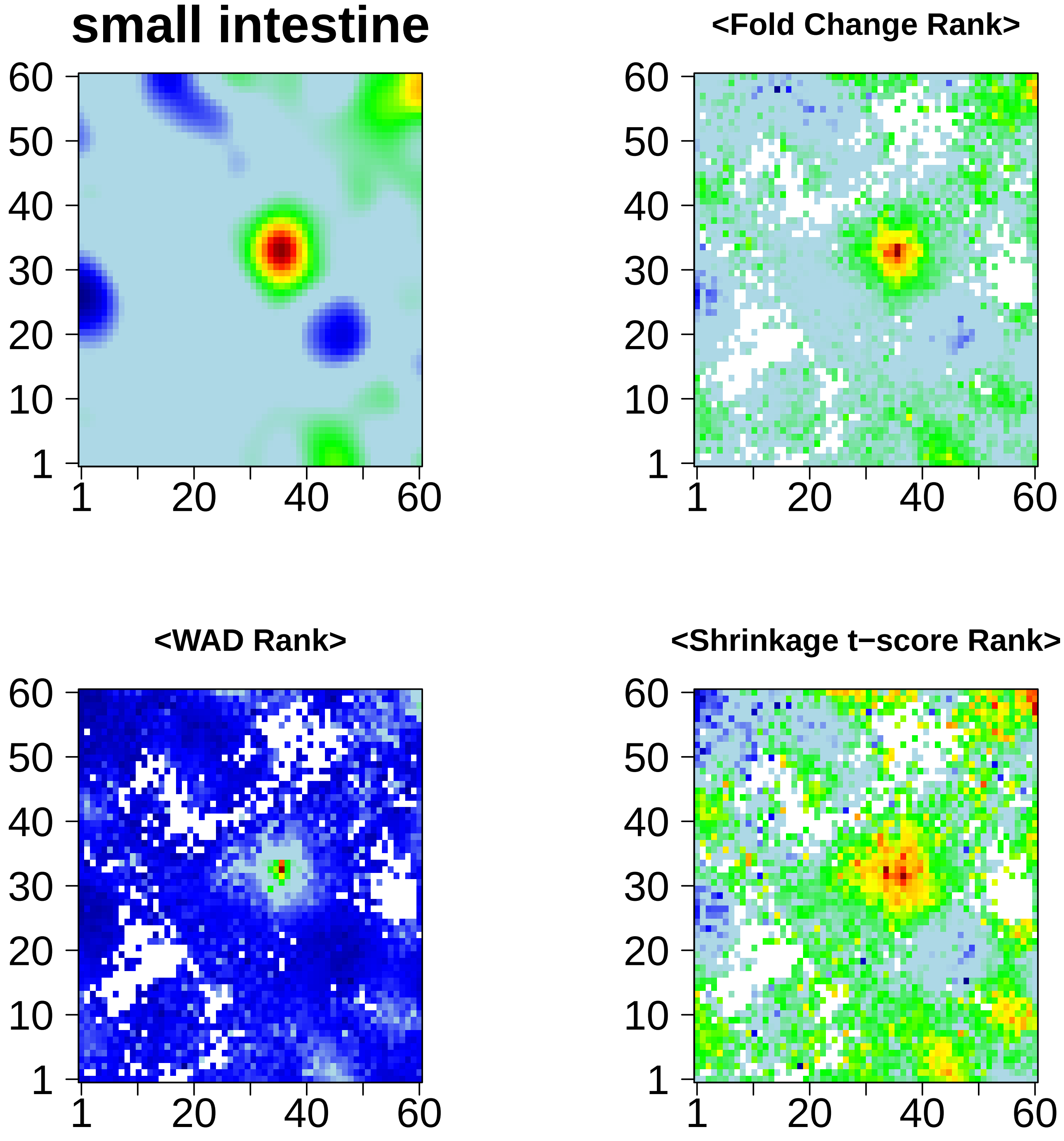
small intestine
<Fold Change Rank>
<WAD Rank>
<Shrinkage t−score Rank>
1
10
20
30
40
50
60

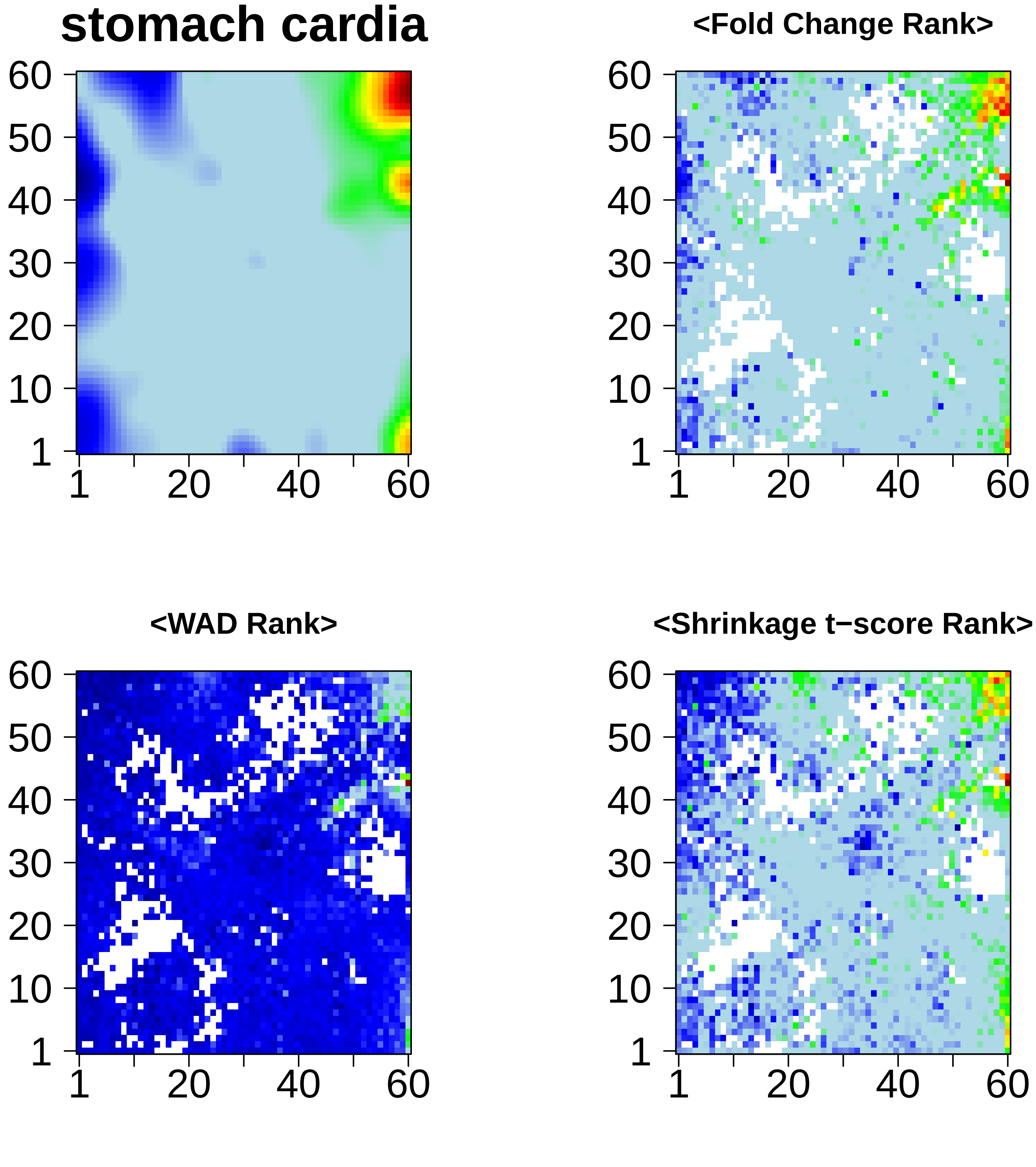
stomach cardia
<Fold Change Rank>
<WAD Rank>
<Shrinkage t−score Rank>
1
10
20
30
40
50
60
1
20
40
60

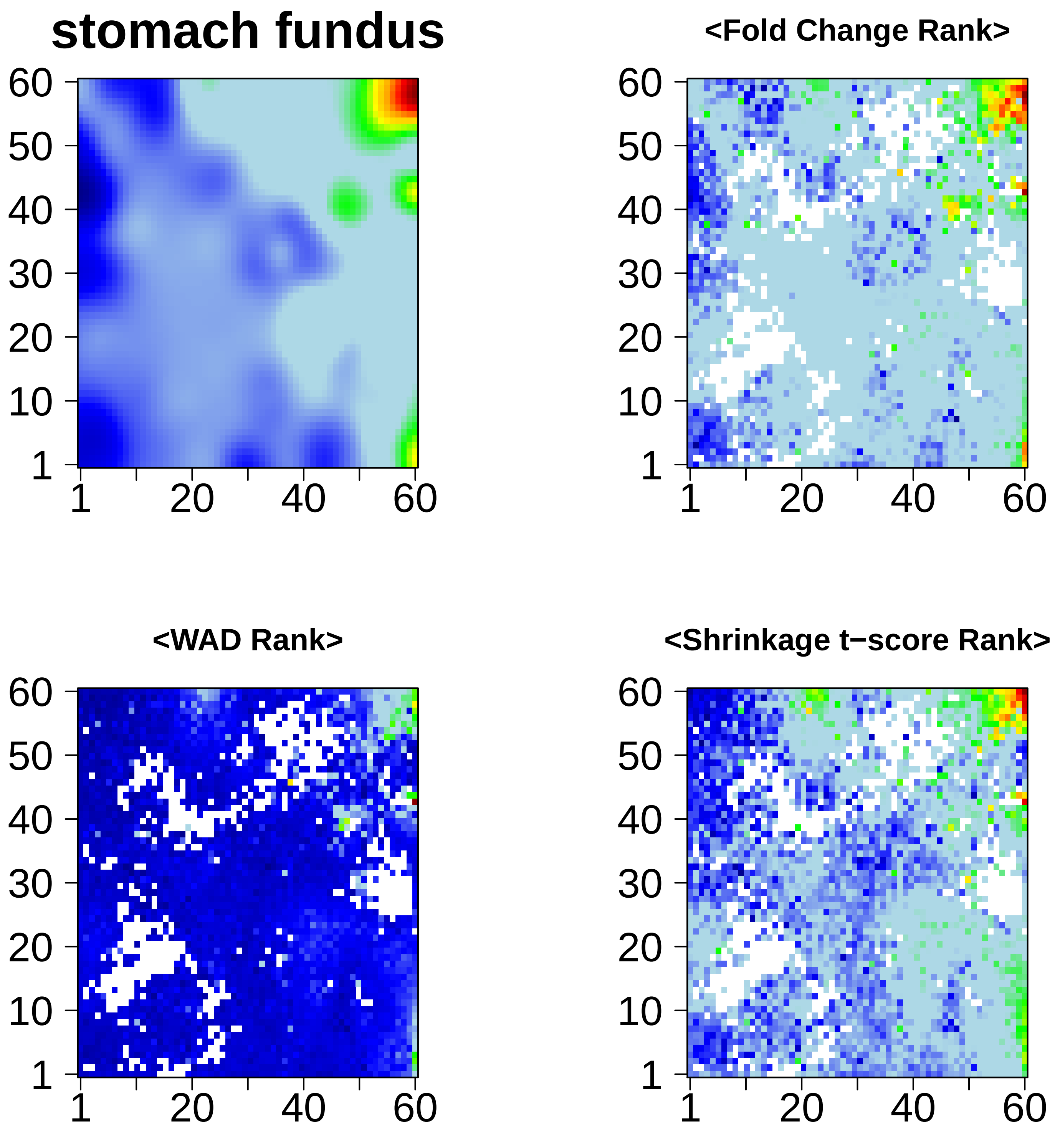

stomach fundus
<Fold Change Rank>
<WAD Rank>
<Shrinkage t−score Rank>
60
50
40
30
20
10
1
1
20
40
60

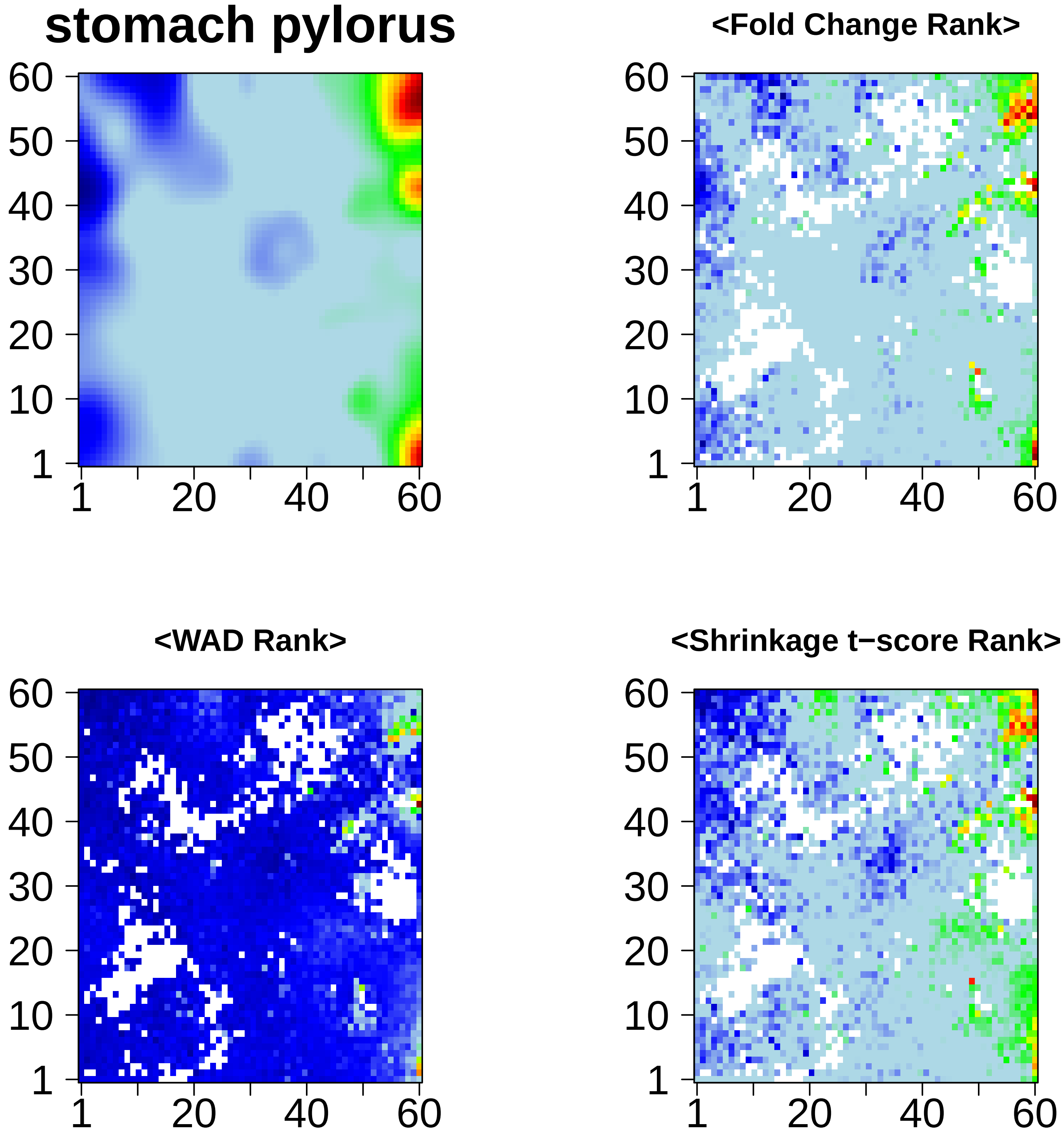
stomach pylorus
<Fold Change Rank>
<WAD Rank>
<Shrinkage t−score Rank>

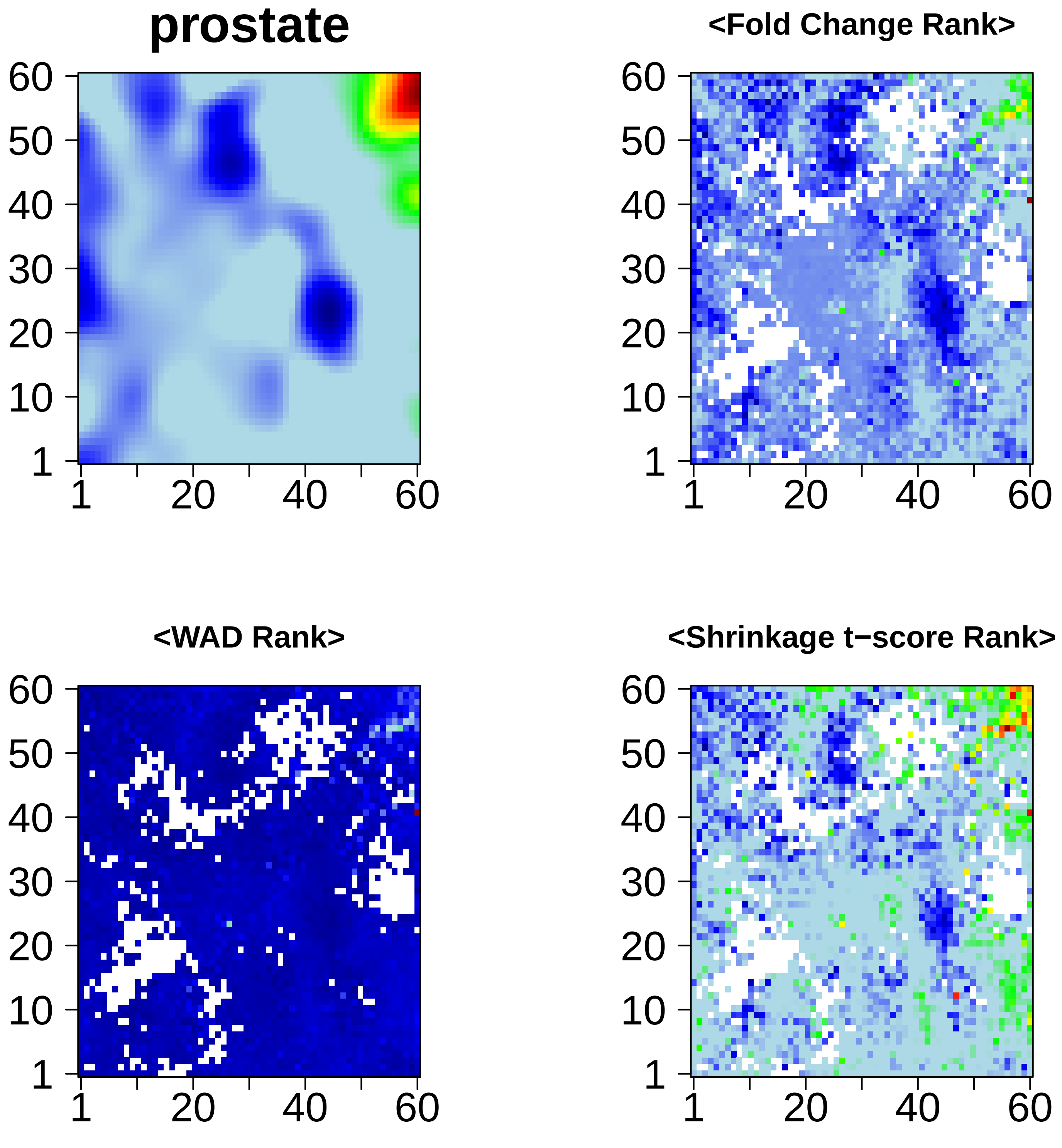
prostate
<Fold Change Rank>
<WAD Rank>
<Shrinkage t−score Rank>
1
10
20
30
40
50
60

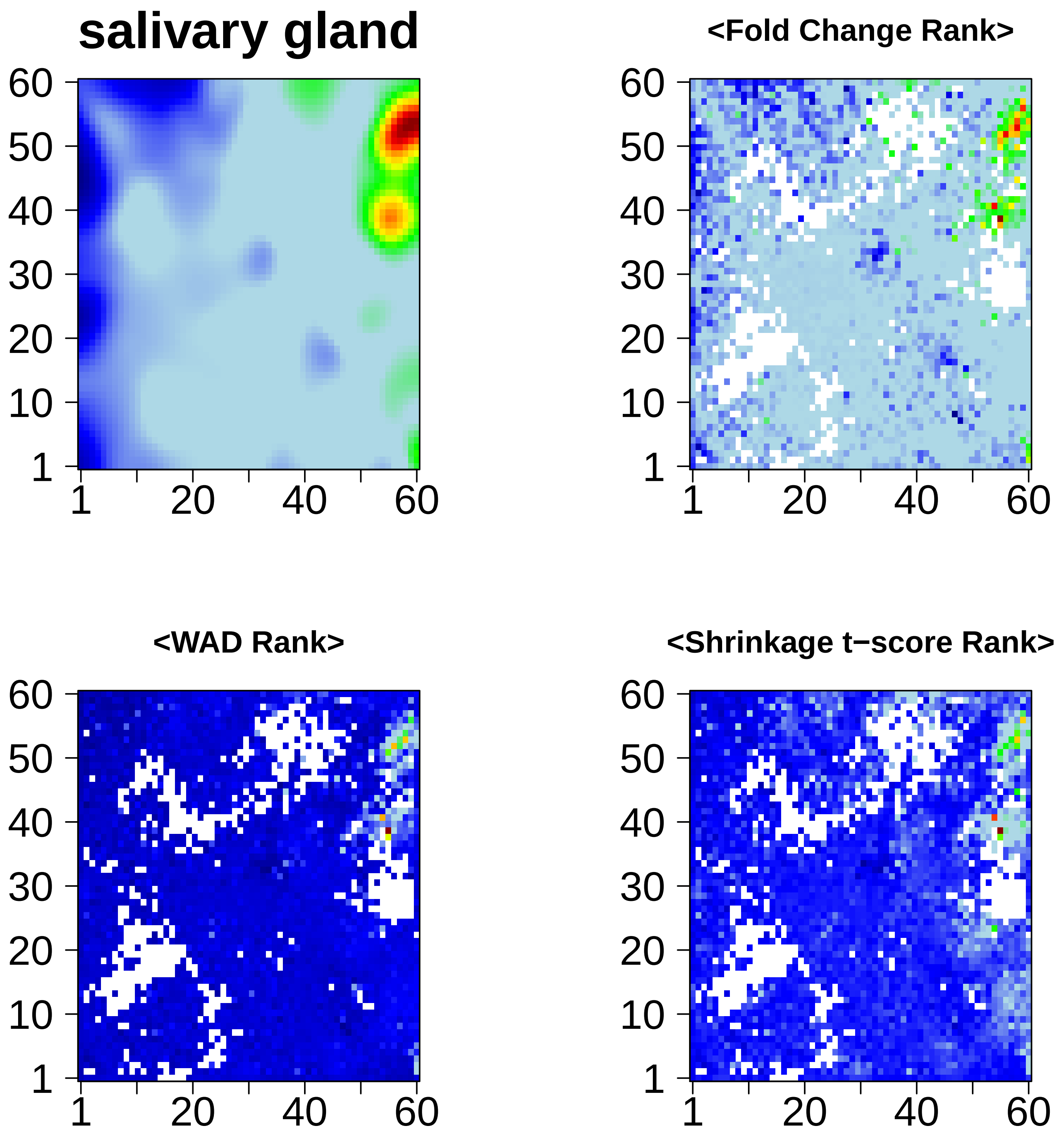

salivary gland
<Fold Change Rank>
<WAD Rank>
<Shrinkage t−score Rank>
60
50
40
30
20
10
1
1
20
40
60

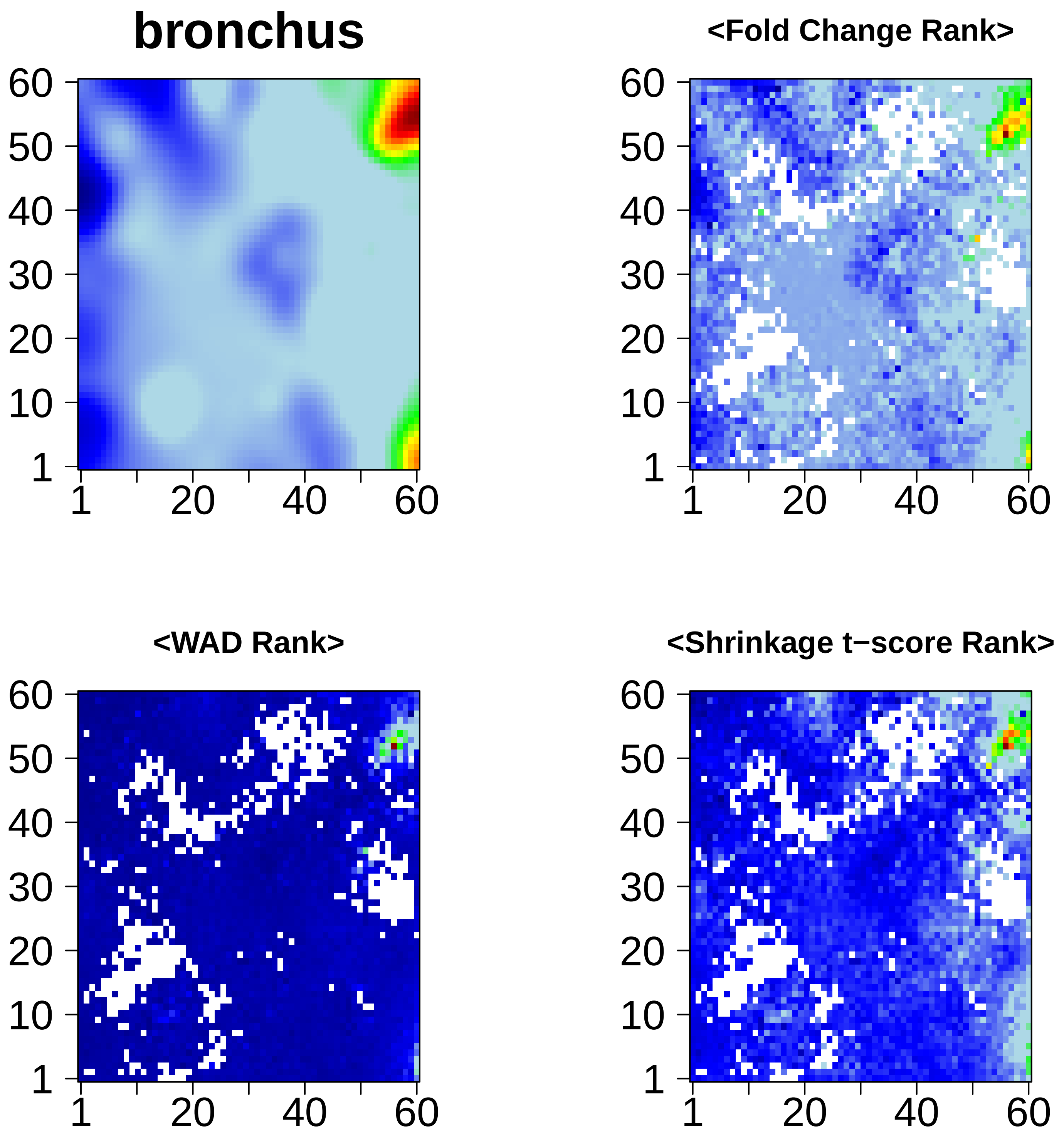

bronchus
<Fold Change Rank>
<WAD Rank>
<Shrinkage t−score Rank>
60
50
40
30
20
10
1
1
20
40
60

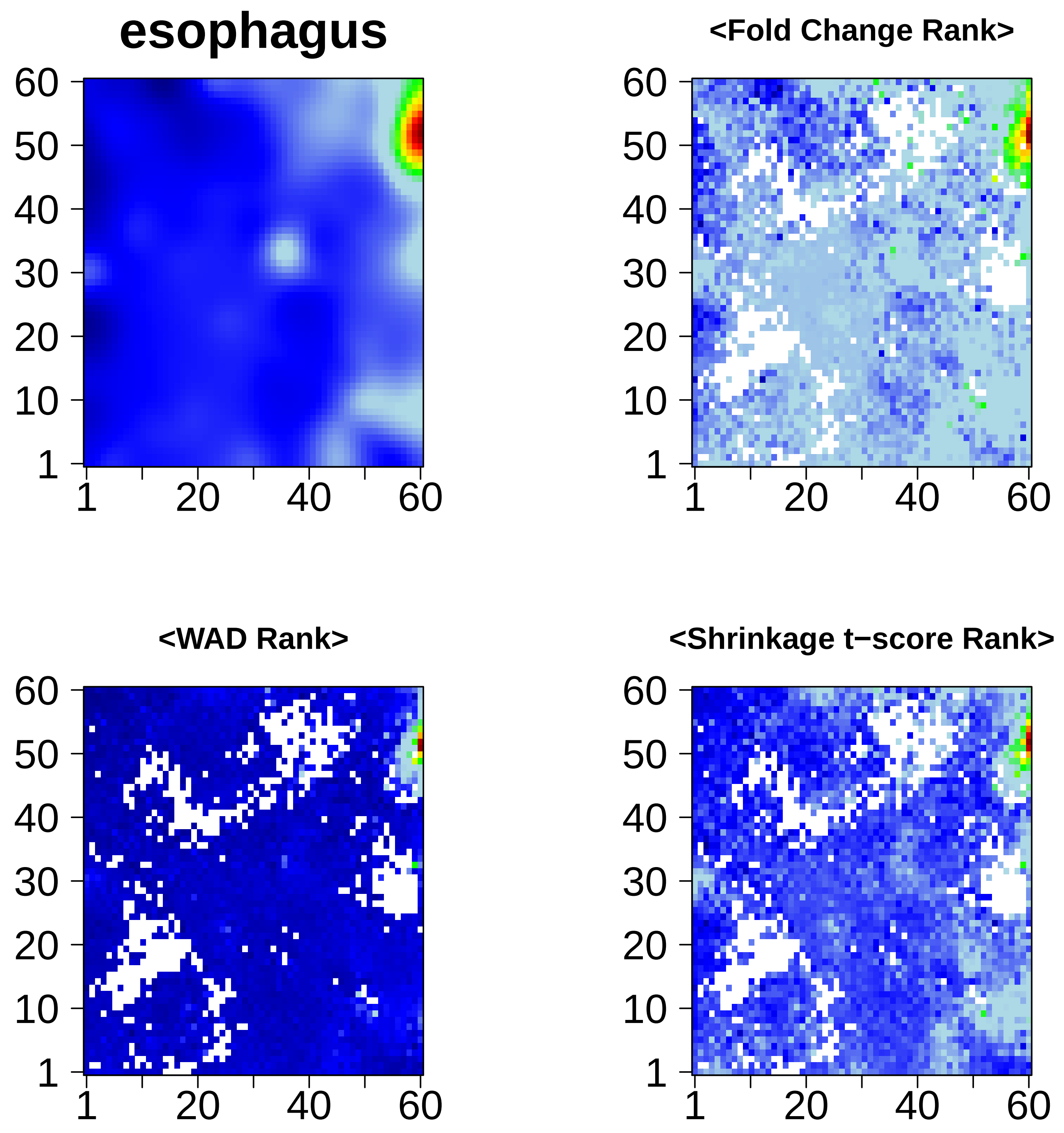
esophagus
<Fold Change Rank>
<WAD Rank>
<Shrinkage t−score Rank>

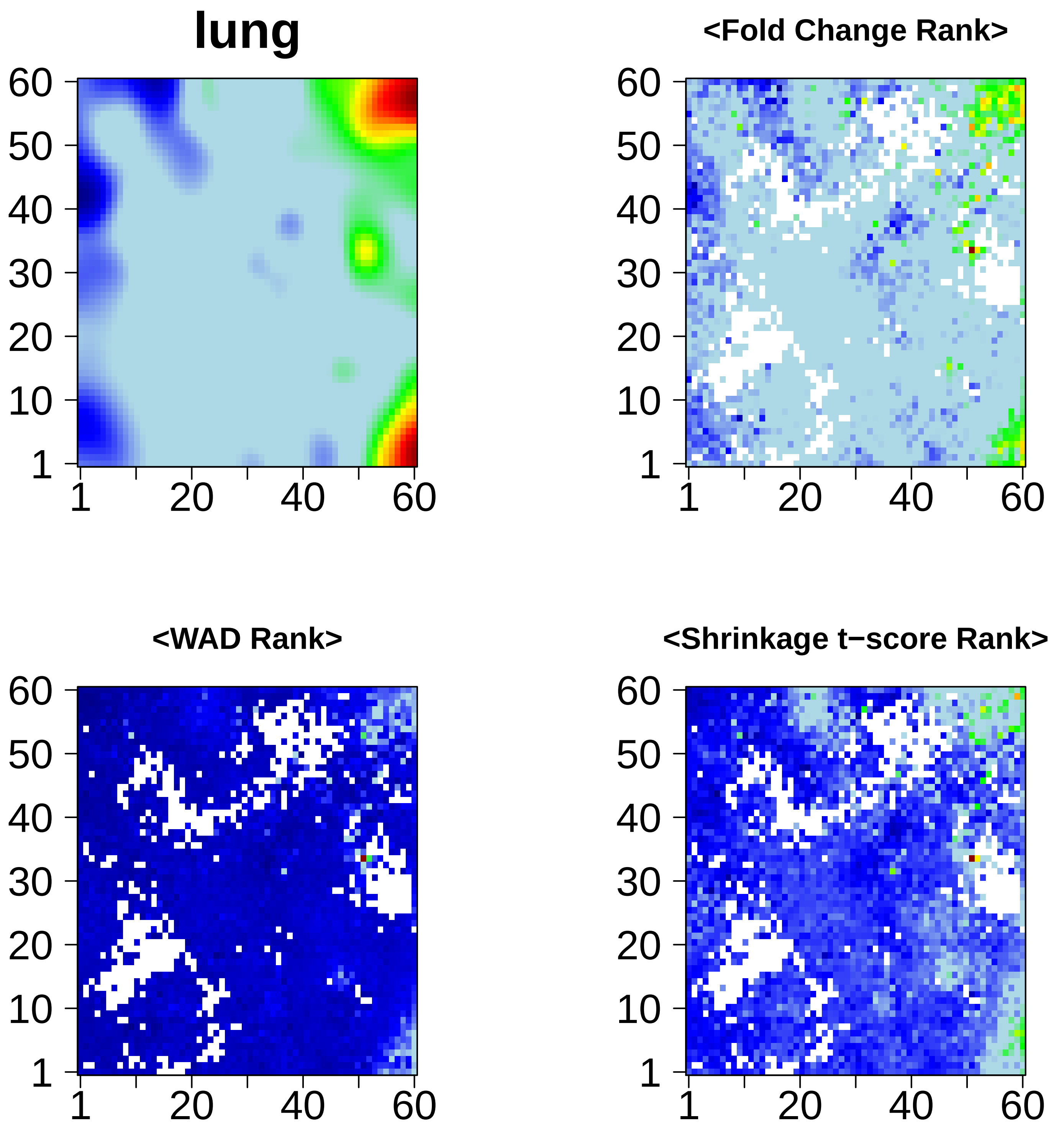
lung
<Fold Change Rank>
<WAD Rank>
<Shrinkage t−score Rank>
60
50
40
30
20
10
1
1
20
40
60

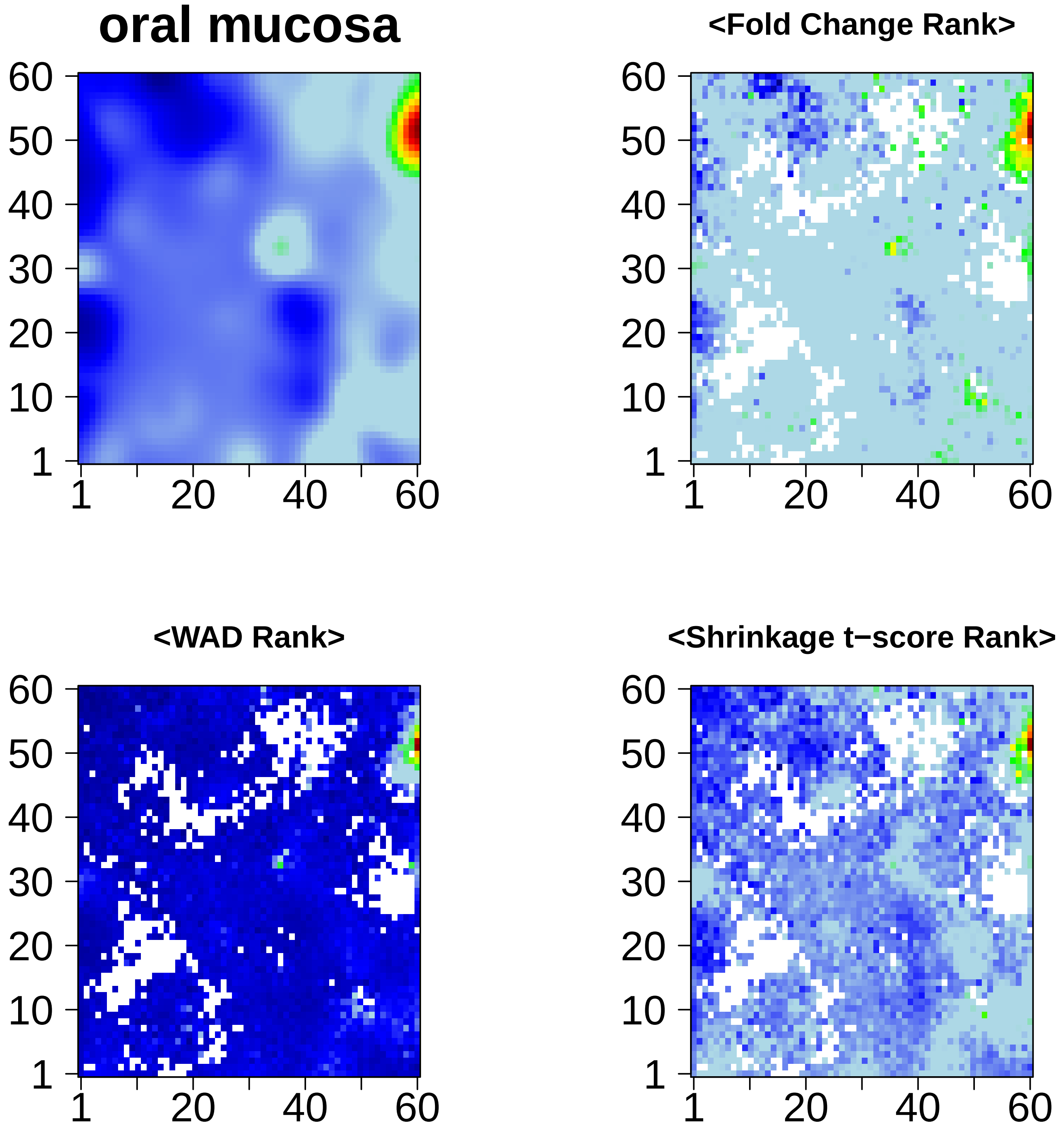
oral mucosa
<Fold Change Rank>
<WAD Rank>
<Shrinkage t−score Rank>
1
10
20
30
40
50
60

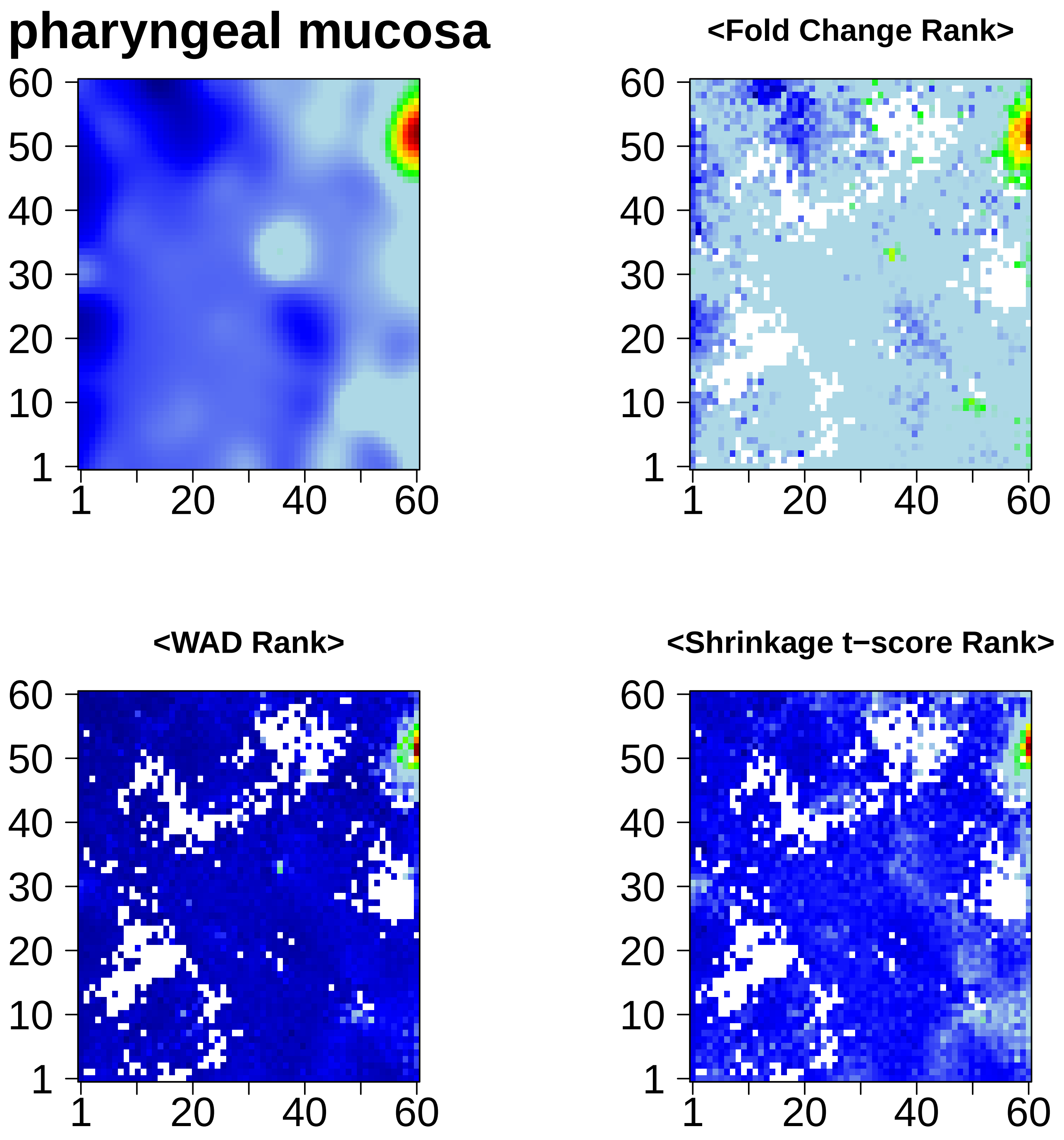

pharyngeal mucosa
<Fold Change Rank>
<WAD Rank>
<Shrinkage t−score Rank>
60
50
40
30
20
10
1
1
20
40
60

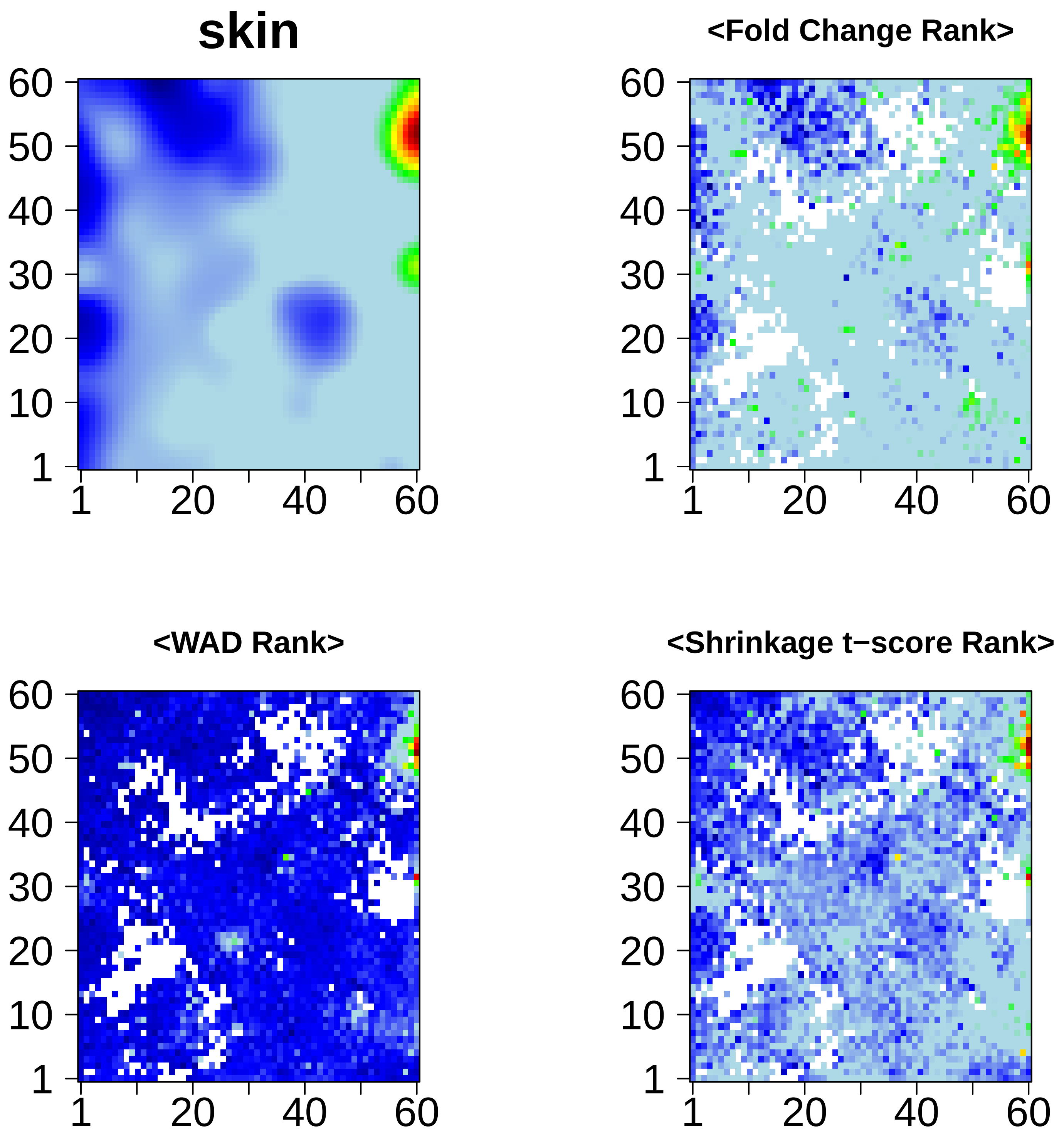
skin
<Fold Change Rank>
<WAD Rank>
<Shrinkage t−score Rank>
1
10
20
30
40
50
60
1
20
40
60

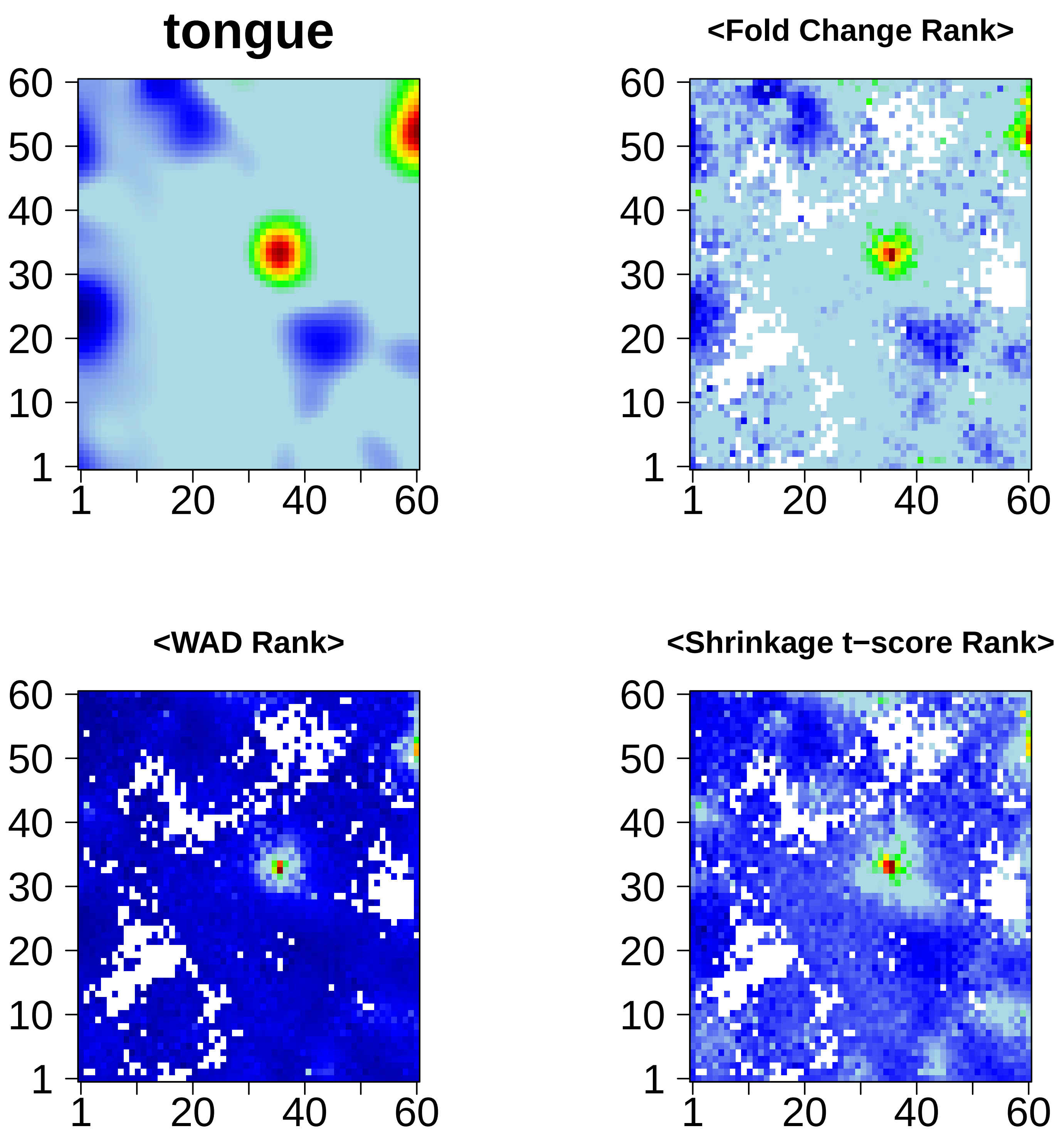

tongue
<Fold Change Rank>
<WAD Rank>
<Shrinkage t−score Rank>
60
50
40
30
20
10
1
1
20
40
60

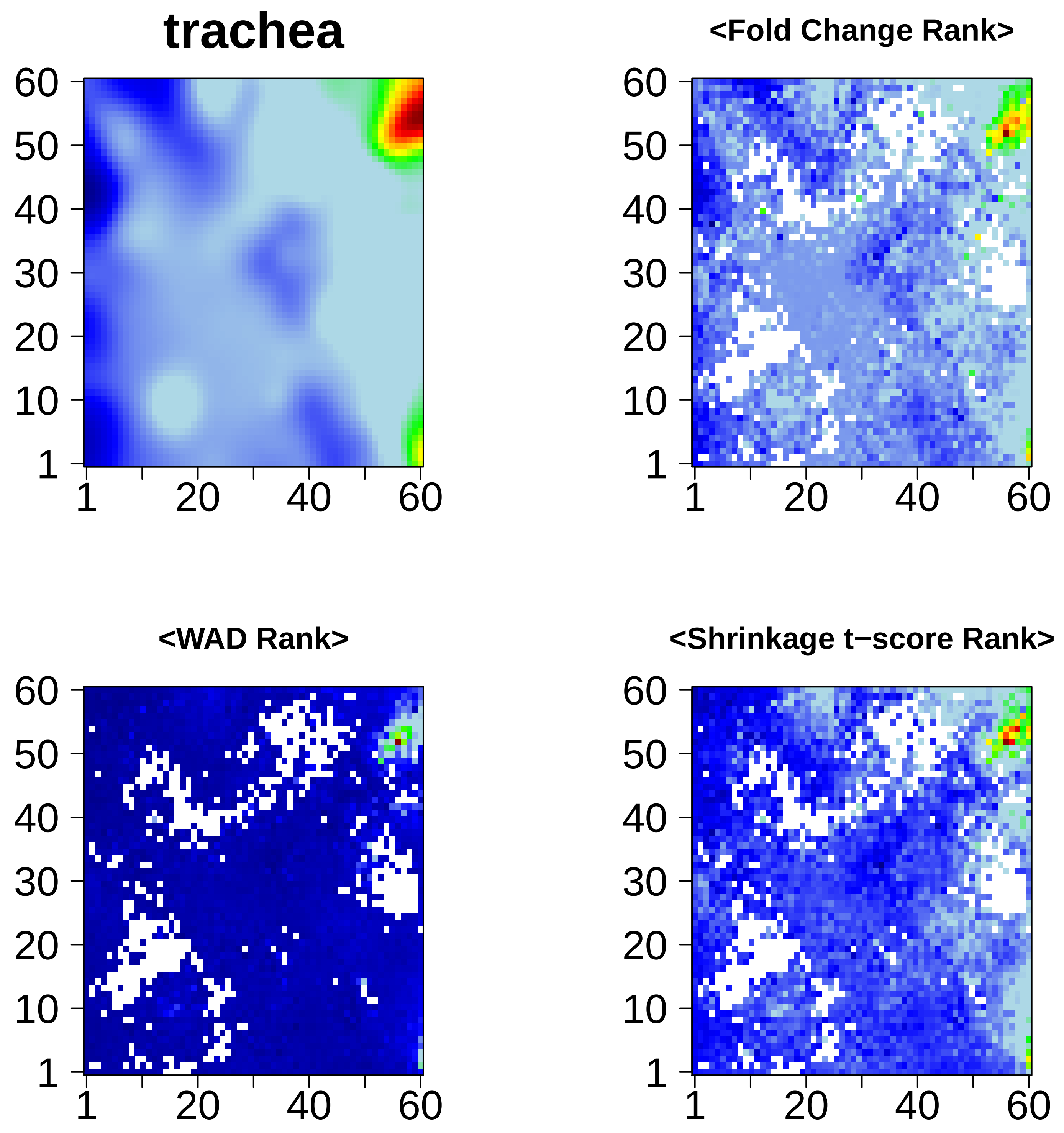
trachea
<Fold Change Rank>
<WAD Rank>
<Shrinkage t−score Rank>
1
10
20
30
40
50
60
1
20
40
60

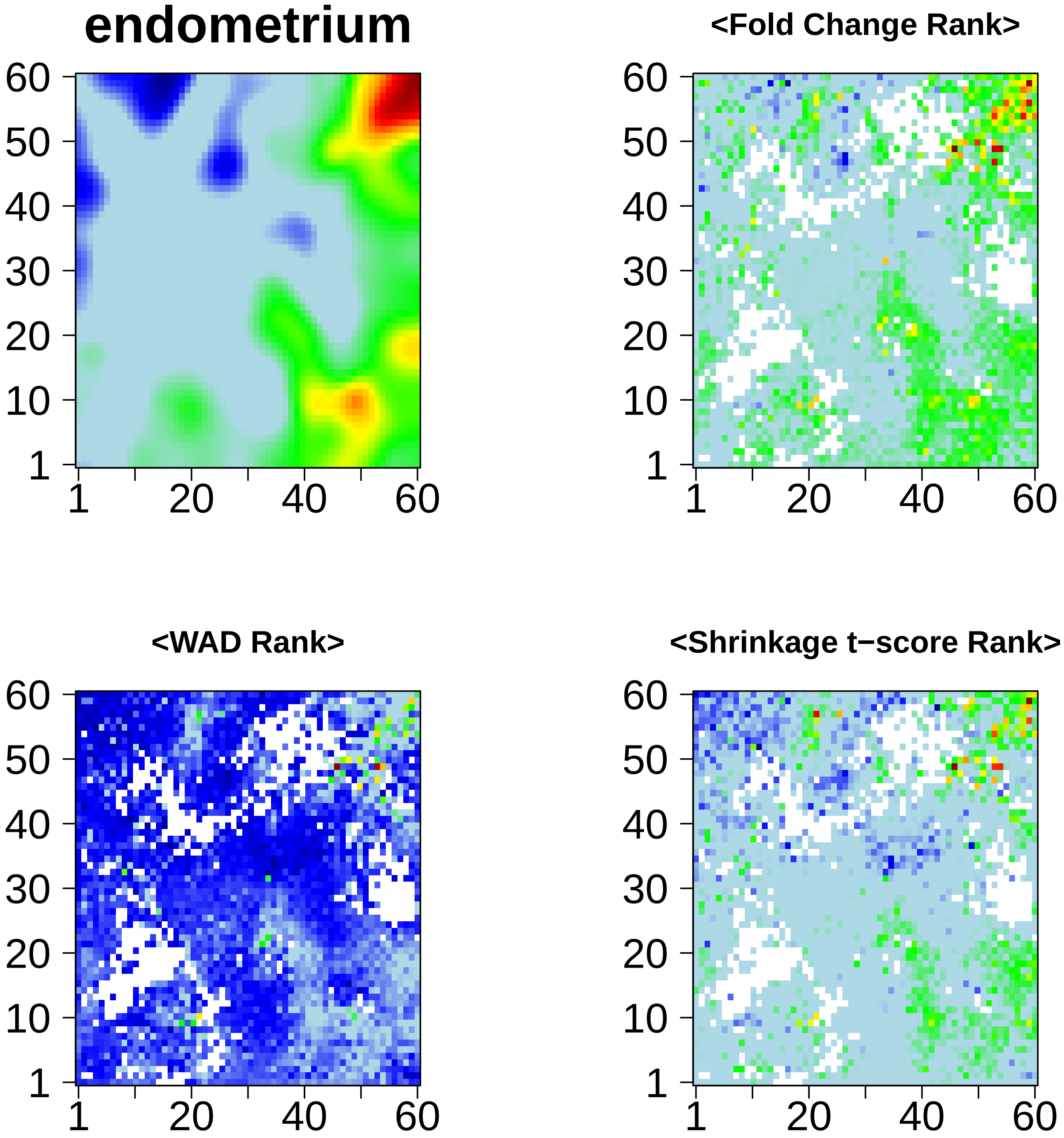
endometrium
<Fold Change Rank>
<WAD Rank>
<Shrinkage t−score Rank>
60
50
40
30
20
10
1
1
20
40
60

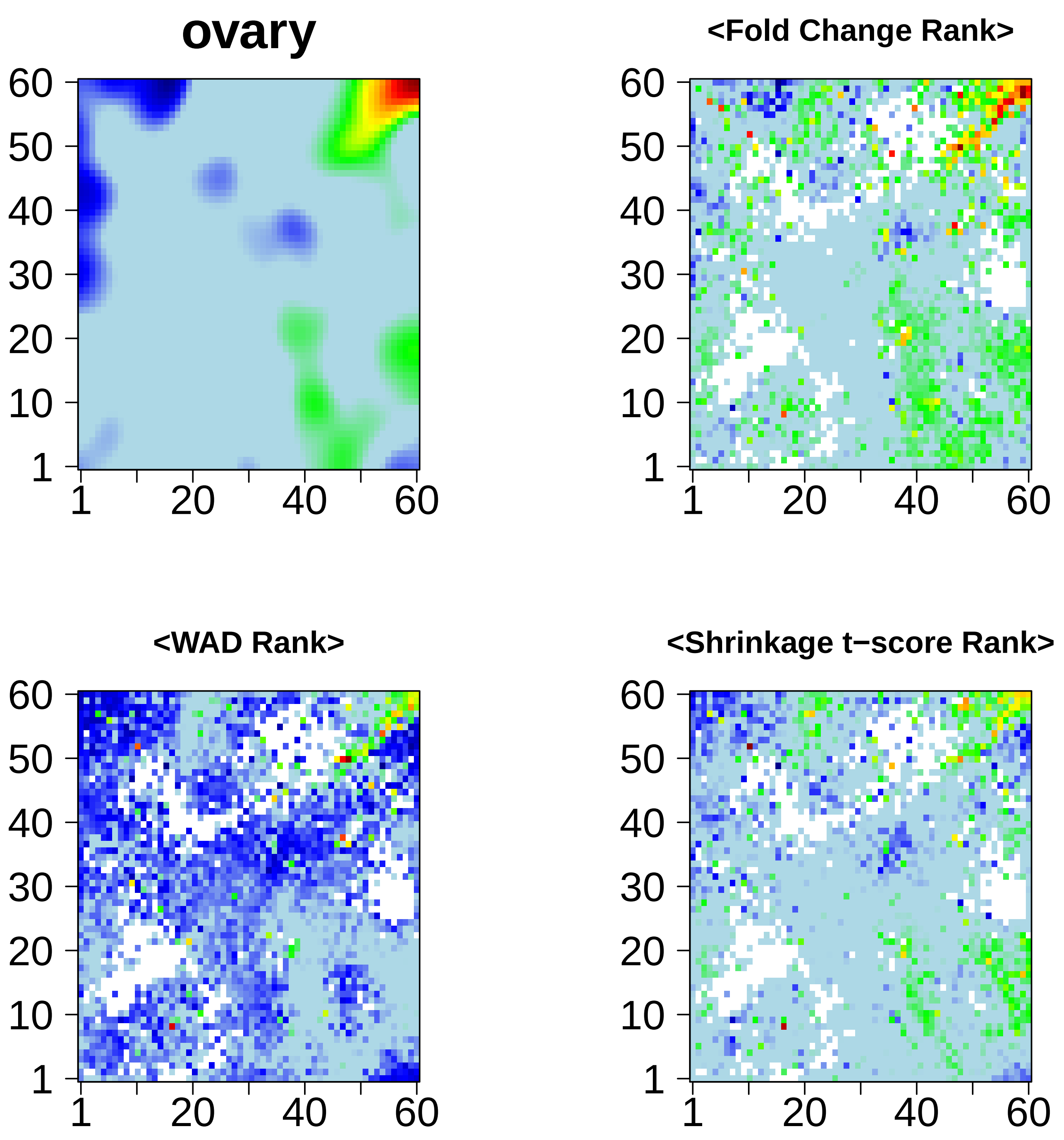
ovary
<Fold Change Rank>
<WAD Rank>
<Shrinkage t−score Rank>
1
10
20
30
40
50
60
1
20
40
60

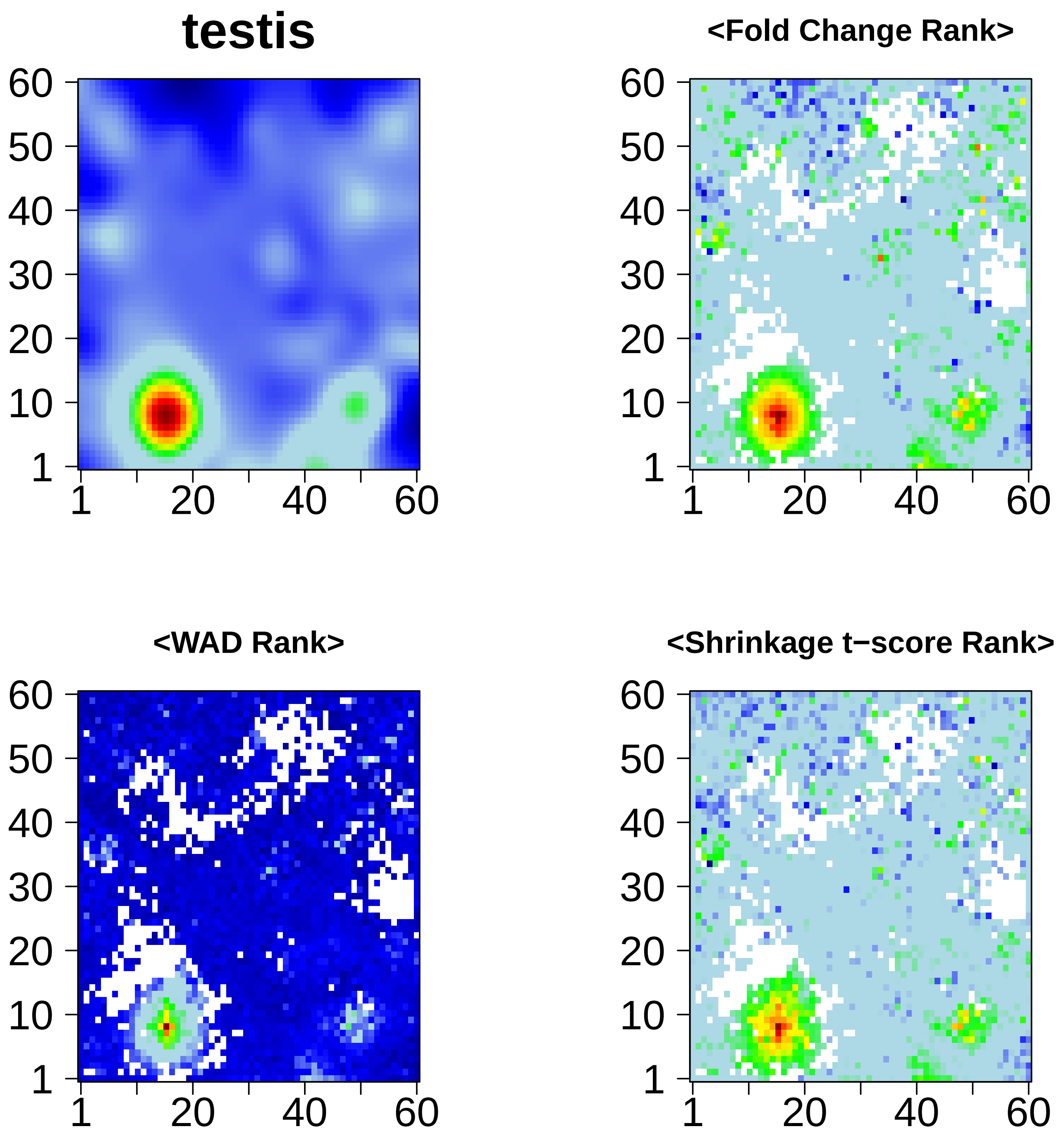
testis
<Fold Change Rank>
<WAD Rank>
<Shrinkage t−score Rank>
1
10
20
30
40
50
60

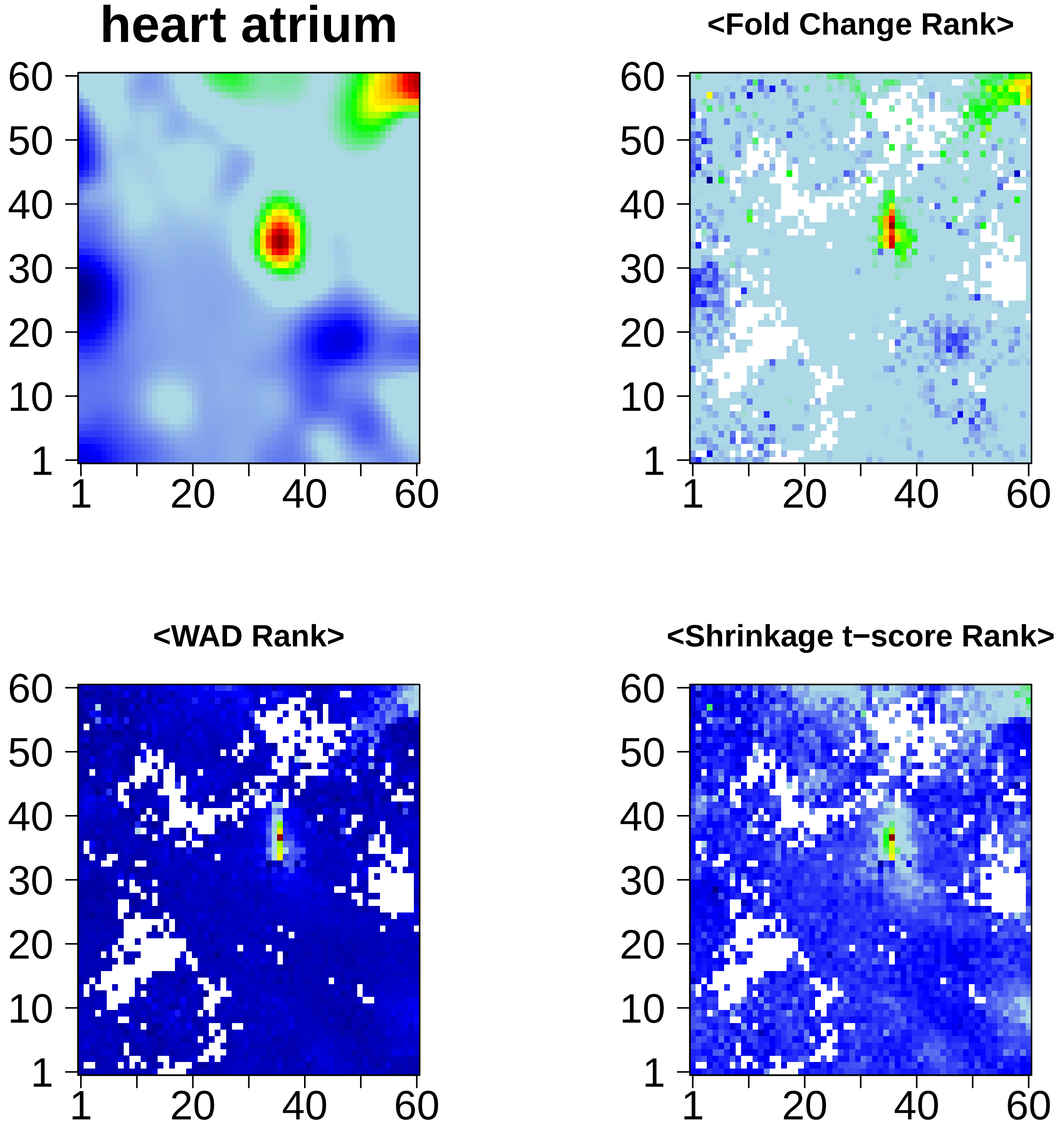
heart atrium
<Fold Change Rank>
<WAD Rank>
<Shrinkage t−score Rank>
60
50
40
30
20
10
1
1
20
40
60

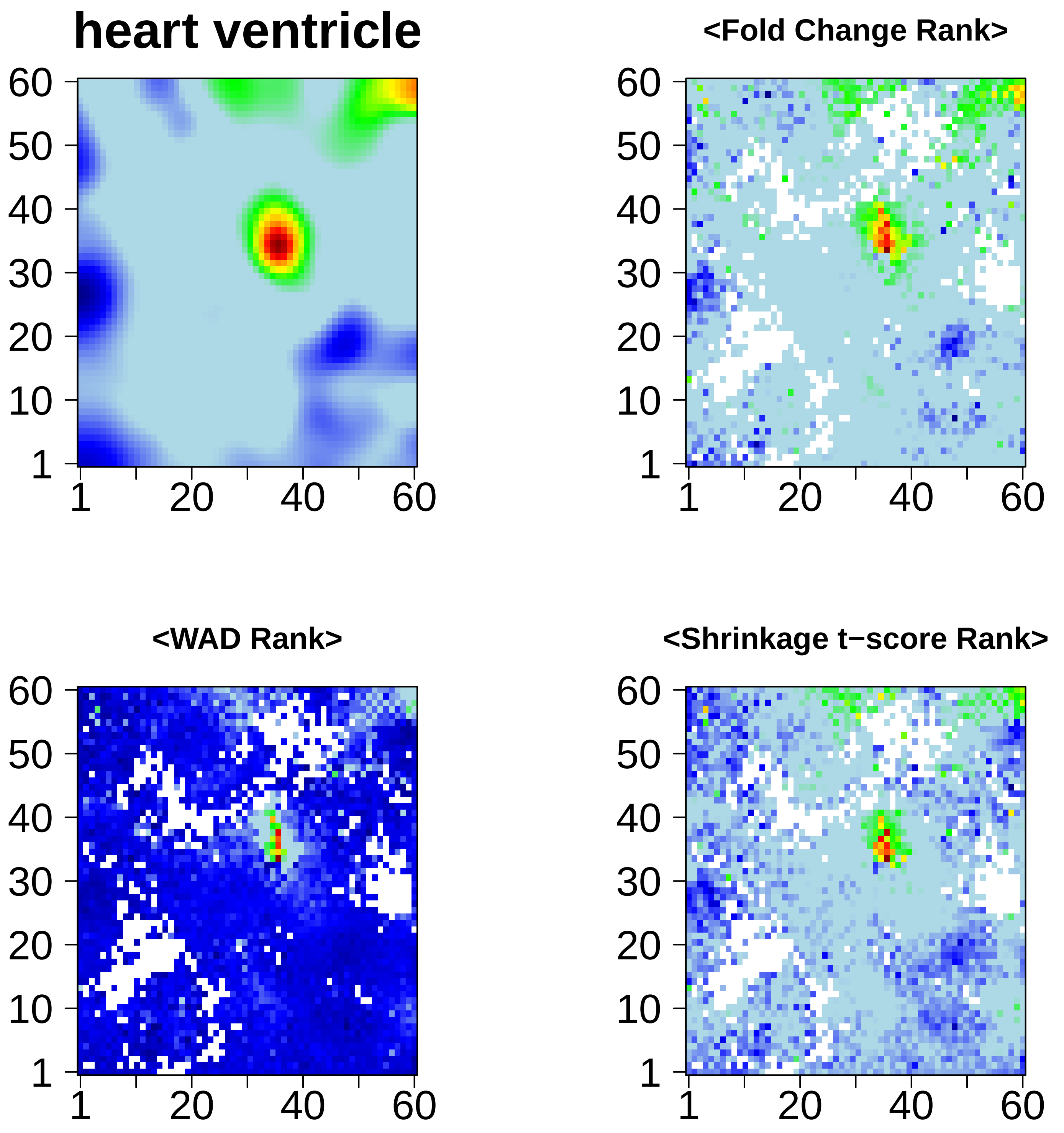
heart ventricle
<Fold Change Rank>
<WAD Rank>
<Shrinkage t−score Rank>
1
10
20
30
40
50
60

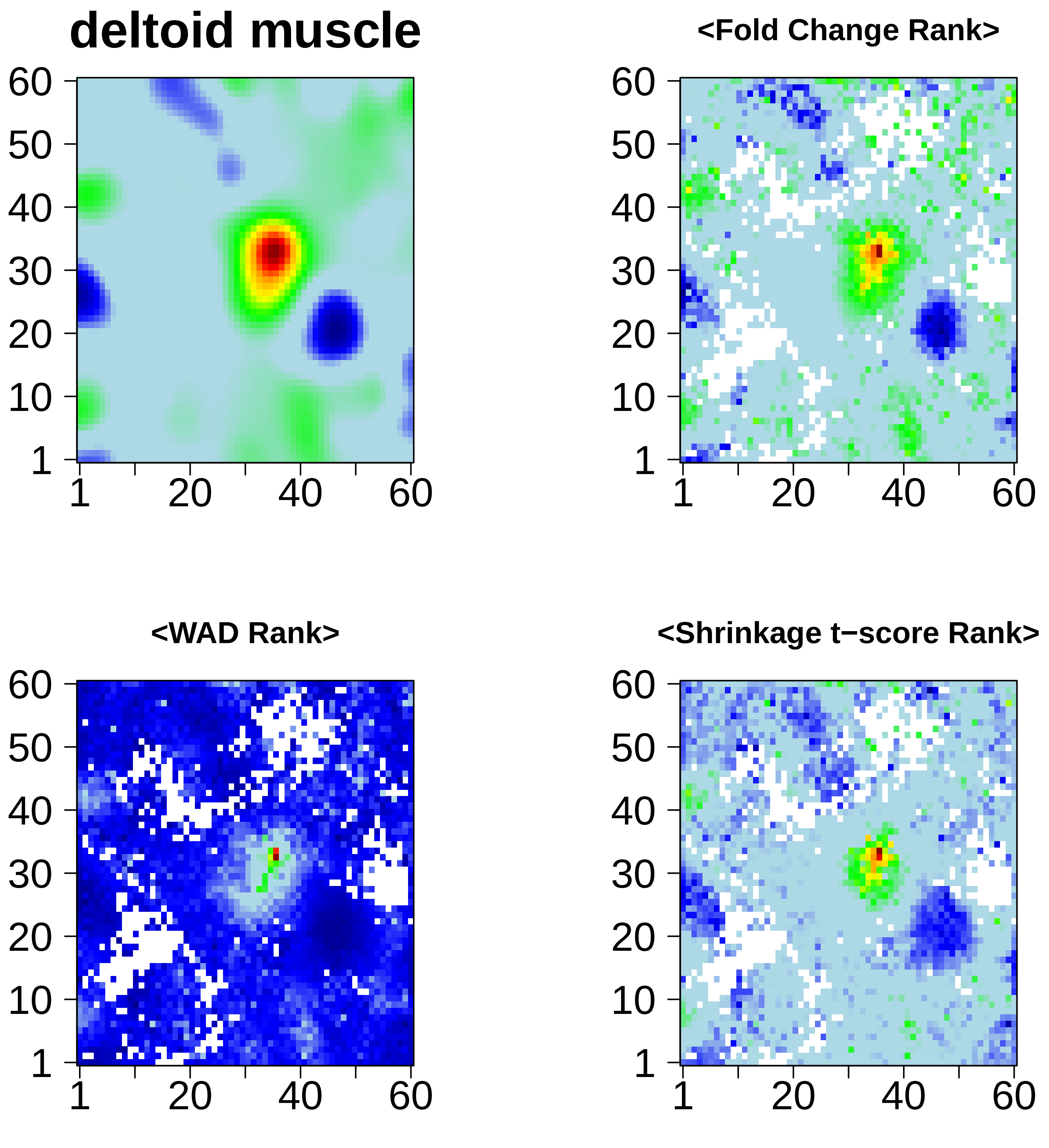
deltoid muscle
<Fold Change Rank>
<WAD Rank>
<Shrinkage t−score Rank>
1 10 20 30 40 50 60
1 20 40 60

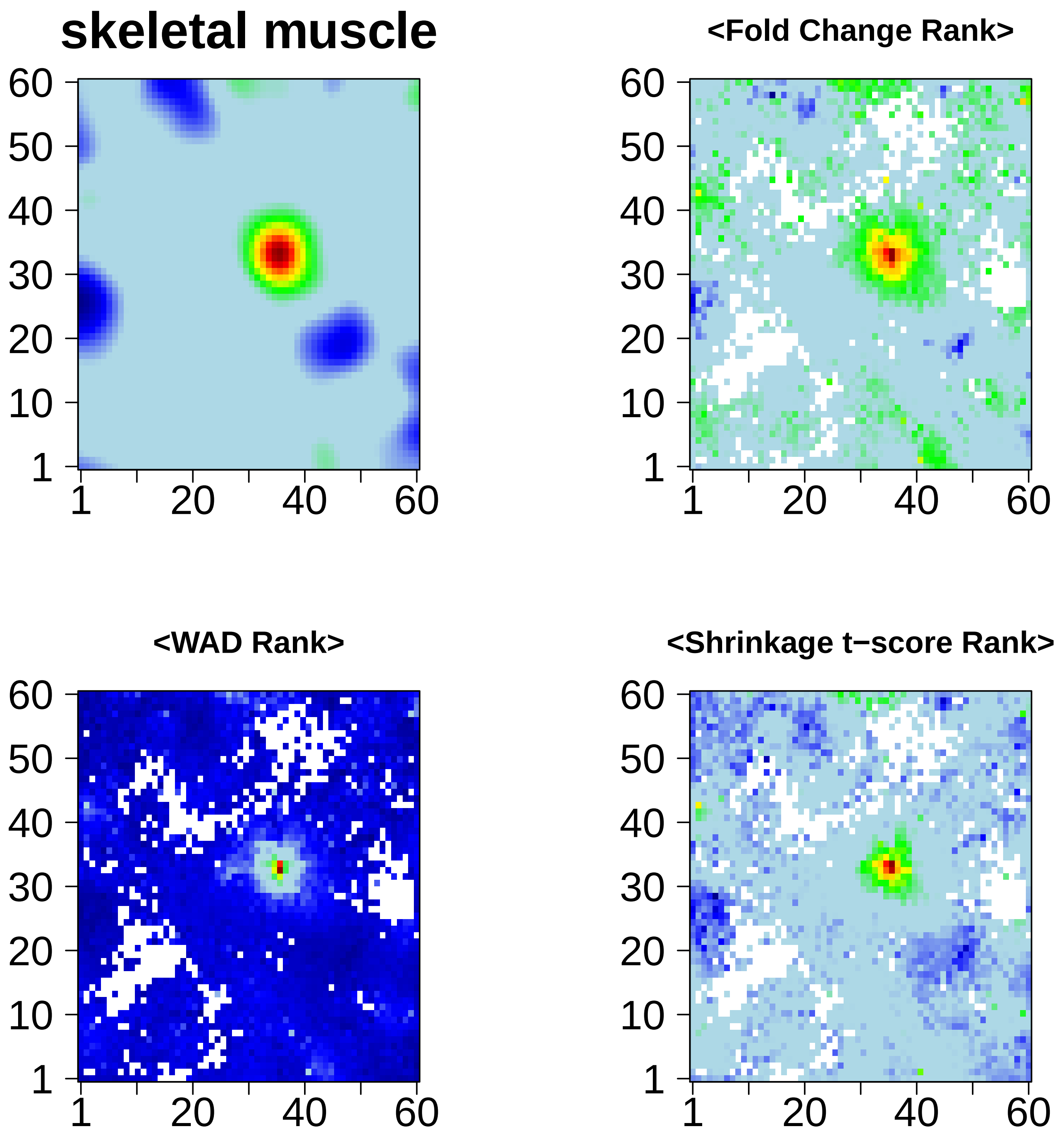
skeletal muscle
<Fold Change Rank>
<WAD Rank>
<Shrinkage t−score Rank>
1
10
20
30
40
50
60

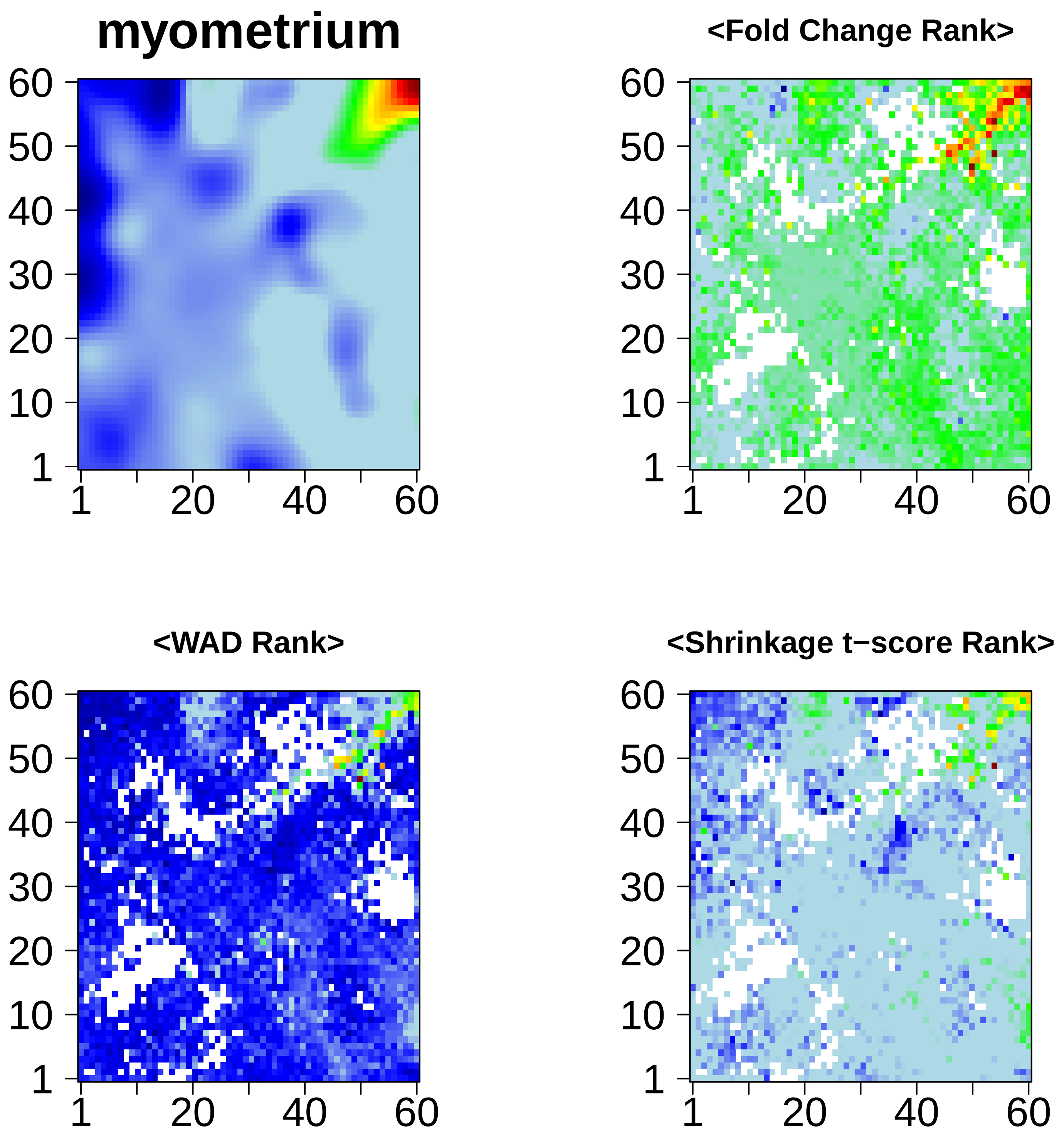
myometrium
<Fold Change Rank>
<WAD Rank>
<Shrinkage t−score Rank>
60
50
40
30
20
10
1
1
20
40
60

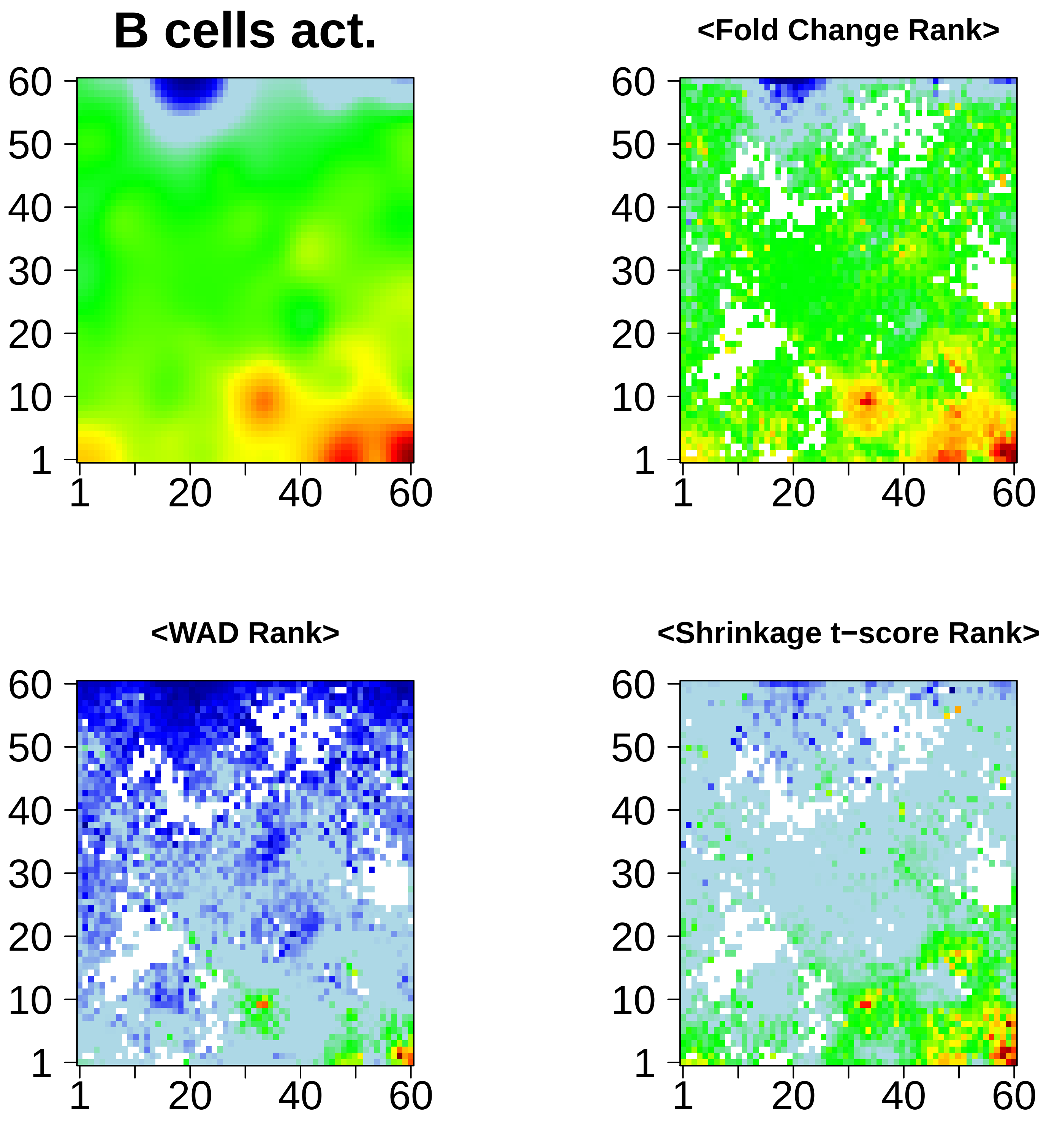
B cells act.
<Fold Change Rank>
<WAD Rank>
<Shrinkage t−score Rank>
60
50
40
30
20
10
1
1
20
40
60

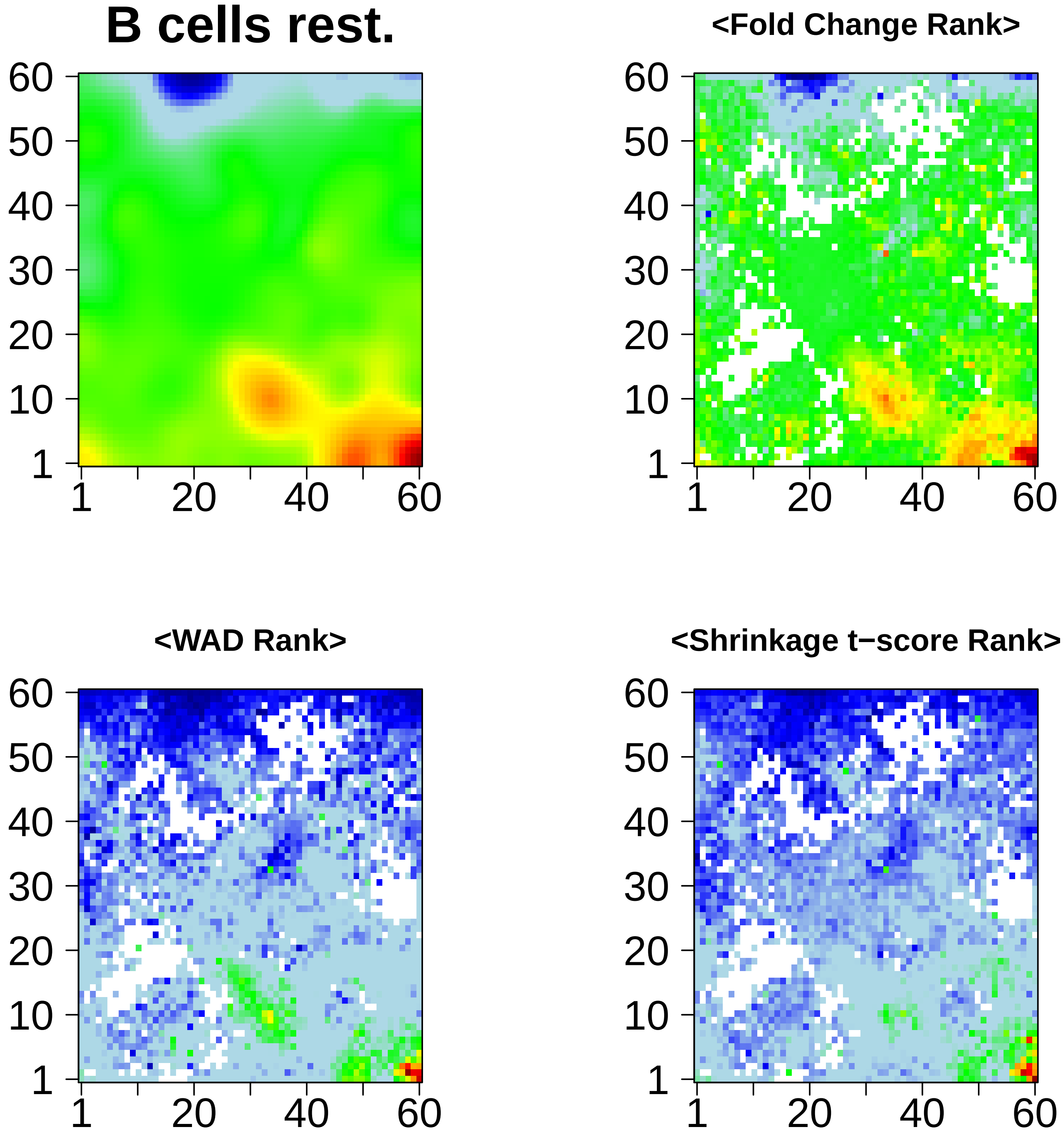
B cells rest.
<Fold Change Rank>
<WAD Rank>
<Shrinkage t−score Rank>
60
50
40
30
20
10
1
1
20
40
60

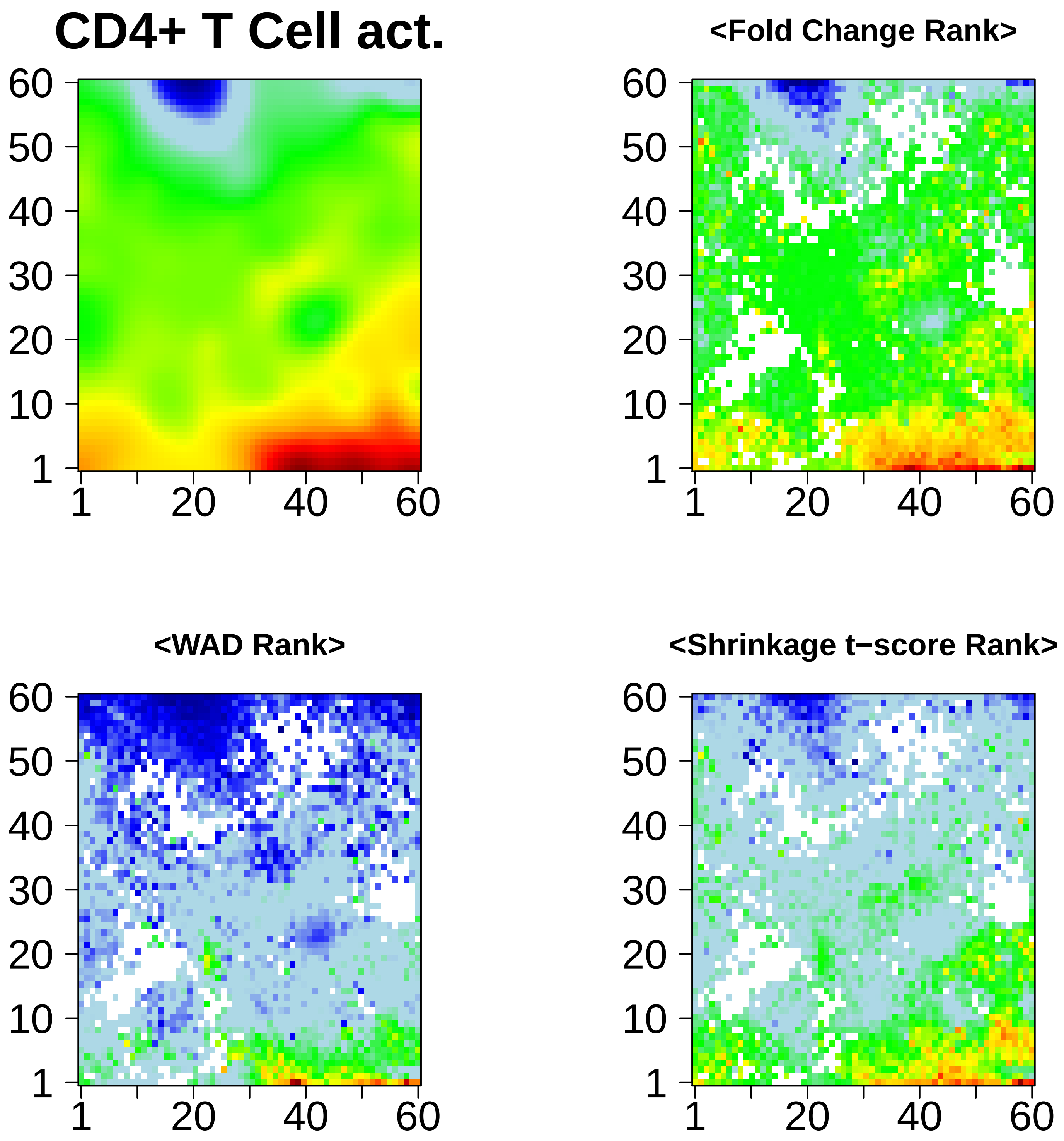
CD4+ T Cell act.
<Fold Change Rank>
<WAD Rank>
<Shrinkage t−score Rank>
60
50
40
30
20
10
1
1
20
40
60

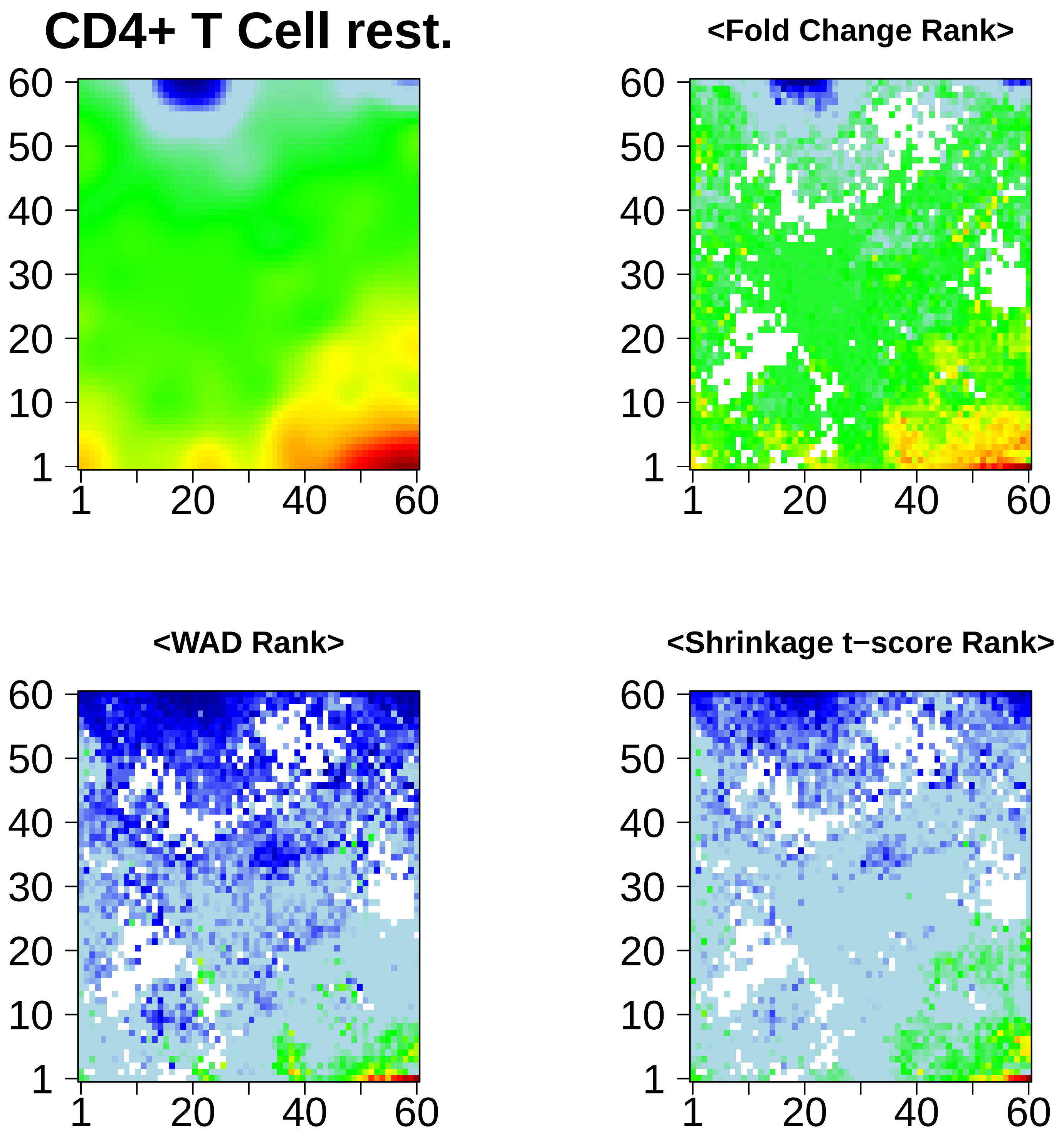
CD4+ T Cell rest.
<Fold Change Rank>
<WAD Rank>
<Shrinkage t−score Rank>
1
10
20
30
40
50
60

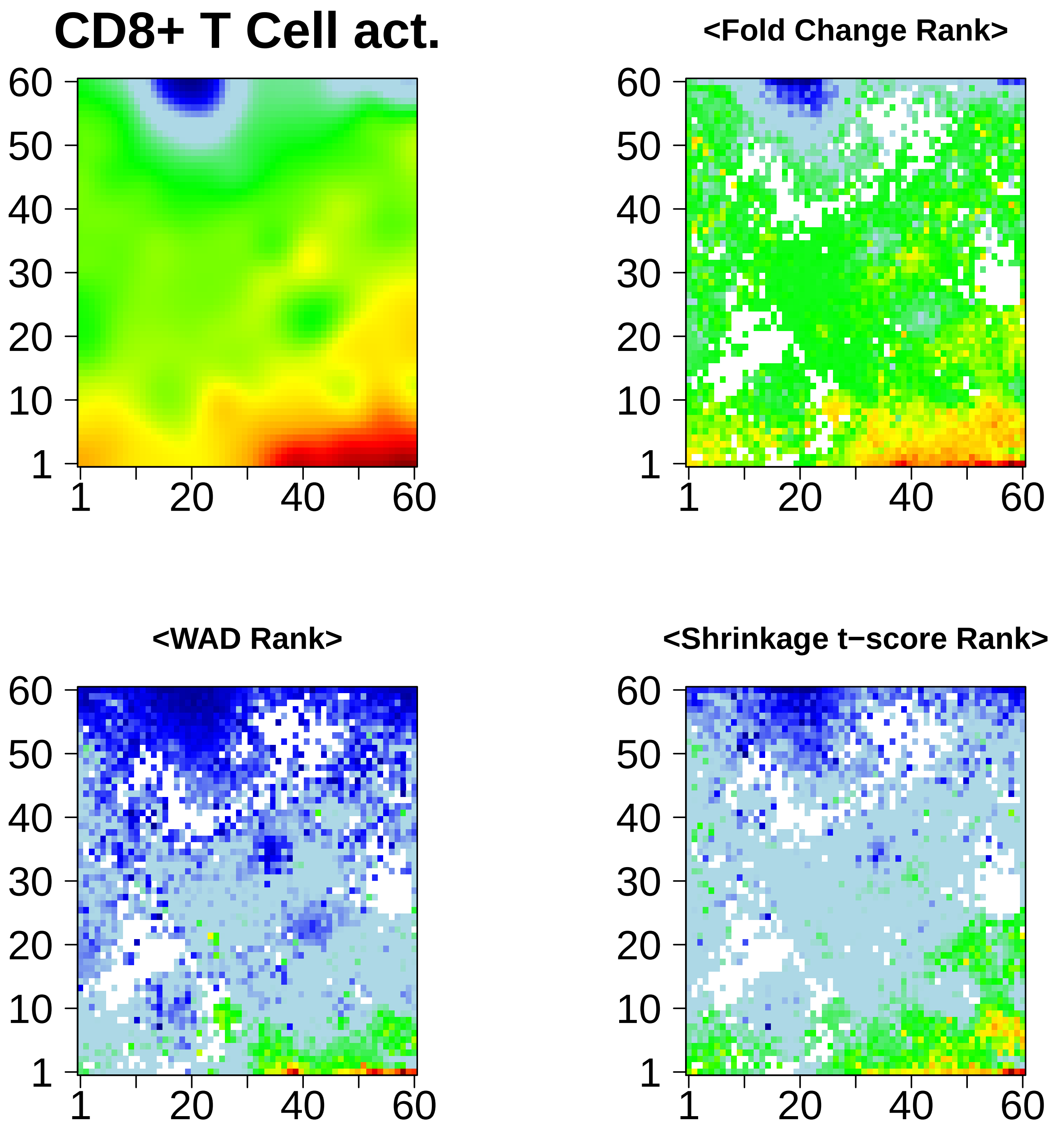
CD8+ T Cell act.
<Fold Change Rank>
<WAD Rank>
<Shrinkage t−score Rank>
60
50
40
30
20
10
1
1
20
40
60

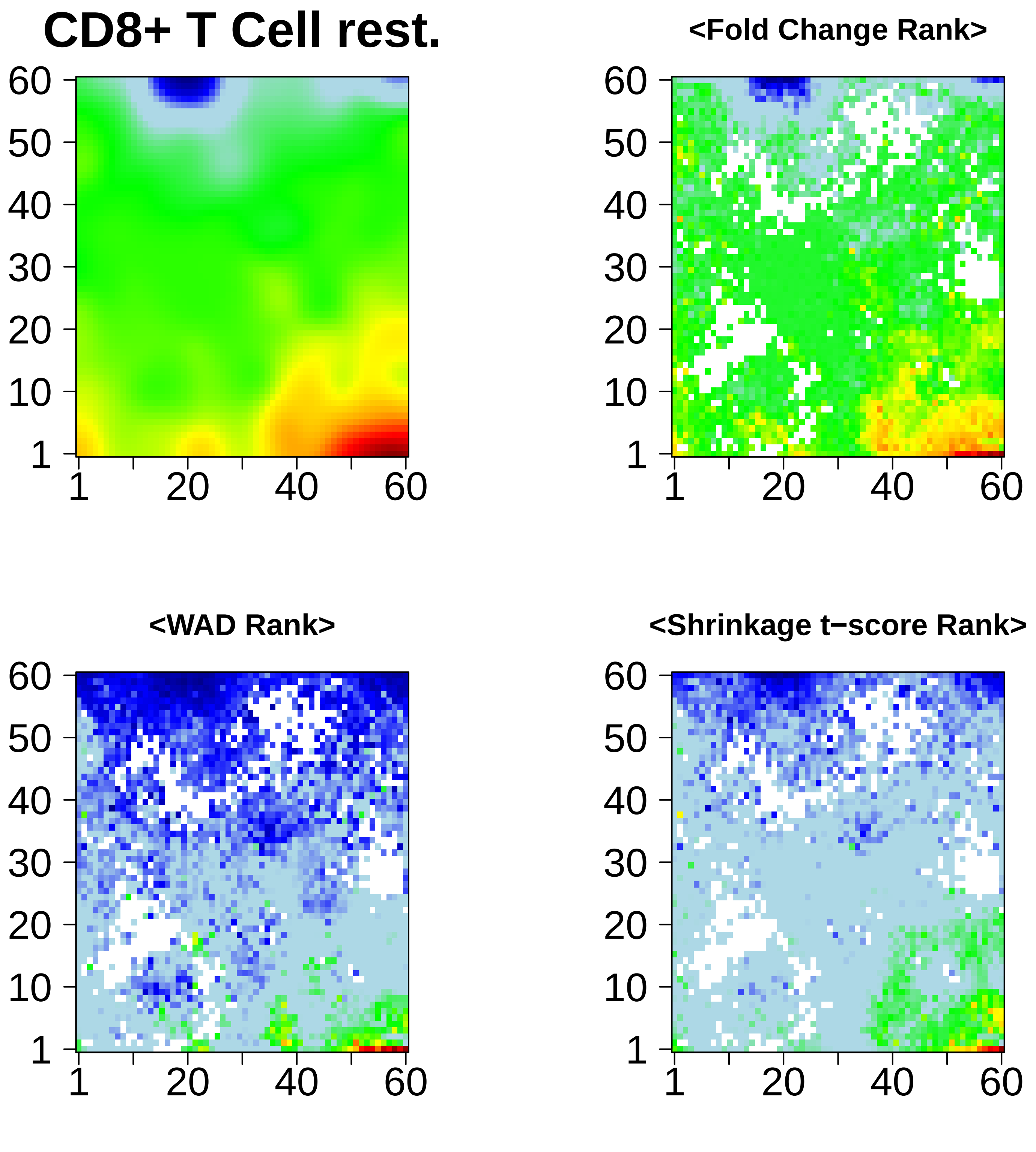

CD8+ T Cell rest.
<Fold Change Rank>
<WAD Rank>
<Shrinkage t−score Rank>
60
50
40
30
20
10
1
1
20
40
60

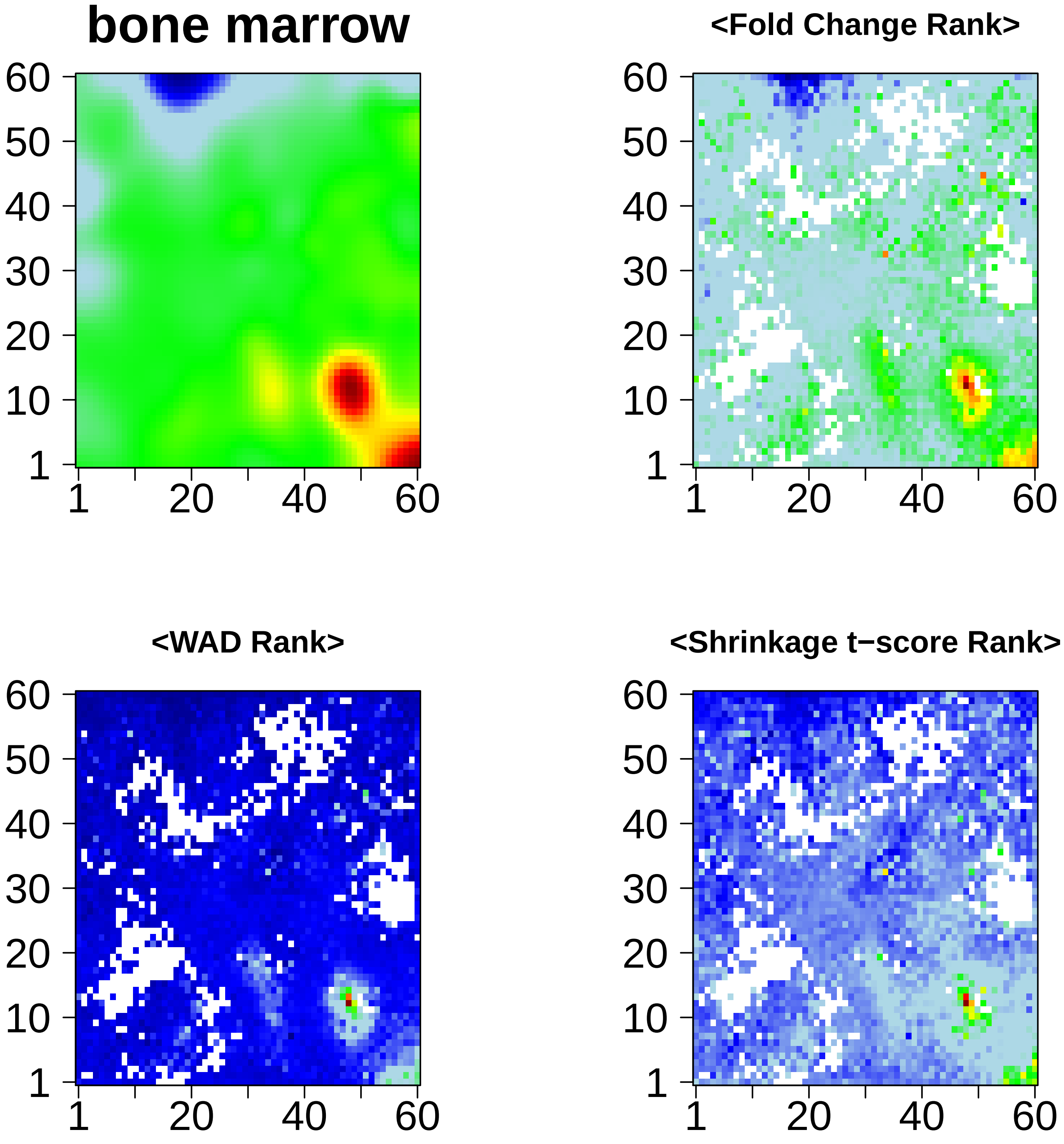

bone marrow
<Fold Change Rank>
<WAD Rank>
<Shrinkage t−score Rank>
60
50
40
30
20
10
1
1
20
40
60

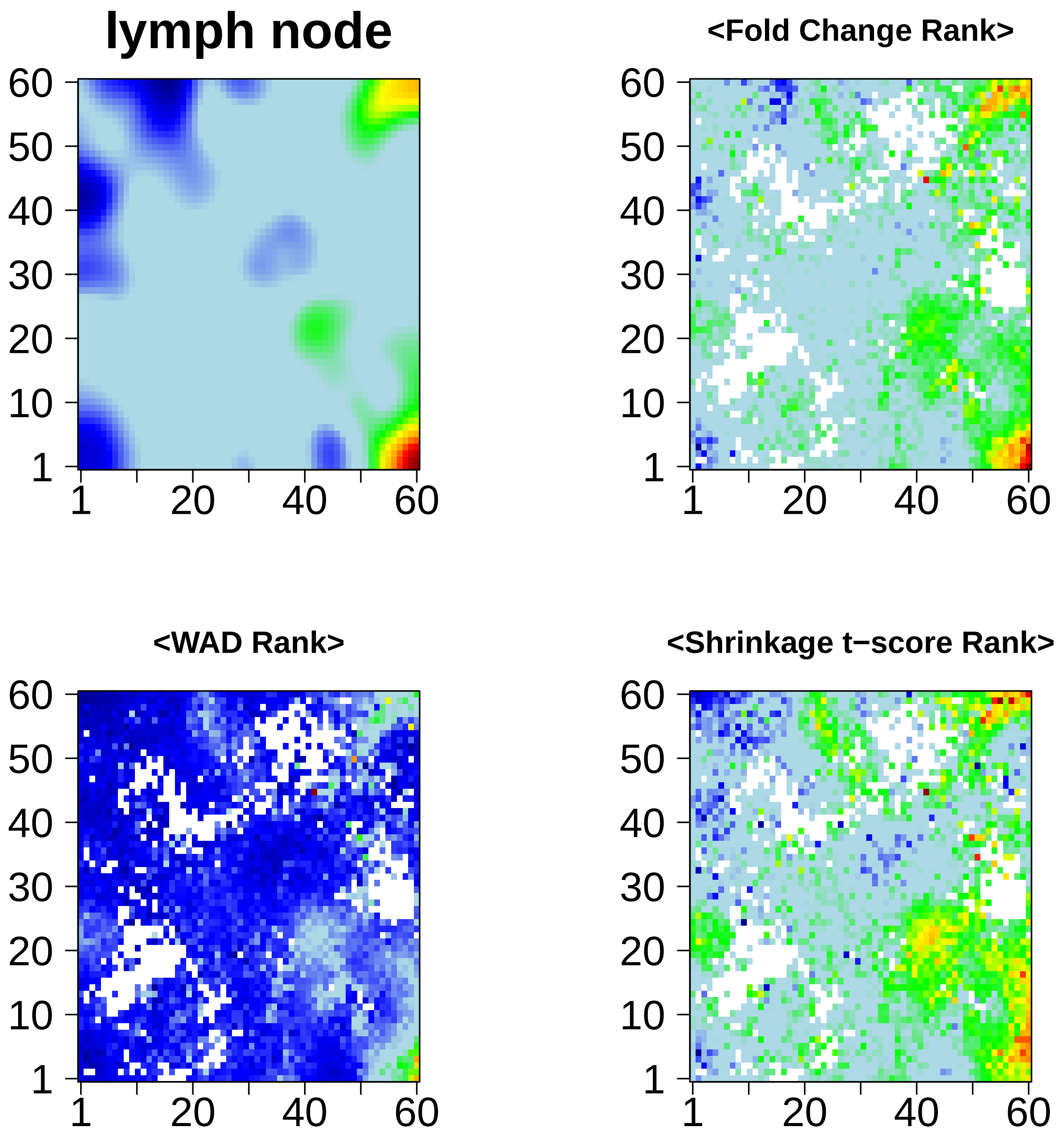
lymph node
<Fold Change Rank>
<WAD Rank>
<Shrinkage t−score Rank>
1
10
20
30
40
50
60

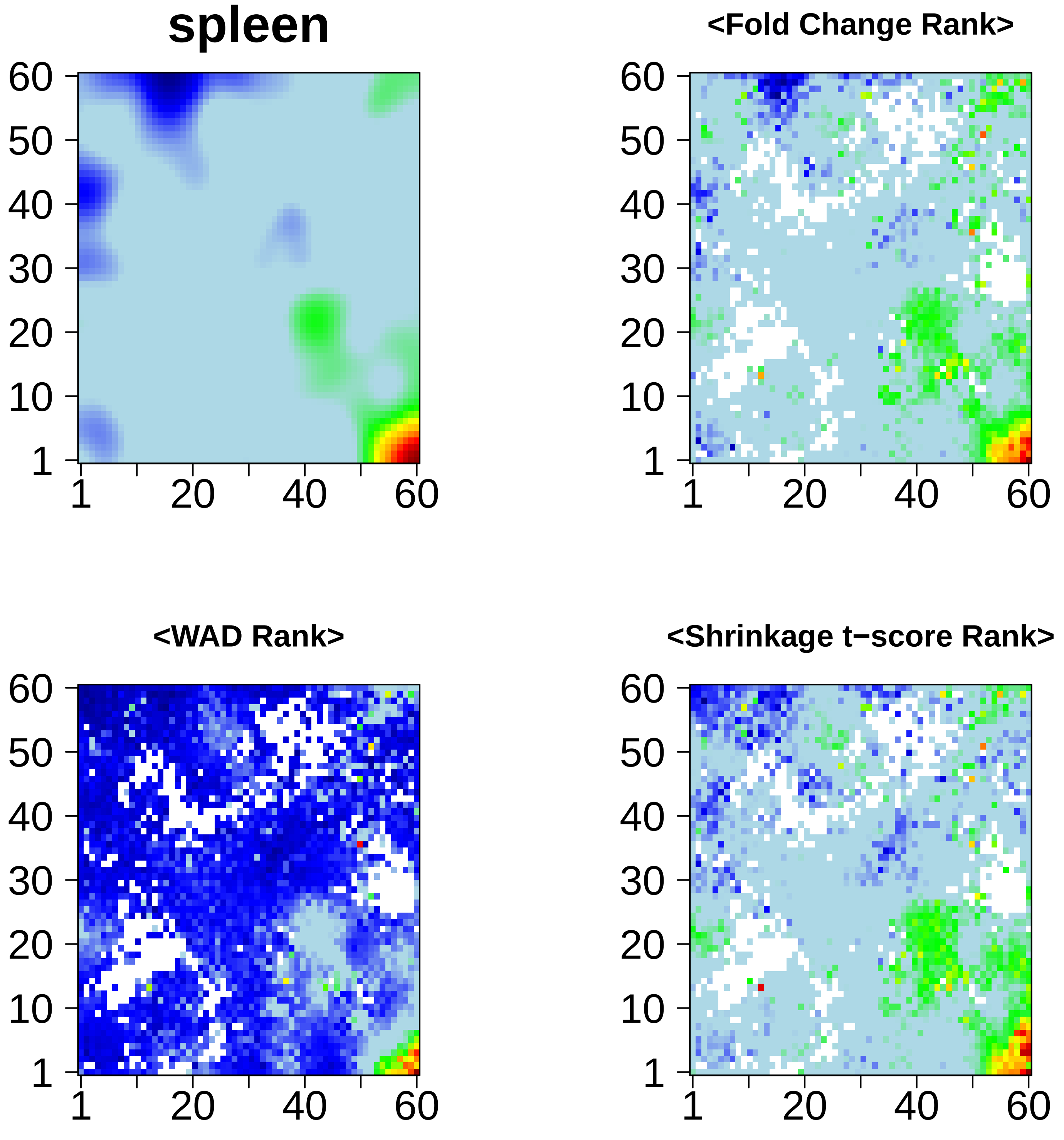

spleen
<Fold Change Rank>
<WAD Rank>
<Shrinkage t−score Rank>
1
10
20
30
40
50
60
1
20
40
60

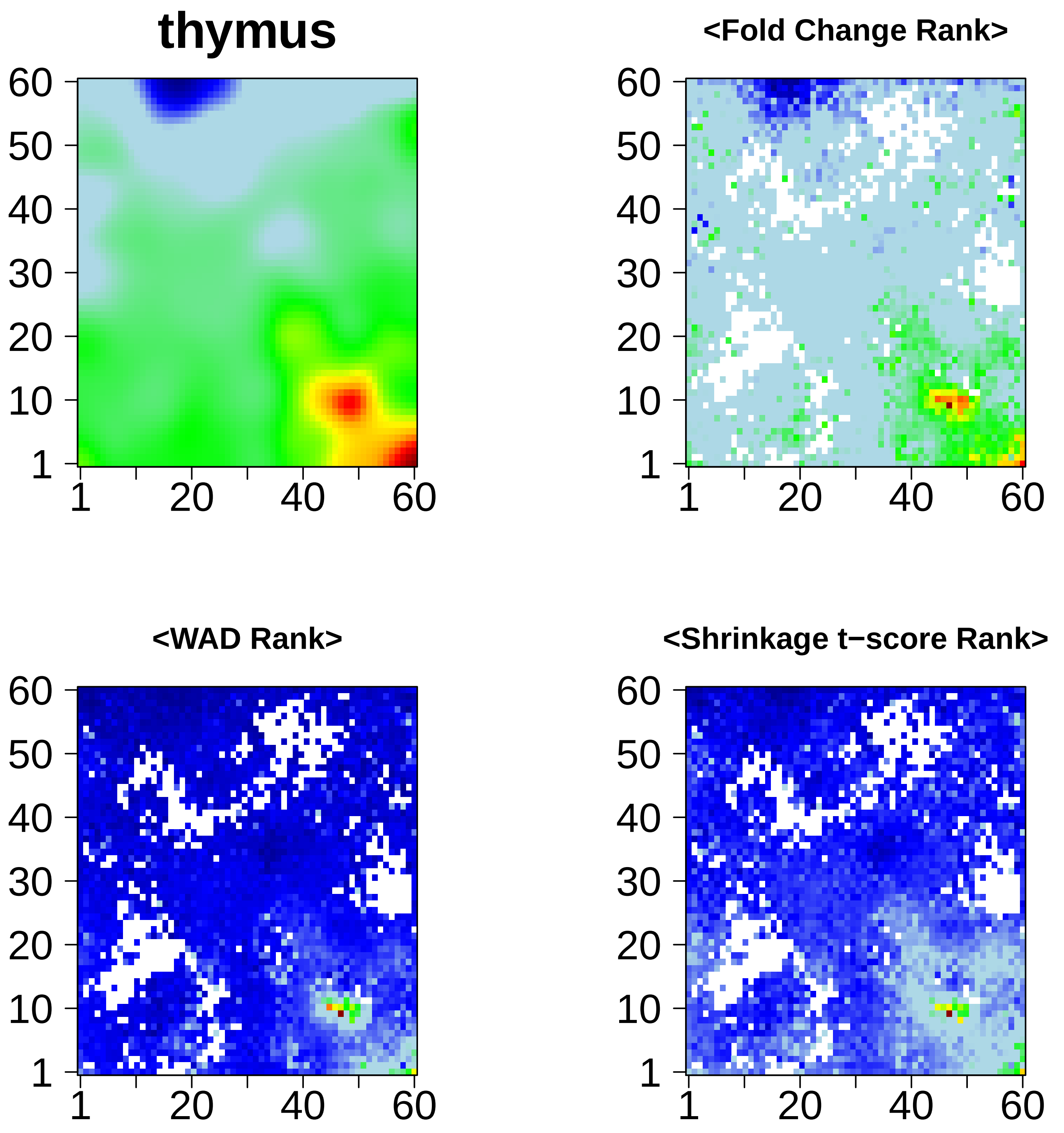
thymus
<Fold Change Rank>
<WAD Rank>
<Shrinkage t−score Rank>
1
10
20
30
40
50
60

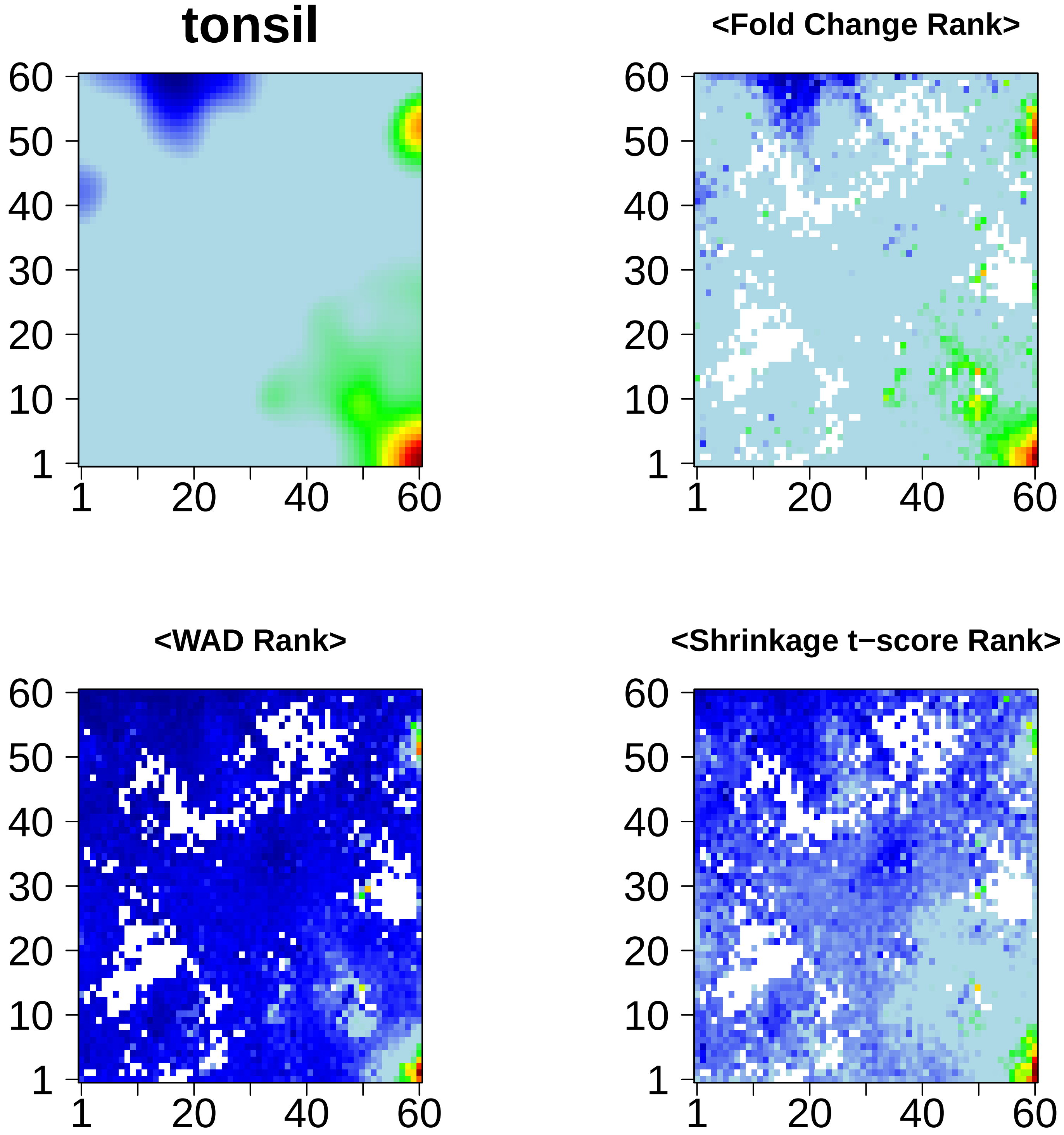
tonsil
<Fold Change Rank>
<WAD Rank>
<Shrinkage t−score Rank>
1
10
20
30
40
50
60

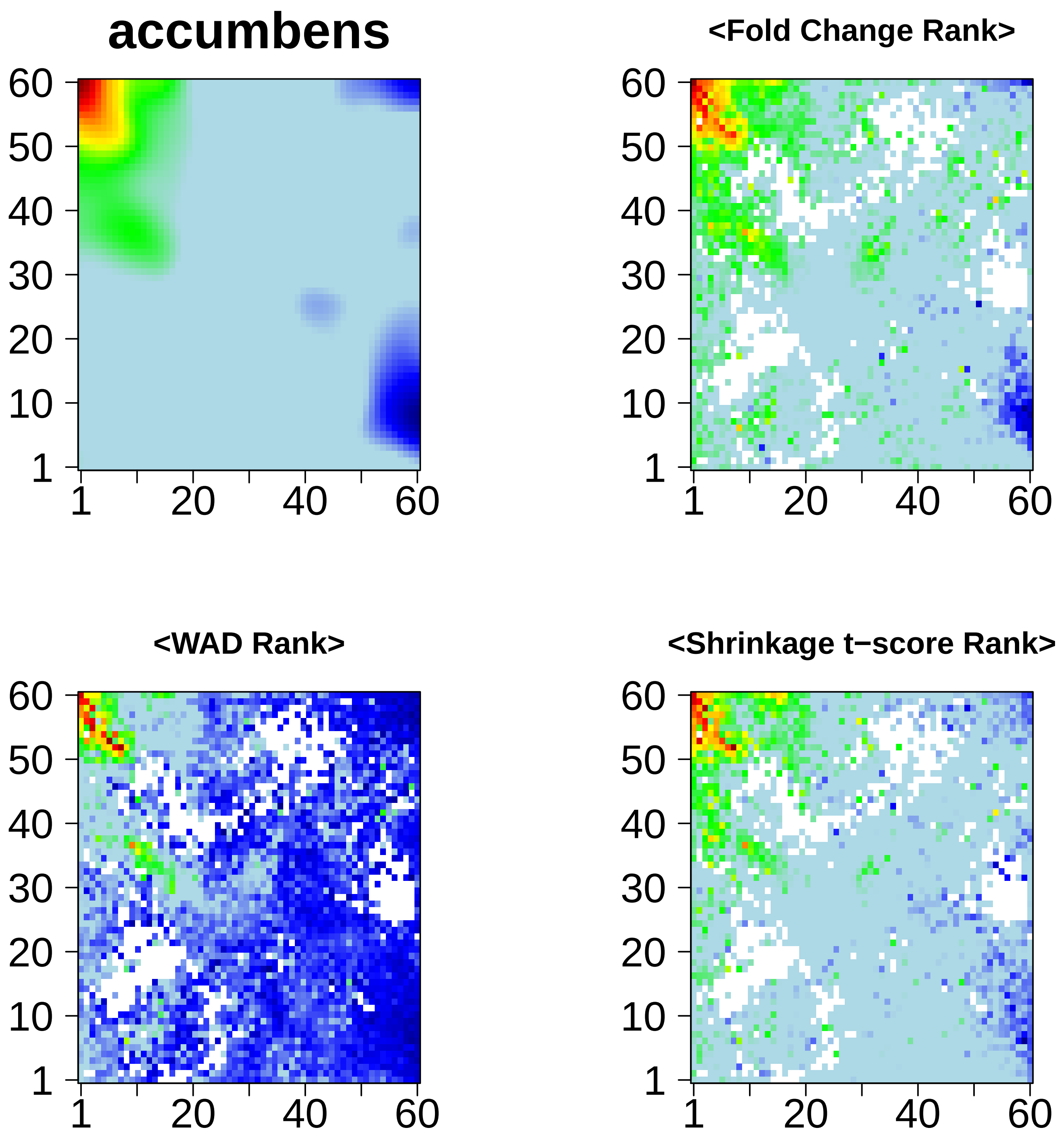
accumbens
<Fold Change Rank>
<WAD Rank>
<Shrinkage t−score Rank>
1
20
40
60
1
10
20
30
40
50
60

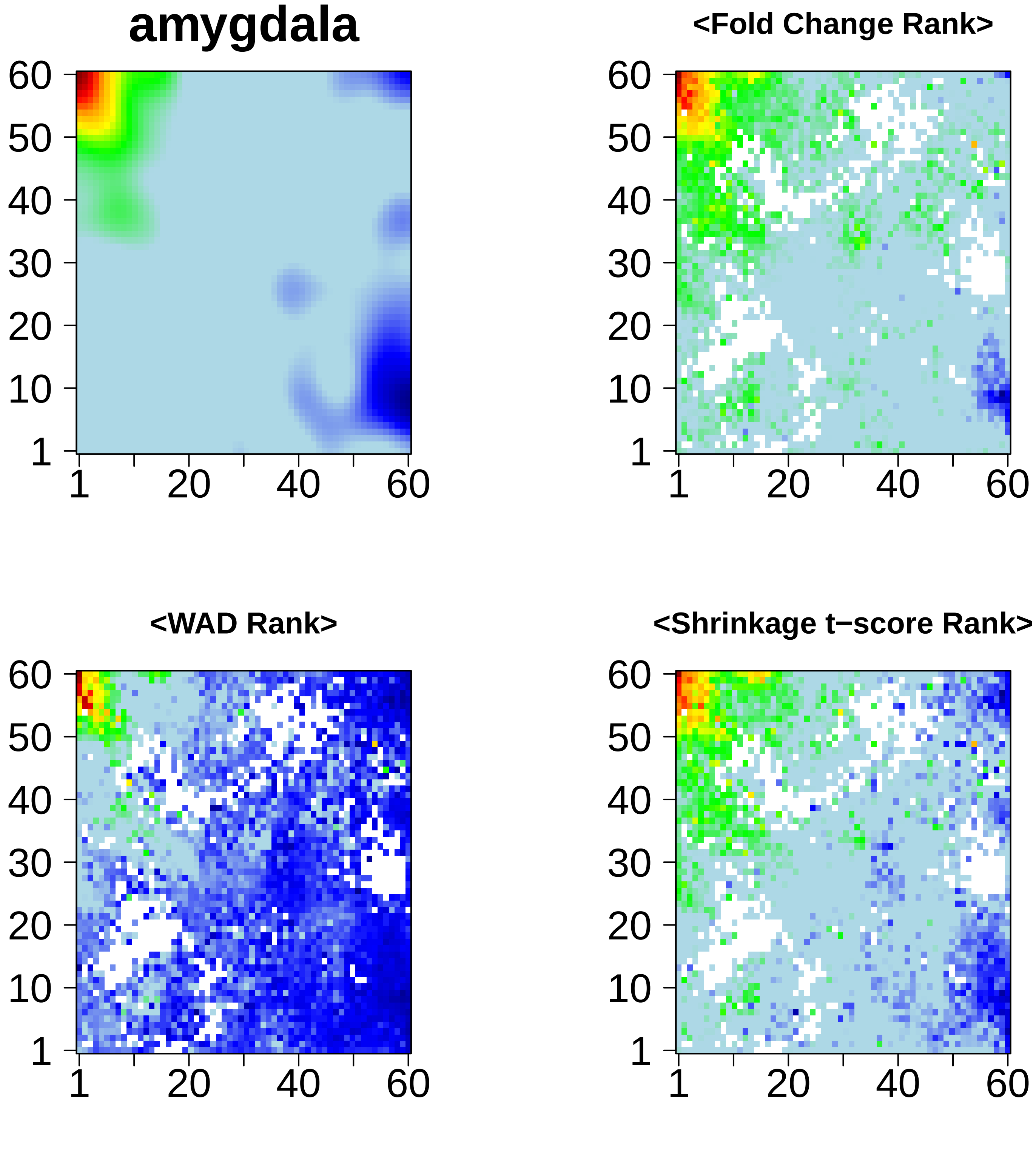
amygdala
<Fold Change Rank>
<WAD Rank>
<Shrinkage t−score Rank>
60
50
40
30
20
10
1
1
20
40
60

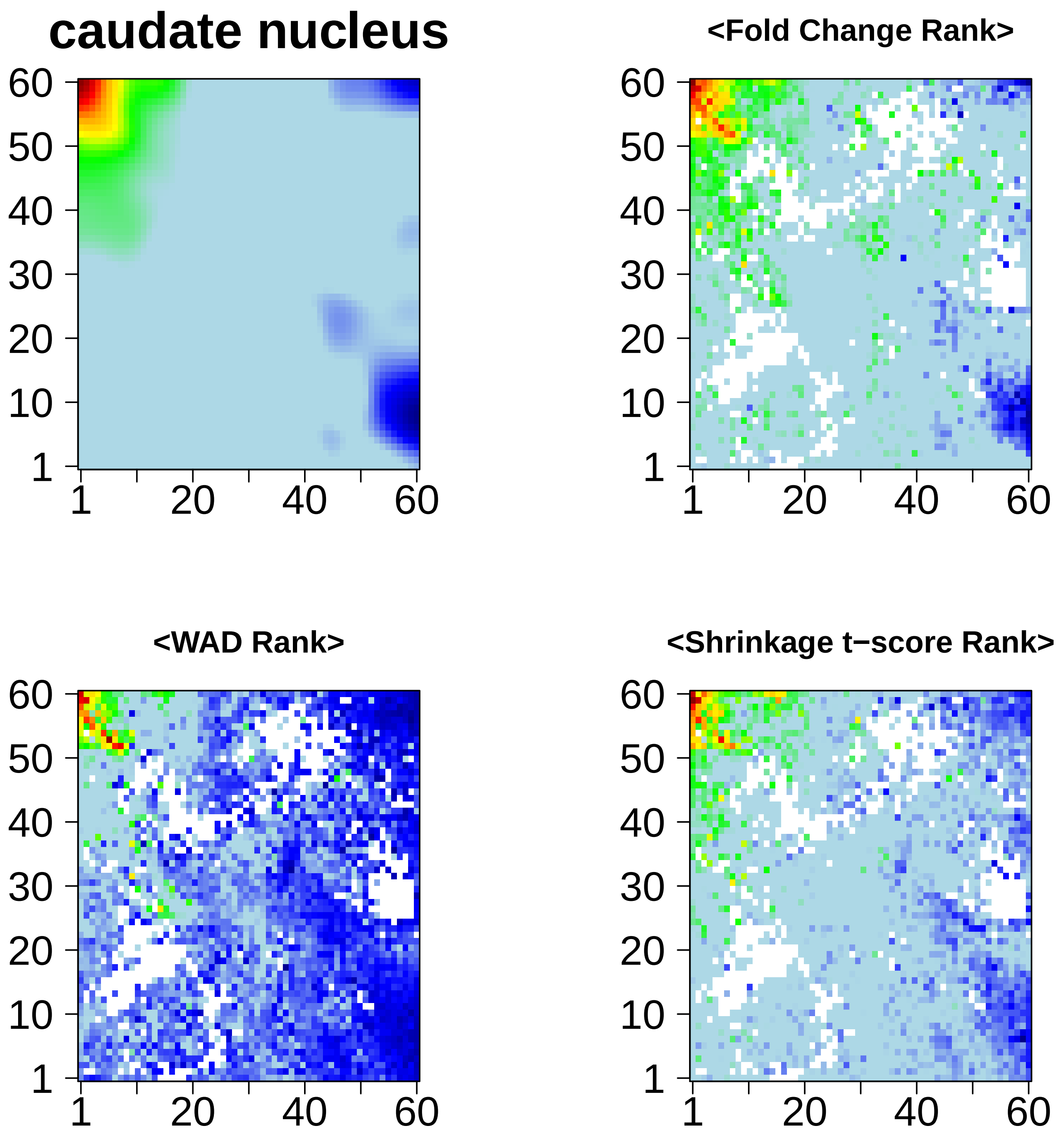
caudate nucleus
<Fold Change Rank>
<WAD Rank>
<Shrinkage t−score Rank>
1
10
20
30
40
50
60

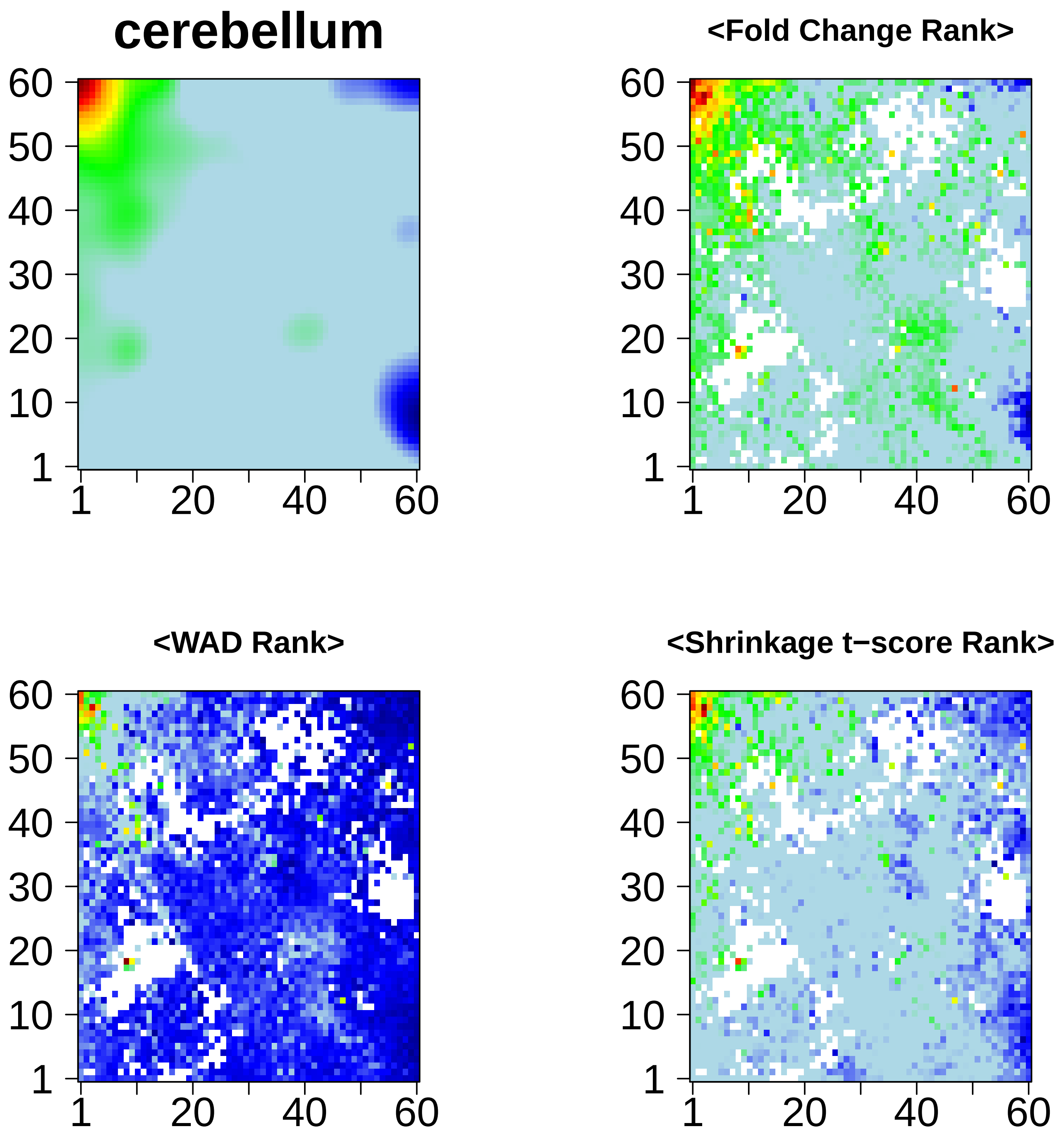
cerebellum
<Fold Change Rank>
<WAD Rank>
<Shrinkage t−score Rank>
60
50
40
30
20
10
1
1
20
40
60

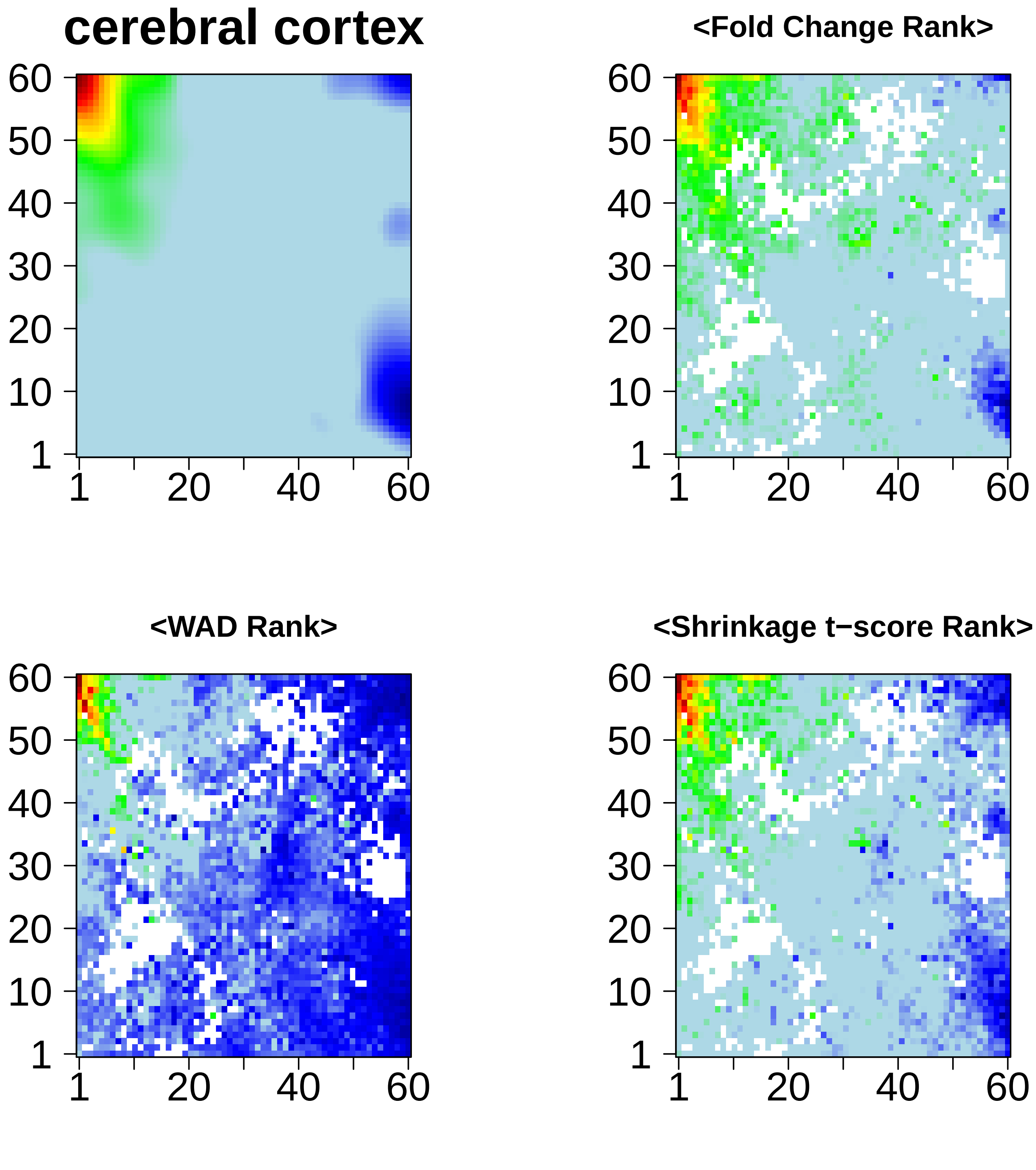
cerebral cortex
<Fold Change Rank>
<WAD Rank>
<Shrinkage t−score Rank>
1
10
20
30
40
50
60

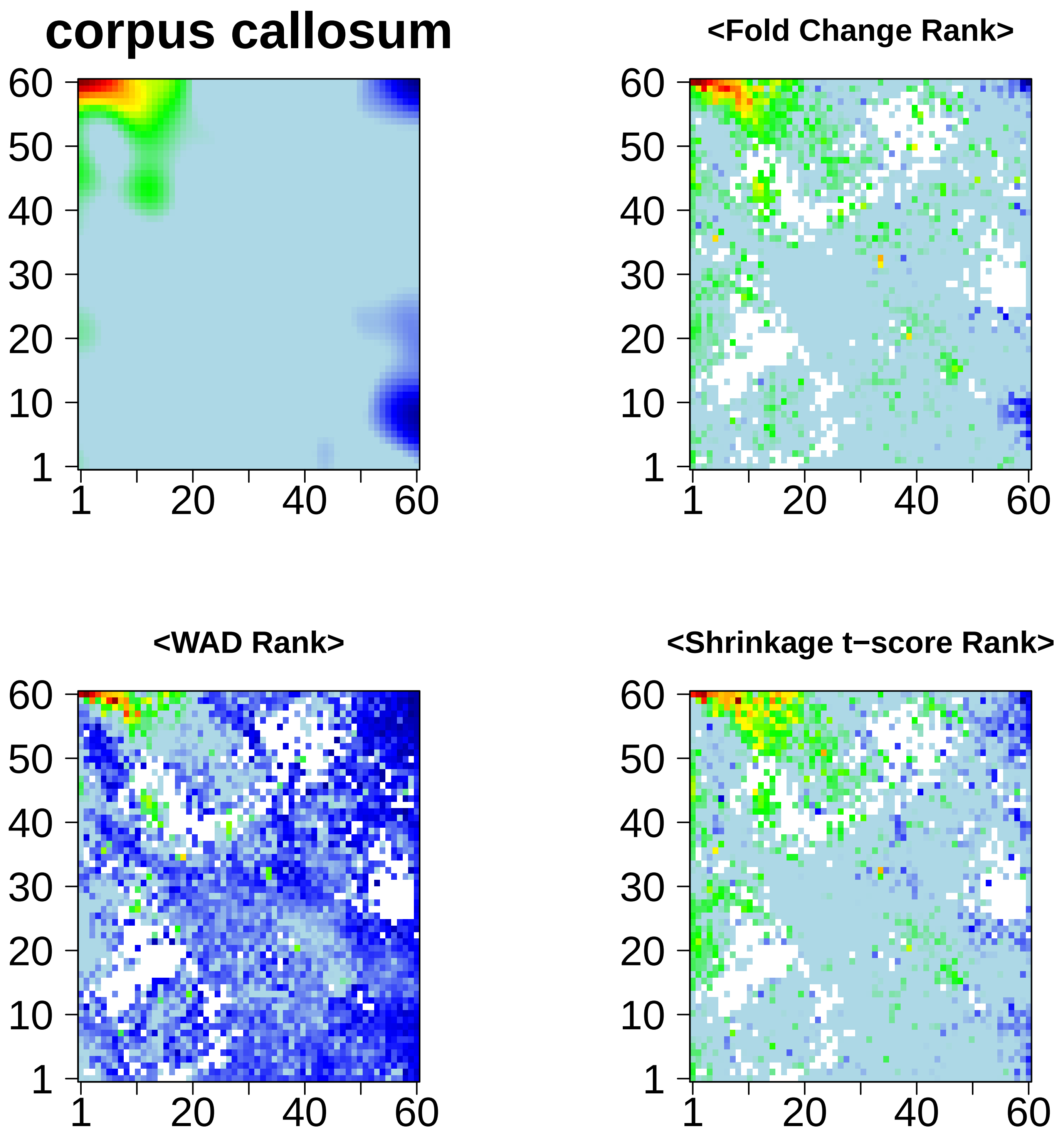
corpus callosum
<Fold Change Rank>
<WAD Rank>
<Shrinkage t−score Rank>
60
50
40
30
20
10
1
1
20
40
60

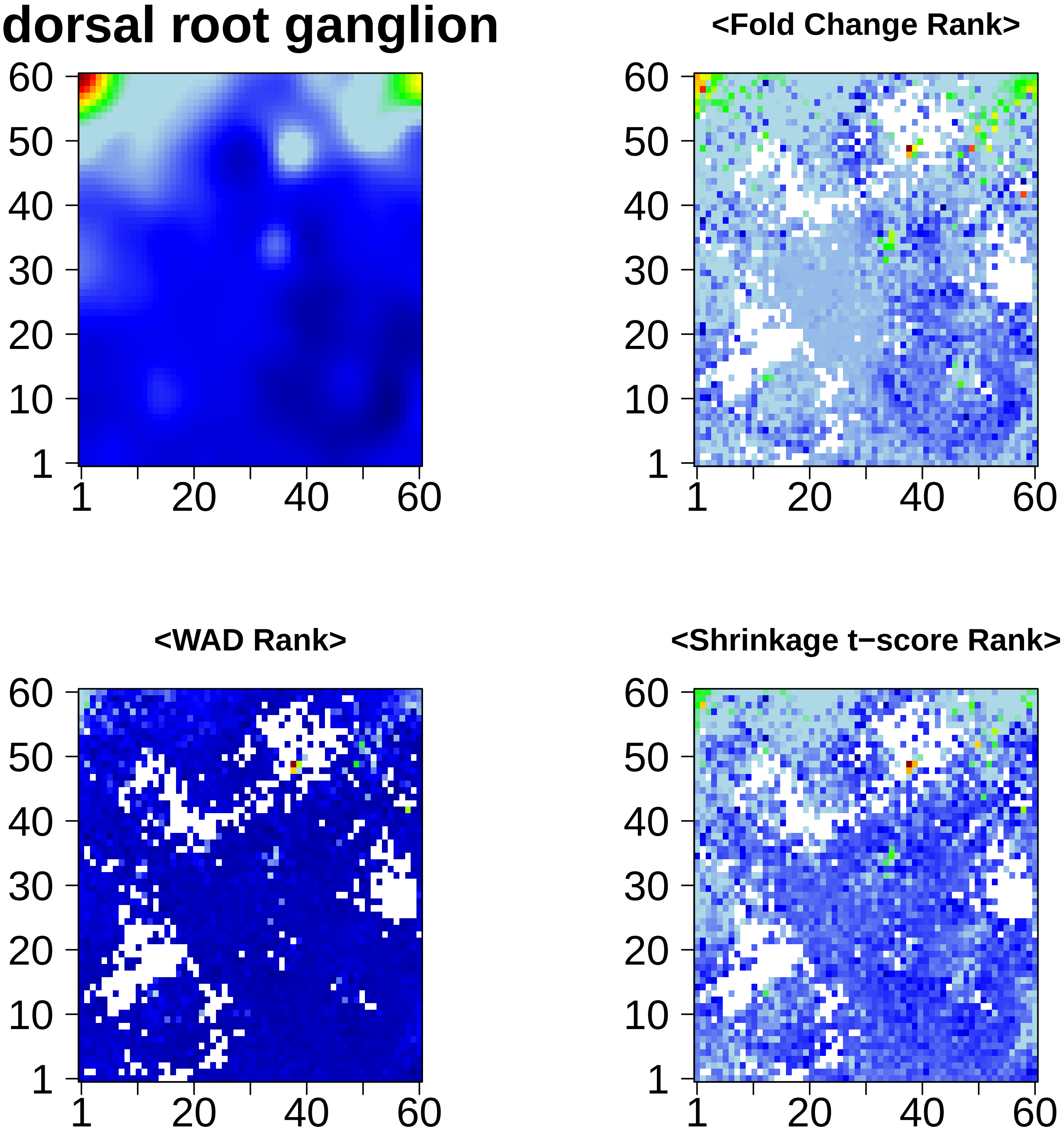
dorsal root ganglion
<Fold Change Rank>
<WAD Rank>
<Shrinkage t−score Rank>
60
50
40
30
20
10
1
1
20
40
60

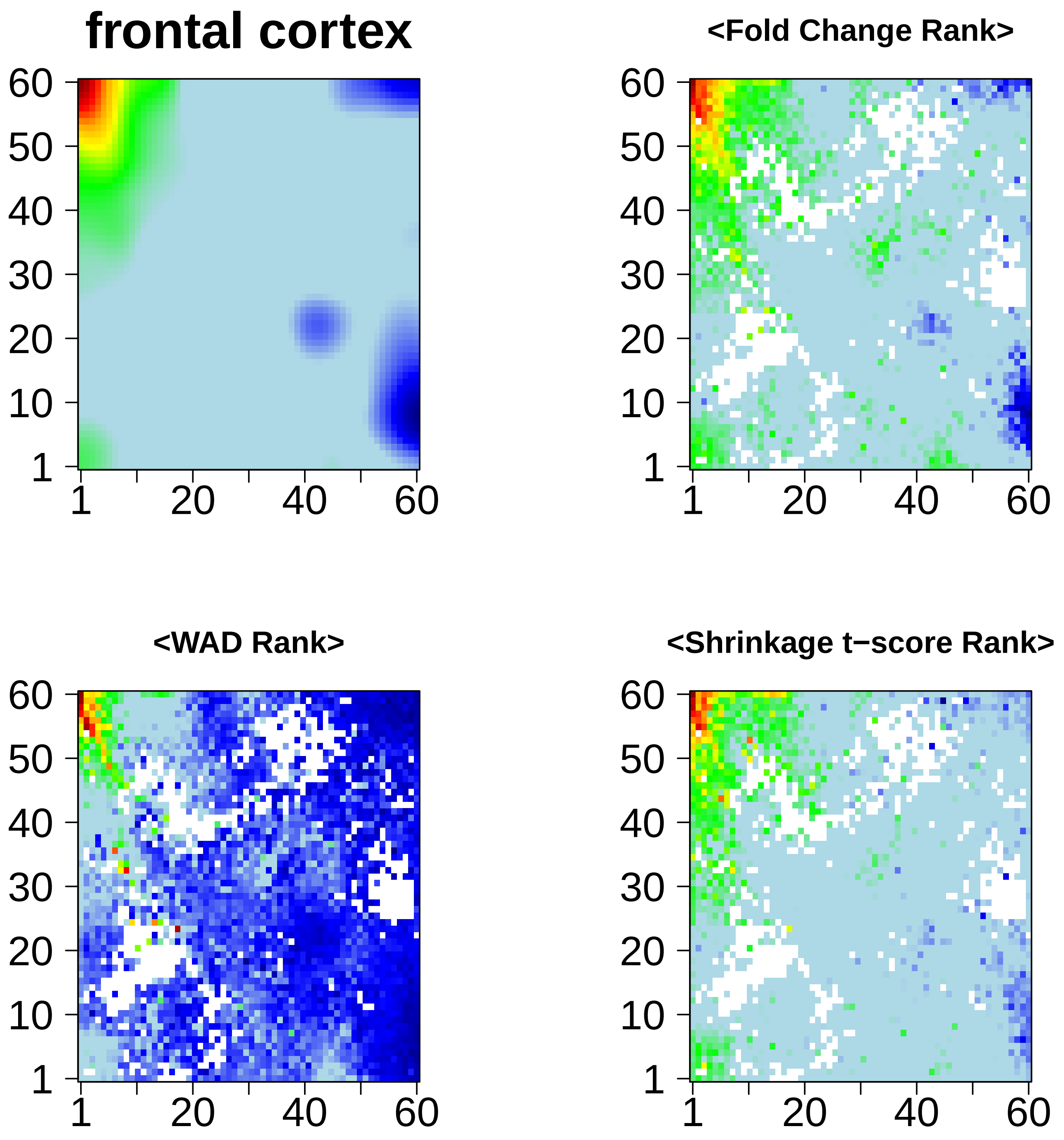
frontal cortex
<Fold Change Rank>
<WAD Rank>
<Shrinkage t−score Rank>
60
50
40
30
20
10
1
1
20
40
60

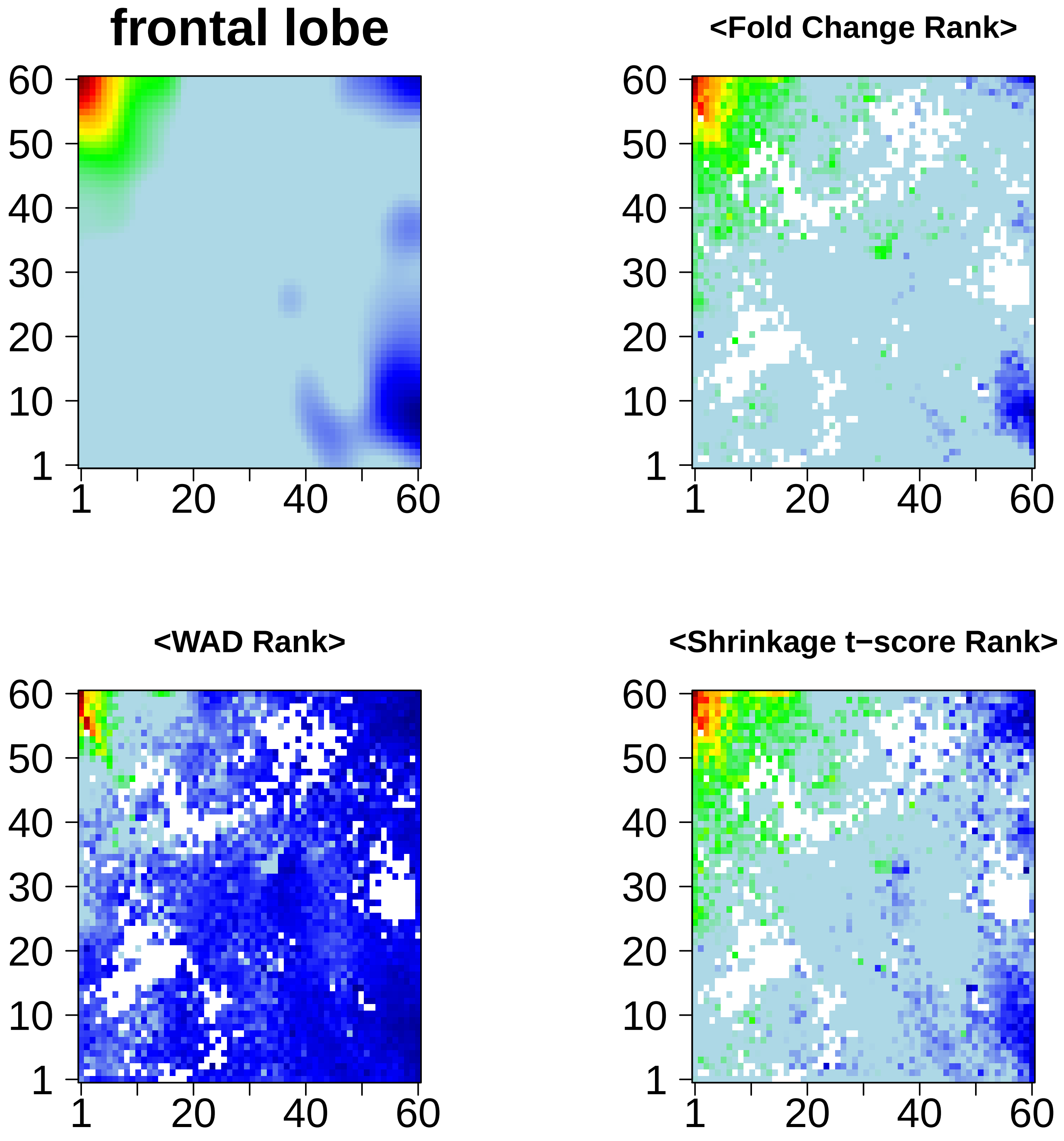
frontal lobe
<Fold Change Rank>
<WAD Rank>
<Shrinkage t−score Rank>
60
50
40
30
20
10
1
1
20
40
60

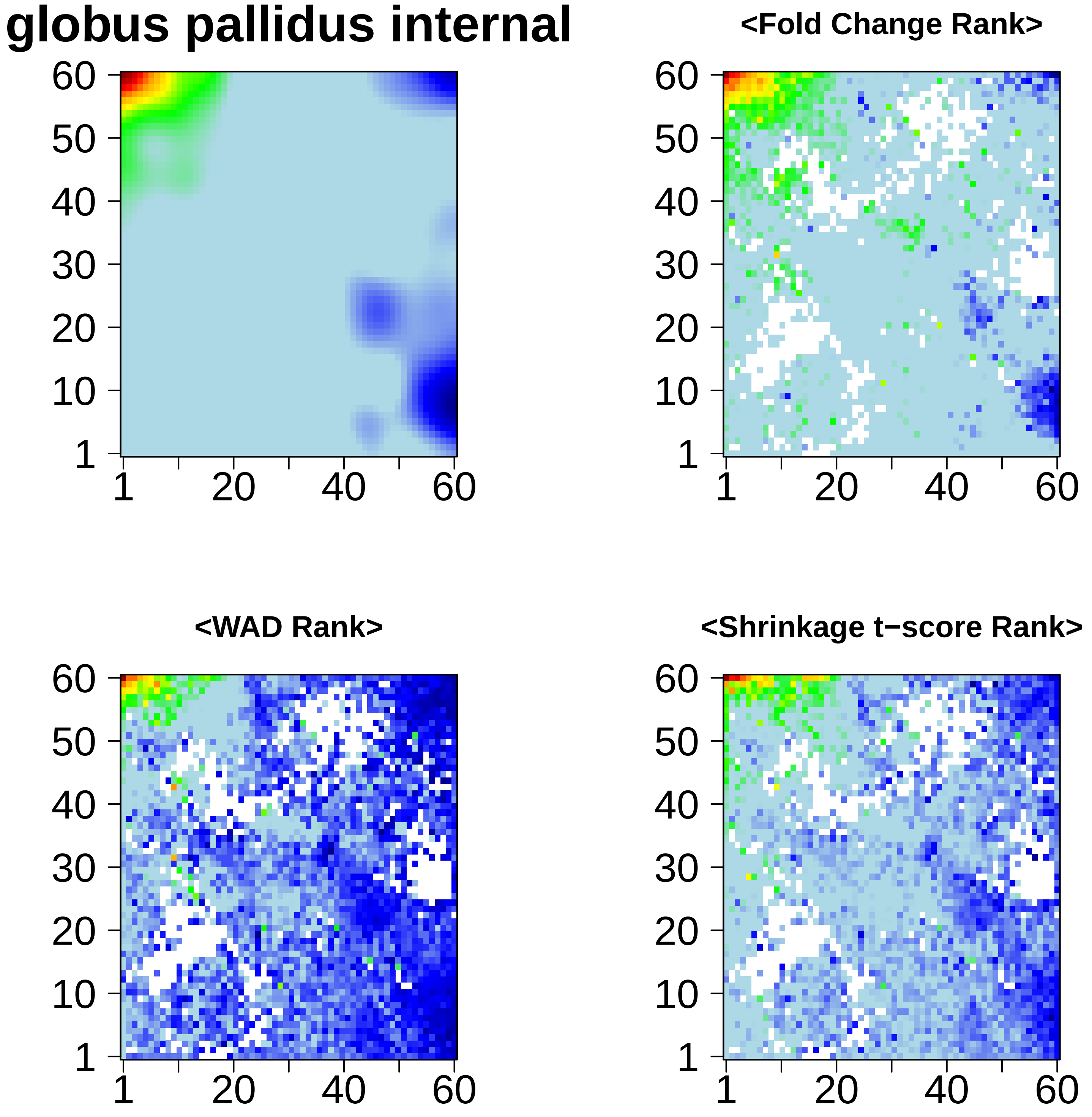
globus pallidus internal
<Fold Change Rank>
<WAD Rank>
<Shrinkage t−score Rank>
60
50
40
30
20
10
1
1
20
40
60

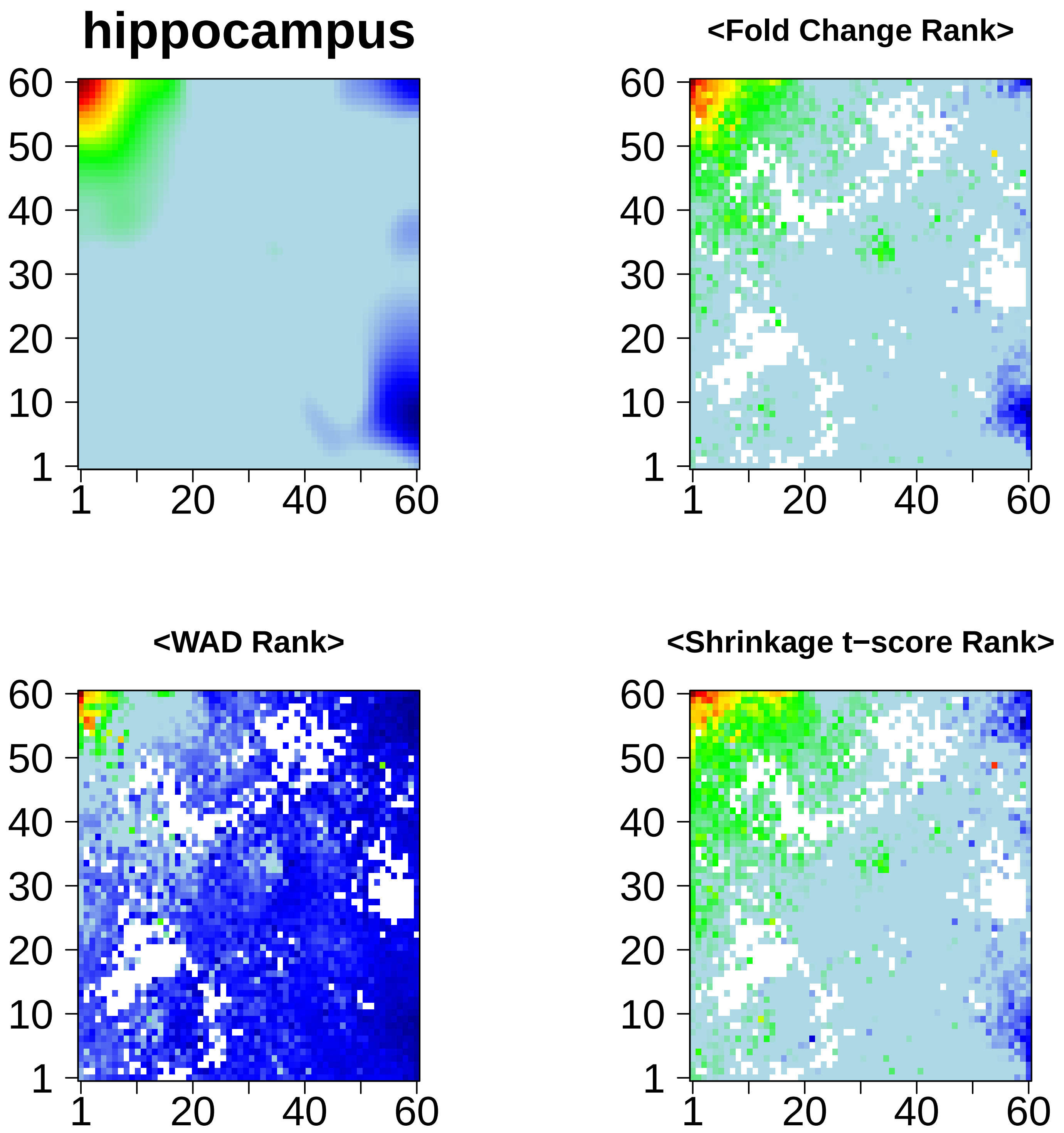
hippocampus
<Fold Change Rank>
<WAD Rank>
<Shrinkage t−score Rank>
60
50
40
30
20
10
1
1
20
40
60

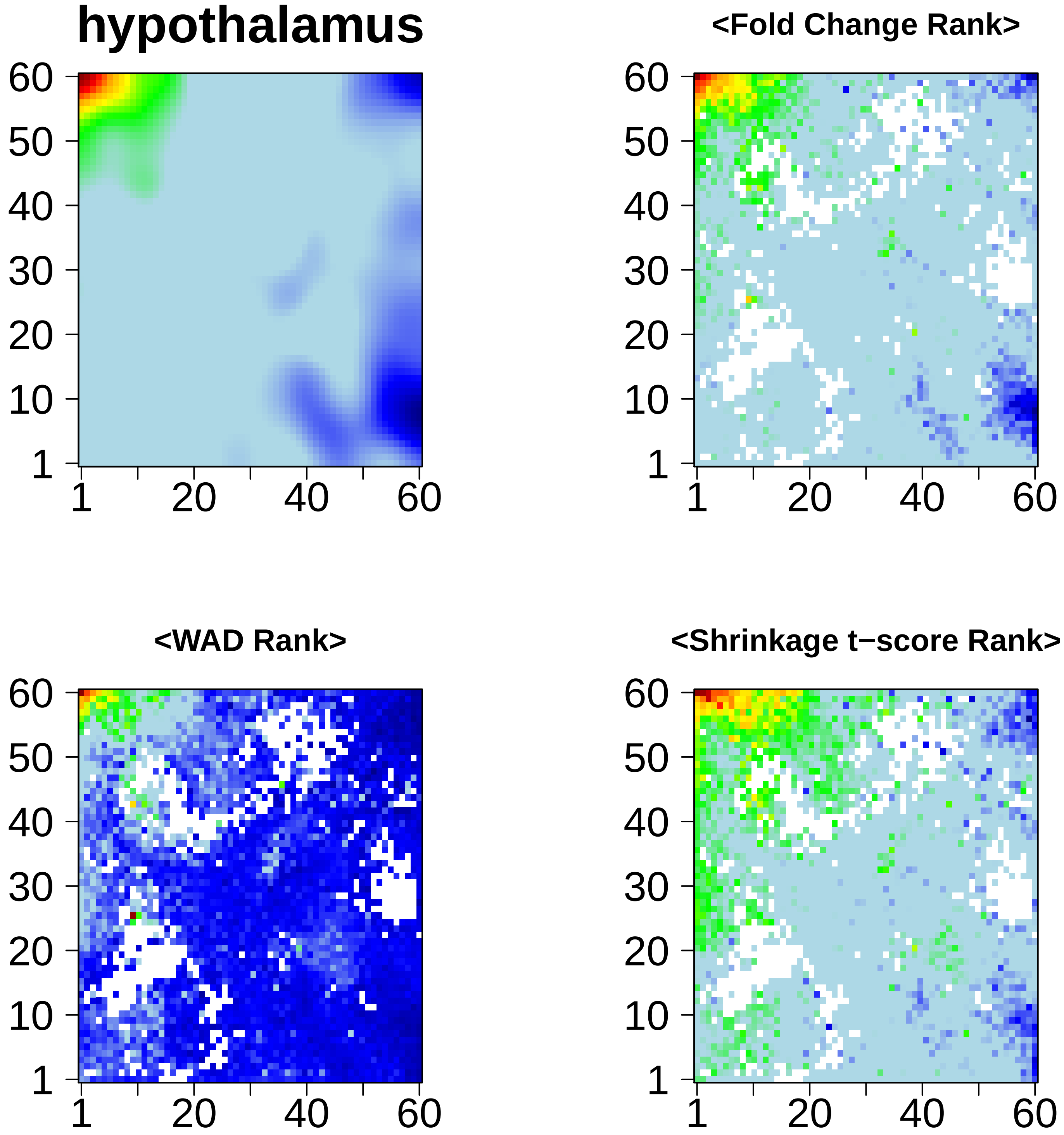
hypothalamus
<Fold Change Rank>
<WAD Rank>
<Shrinkage t−score Rank>
1
10
20
30
40
50
60
1
20
40
60

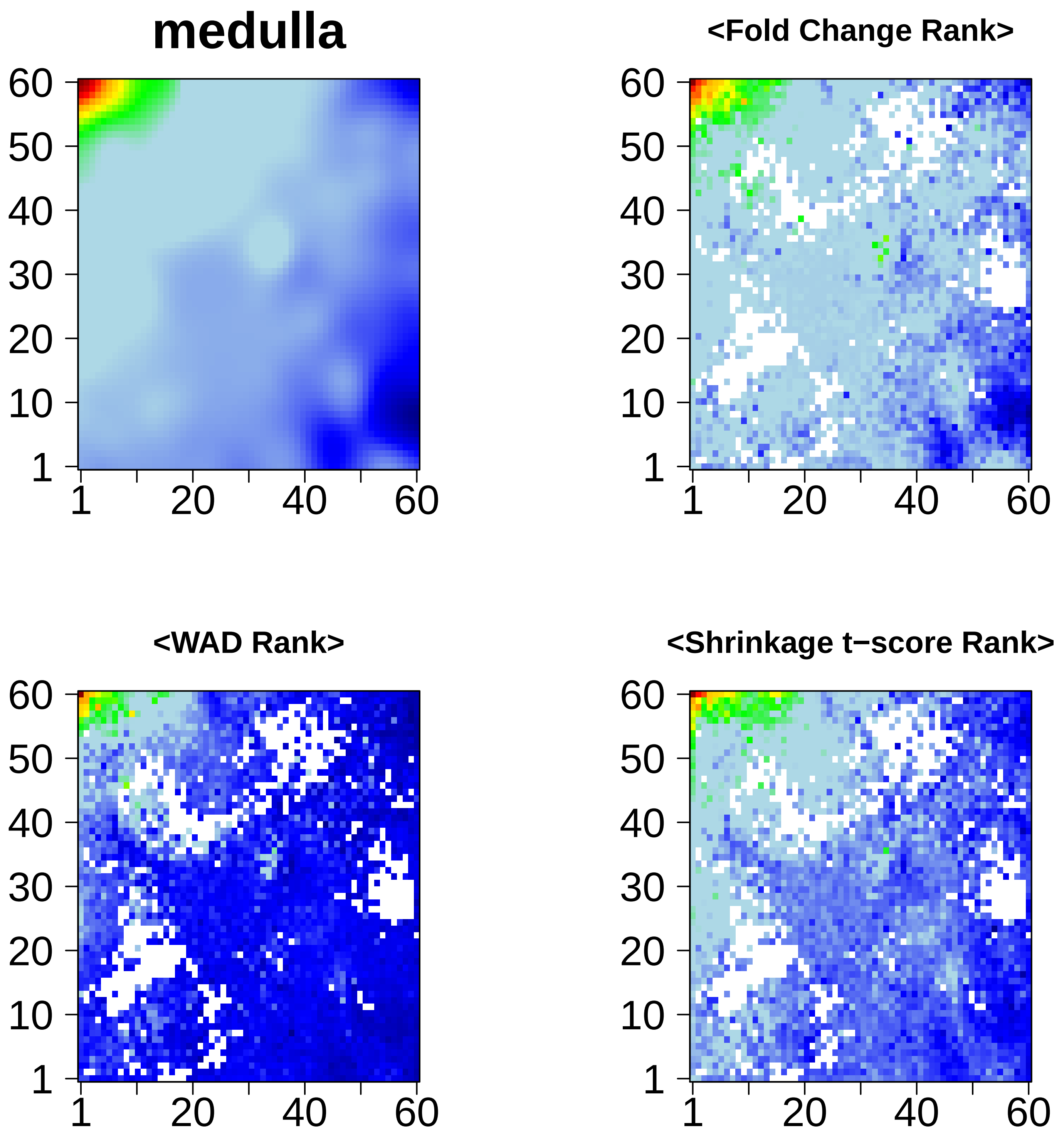

medulla
<Fold Change Rank>
<WAD Rank>
<Shrinkage t−score Rank>
1
10
20
30
40
50
60

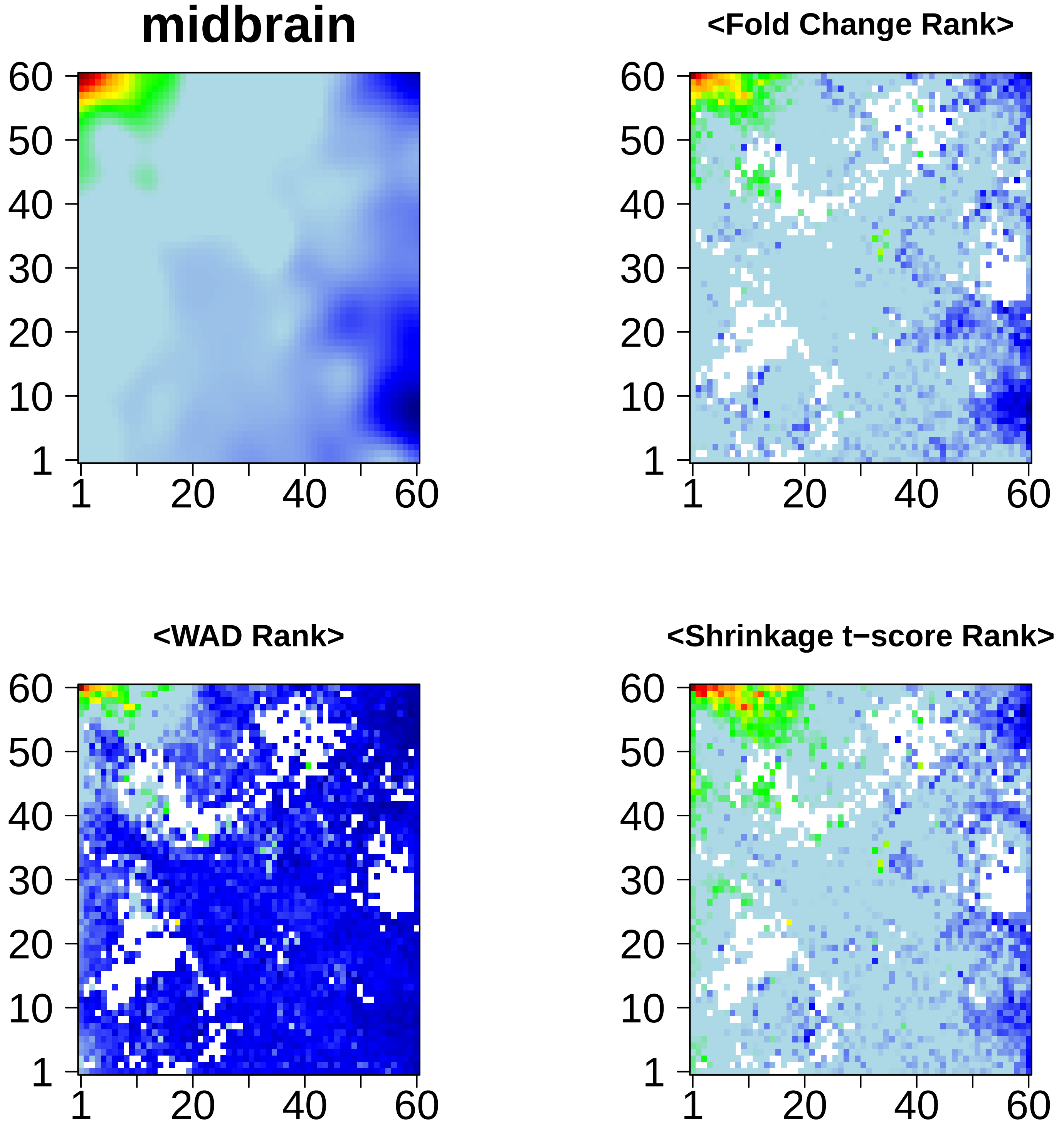
midbrain
<Fold Change Rank>
<WAD Rank>
<Shrinkage t−score Rank>
60
50
40
30
20
10
1
1
20
40
60

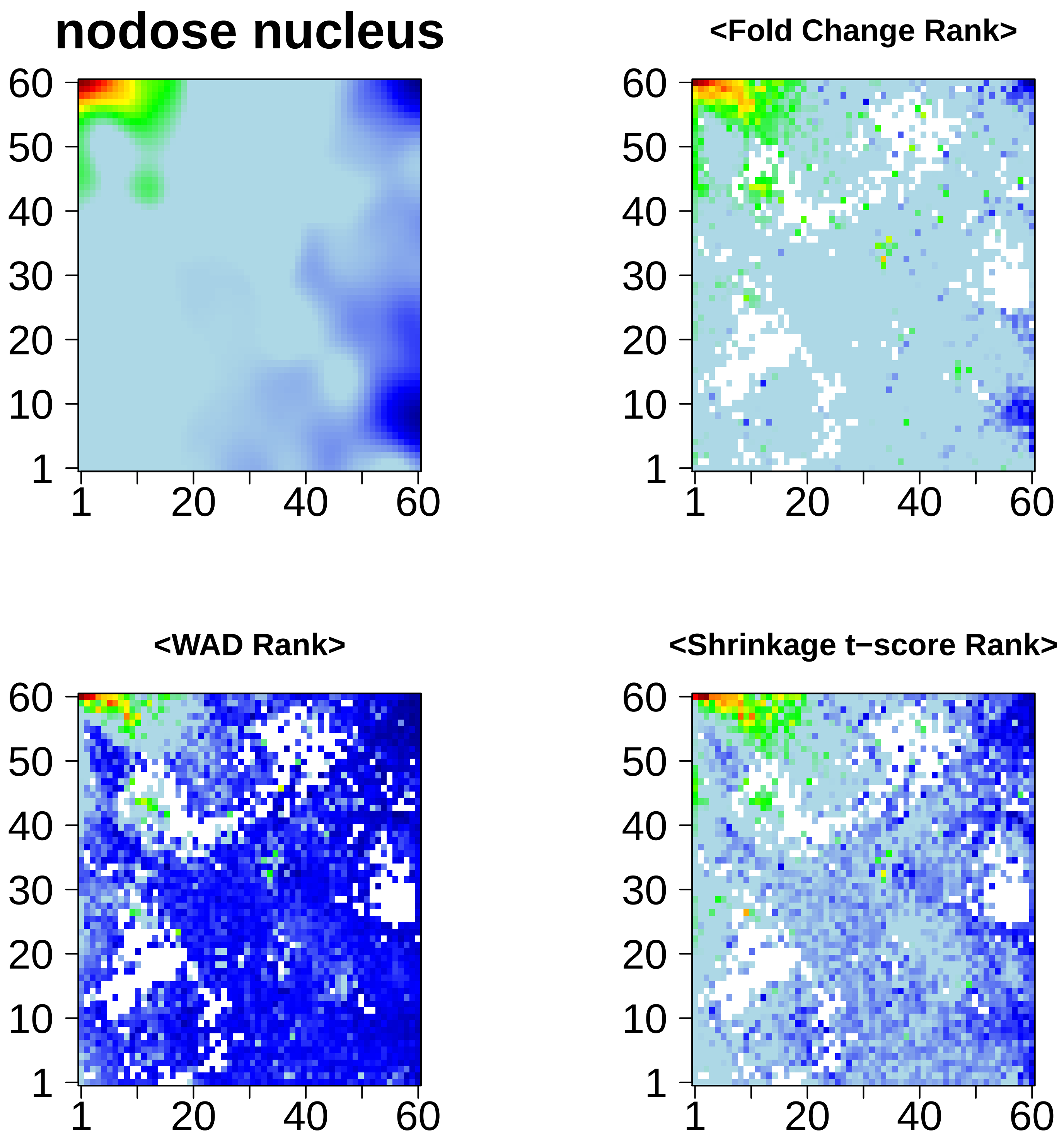
nodose nucleus
<Fold Change Rank>
<WAD Rank>
<Shrinkage t−score Rank>
60
50
40
30
20
10
1
1
20
40
60

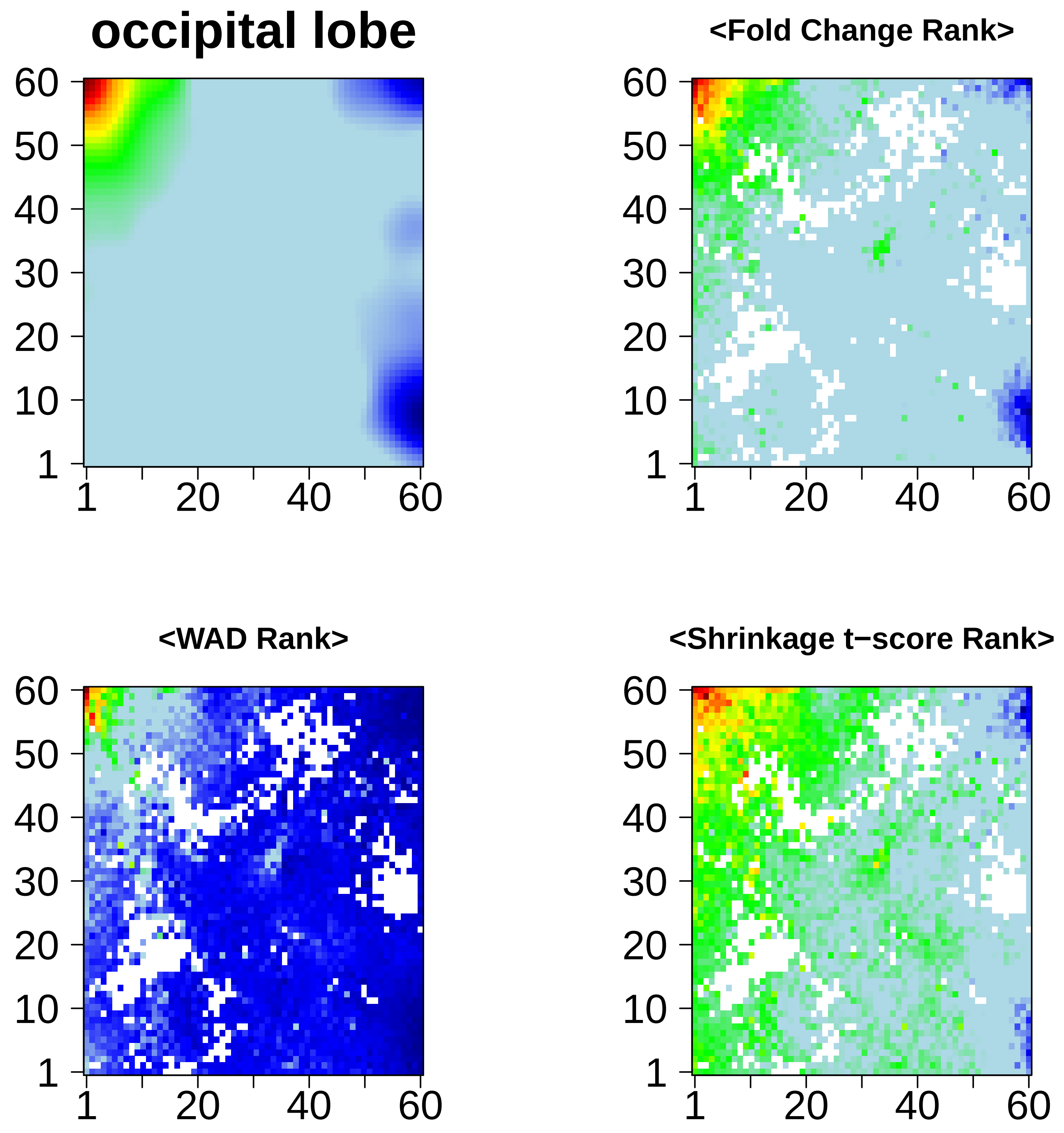
occipital lobe
<Fold Change Rank>
<WAD Rank>
<Shrinkage t−score Rank>
1
10
20
30
40
50
60

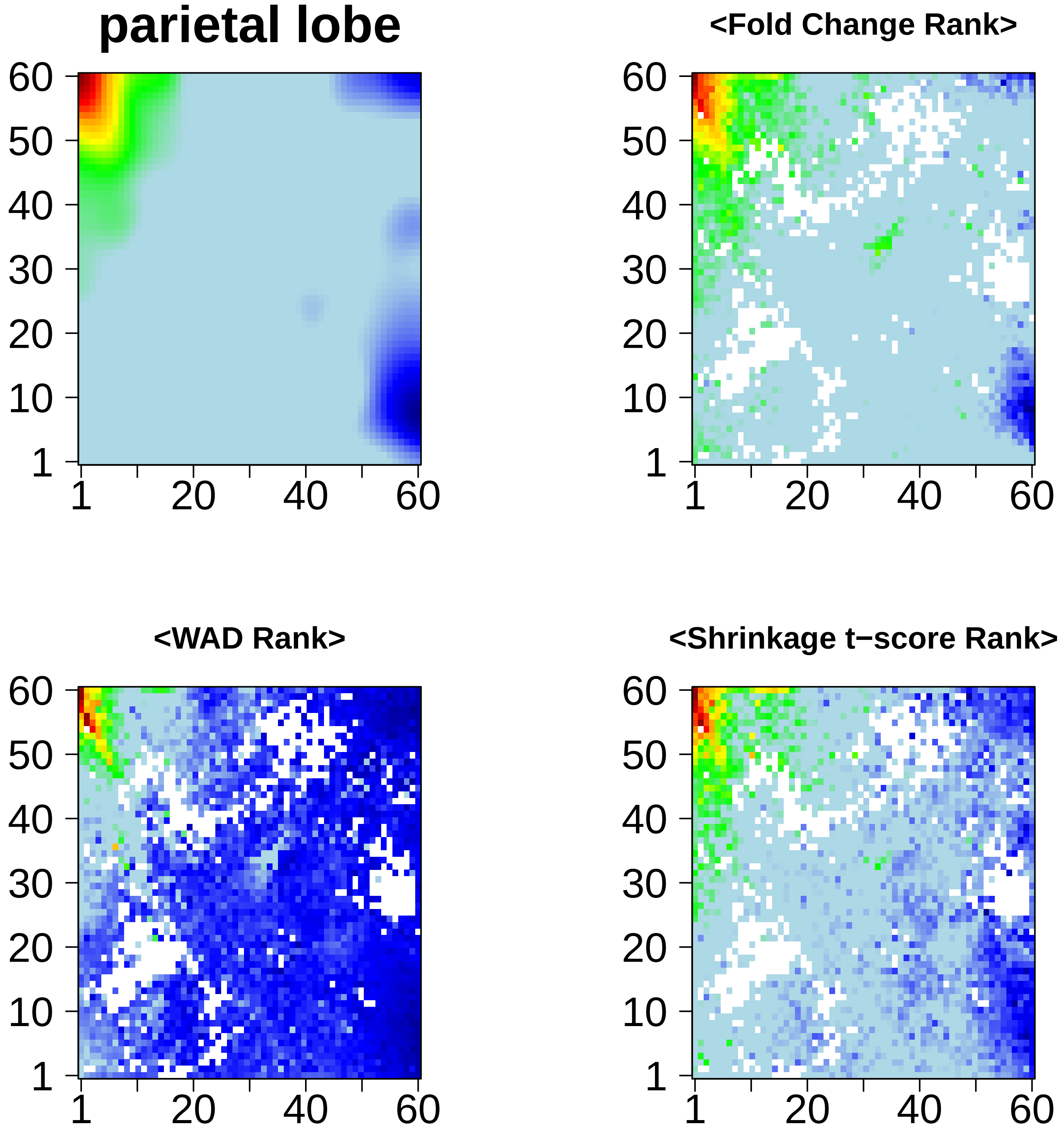
parietal lobe
<Fold Change Rank>
<WAD Rank>
<Shrinkage t−score Rank>
60
50
40
30
20
10
1
1
20
40
60

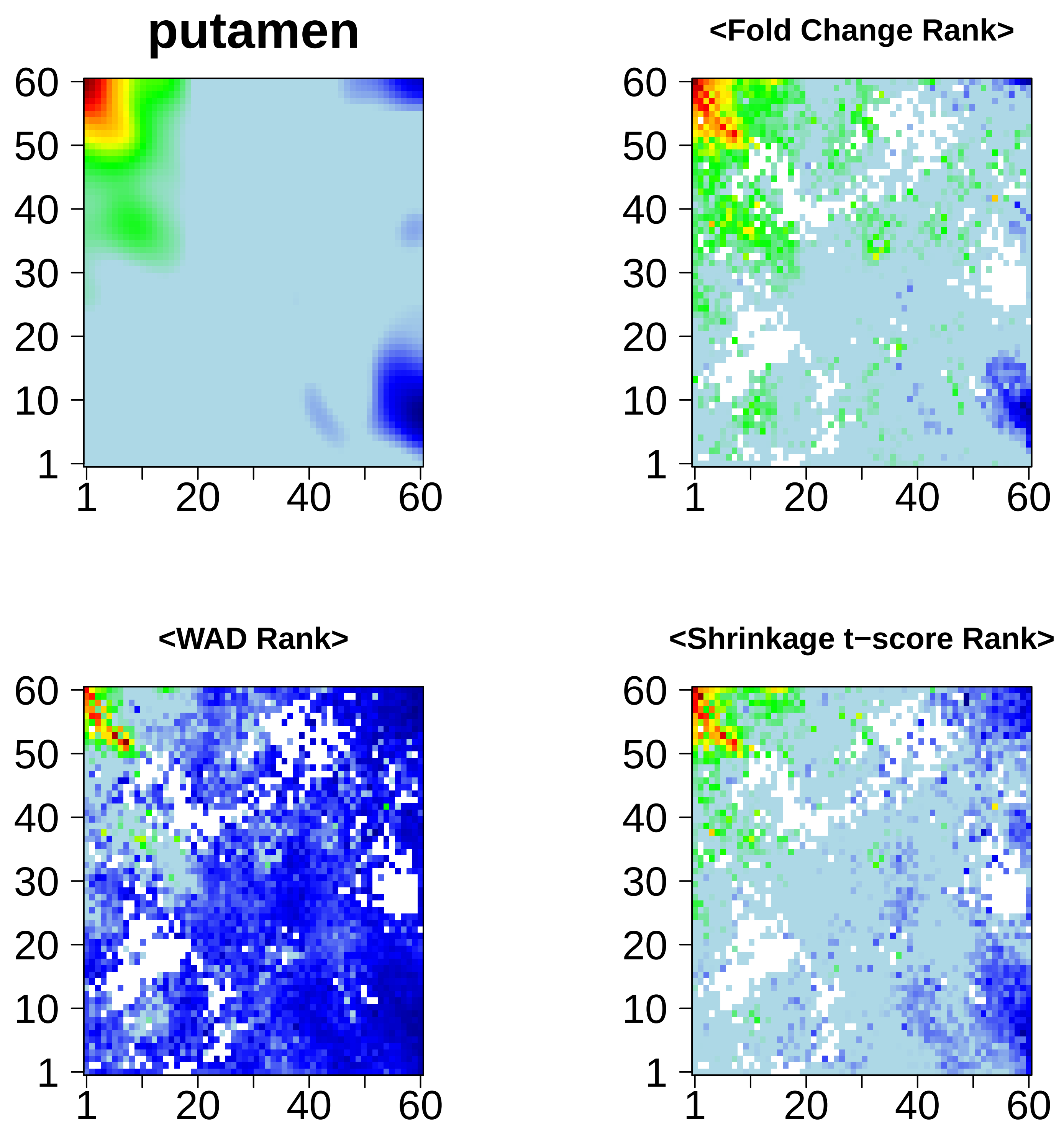
putamen
<Fold Change Rank>
<WAD Rank>
<Shrinkage t−score Rank>
1
10
20
30
40
50
60

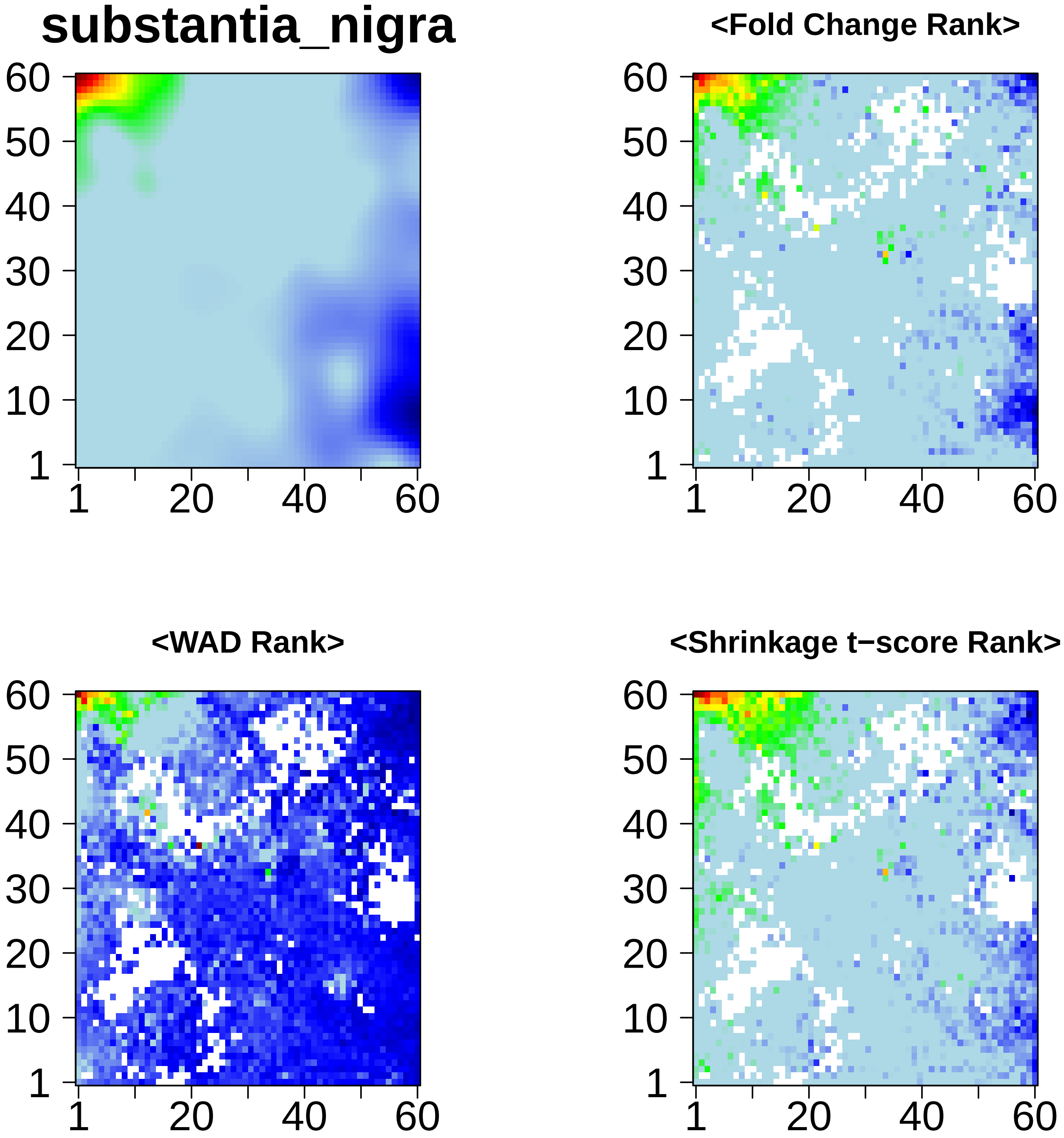

substantia_nigra
<Fold Change Rank>
<WAD Rank>
<Shrinkage t−score Rank>

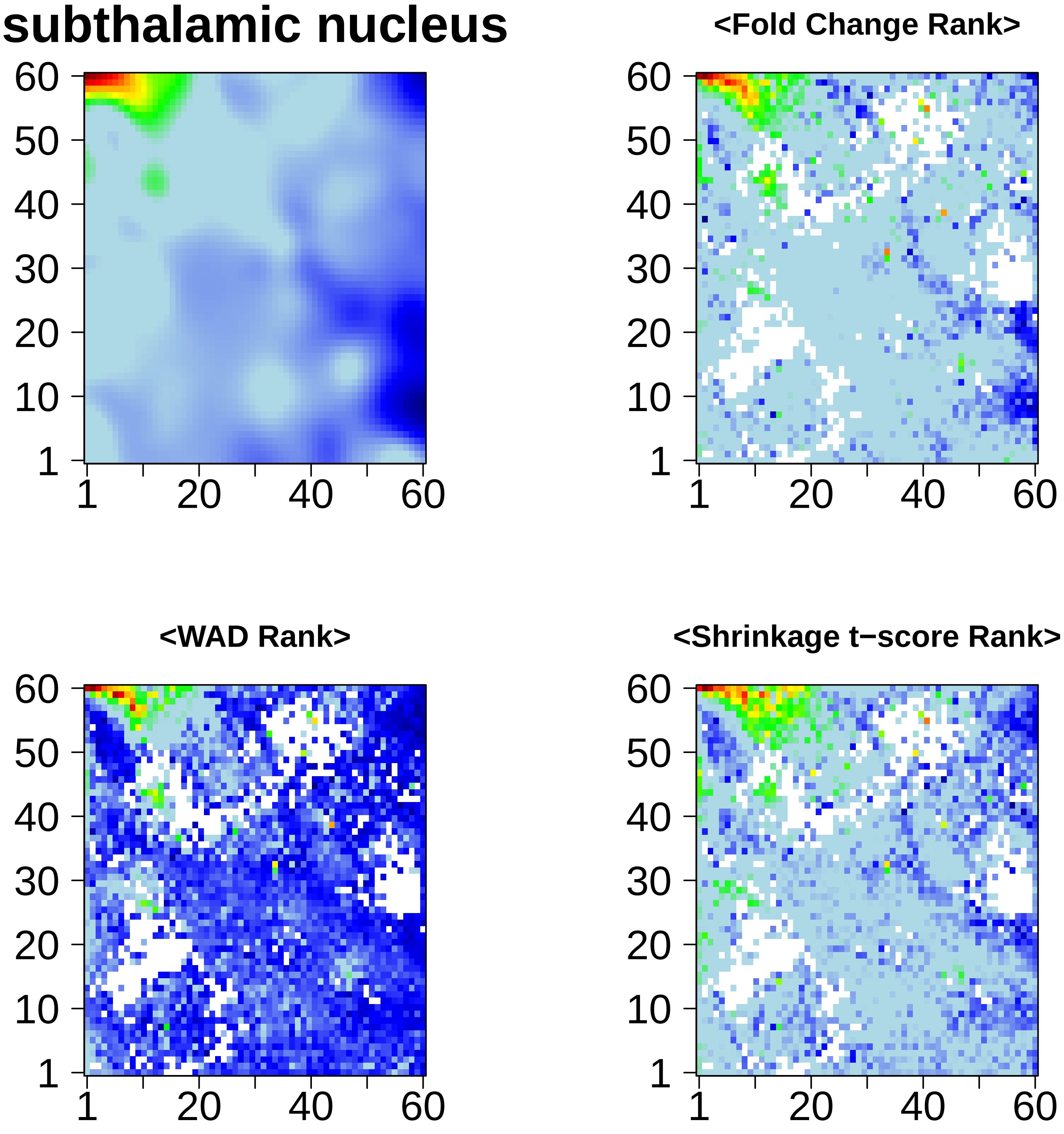

subthalamic nucleus
<Fold Change Rank>
<WAD Rank>
<Shrinkage t−score Rank>
60
50
40
30
20
10
1
1
20
40
60

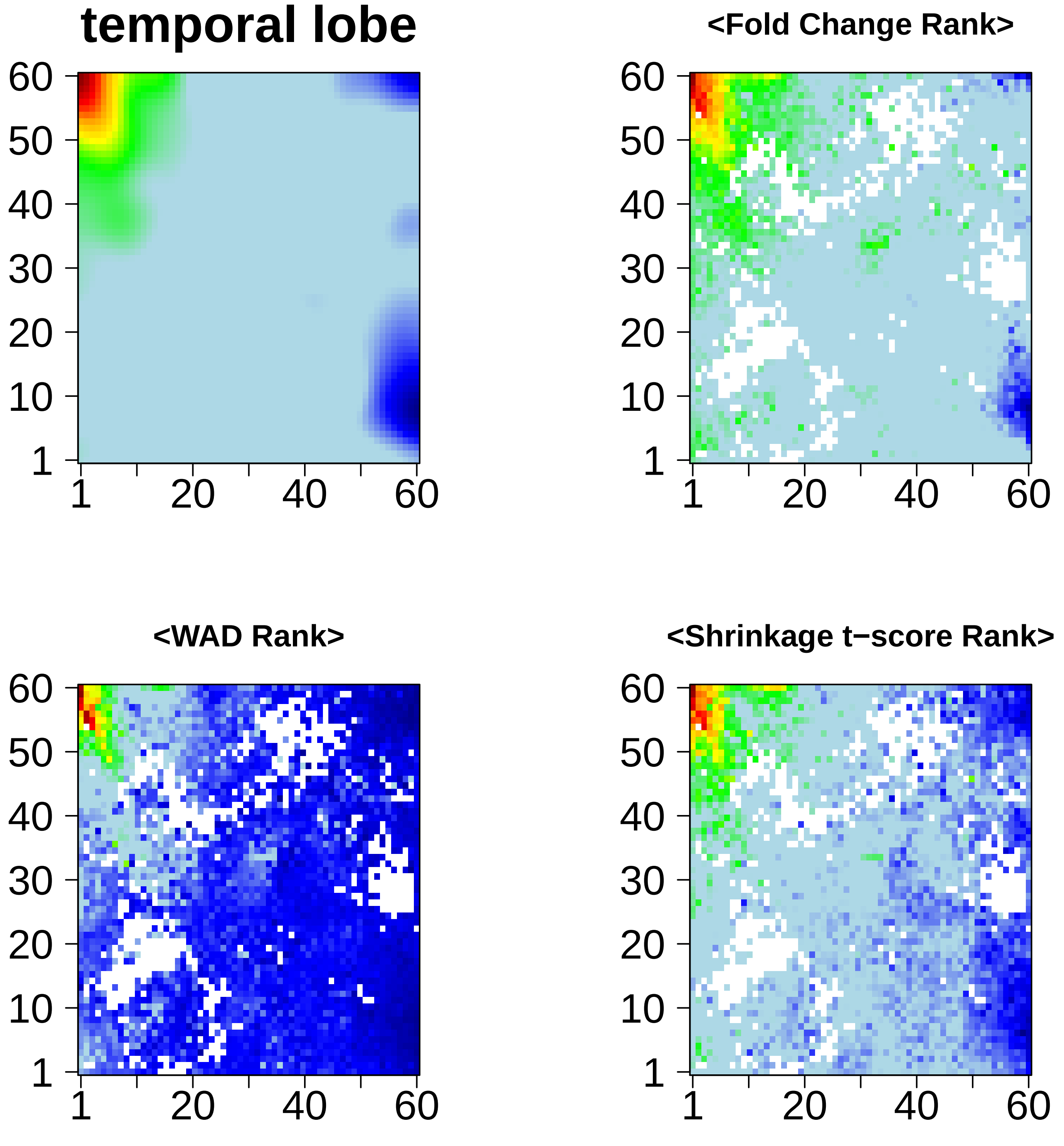
temporal lobe
<Fold Change Rank>
<WAD Rank>
<Shrinkage t−score Rank>
60
50
40
30
20
10
1
1
20
40
60

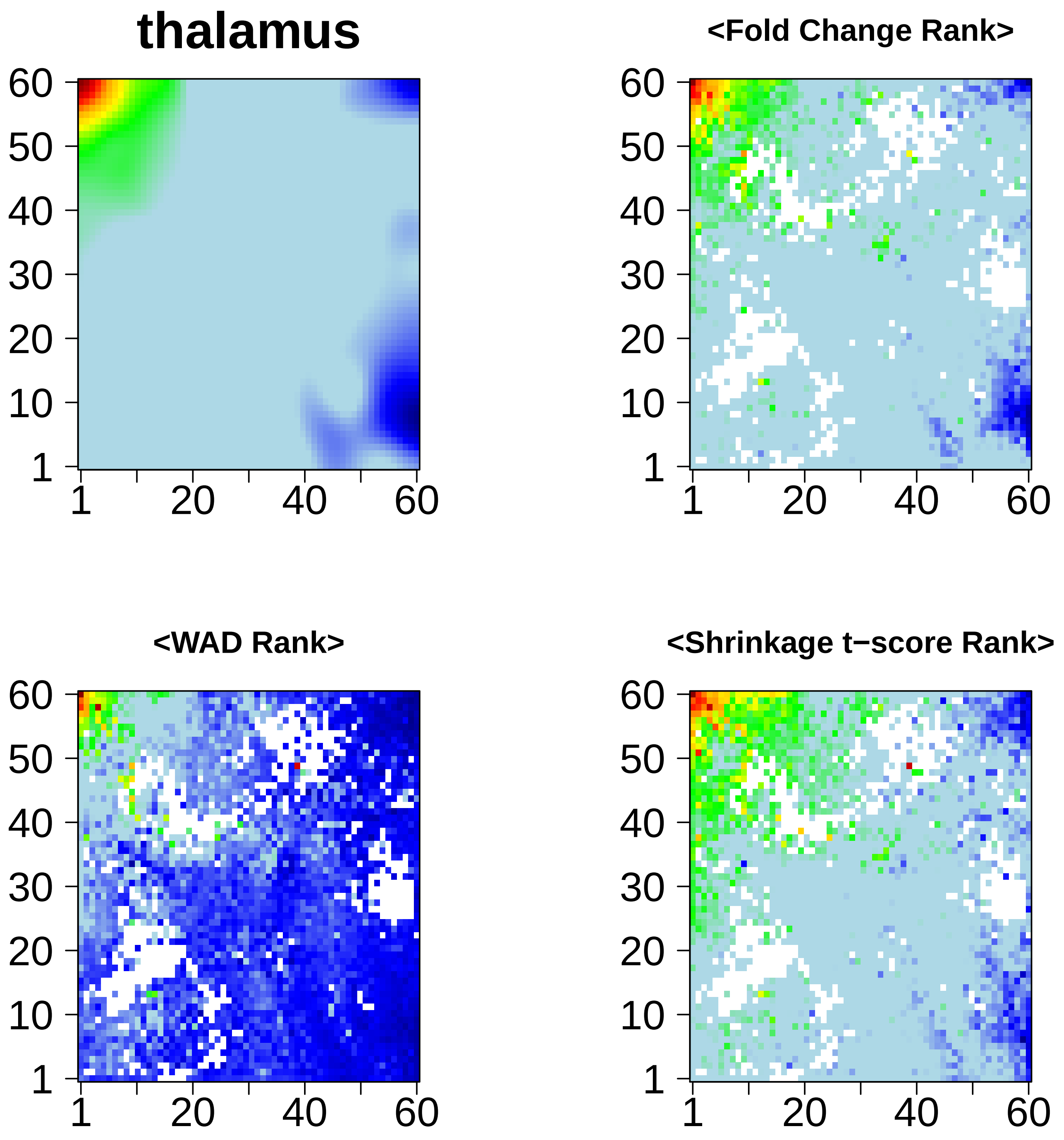
thalamus
<Fold Change Rank>
<WAD Rank>
<Shrinkage t−score Rank>
60
50
40
30
20
10
1
1
20
40
60

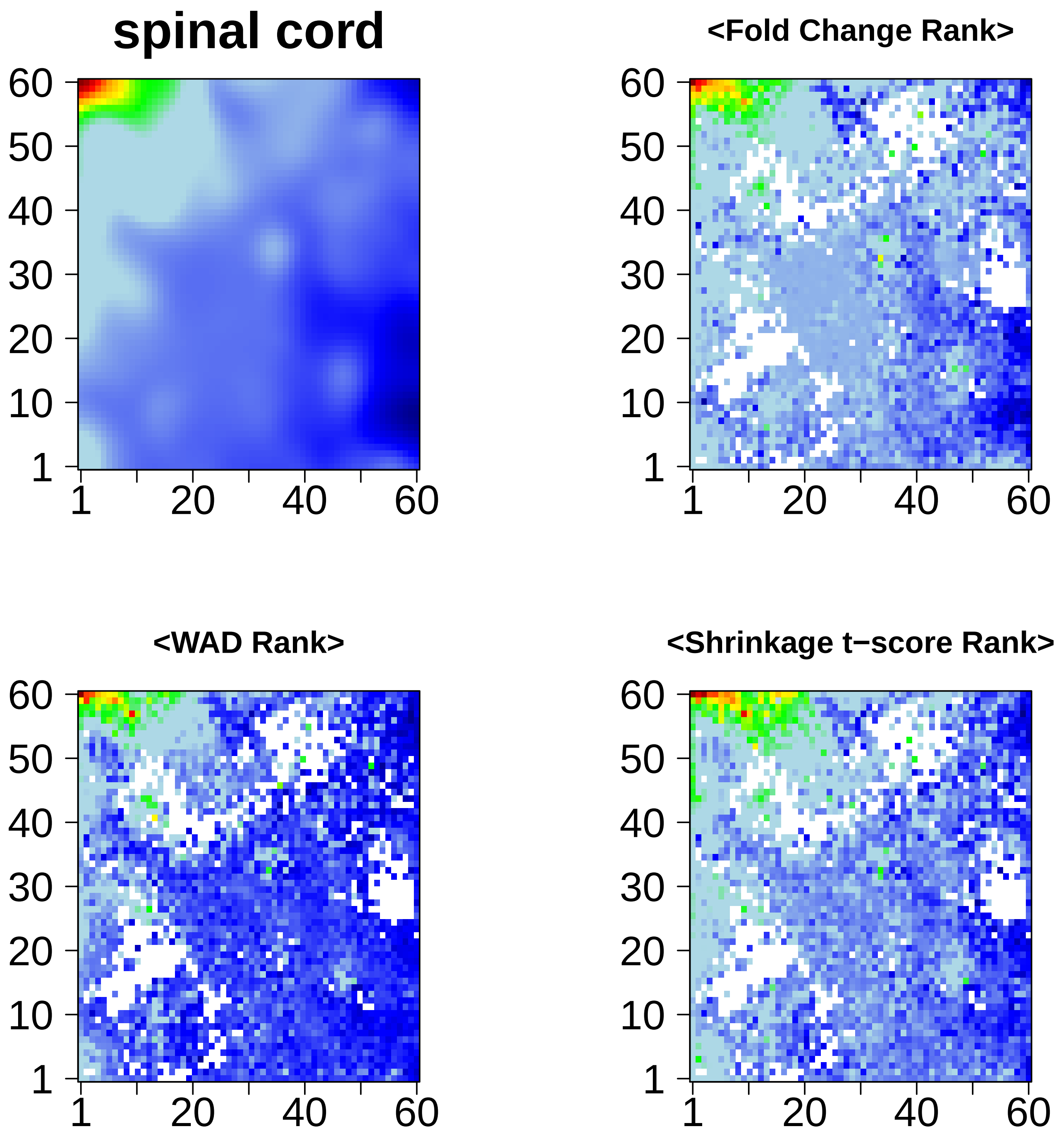

spinal cord
<Fold Change Rank>
<WAD Rank>
<Shrinkage t−score Rank>
60
50
40
30
20
10
1
1
20
40
60
