# Supplementary material for: Mining SOM expression portraits: feature selection and integrating concepts of molecular function
Source: BioData Min. 2012 Oct 8;5:18. doi: 10.1186/1756-0381-5-18 (PMC3599960; doi:10.1186/1756-0381-5-18)
Supplement: Additional file 3 — Atlas of errors and p-value distributions of all tissues studied. [file 1756-0381-5-18-S3.pdf]

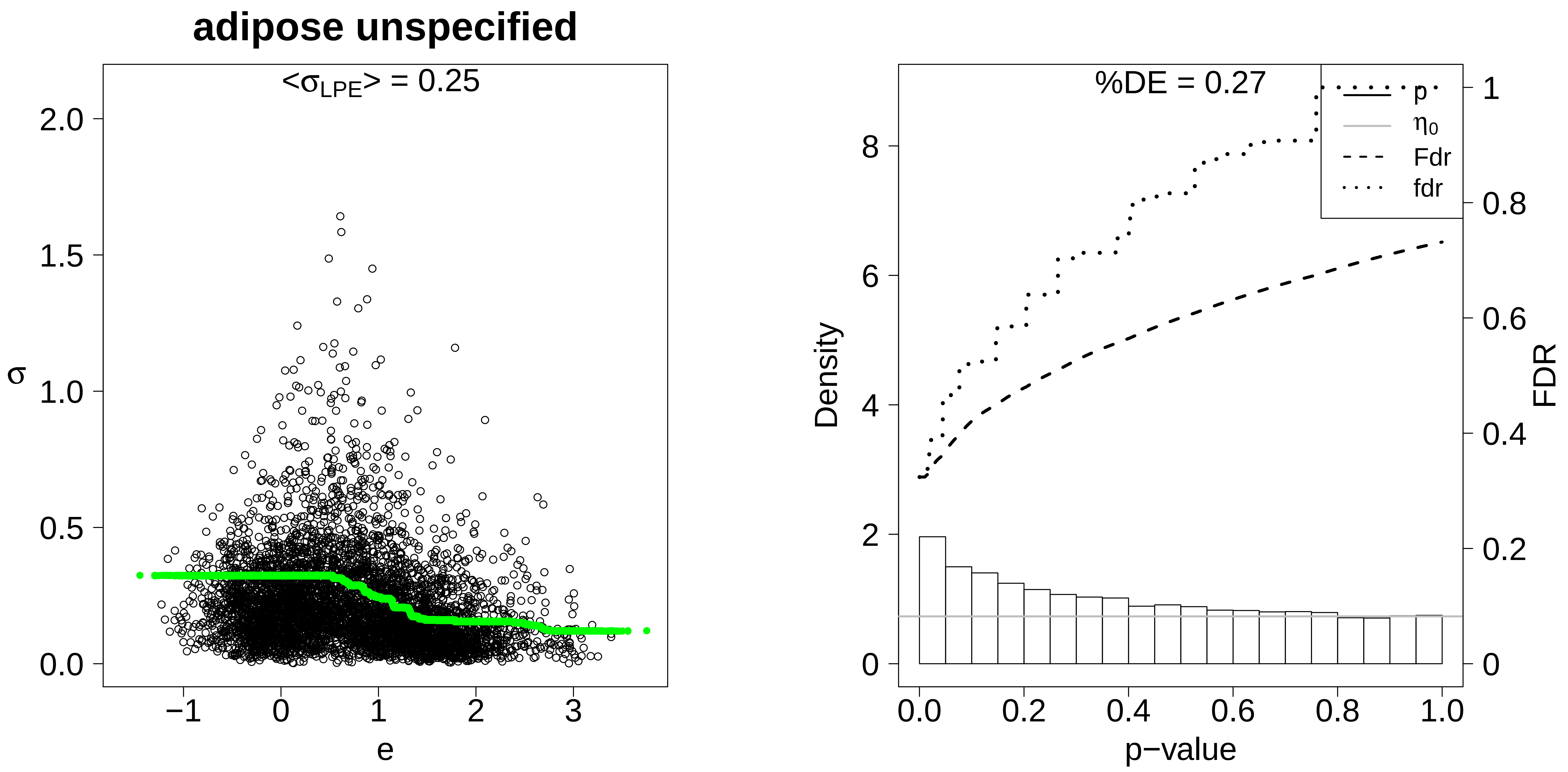

adipose unspecified
<σLPE> = 0.25
σ
e
2.0
1.5
1.0
0.5
0.0
−1
0
1
2
3
%DE = 0.27
Density
p−value
FDR
p
η0
Fdr
fdr
8
6
4
2
0
0.0
0.2
0.4
0.6
0.8
1.0
1
0.8
0.6
0.4
0.2
0

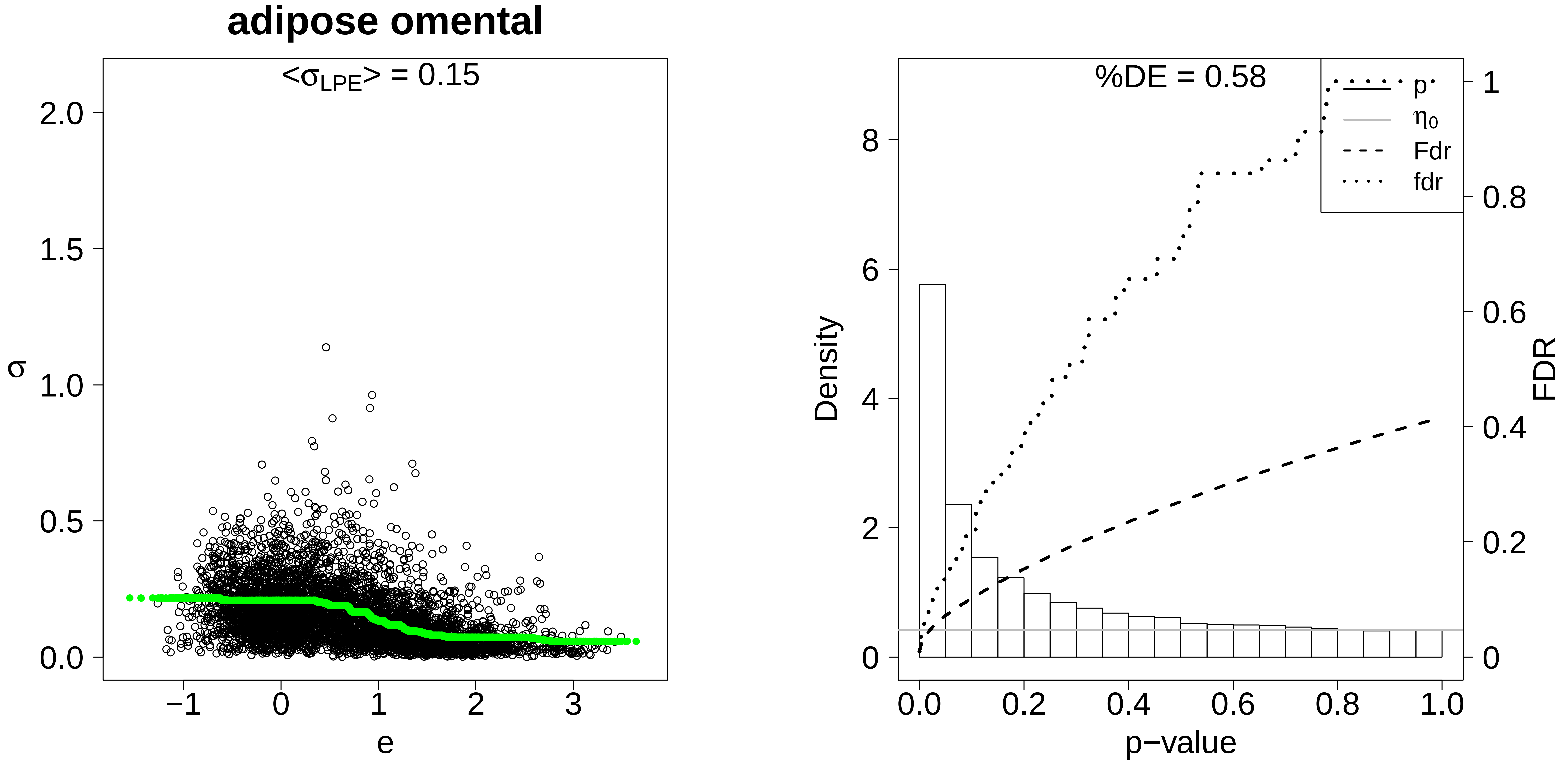

adipose omental
<σLPE> = 0.15
σ
e
%DE = 0.58
p
η0
Fdr
fdr
Density
p−value
FDR

adipose subcutaneous

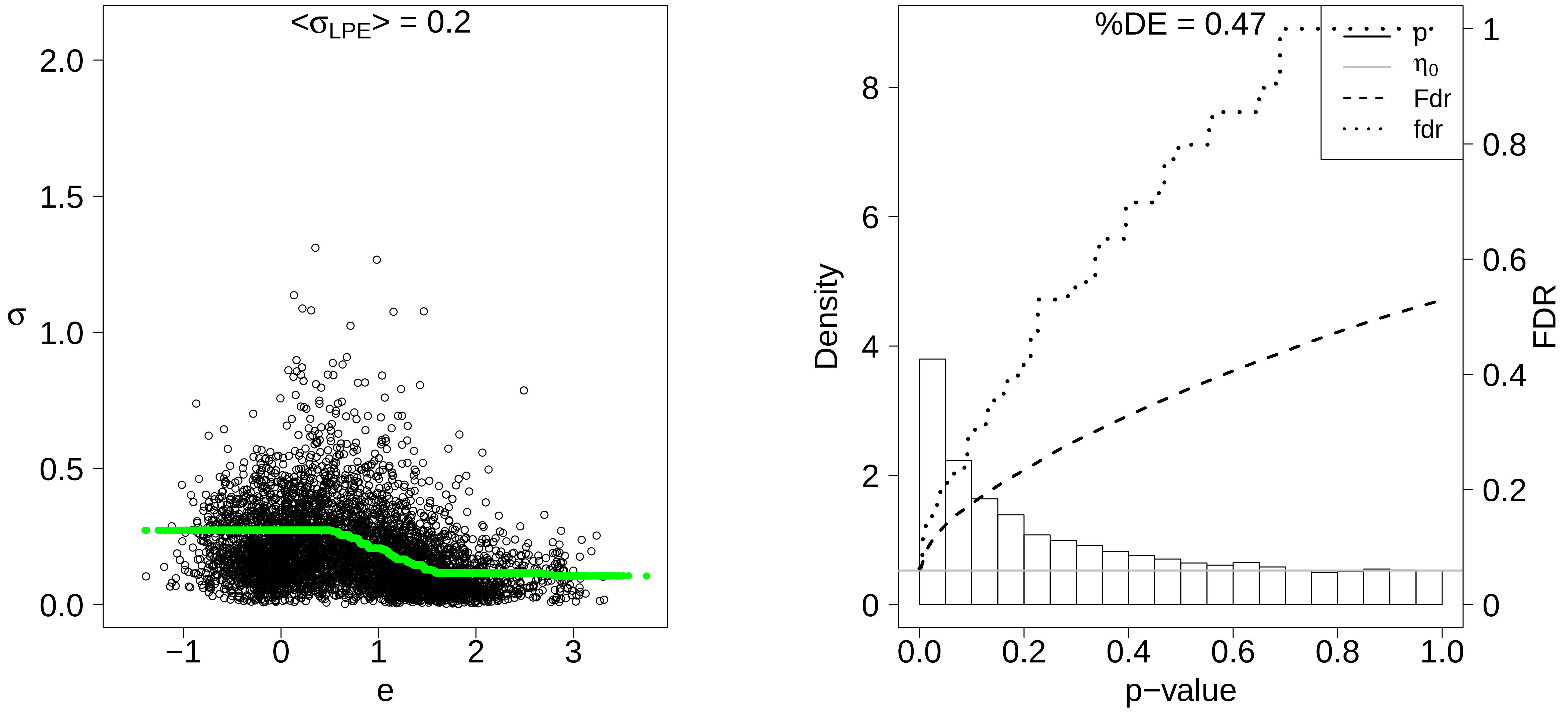

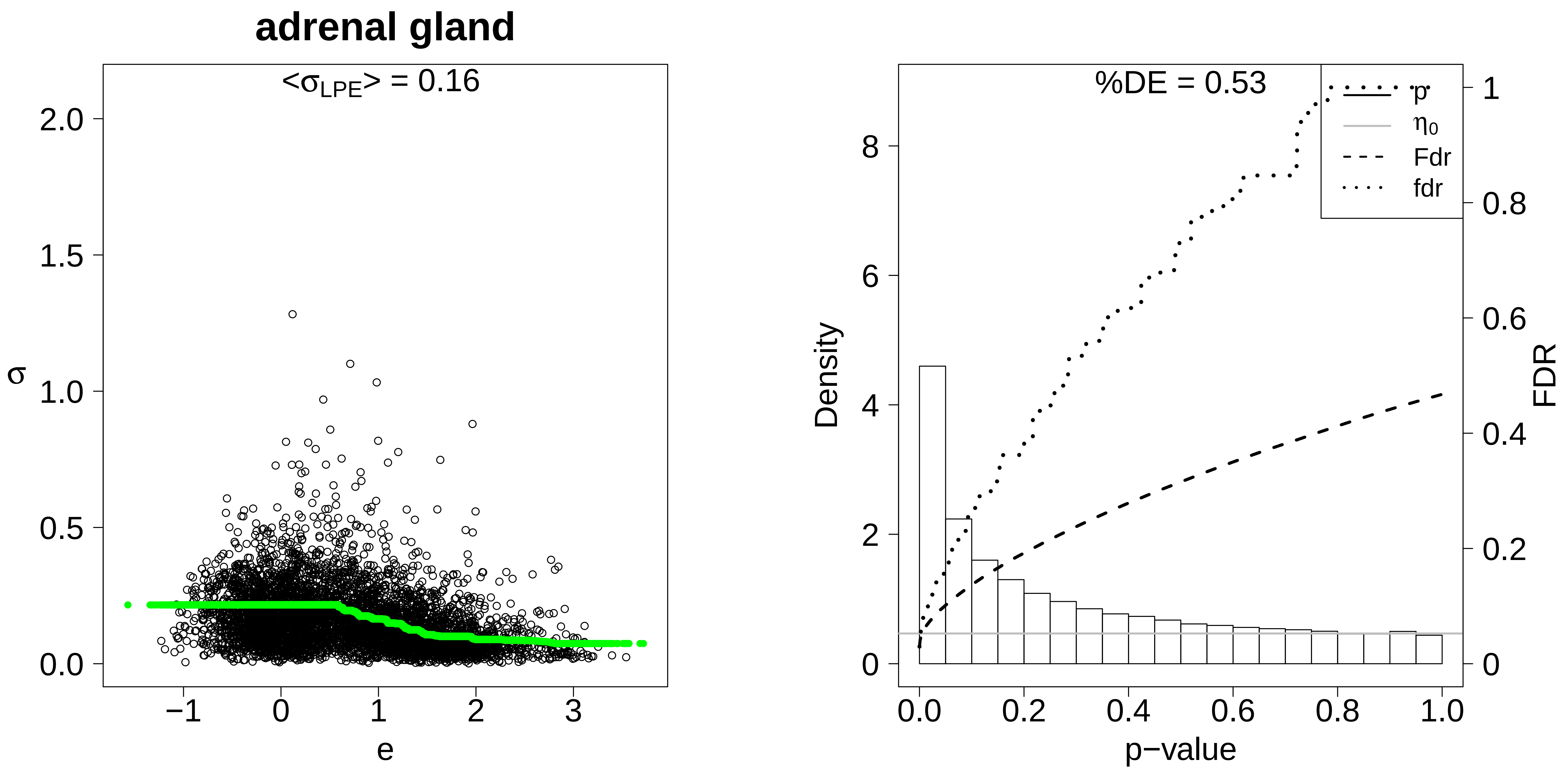

adrenal gland
<σ_LPE> = 0.16
σ
e
2.0
1.5
1.0
0.5
0.0
−1
0
1
2
3
%DE = 0.53
p
η0
Fdr
fdr
Density
FDR
p−value
8
6
4
2
0
0.0
0.2
0.4
0.6
0.8
1.0
1
0.8
0.6
0.4
0.2
0

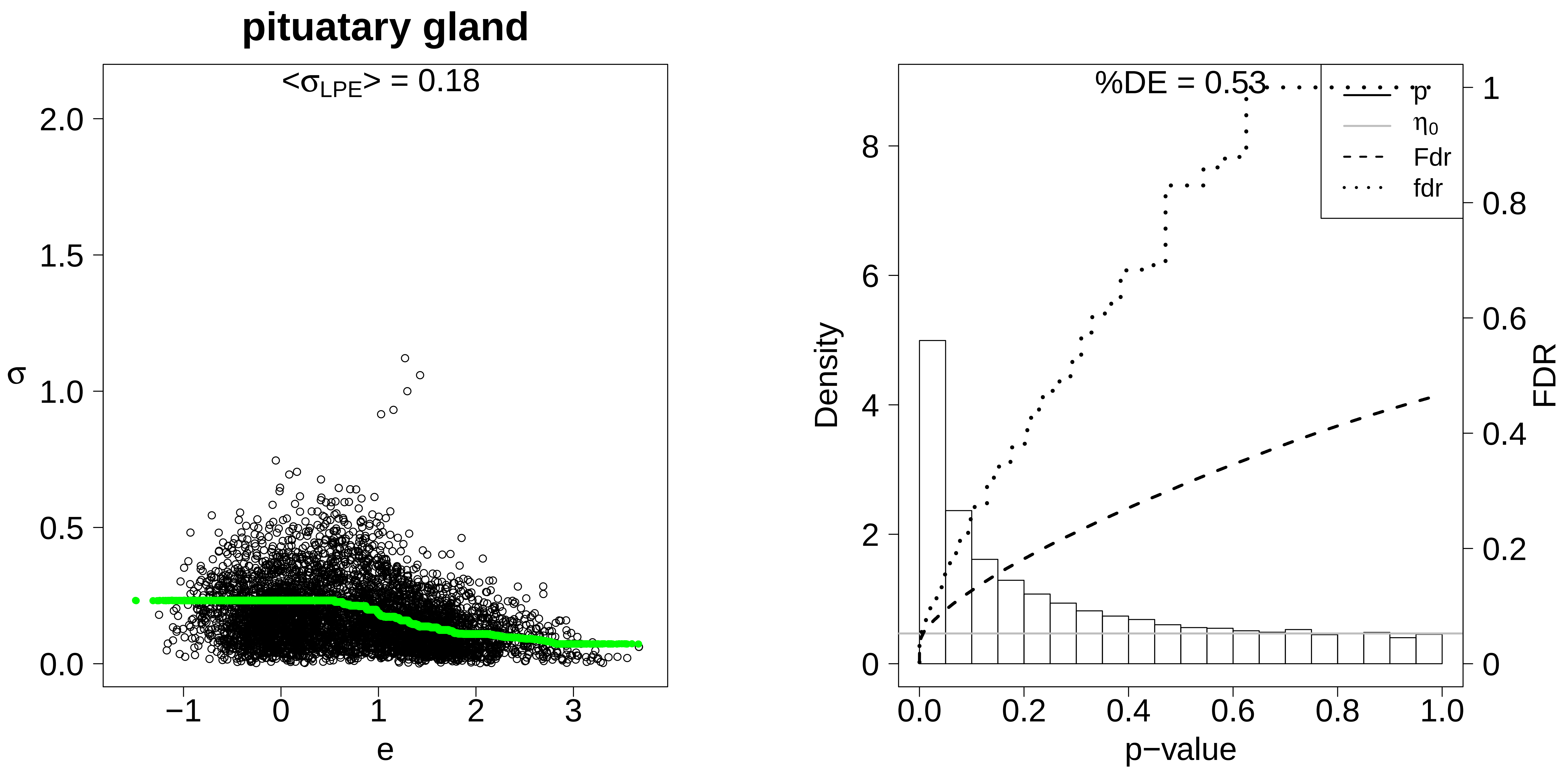

pituatary gland
<σLPE> = 0.18
σ
e
%DE = 0.53
p
η0
Fdr
fdr
Density
FDR
p−value

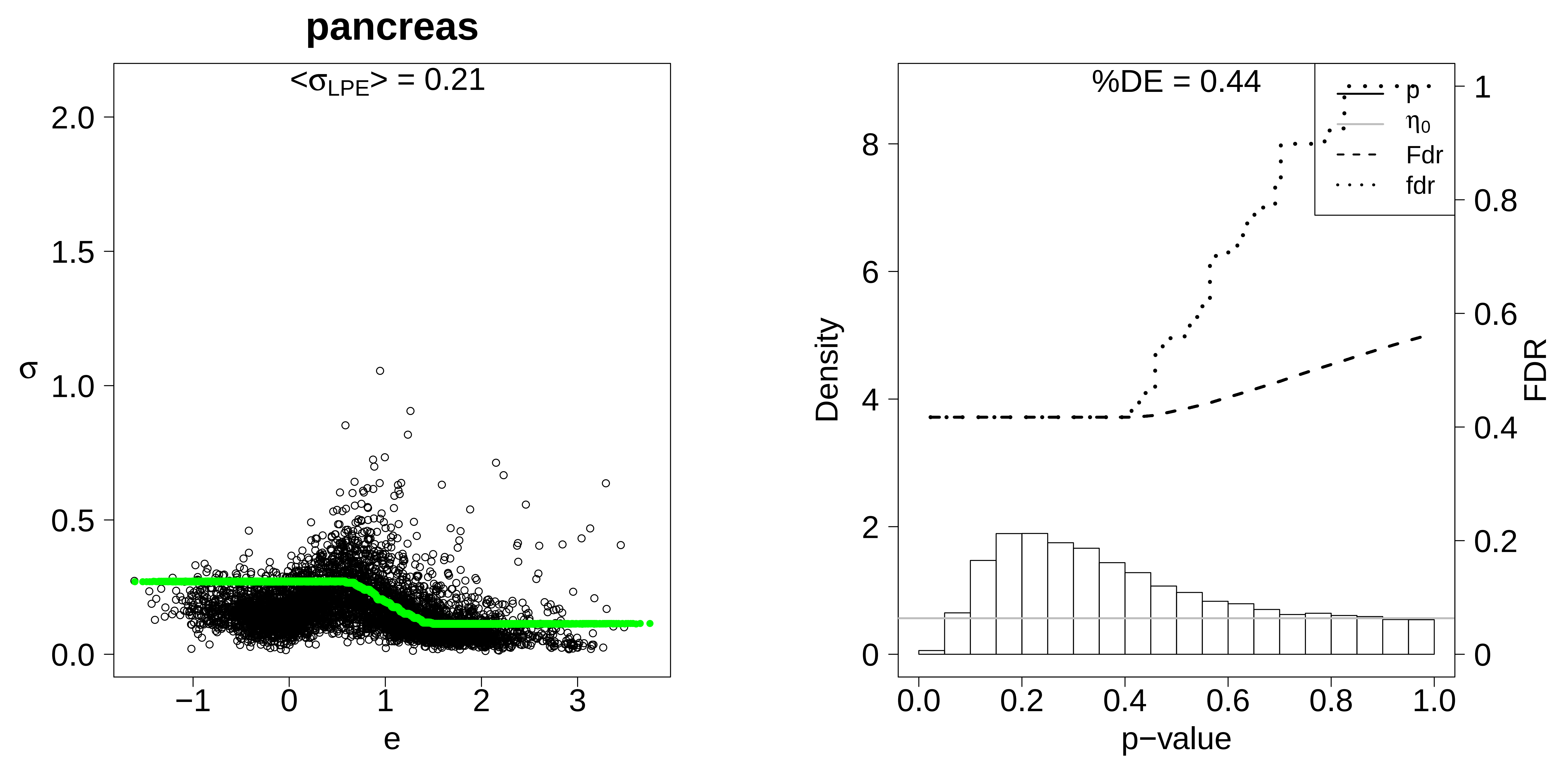
pancreas
<σLPE> = 0.21
σ
e
%DE = 0.44
p
η0
Fdr
fdr
Density
FDR
p−value

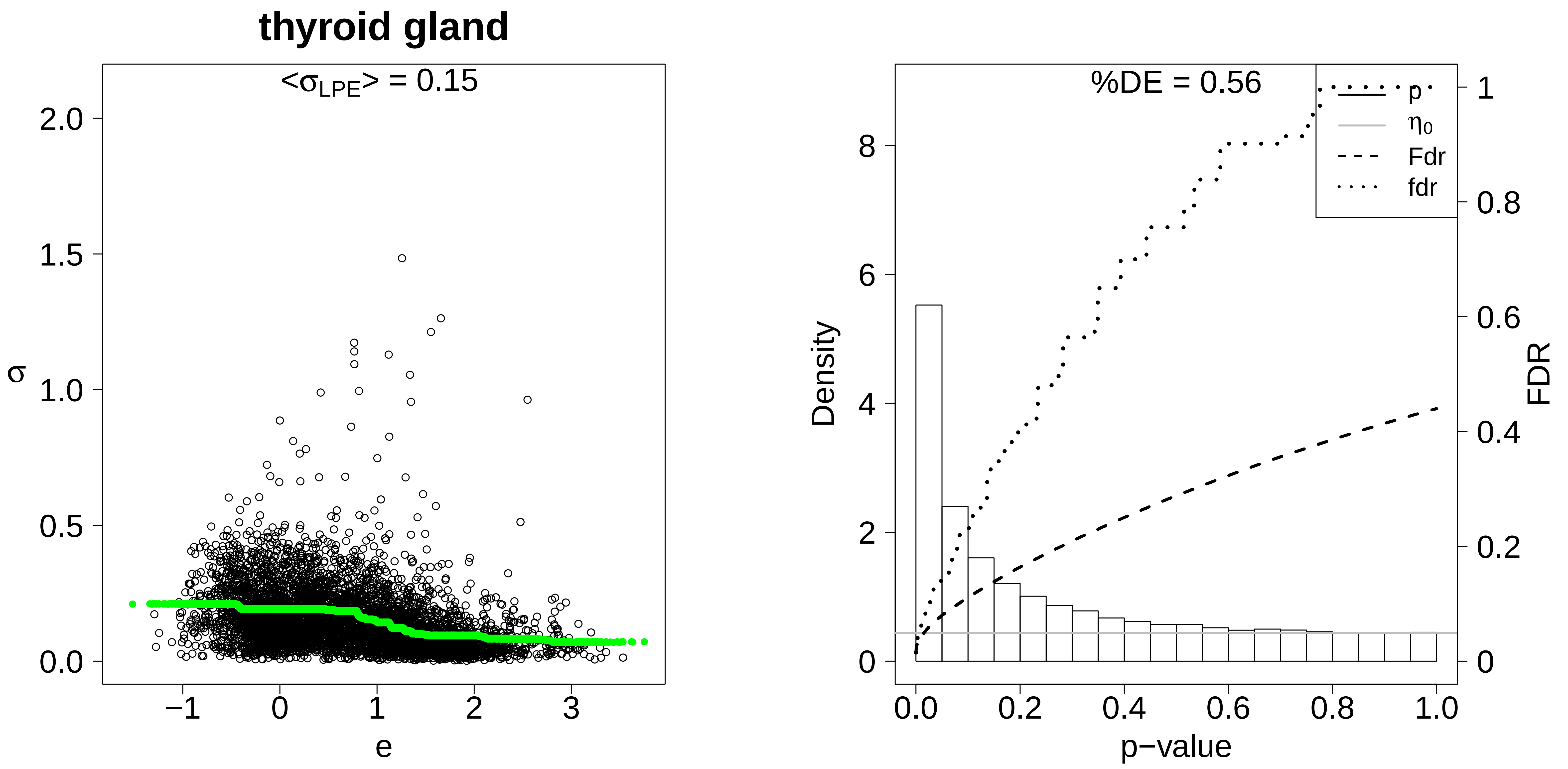
thyroid gland
<σLPE> = 0.15
σ
e
2.0
1.5
1.0
0.5
0.0
−1
0
1
2
3
%DE = 0.56
Density
p−value
FDR
p
η0
Fdr
fdr
0
2
4
6
8
0.0
0.2
0.4
0.6
0.8
1.0
0
0.2
0.4
0.6
0.8
1

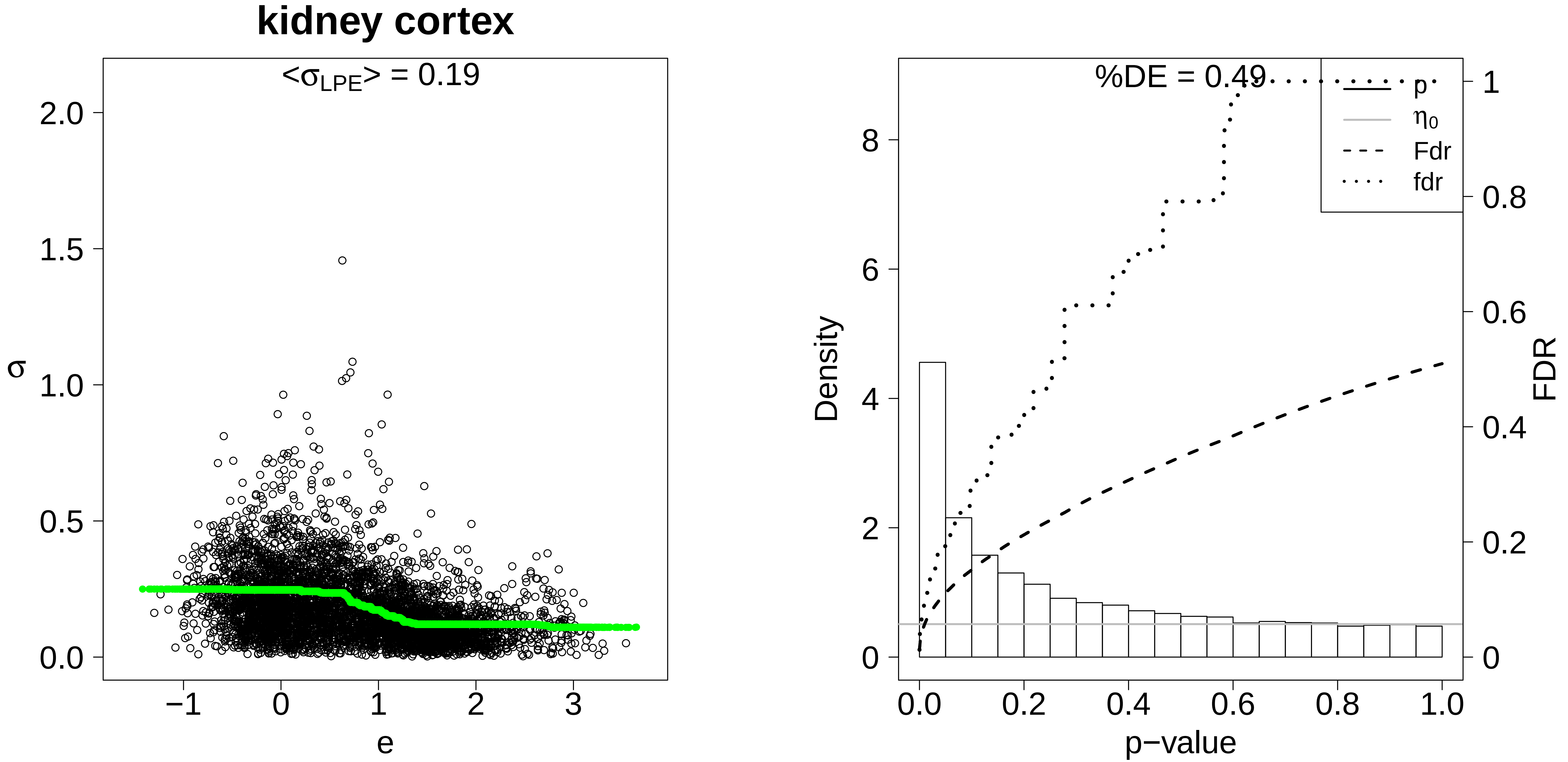

kidney cortex
<σLPE> = 0.19
σ
e
%DE = 0.49
p
η0
Fdr
fdr
Density
FDR
p−value

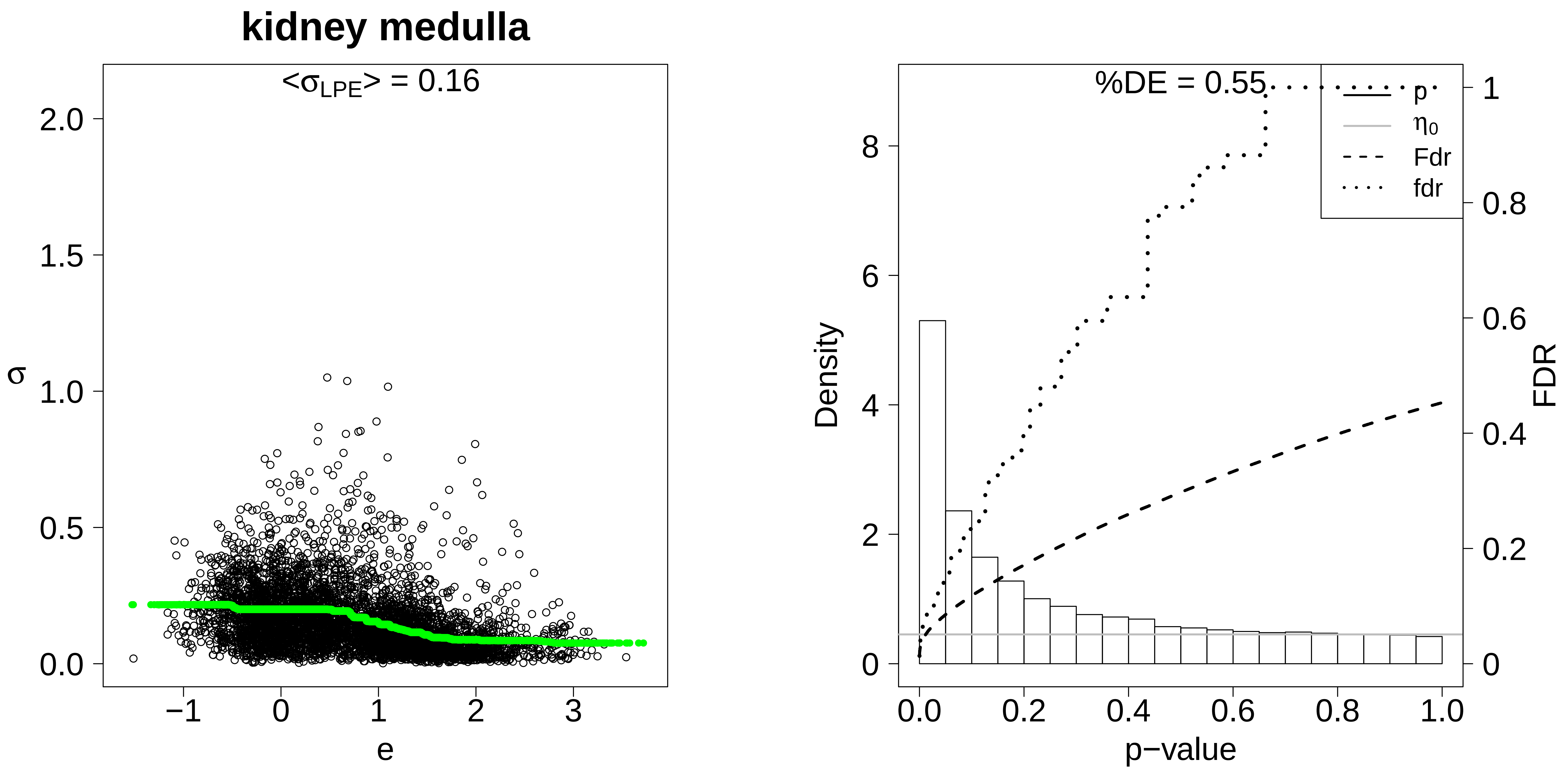

kidney medulla
<σ_LPE> = 0.16
σ
e
%DE = 0.55
p
η0
Fdr
fdr
Density
FDR
p−value

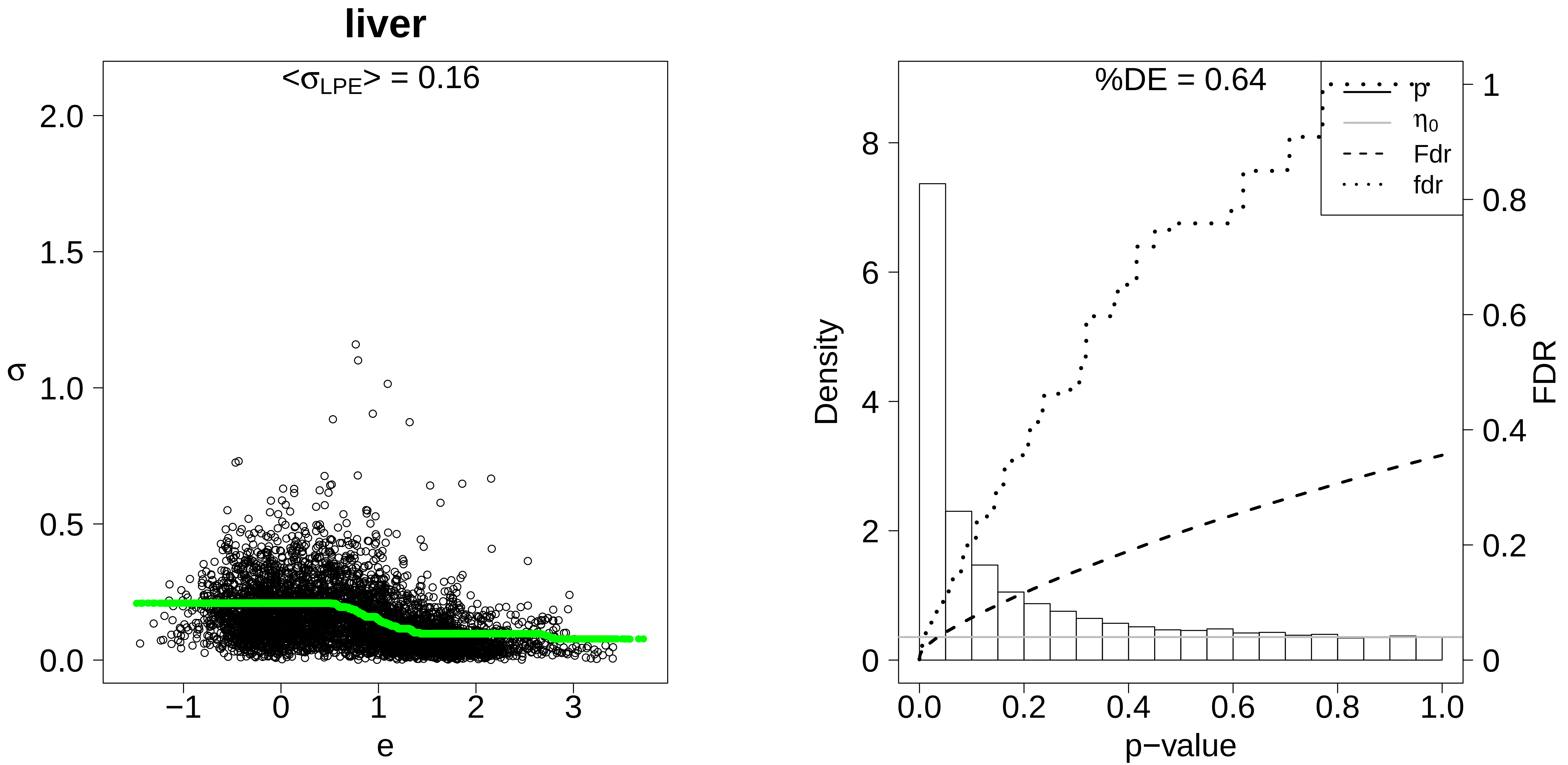
liver
<σLPE> = 0.16
σ
e
2.0
1.5
1.0
0.5
0.0
−1
0
1
2
3
%DE = 0.64
p
η0
Fdr
fdr
Density
FDR
p−value
8
6
4
2
0
0.0
0.2
0.4
0.6
0.8
1.0
1
0.8
0.6
0.4
0.2
0

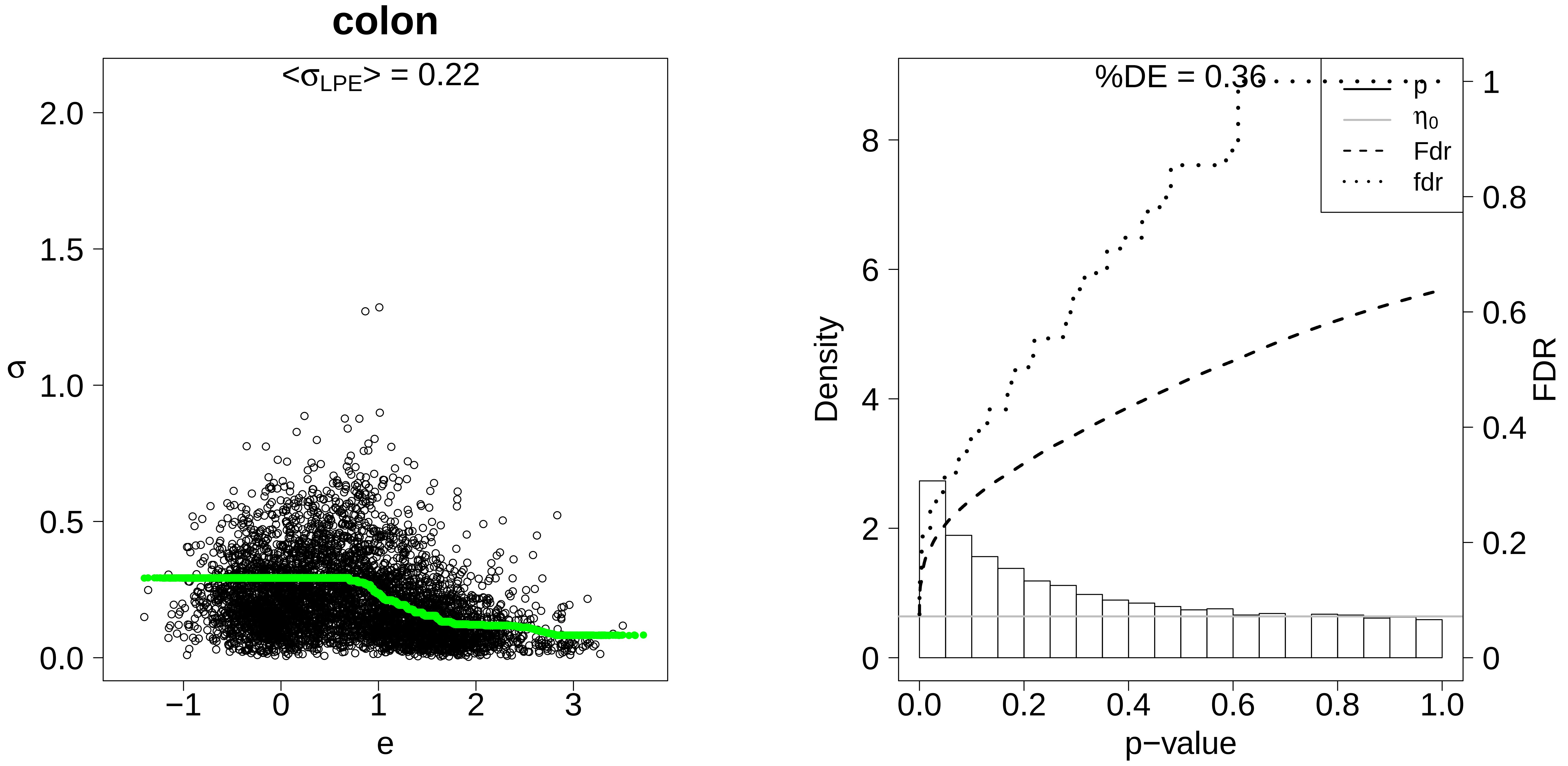
colon
<σLPE> = 0.22
σ
e
2.0
1.5
1.0
0.5
0.0
−1
0
1
2
3
%DE = 0.36
p
η0
Fdr
fdr
Density
p−value
FDR
8
6
4
2
0
0.0
0.2
0.4
0.6
0.8
1.0
1
0.8
0.6
0.4
0.2
0

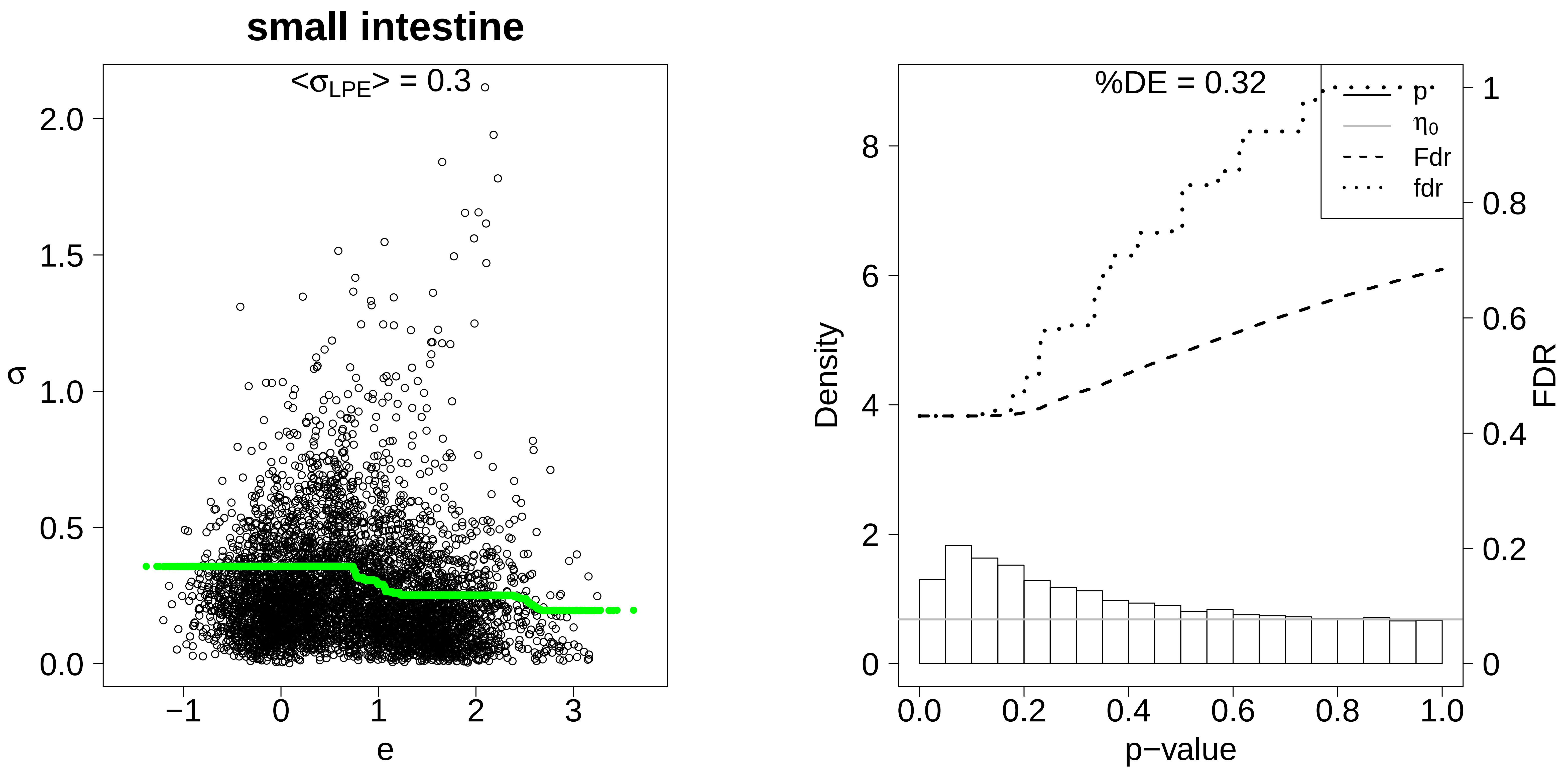

small intestine
<σ_LPE> = 0.3
σ
e
%DE = 0.32
p
η0
Fdr
fdr
Density
FDR
p−value

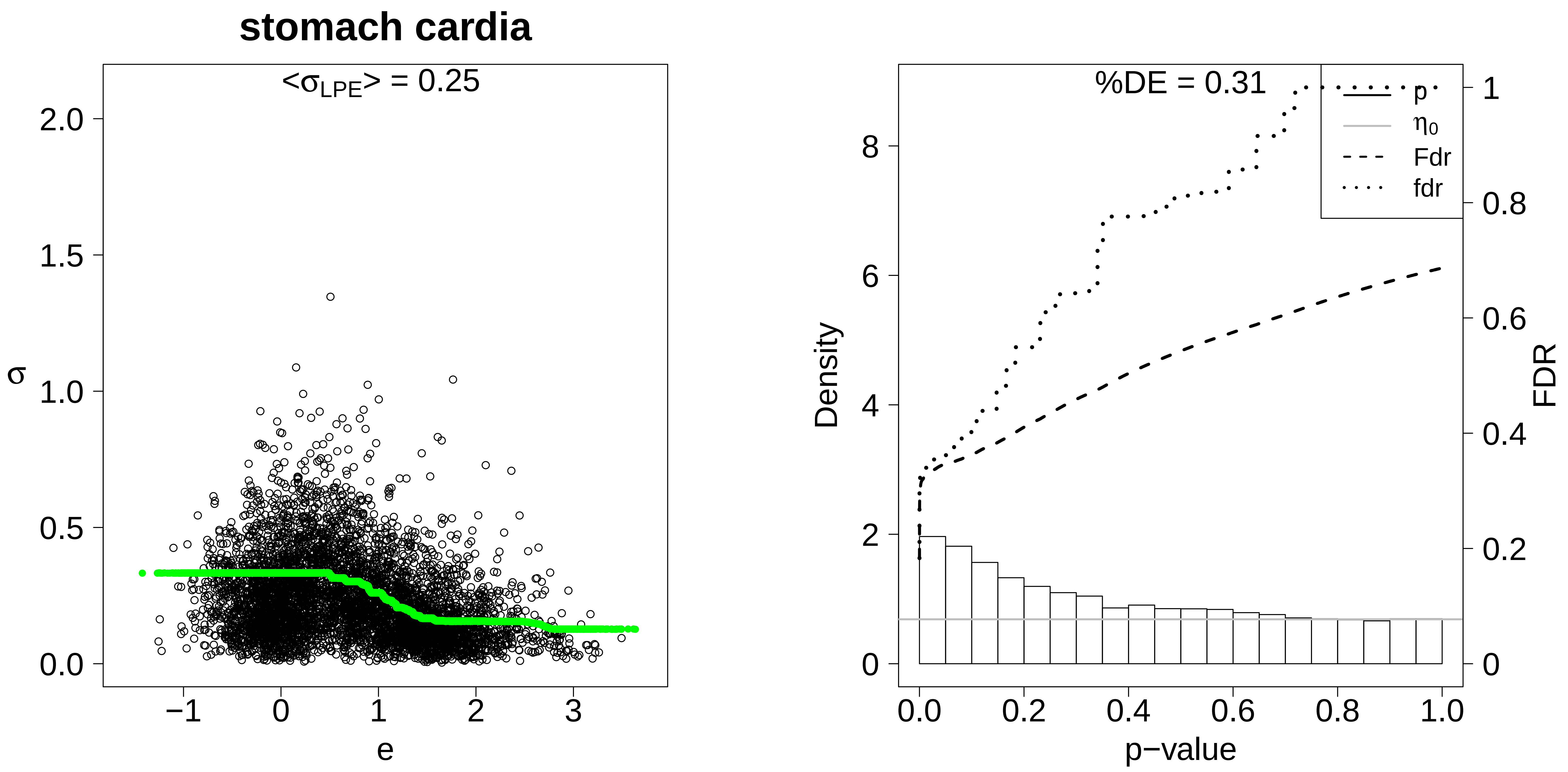
stomach cardia
<σLPE> = 0.25
σ
e
%DE = 0.31
p
η0
Fdr
fdr
Density
FDR
p−value

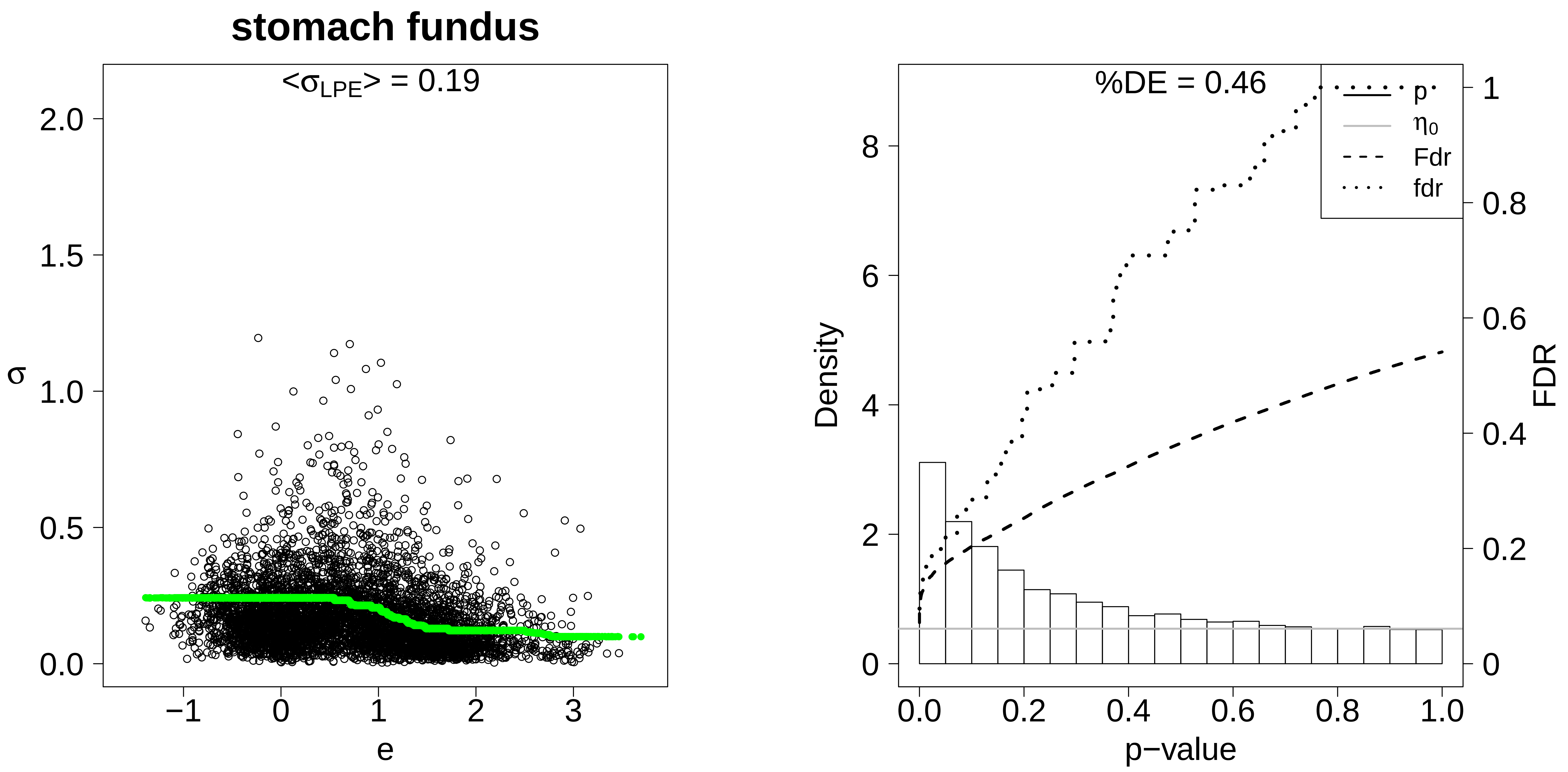

stomach fundus
<σLPE> = 0.19
2.0
1.5
1.0
0.5
0.0
σ
−1
0
1
2
3
e
%DE = 0.46
p
η0
Fdr
fdr
8
6
4
2
0
Density
1
0.8
0.6
0.4
0.2
0
FDR
0.0
0.2
0.4
0.6
0.8
1.0
p−value

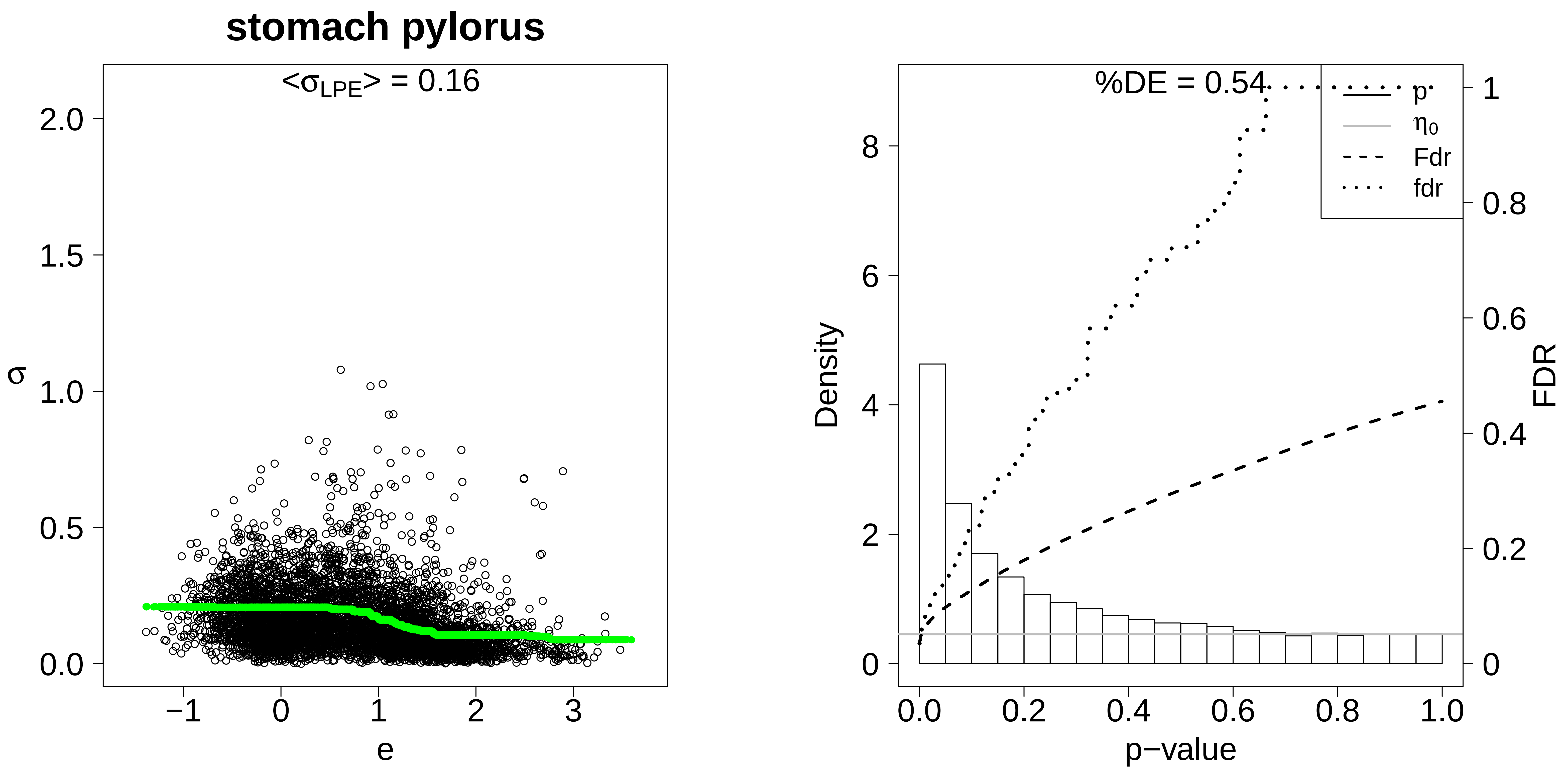
stomach pylorus
<σLPE> = 0.16
σ
e
2.0
1.5
1.0
0.5
0.0
−1
0
1
2
3
%DE = 0.54
p
η0
Fdr
fdr
Density
FDR
p−value
8
6
4
2
0
0.0
0.2
0.4
0.6
0.8
1.0
1
0.8
0.6
0.4
0.2
0

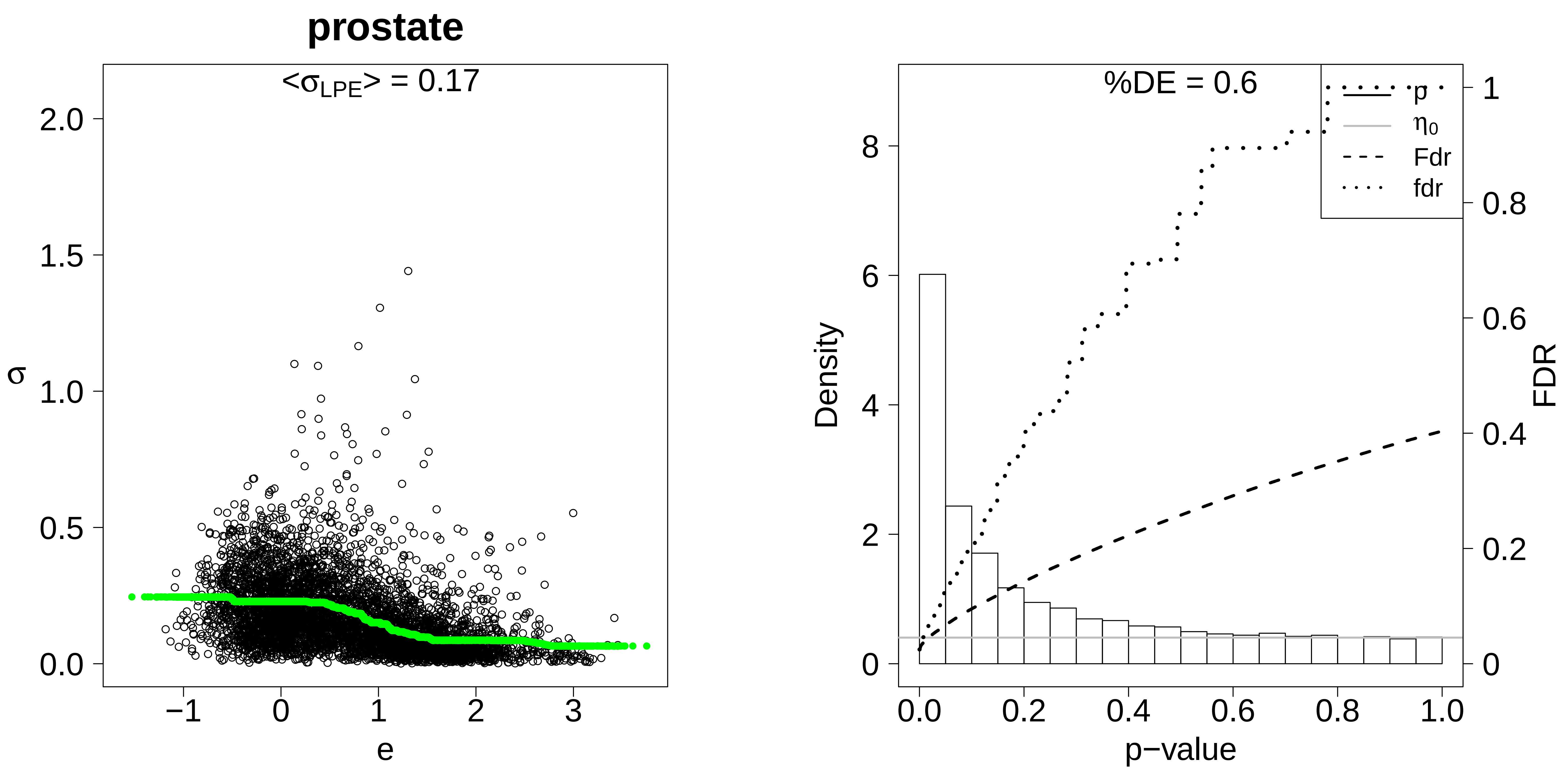

prostate
<σLPE> = 0.17
σ
e
%DE = 0.6
p
η0
Fdr
fdr
Density
FDR
p−value

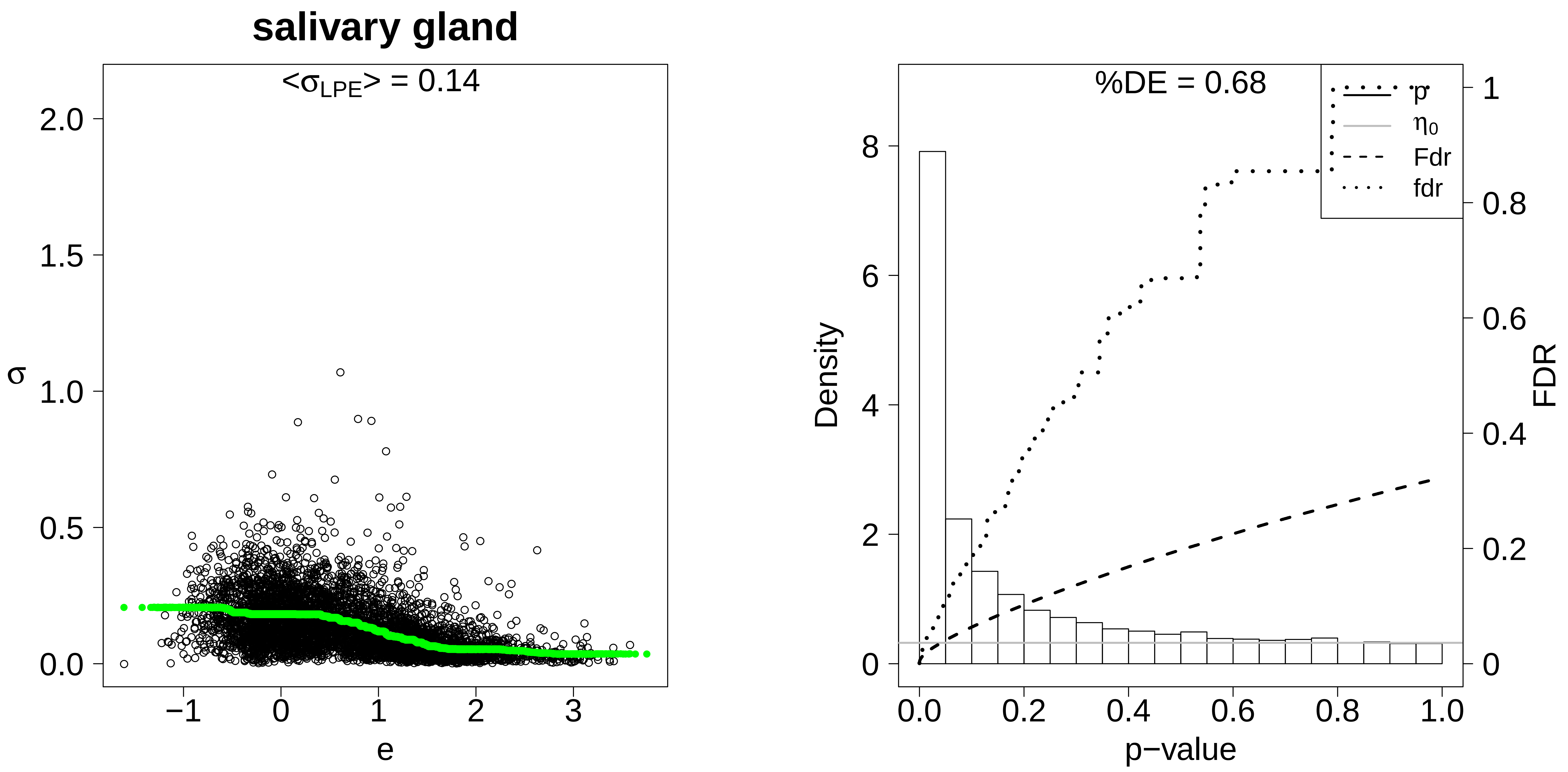
salivary gland
<σLPE> = 0.14
σ
e
%DE = 0.68
p
η0
Fdr
fdr
Density
FDR
p−value

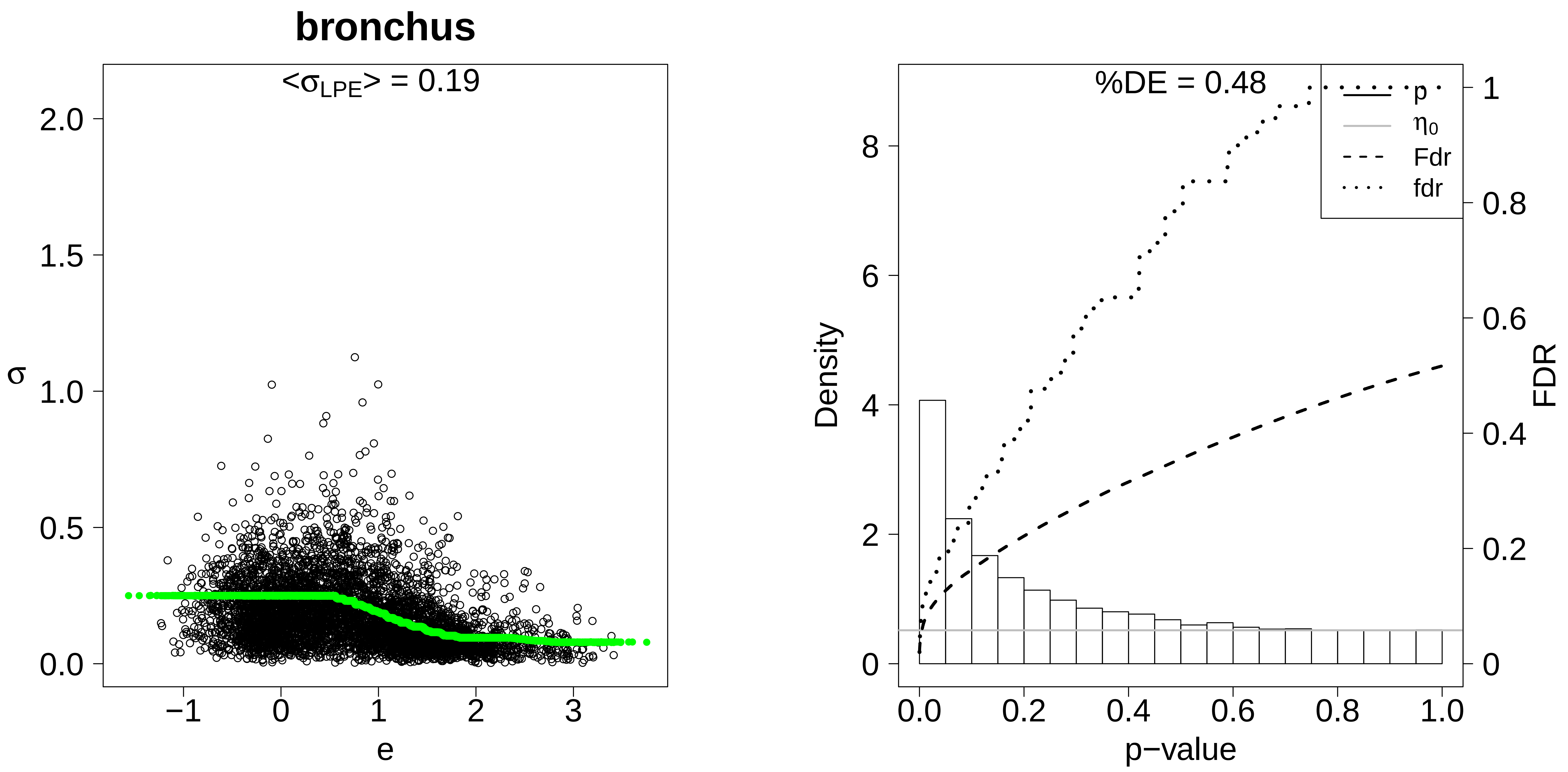

bronchus
<σLPE> = 0.19
σ
e
%DE = 0.48
p
η0
Fdr
fdr
Density
p−value
FDR

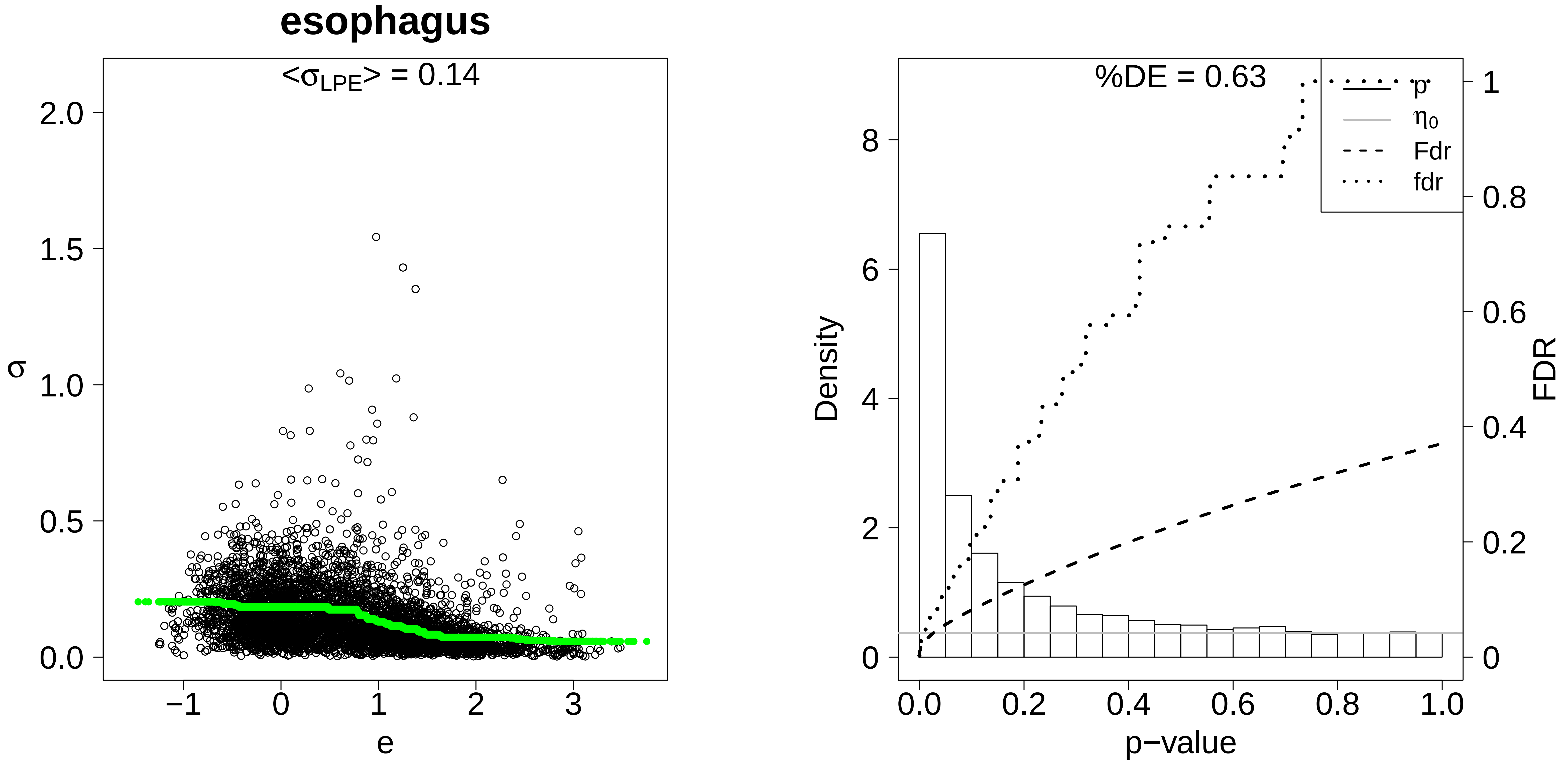

esophagus
<σLPE> = 0.14
σ
e
%DE = 0.63
p
η0
Fdr
fdr
Density
FDR
p−value

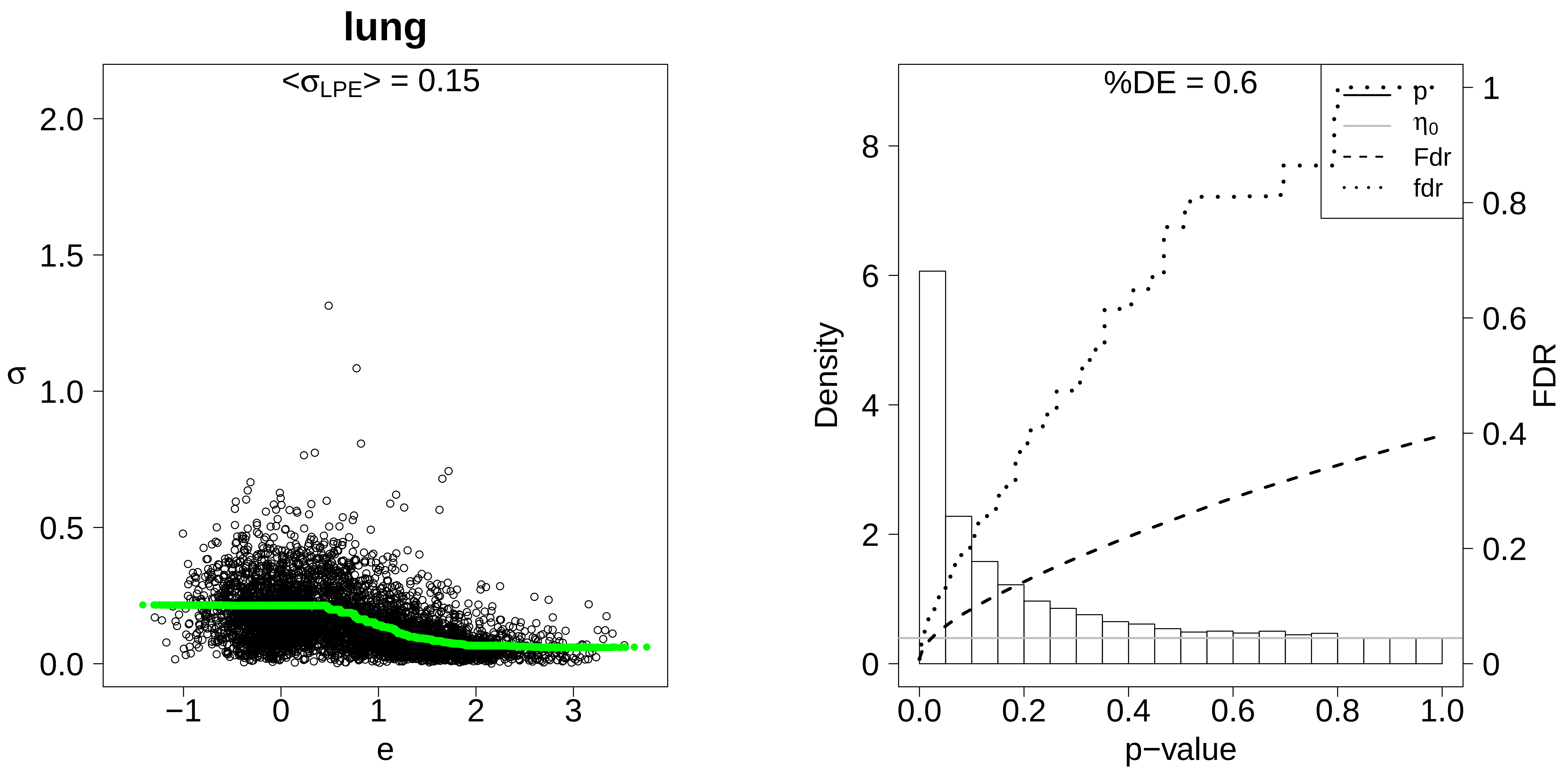

lung
<σ_LPE> = 0.15
σ
e
2.0
1.5
1.0
0.5
0.0
−1
0
1
2
3
%DE = 0.6
p·
η0
Fdr
fdr
Density
FDR
p−value
8
6
4
2
0
0.0
0.2
0.4
0.6
0.8
1.0
1
0.8
0.6
0.4
0.2
0

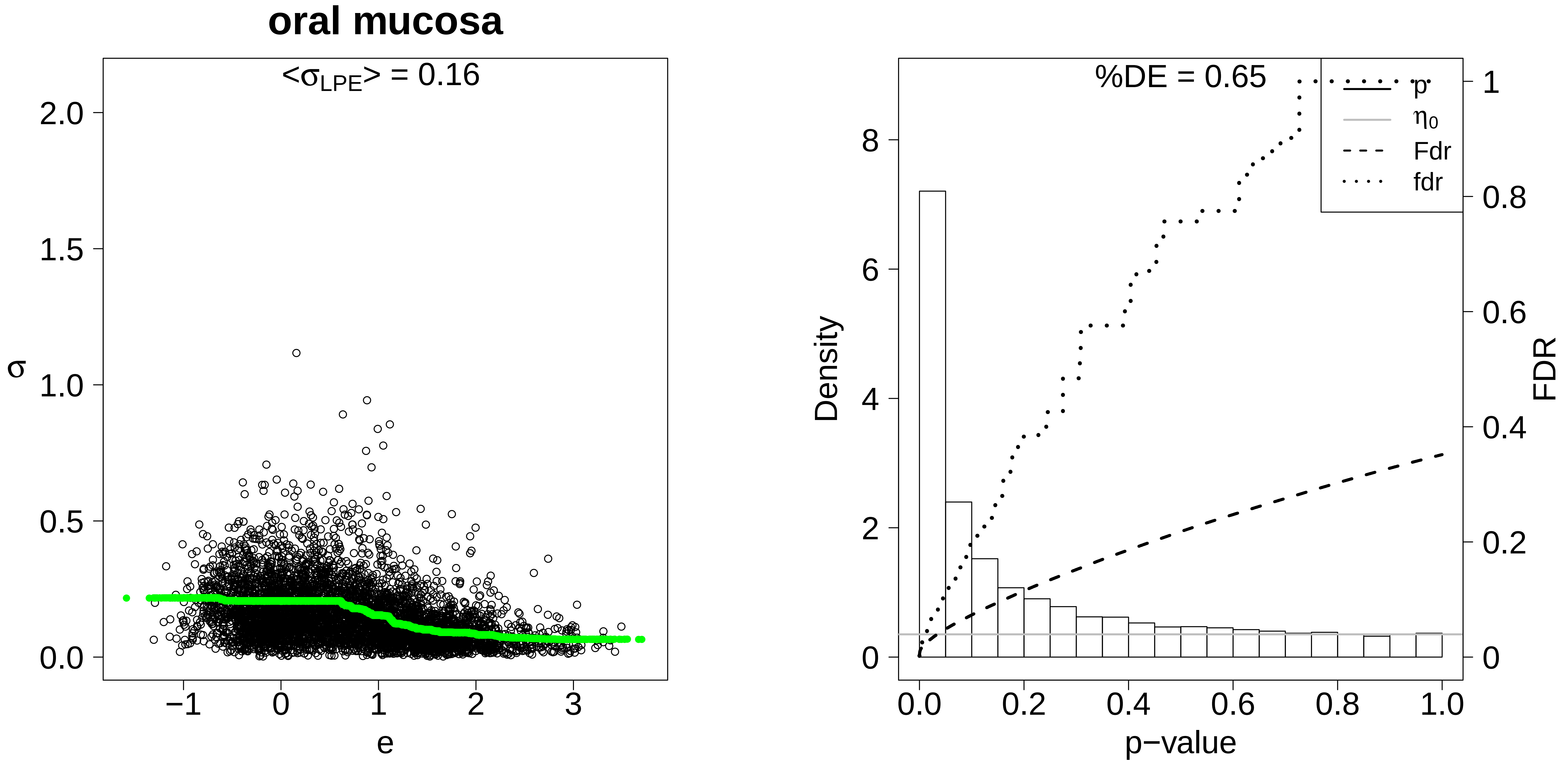

oral mucosa
<σLPE> = 0.16
σ
e
%DE = 0.65
p
η0
Fdr
fdr
Density
FDR
p−value

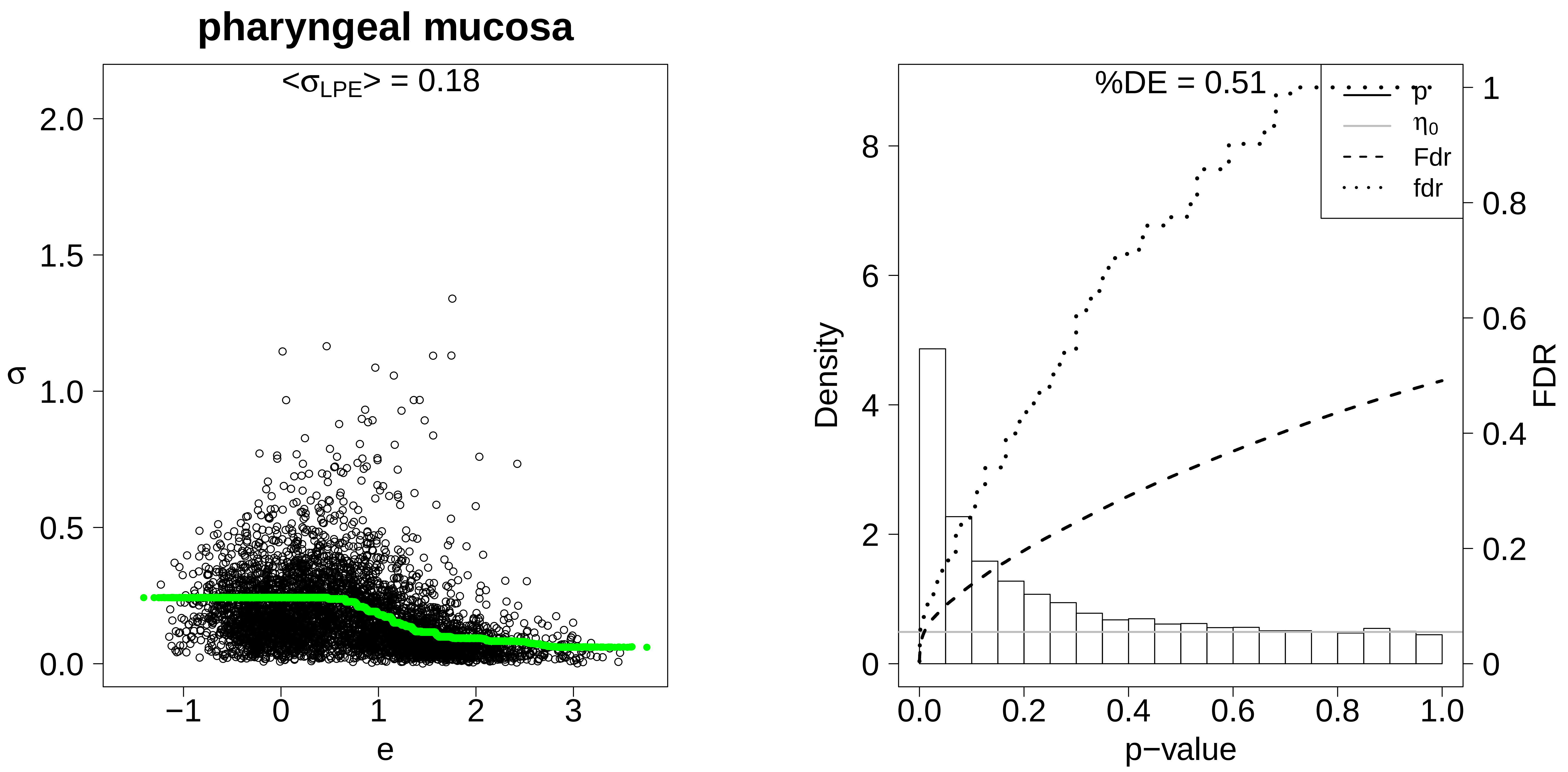

pharyngeal mucosa
<σLPE> = 0.18
σ
e
%DE = 0.51
p
η0
Fdr
fdr
Density
FDR
p−value

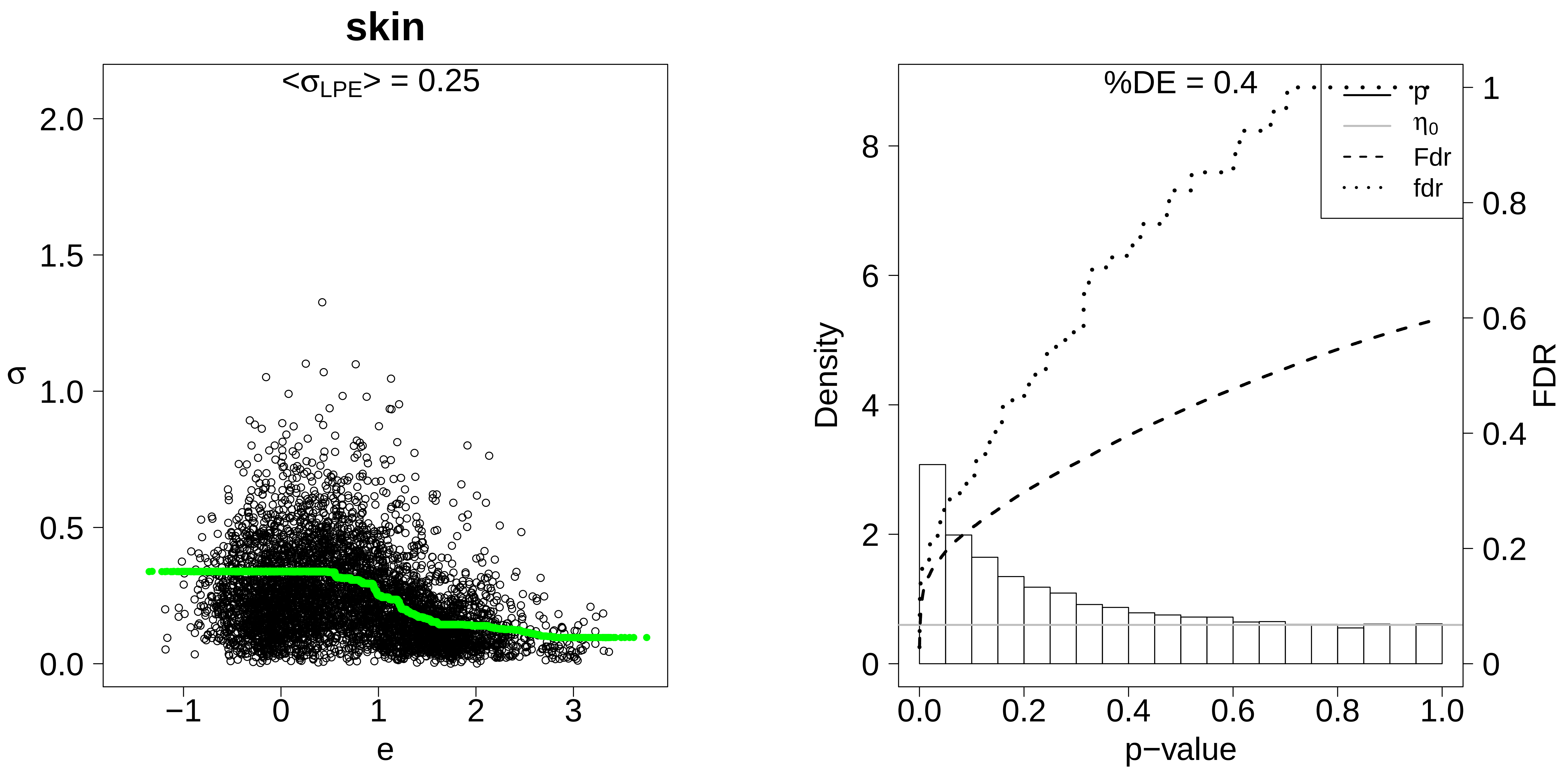

skin
<σLPE> = 0.25
σ
e
%DE = 0.4
p
η0
Fdr
fdr
Density
FDR
p−value

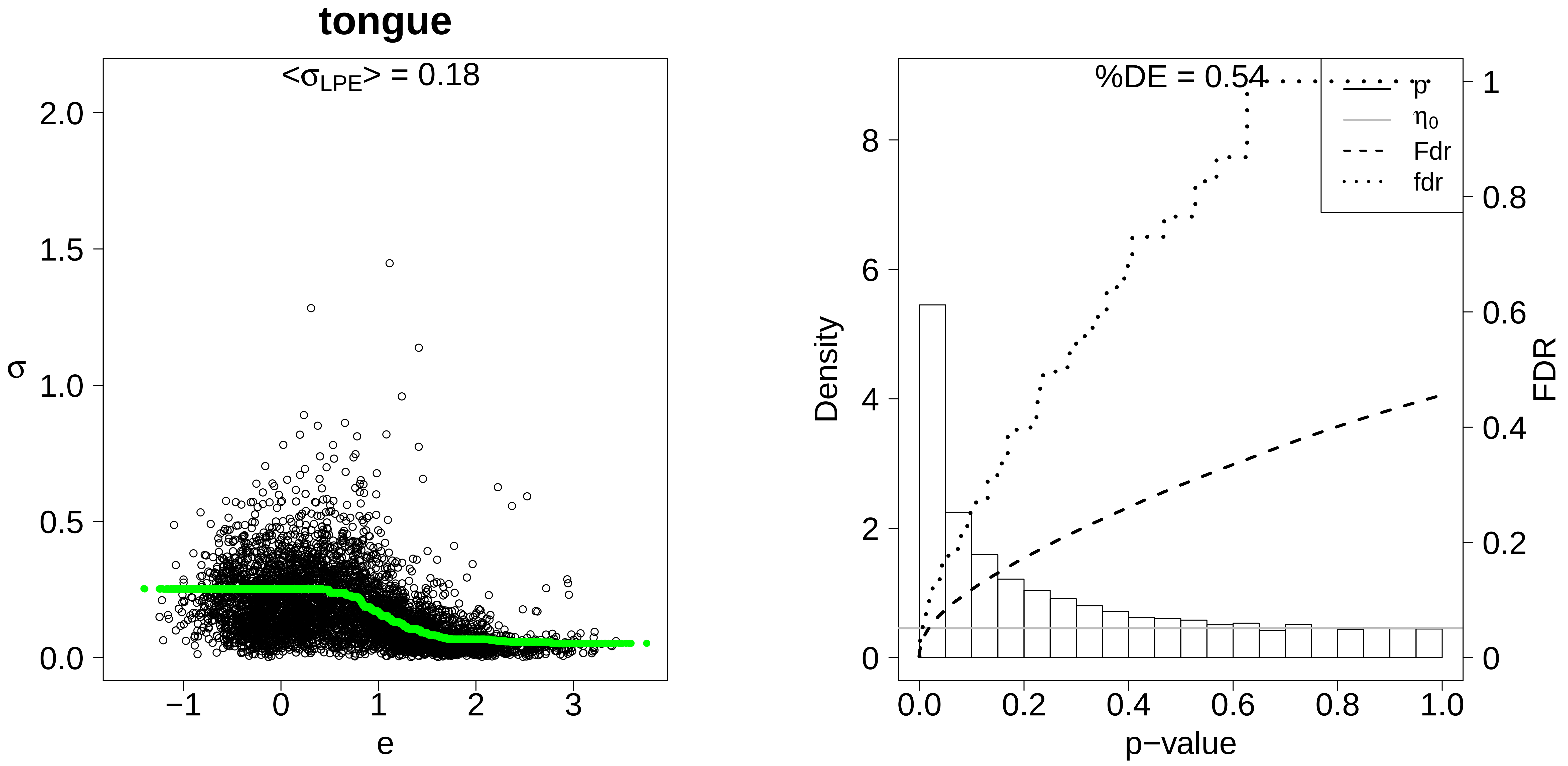

tongue
<σLPE> = 0.18
σ
e
%DE = 0.54
p
η0
Fdr
fdr
Density
p−value
FDR

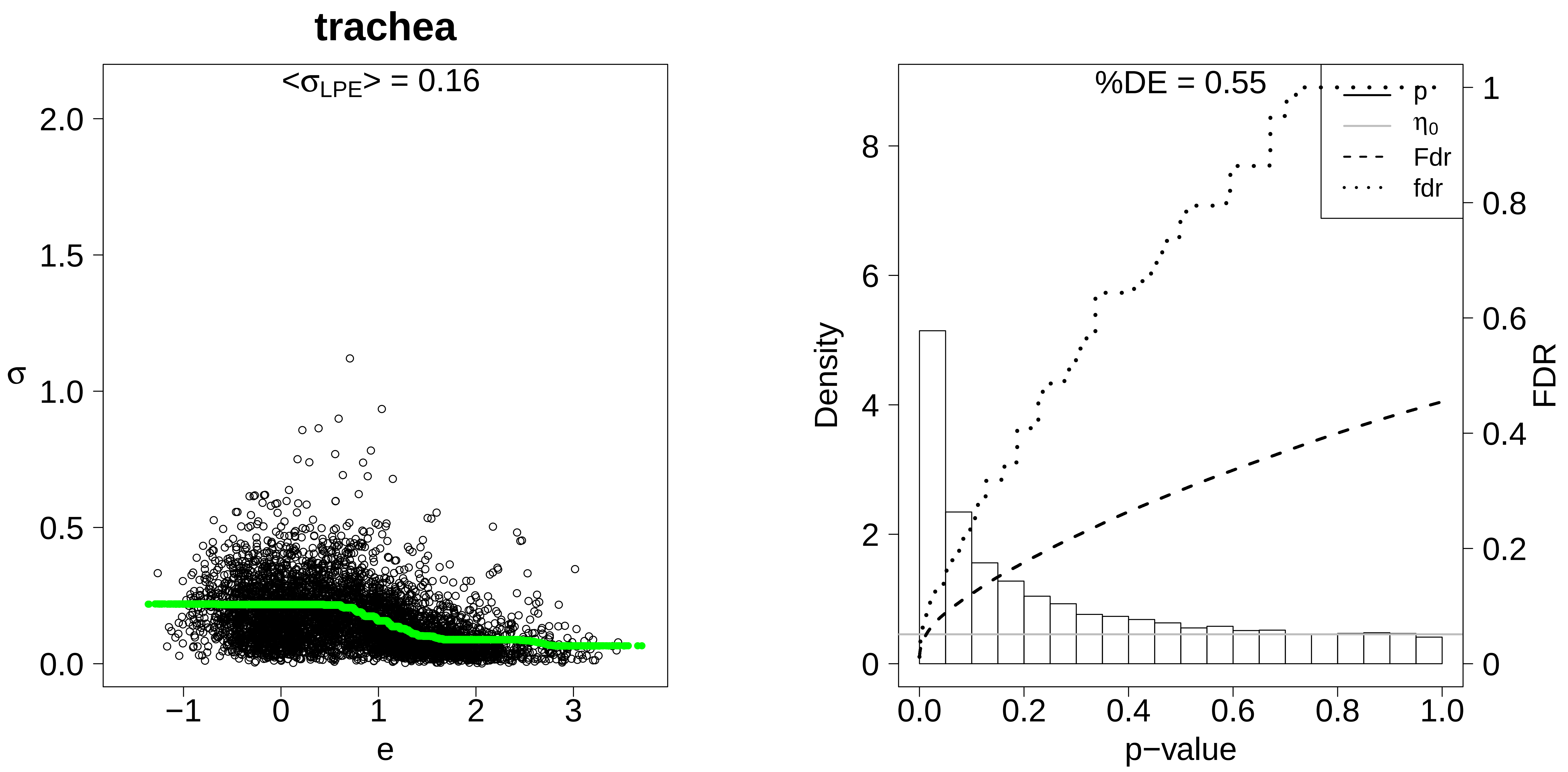

trachea
$<\sigma_{LPE}> = 0.16$
σ
e
%DE = 0.55
p
η_0
Fdr
fdr
Density
FDR
p−value

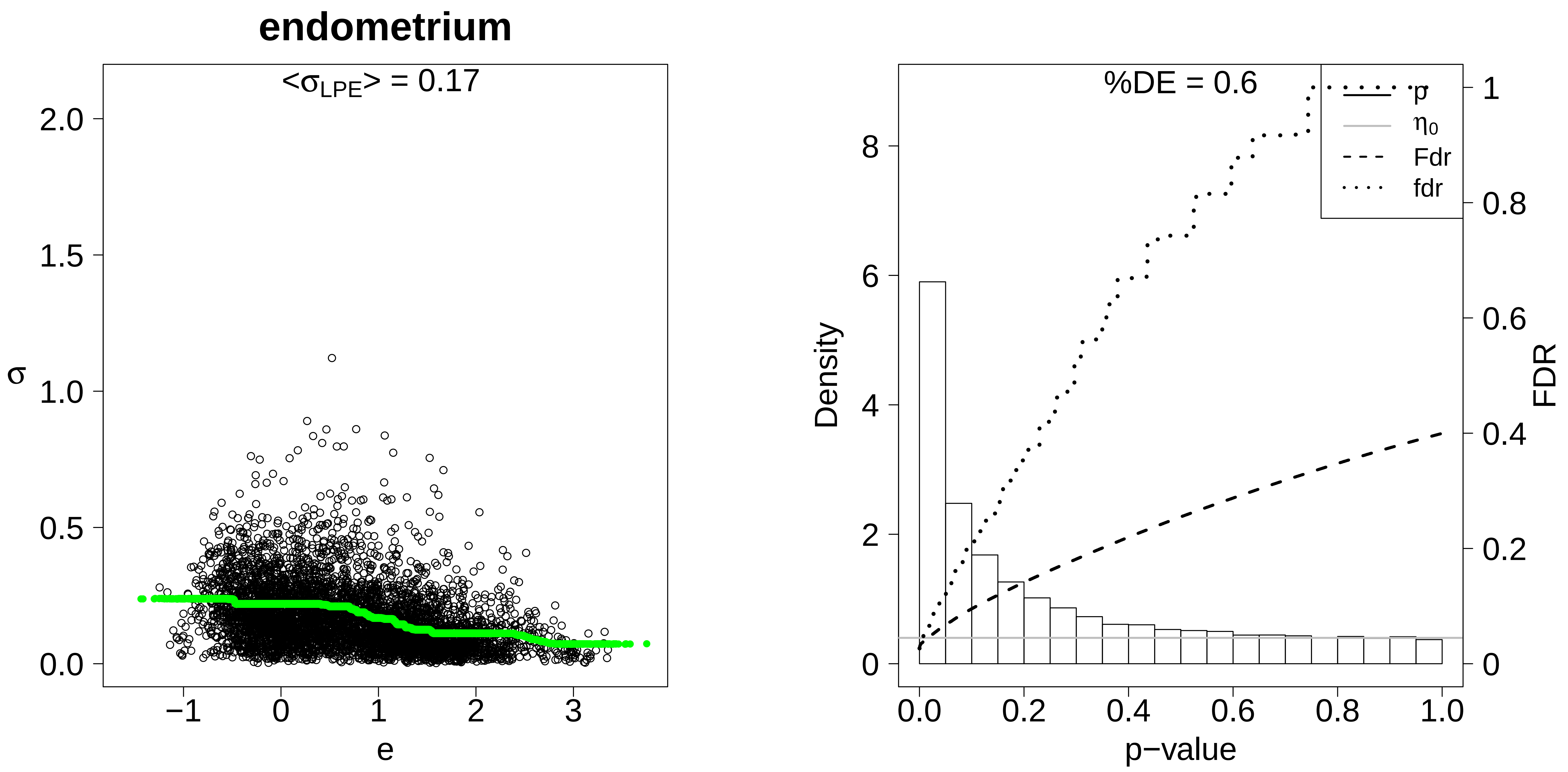

endometrium
<σLPE> = 0.17
σ
e
%DE = 0.6
p
η0
Fdr
fdr
Density
FDR
p−value

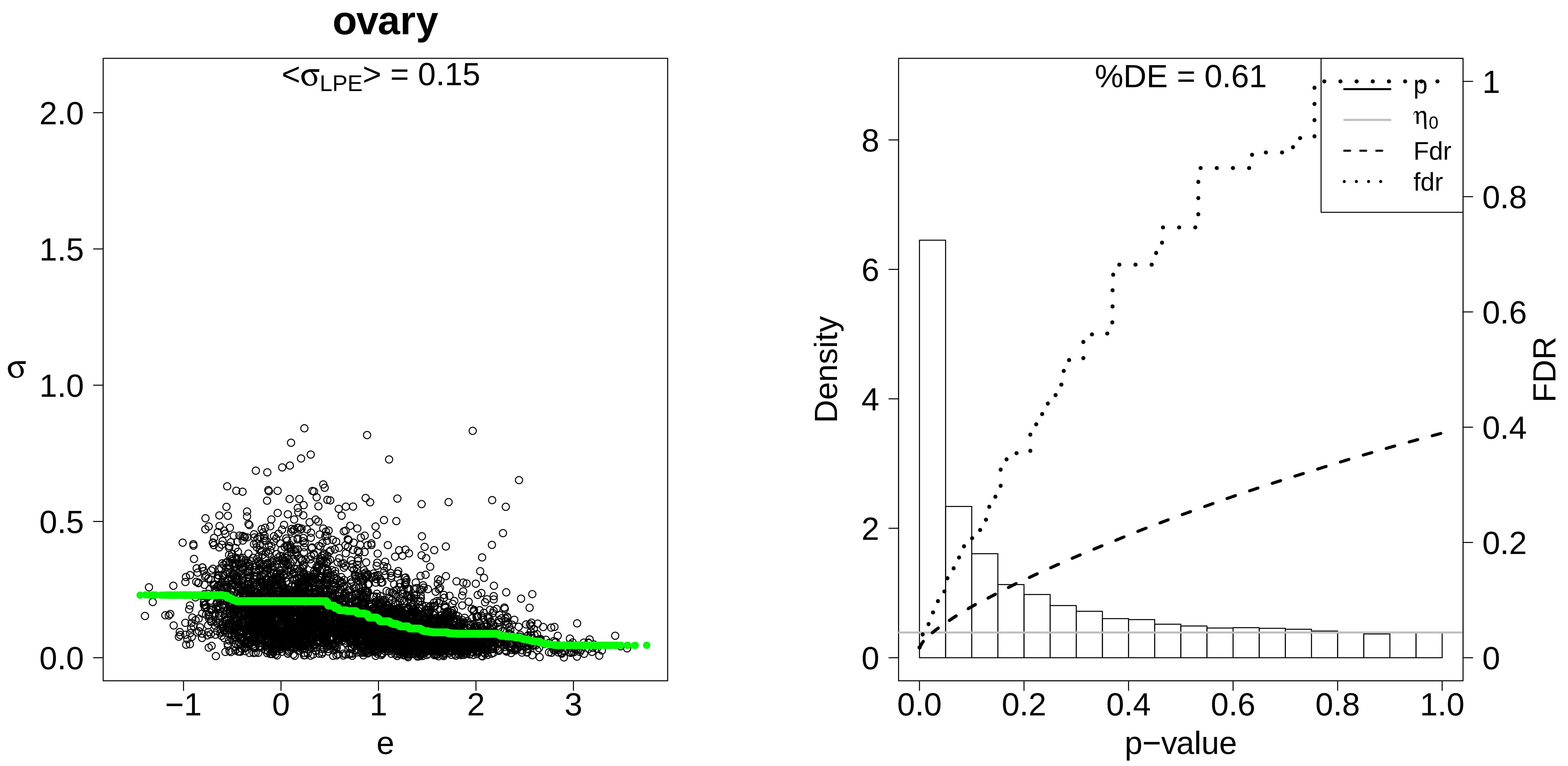

ovary
<σ_LPE> = 0.15
σ
2.0
1.5
1.0
0.5
0.0
−1
0
1
2
3
e
%DE = 0.61
p
η0
Fdr
fdr
Density
8
6
4
2
0
0.0
0.2
0.4
0.6
0.8
1.0
p−value
FDR
1
0.8
0.6
0.4
0.2
0

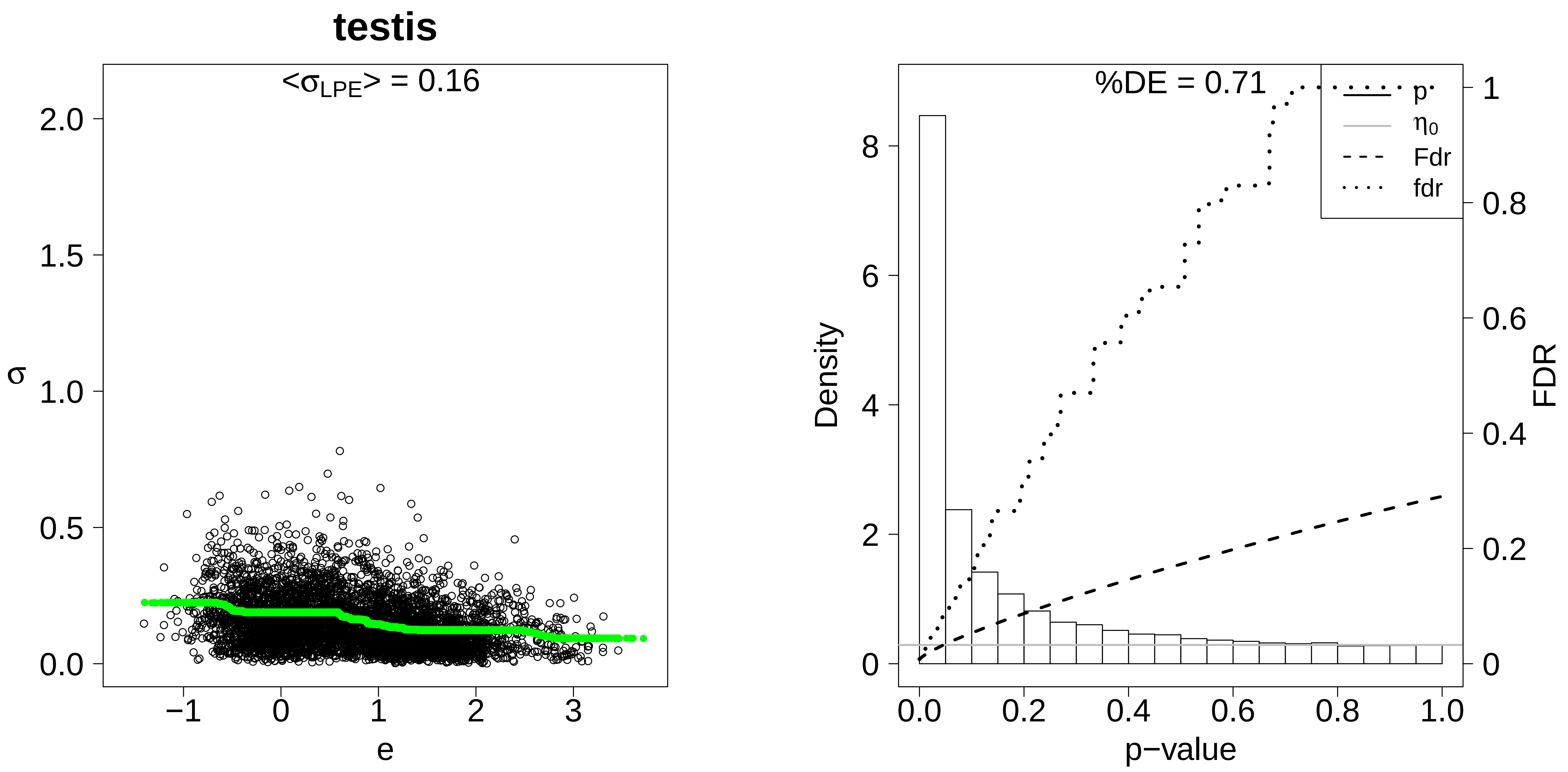

testis
<σ_LPE> = 0.16
σ
e
%DE = 0.71
p
η0
Fdr
fdr
Density
FDR
p−value

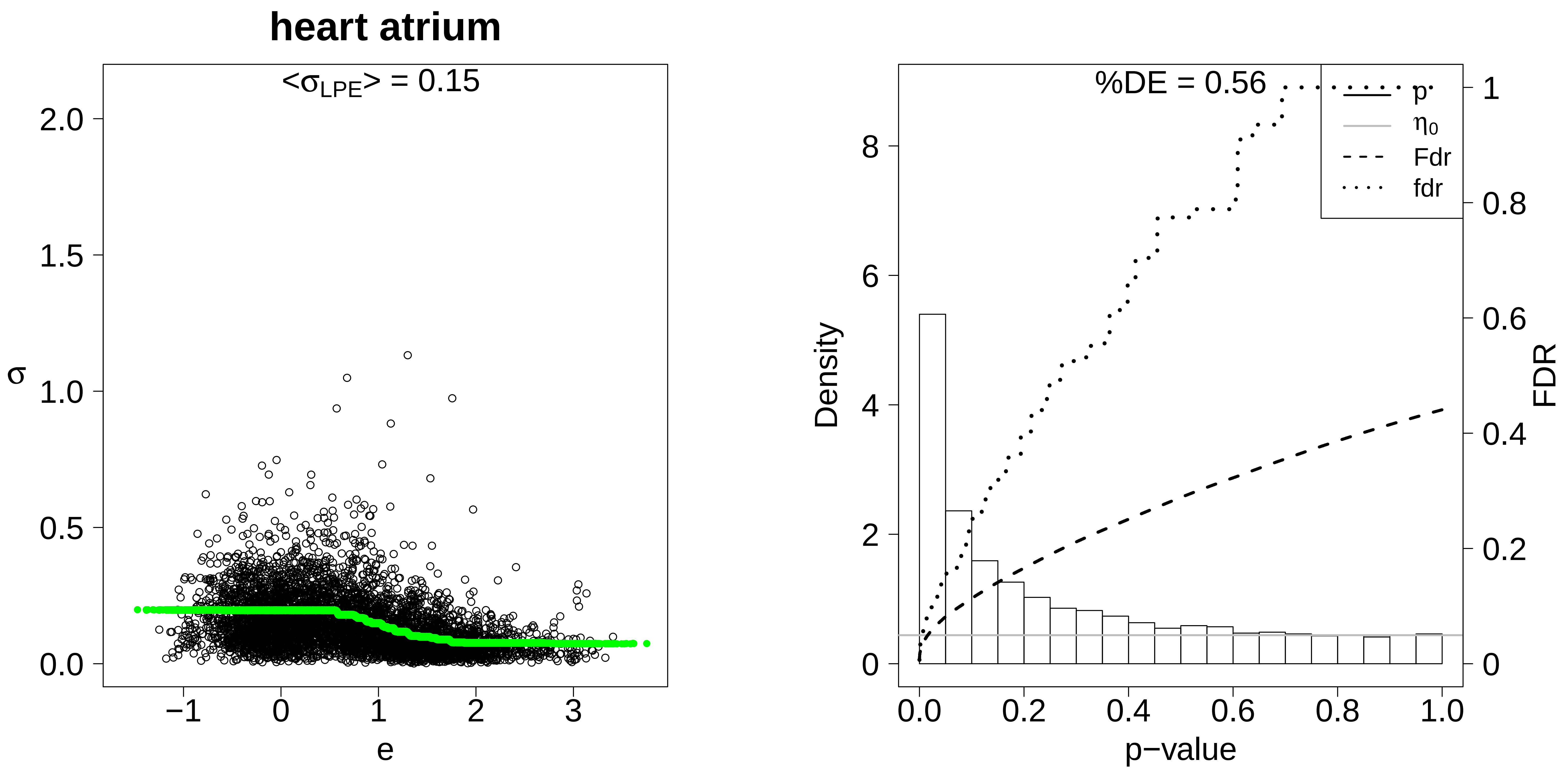
heart atrium
<σLPE> = 0.15
σ
e
%DE = 0.56
p
η0
Fdr
fdr
Density
FDR
p−value

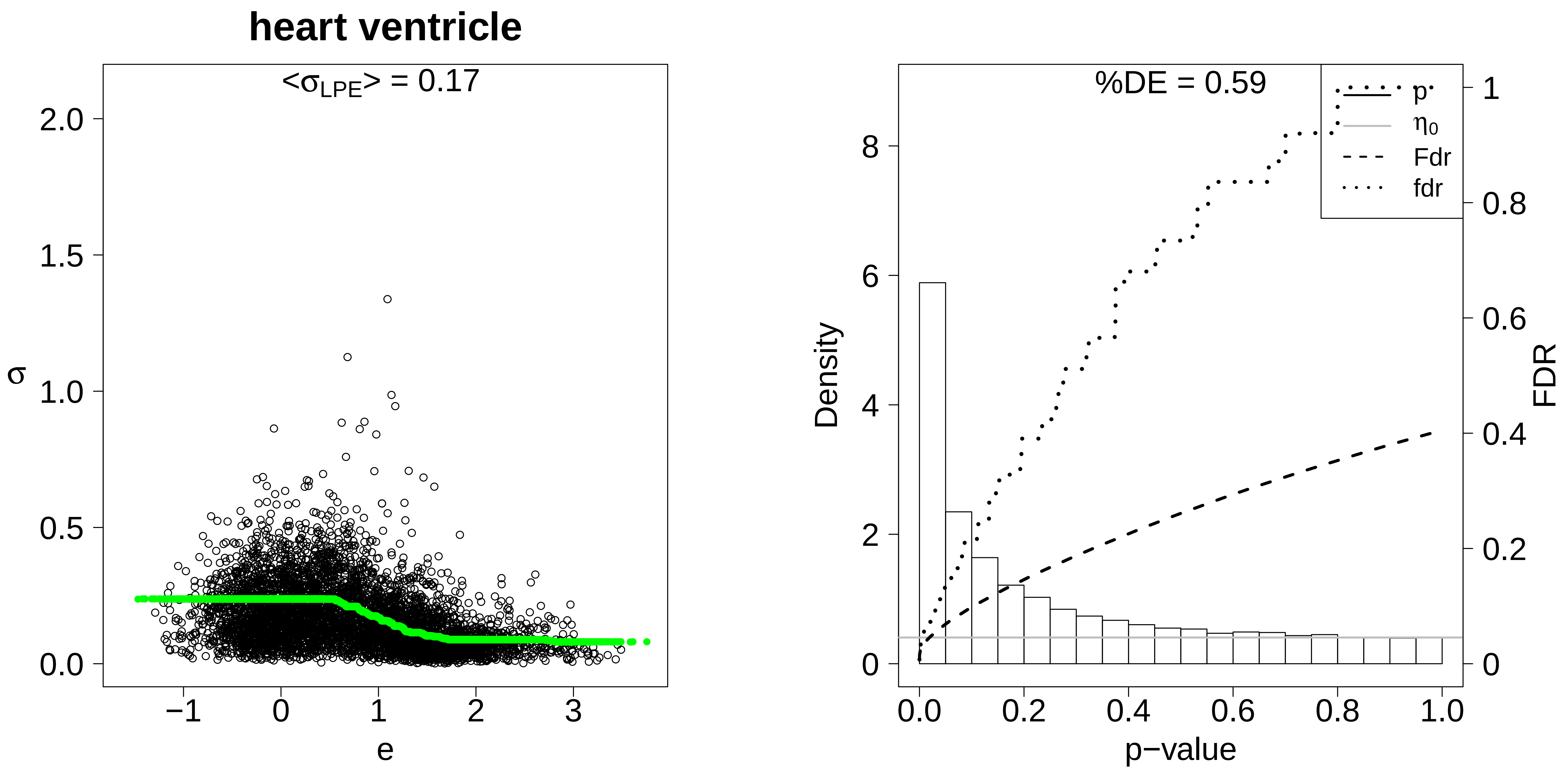

heart ventricle
<σLPE> = 0.17
σ
e
%DE = 0.59
p·
η0
Fdr
fdr
Density
FDR
p−value

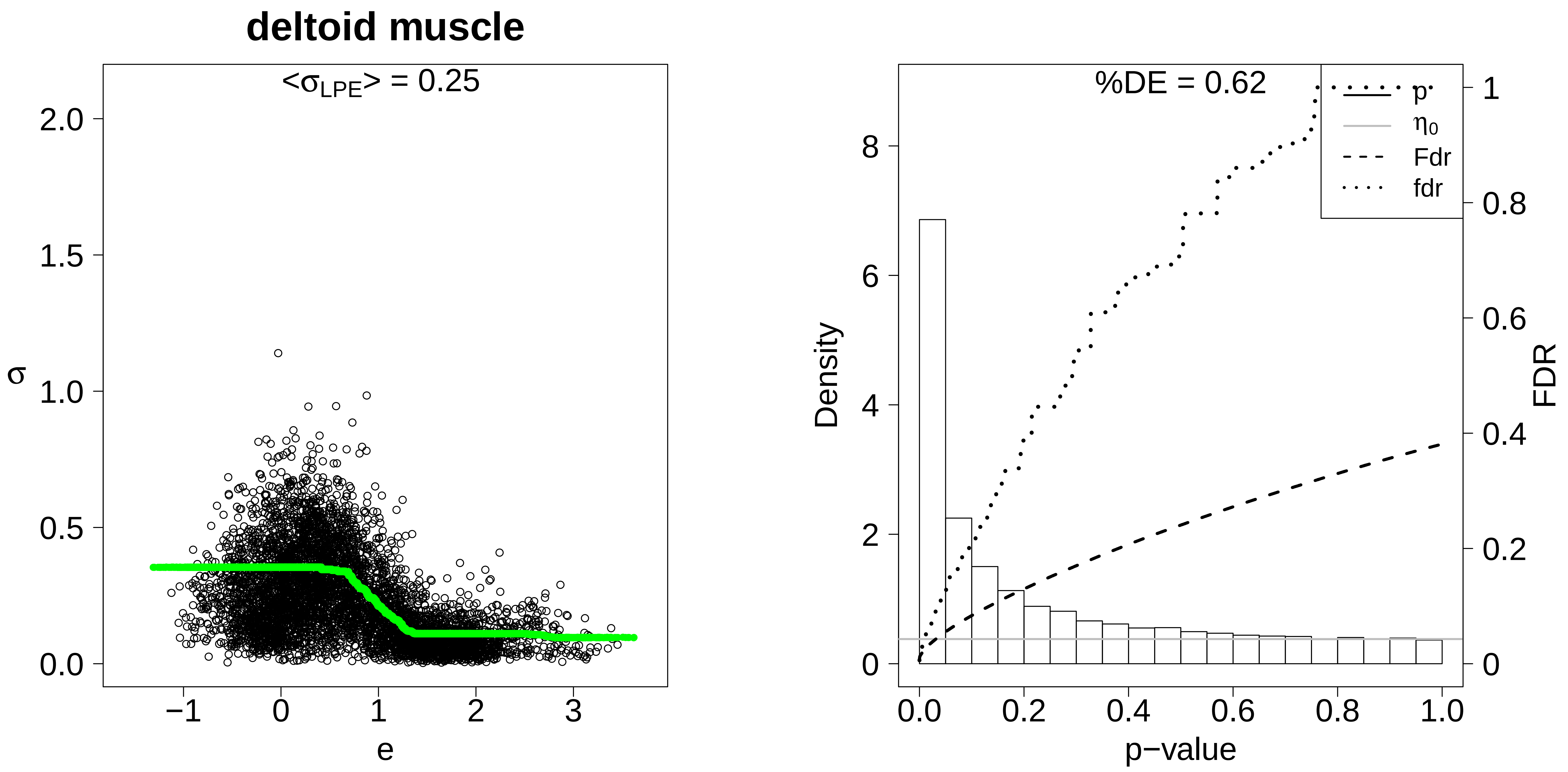

deltoid muscle
<σLPE> = 0.25
σ
e
%DE = 0.62
p·
η0
Fdr
fdr
Density
FDR
p−value

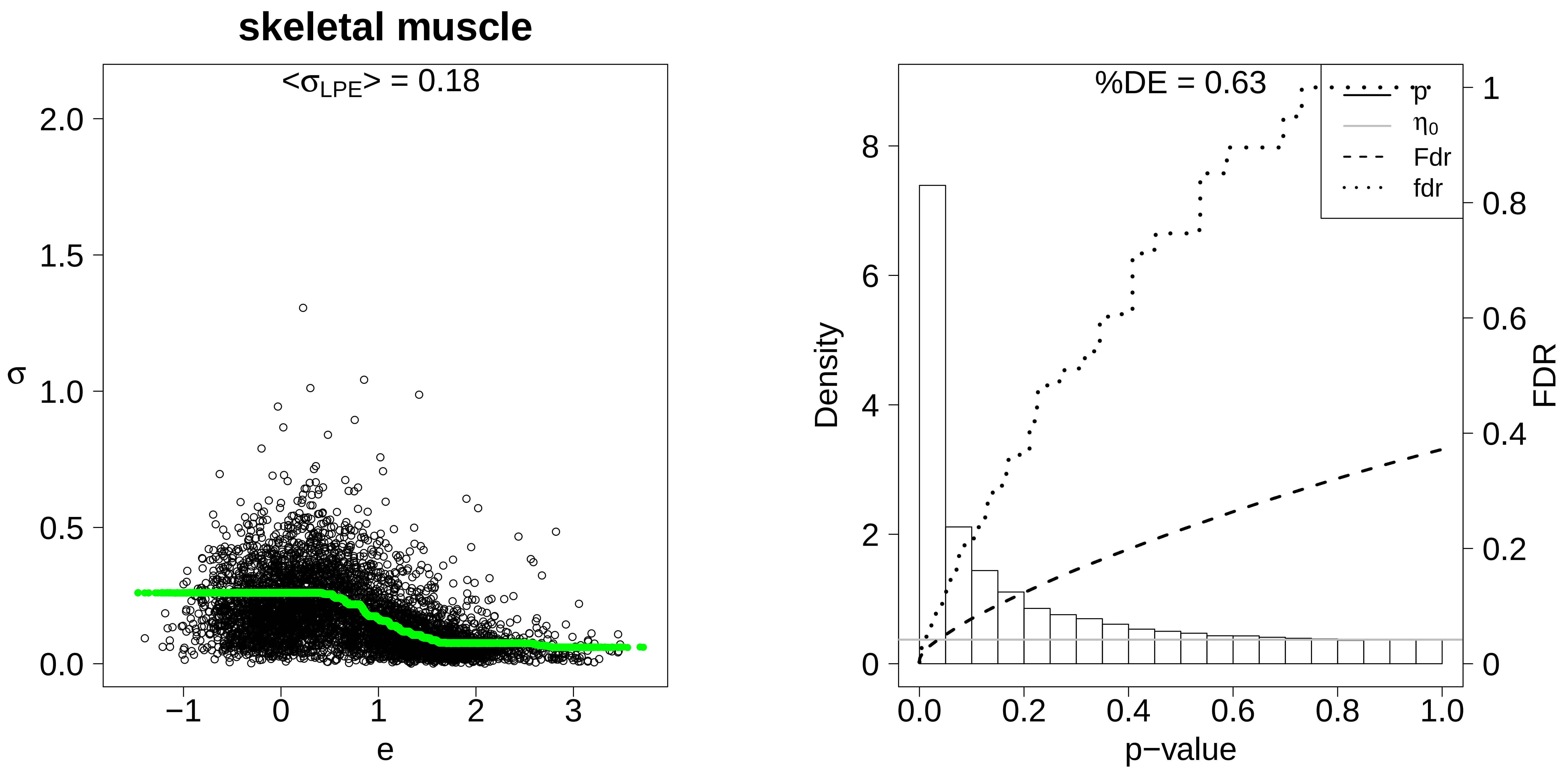

skeletal muscle
<σLPE> = 0.18
σ
e
%DE = 0.63
p
η0
Fdr
fdr
Density
FDR
p−value

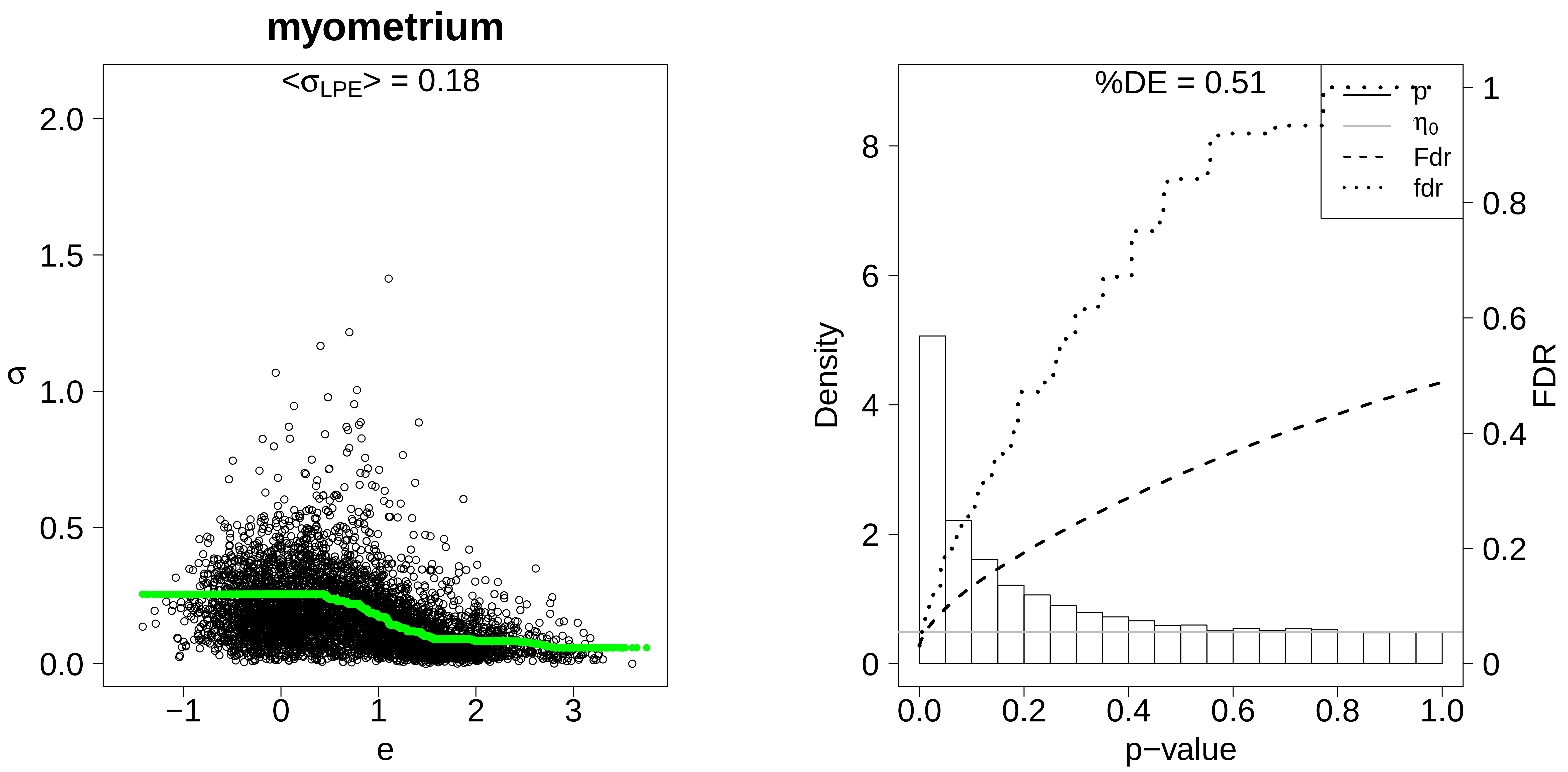

myometrium
<σLPE> = 0.18
σ
e
%DE = 0.51
Density
p−value
FDR
p
η0
Fdr
fdr

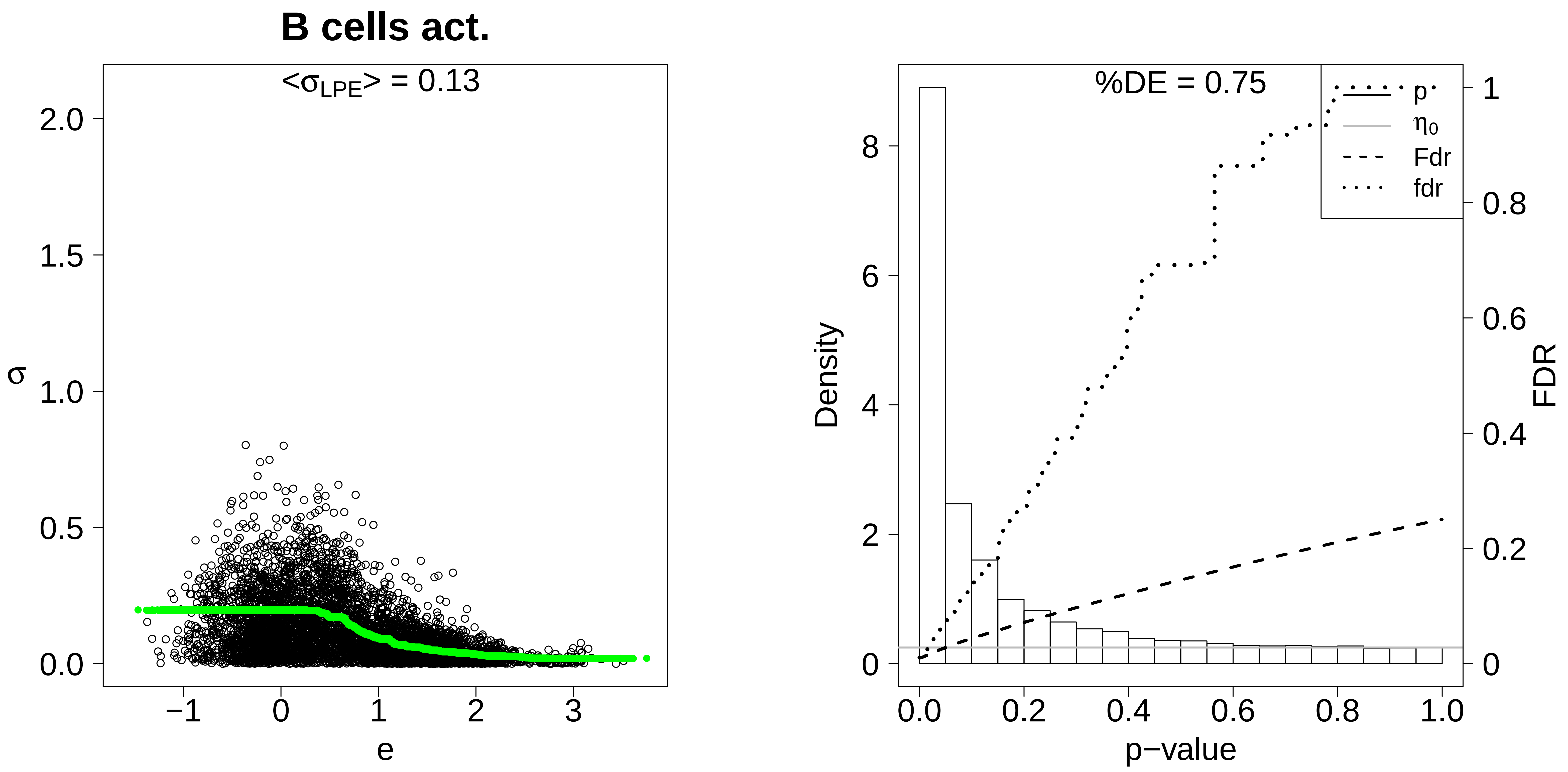

B cells act.
<σLPE> = 0.13
2.0
1.5
1.0
0.5
0.0
σ
−1
0
1
2
3
e
%DE = 0.75
p
η0
Fdr
fdr
Density
8
6
4
2
0
0.0
0.2
0.4
0.6
0.8
1.0
p−value
FDR
1
0.8
0.6
0.4
0.2
0

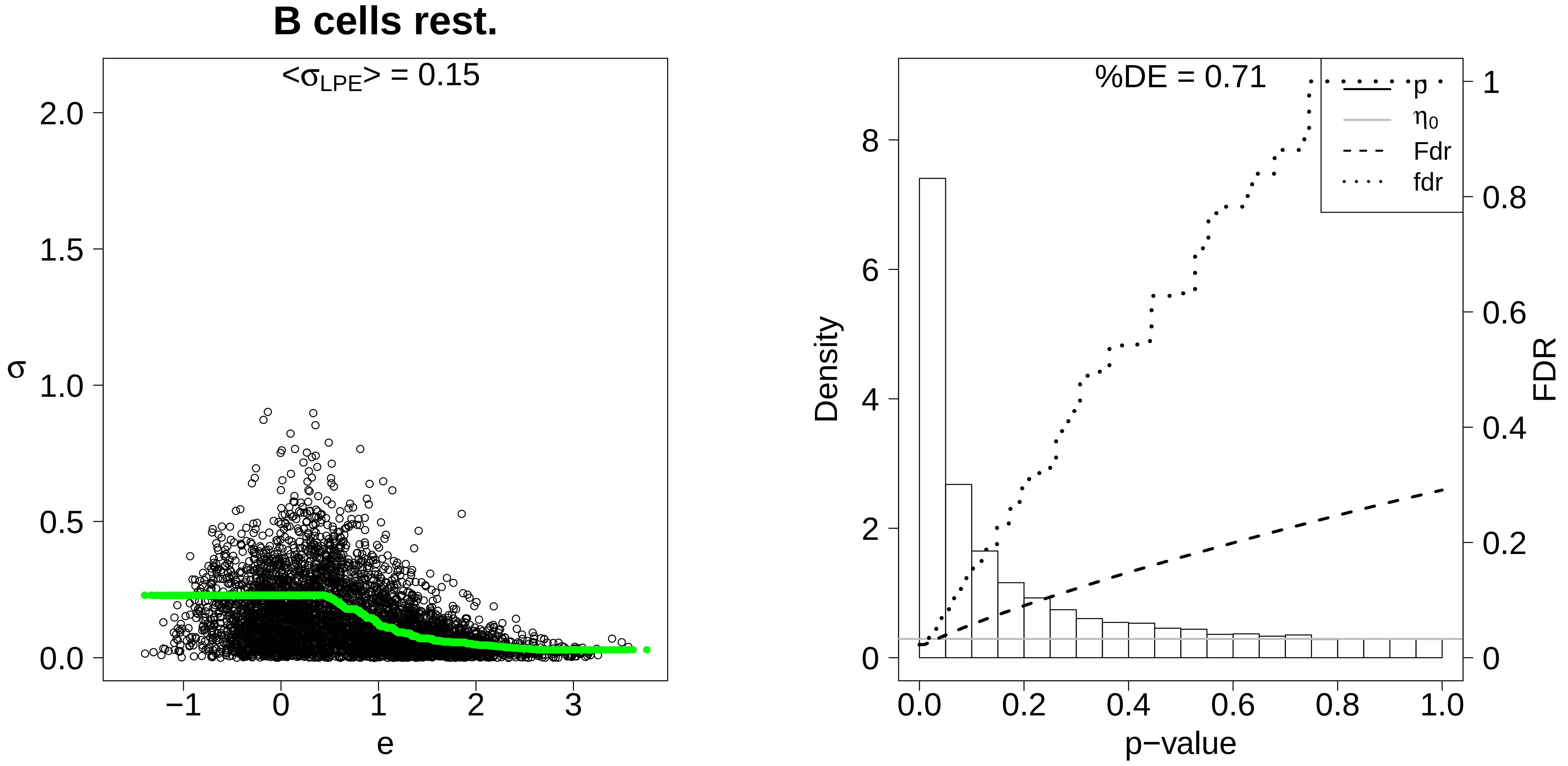
B cells rest.
<σLPE> = 0.15
σ
e
%DE = 0.71
p
η0
Fdr
fdr
Density
FDR
p−value

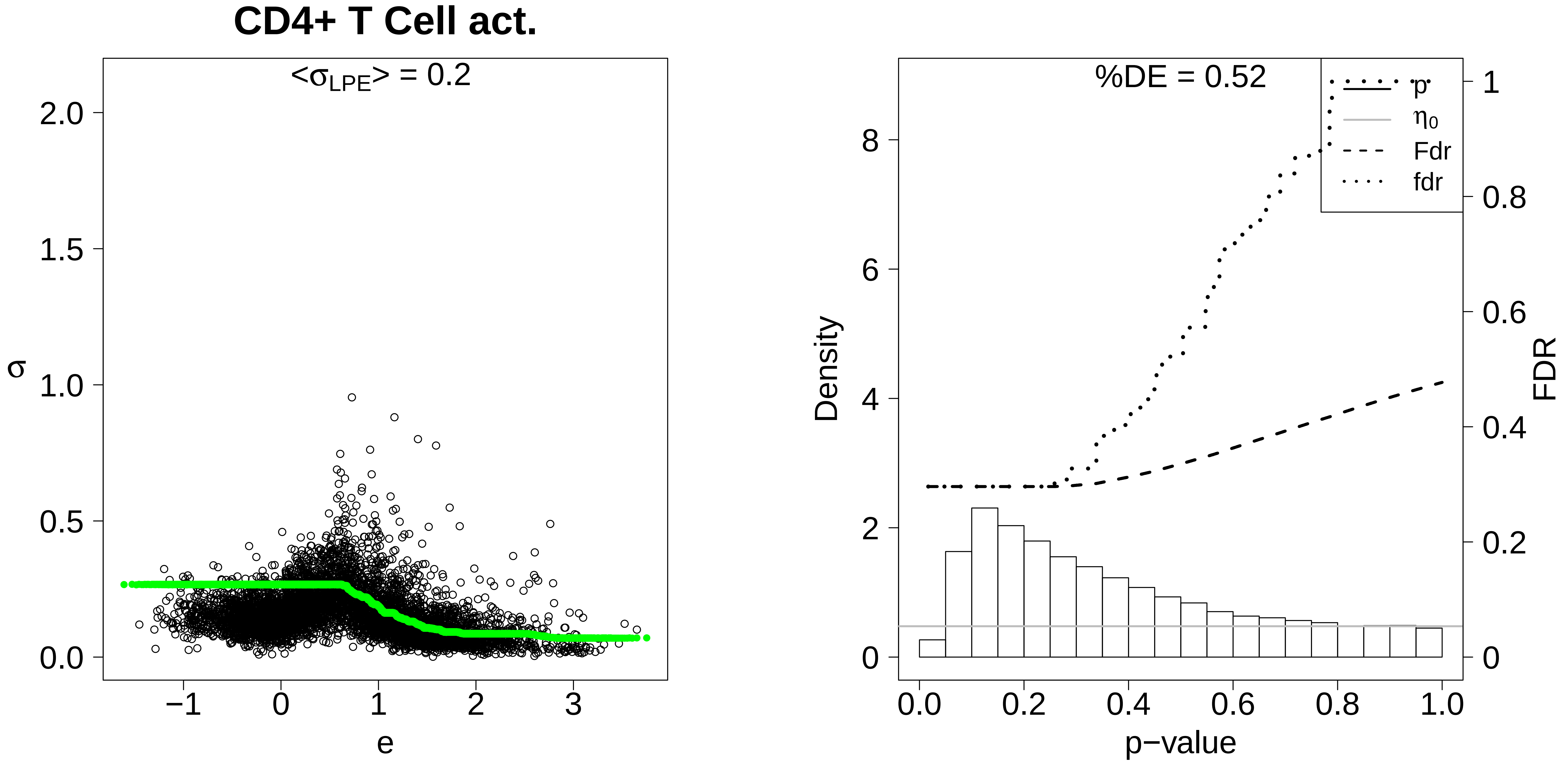
CD4+ T Cell act.
<σLPE> = 0.2
σ
e
2.0
1.5
1.0
0.5
0.0
−1
0
1
2
3
%DE = 0.52
p
η0
Fdr
fdr
Density
FDR
p−value
8
6
4
2
0
0.0
0.2
0.4
0.6
0.8
1.0
1
0.8
0.6
0.4
0.2
0

CD4+ T Cell rest.

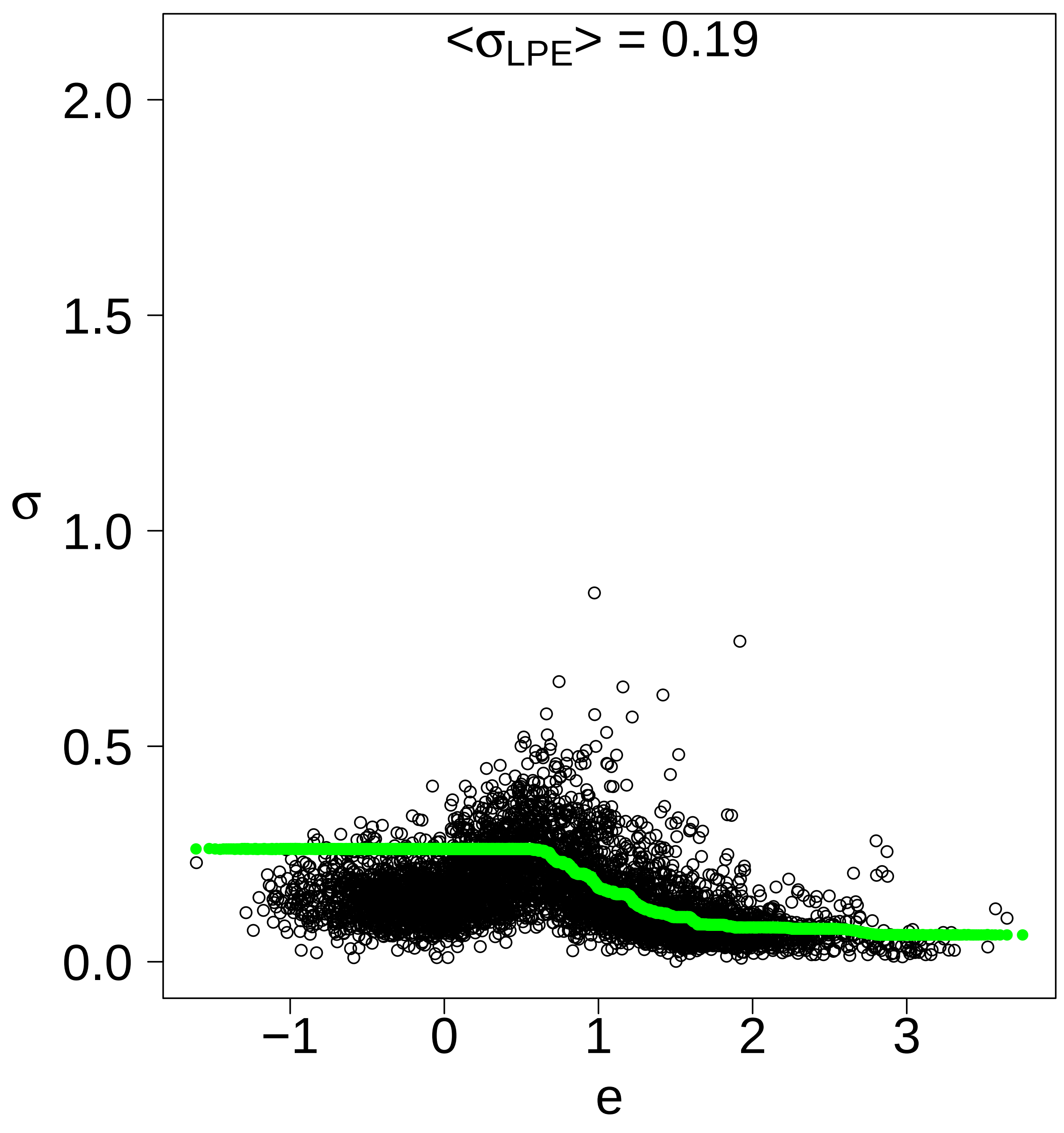

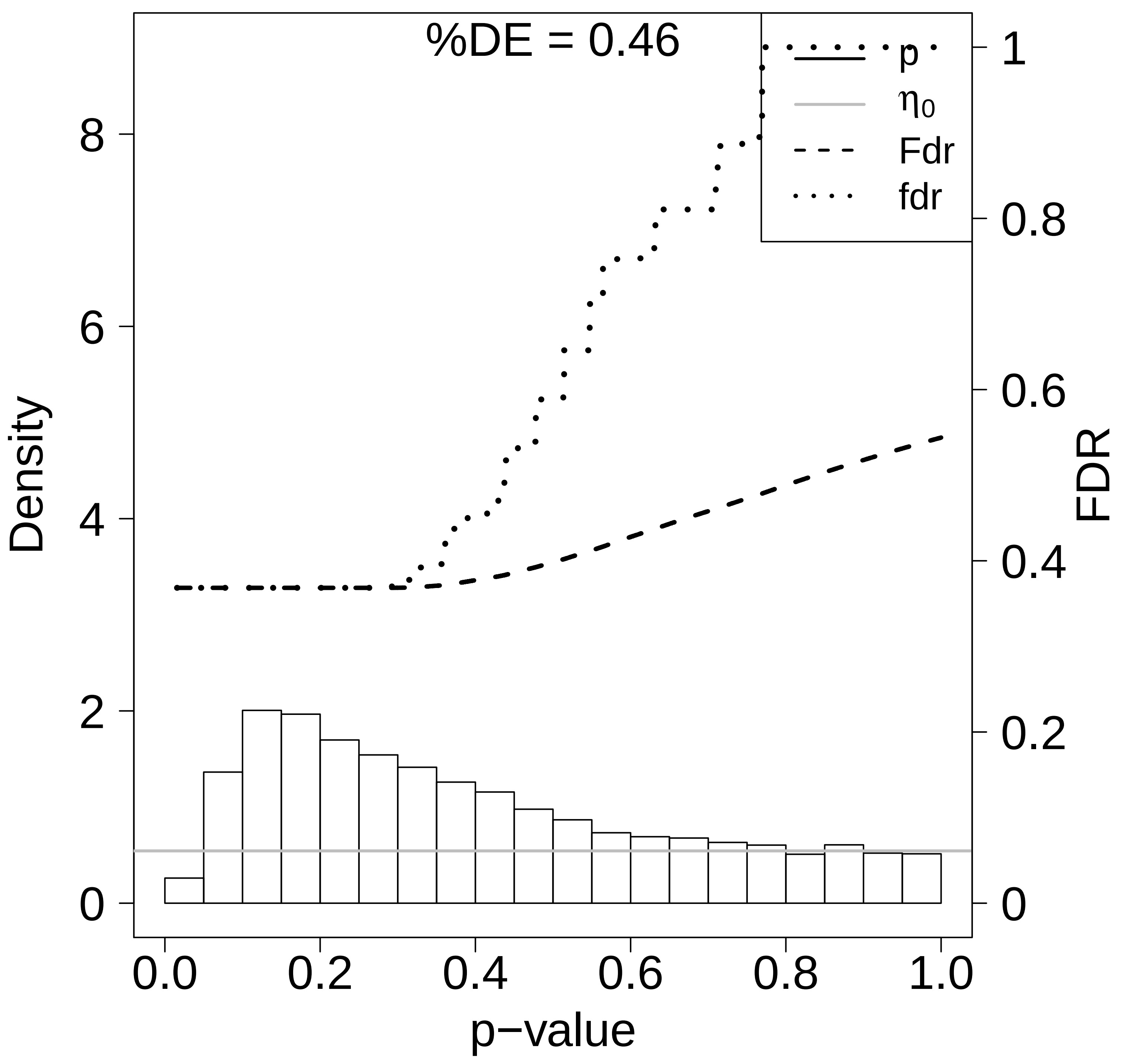

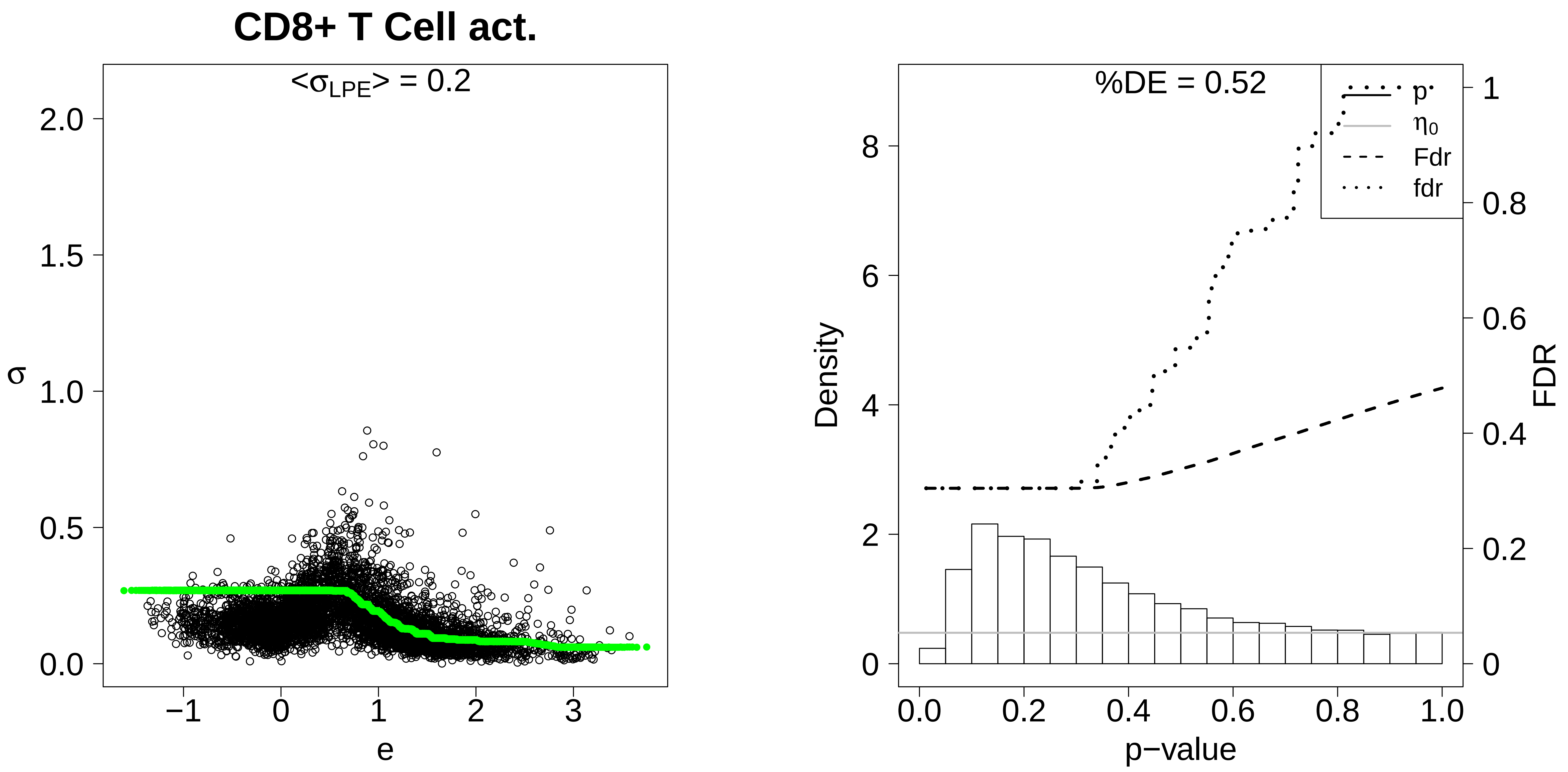

CD8+ T Cell act.
<σLPE> = 0.2
σ
e
%DE = 0.52
p·
η0
Fdr
fdr
Density
FDR
p−value

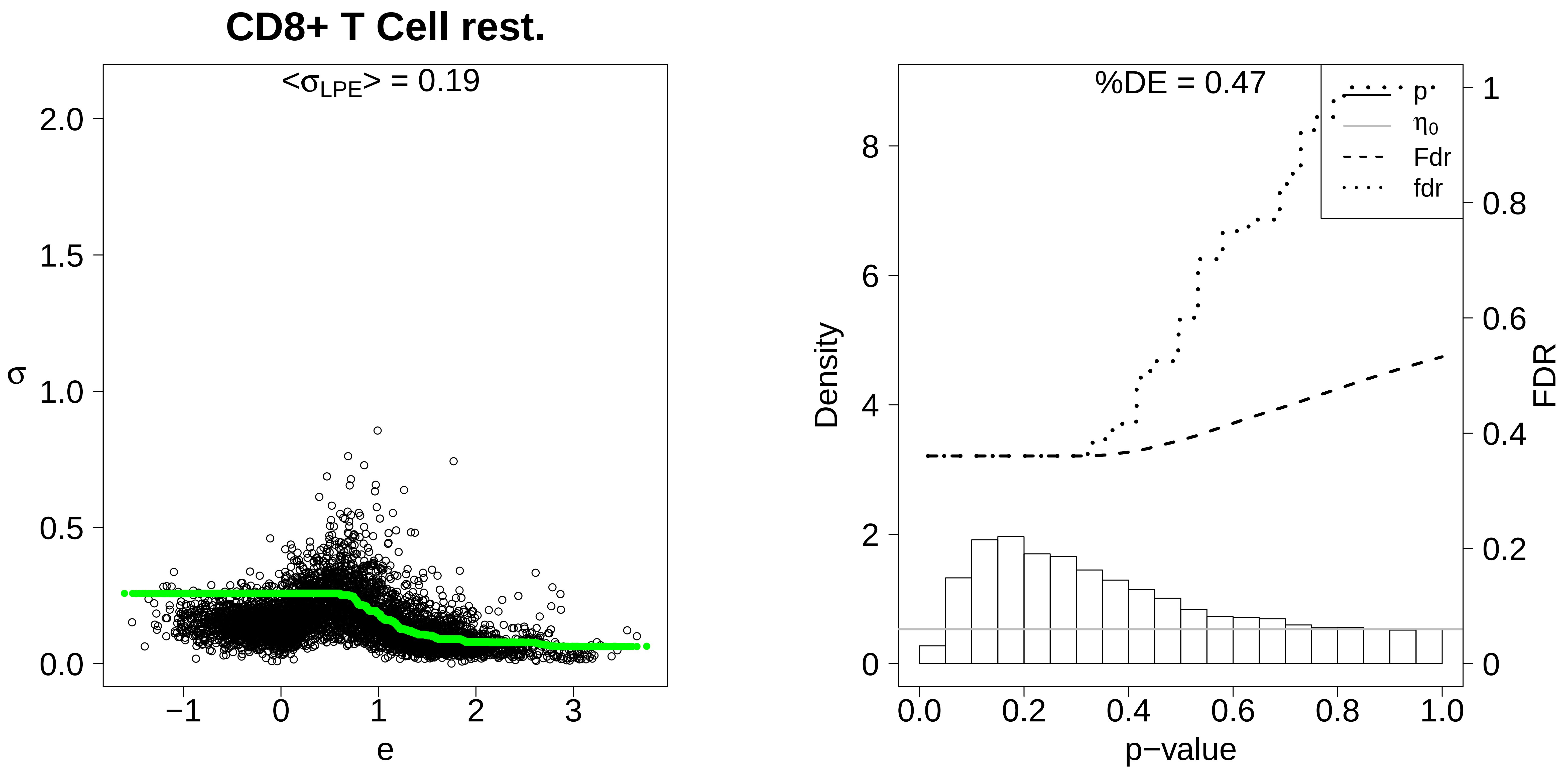

CD8+ T Cell rest.
<σLPE> = 0.19
σ
e
%DE = 0.47
p
η0
Fdr
fdr
Density
FDR
p−value

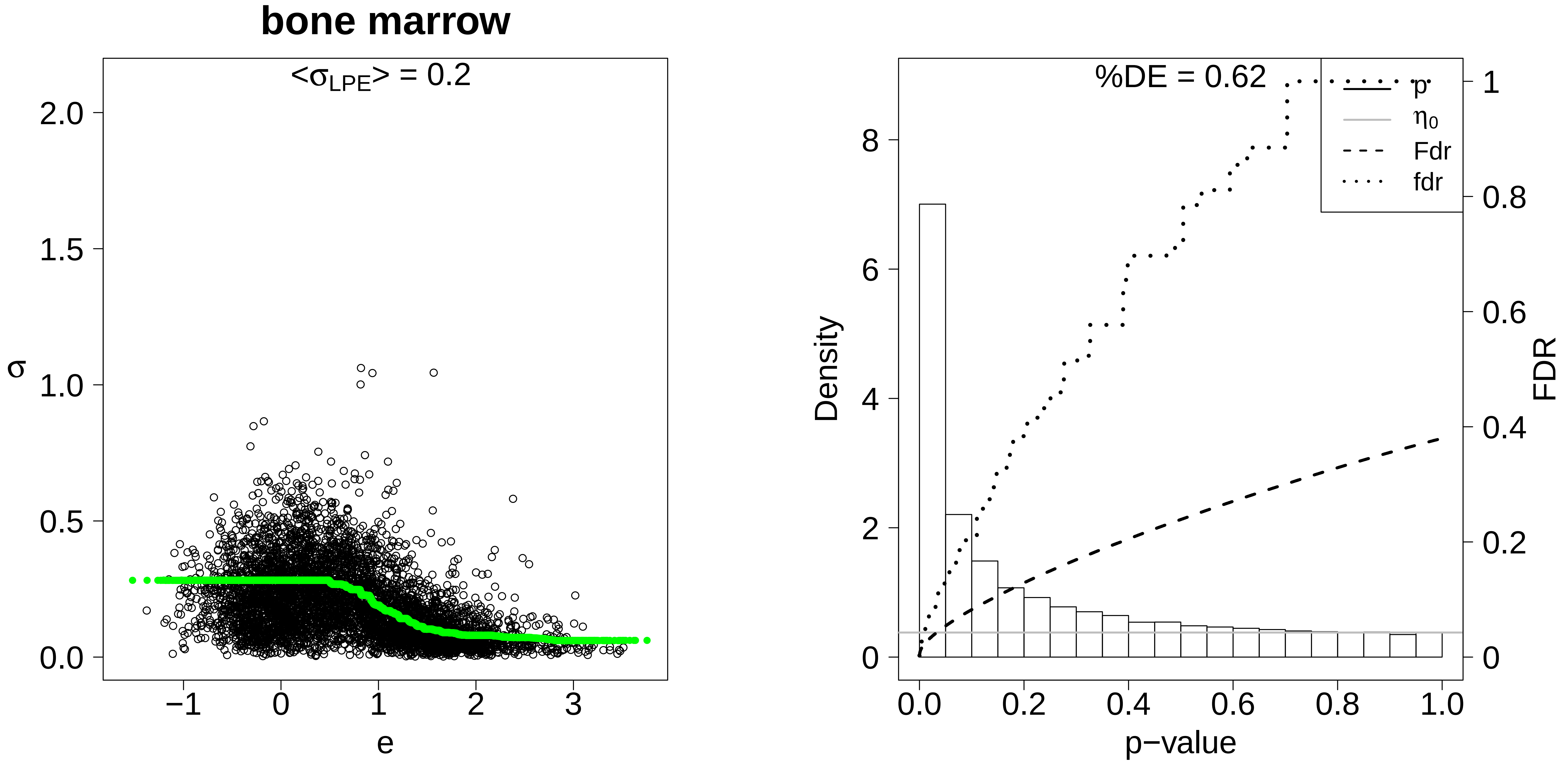
bone marrow
<σLPE> = 0.2
2.0
1.5
1.0
0.5
0.0
σ
−1
0
1
2
3
e
%DE = 0.62
p
η0
Fdr
fdr
Density
8
6
4
2
0
FDR
1
0.8
0.6
0.4
0.2
0
0.0
0.2
0.4
0.6
0.8
1.0
p−value

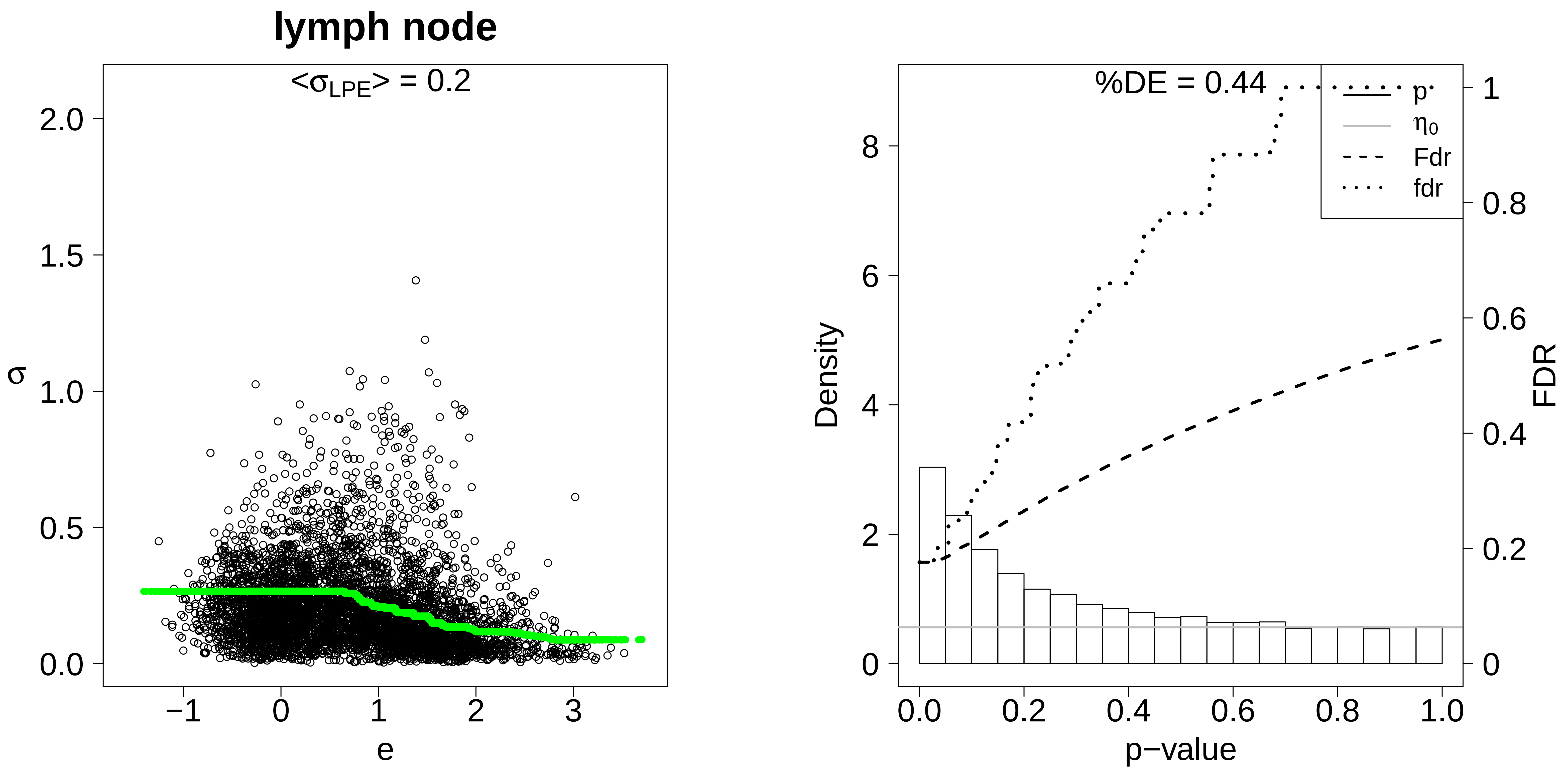

lymph node
<σ_LPE> = 0.2
σ
e
%DE = 0.44
p
η0
Fdr
fdr
Density
FDR
p−value

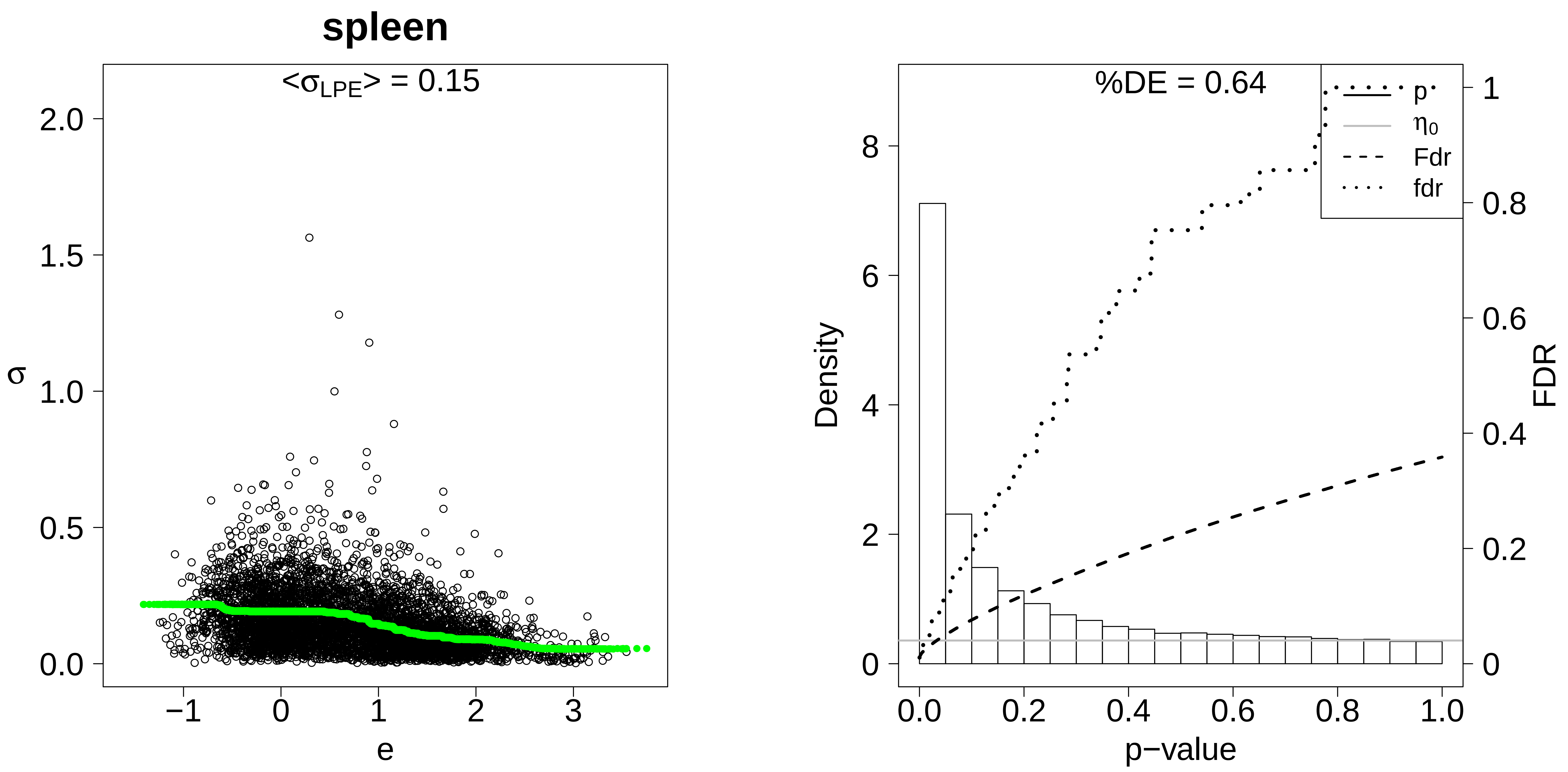

spleen
<σLPE> = 0.15
σ
e
%DE = 0.64
p
η0
Fdr
fdr
Density
FDR
p−value

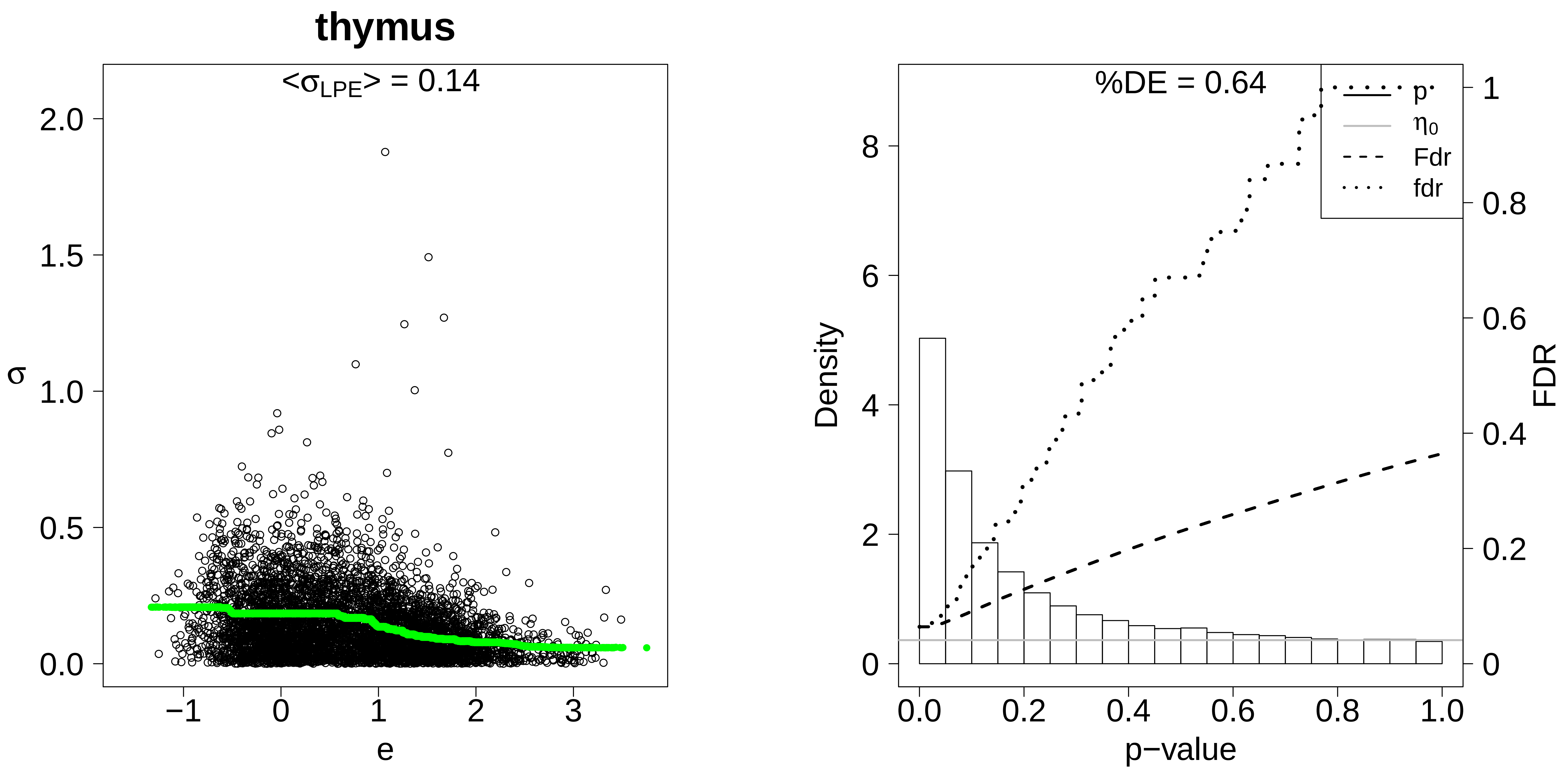
thymus
<σLPE> = 0.14
σ
2.0
1.5
1.0
0.5
0.0
−1
0
1
2
3
e
%DE = 0.64
Density
8
6
4
2
0
0.0
0.2
0.4
0.6
0.8
1.0
p−value
FDR
1
0.8
0.6
0.4
0.2
0
p·
η0
Fdr
fdr

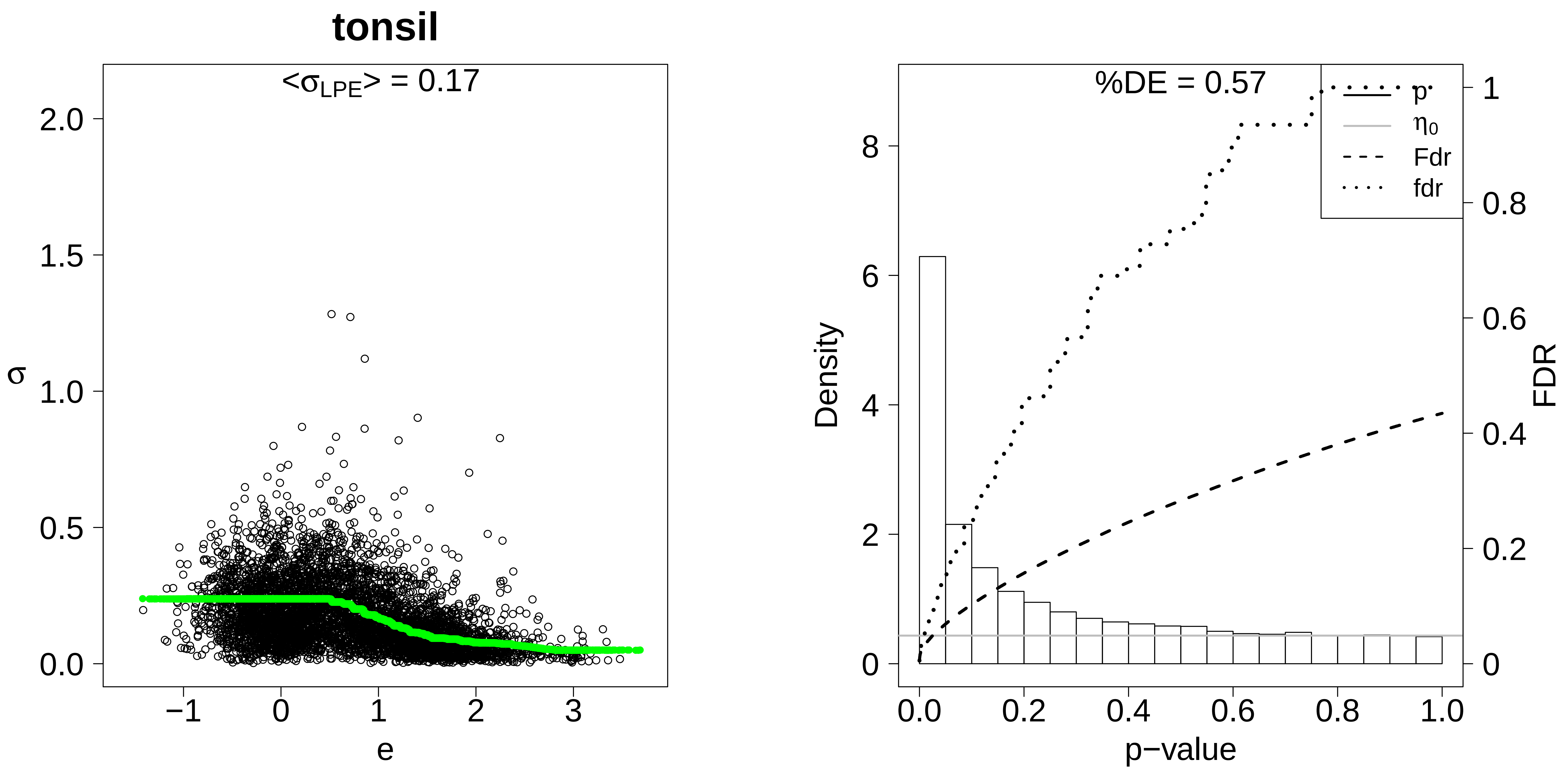

tonsil
<σLPE> = 0.17
σ
e
2.0
1.5
1.0
0.5
0.0
−1
0
1
2
3
%DE = 0.57
p
η0
Fdr
fdr
Density
FDR
p−value
0.0
0.2
0.4
0.6
0.8
1.0
8
6
4
2
0
1
0.8
0.6
0.4
0.2
0

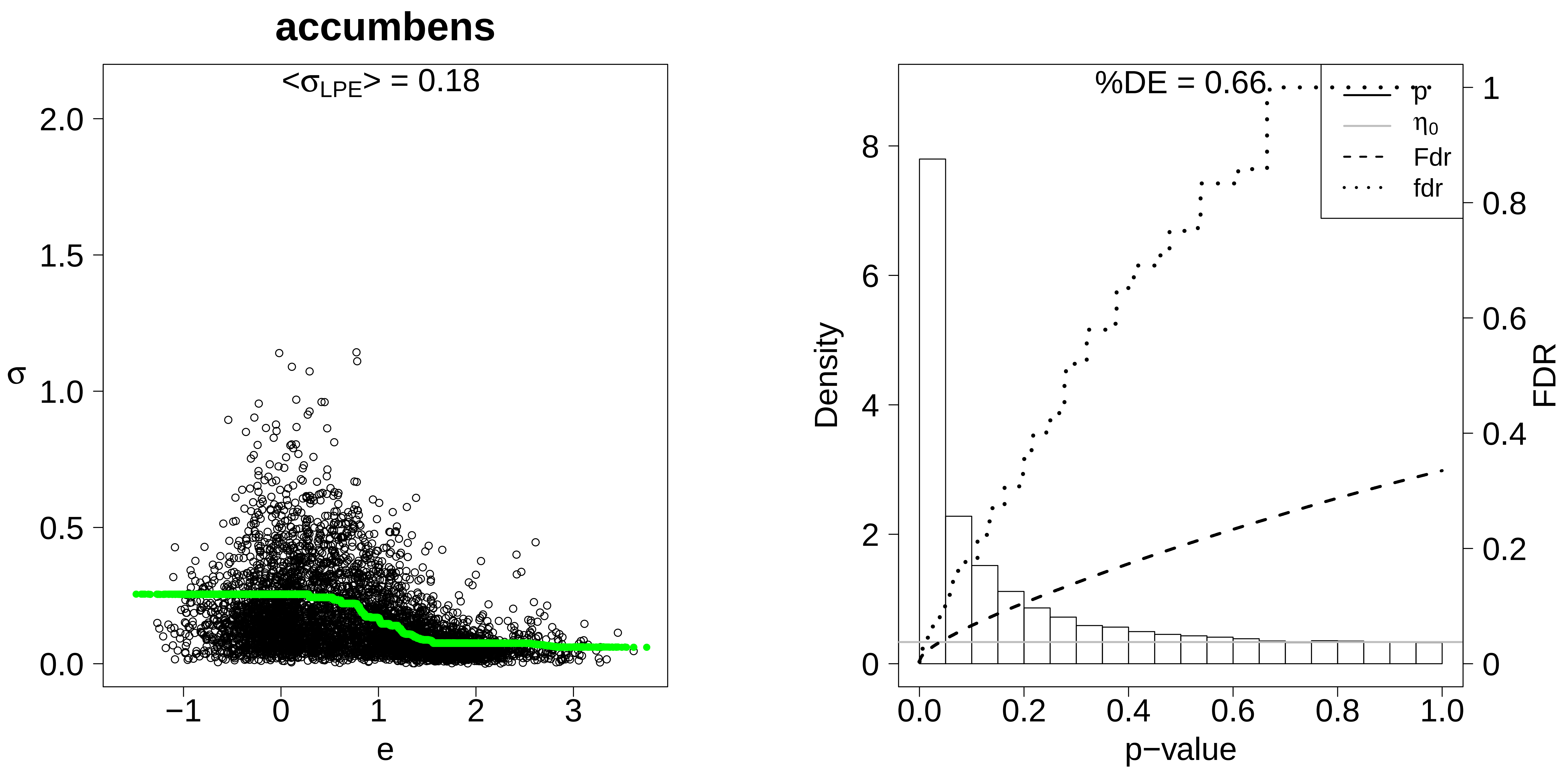

accumbens
<σ_LPE> = 0.18
2.0
1.5
1.0
0.5
0.0
σ
−1
0
1
2
3
e
%DE = 0.66
p
η0
Fdr
fdr
8
6
4
2
0
Density
1
0.8
0.6
0.4
0.2
0
FDR
0.0
0.2
0.4
0.6
0.8
1.0
p−value

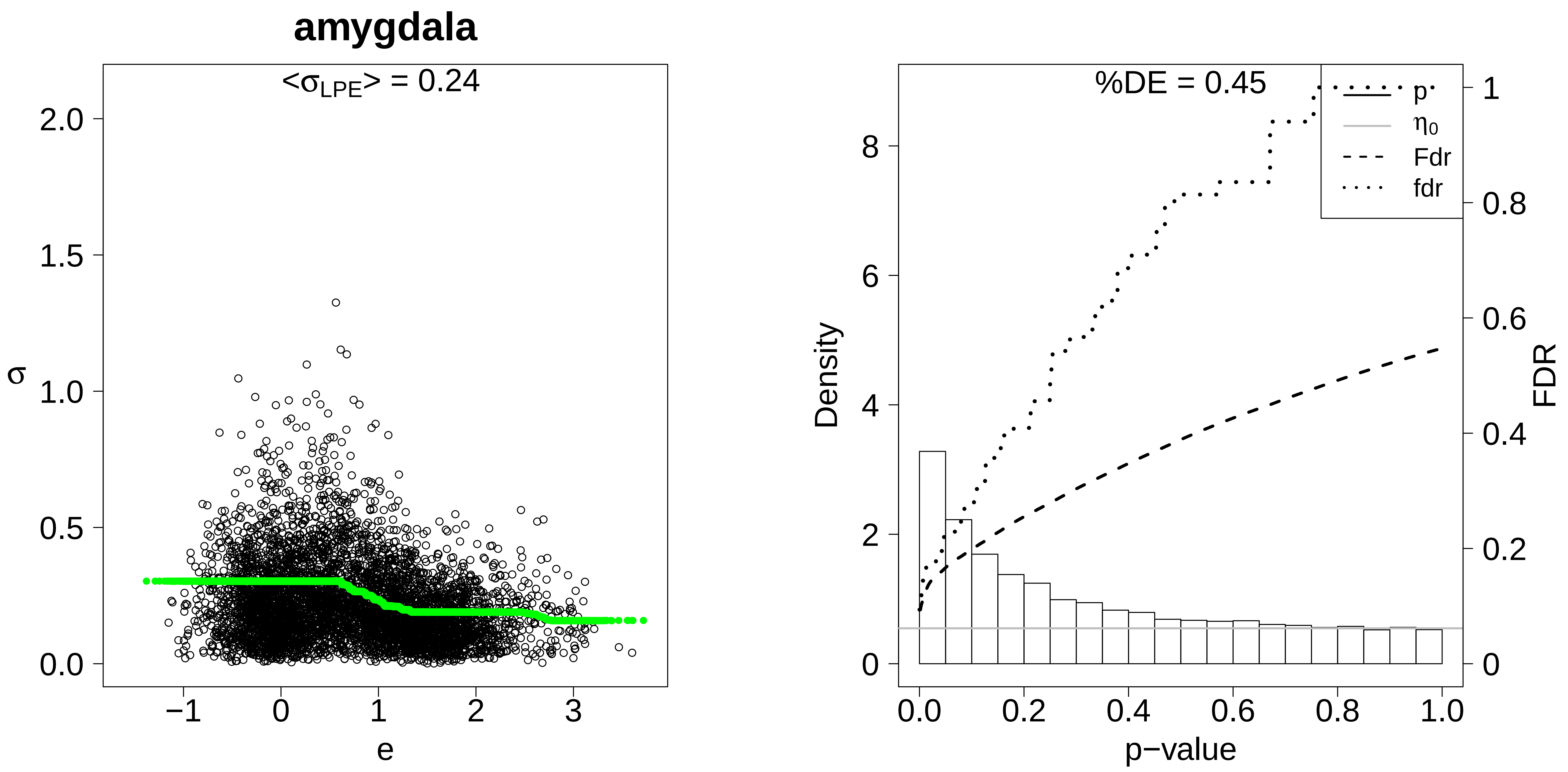

amygdala
<σLPE> = 0.24
σ
e
%DE = 0.45
p
η0
Fdr
fdr
Density
FDR
p−value

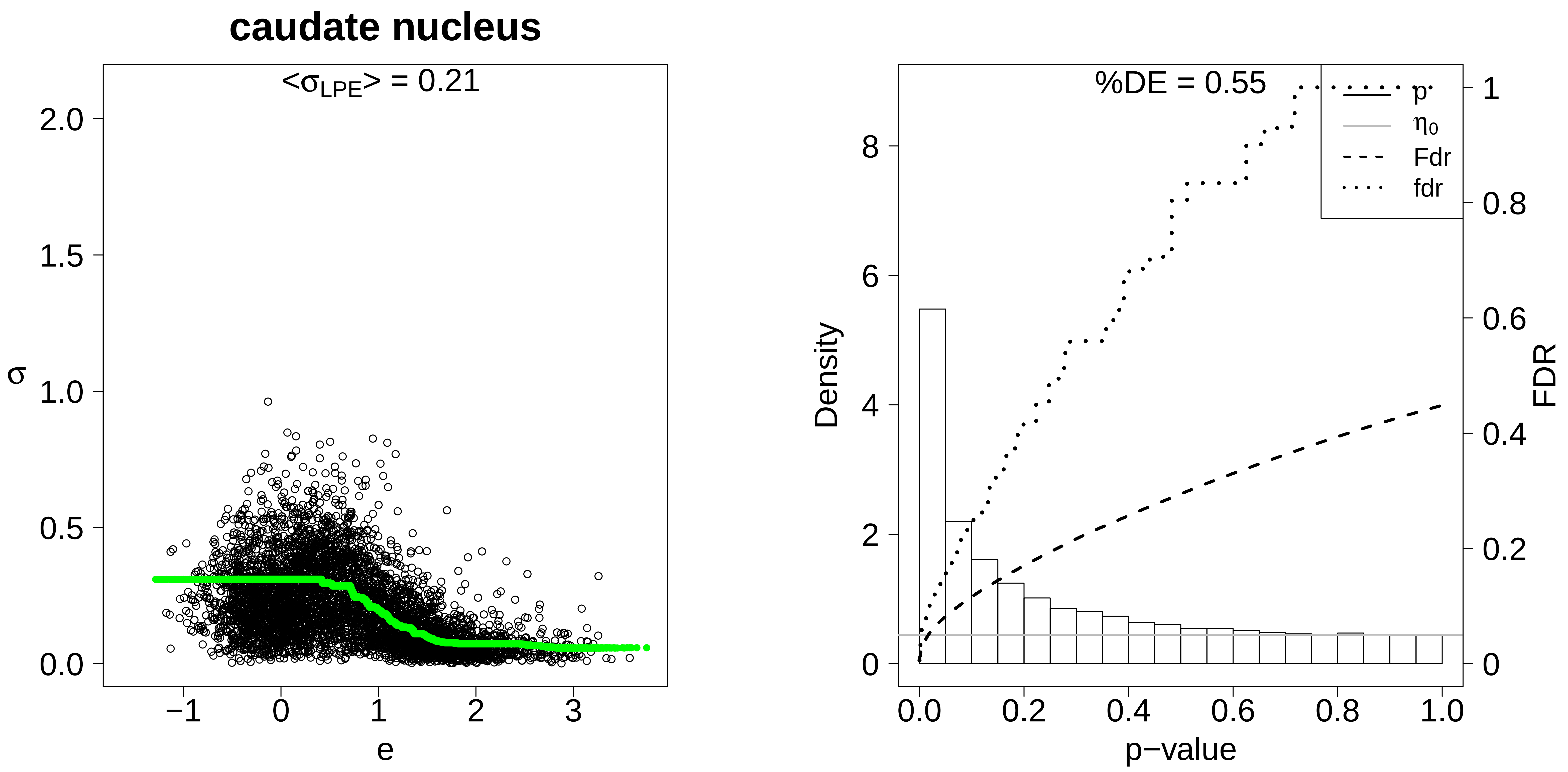

caudate nucleus
<σLPE> = 0.21
σ
e
%DE = 0.55
Density
FDR
p−value
p
η0
Fdr
fdr

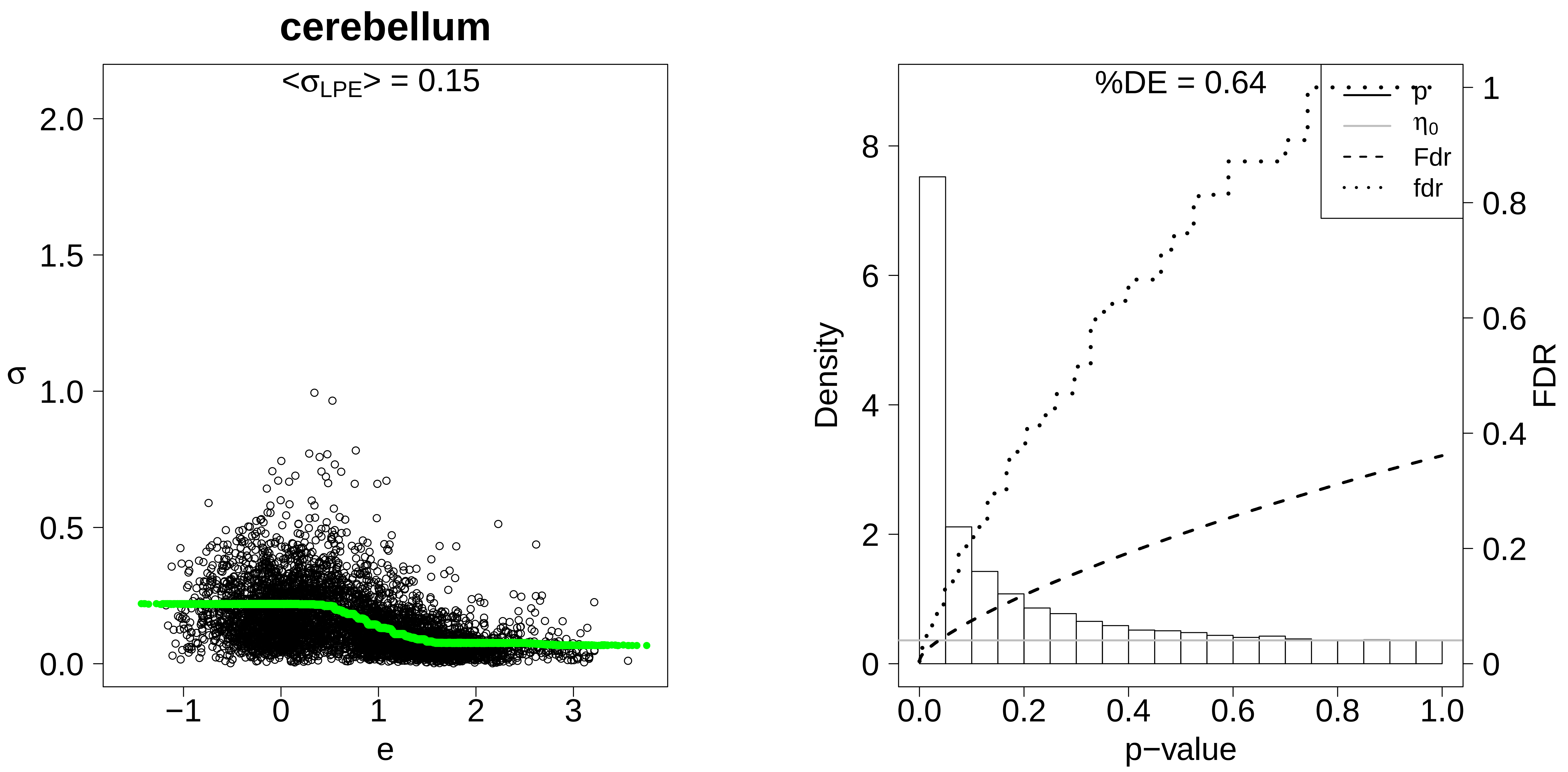
cerebellum
<σLPE> = 0.15
σ
e
%DE = 0.64
p
η0
Fdr
fdr
Density
FDR
p−value

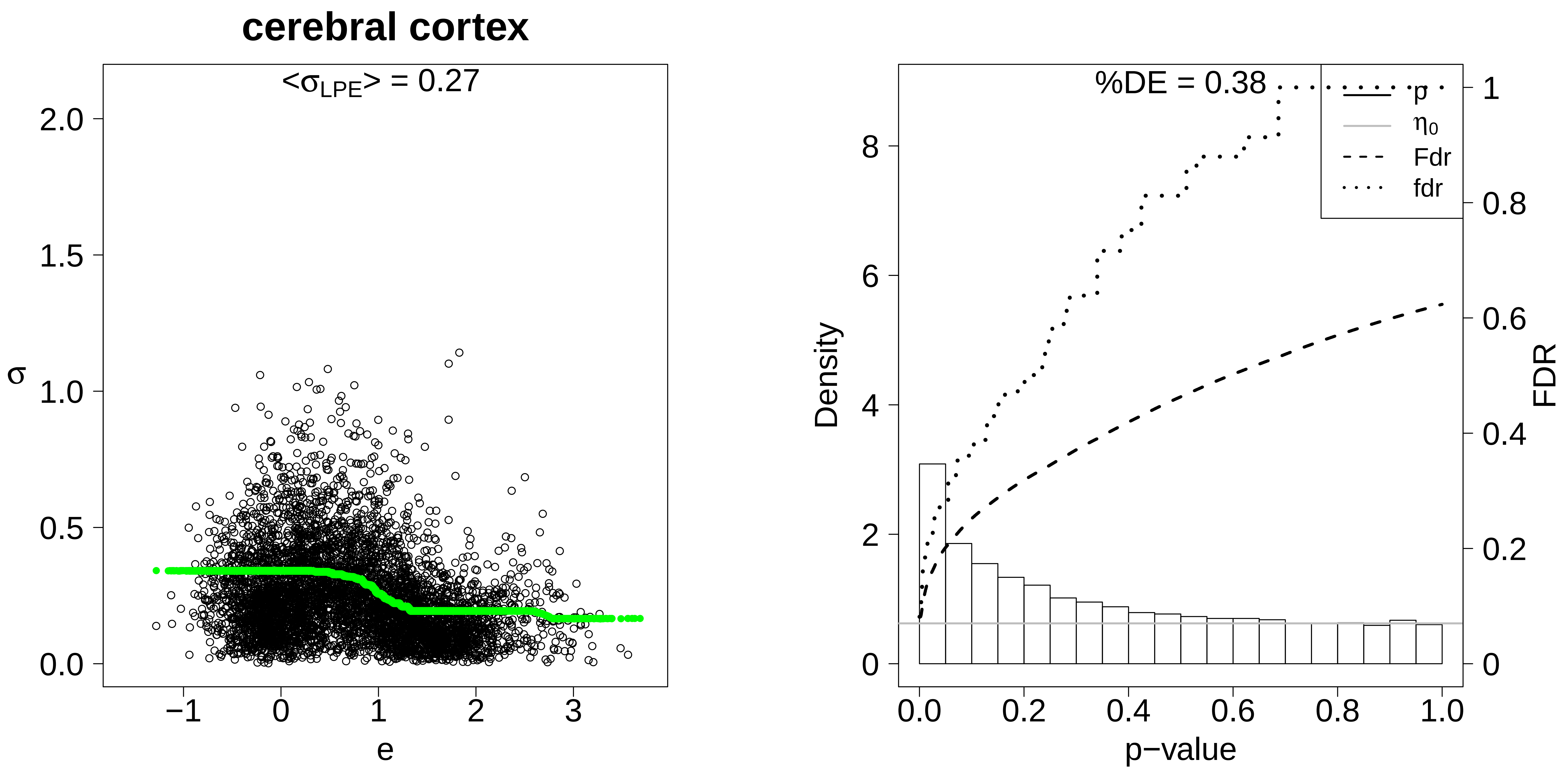

cerebral cortex
<σLPE> = 0.27
σ
e
%DE = 0.38
p
η0
Fdr
fdr
Density
FDR
p−value

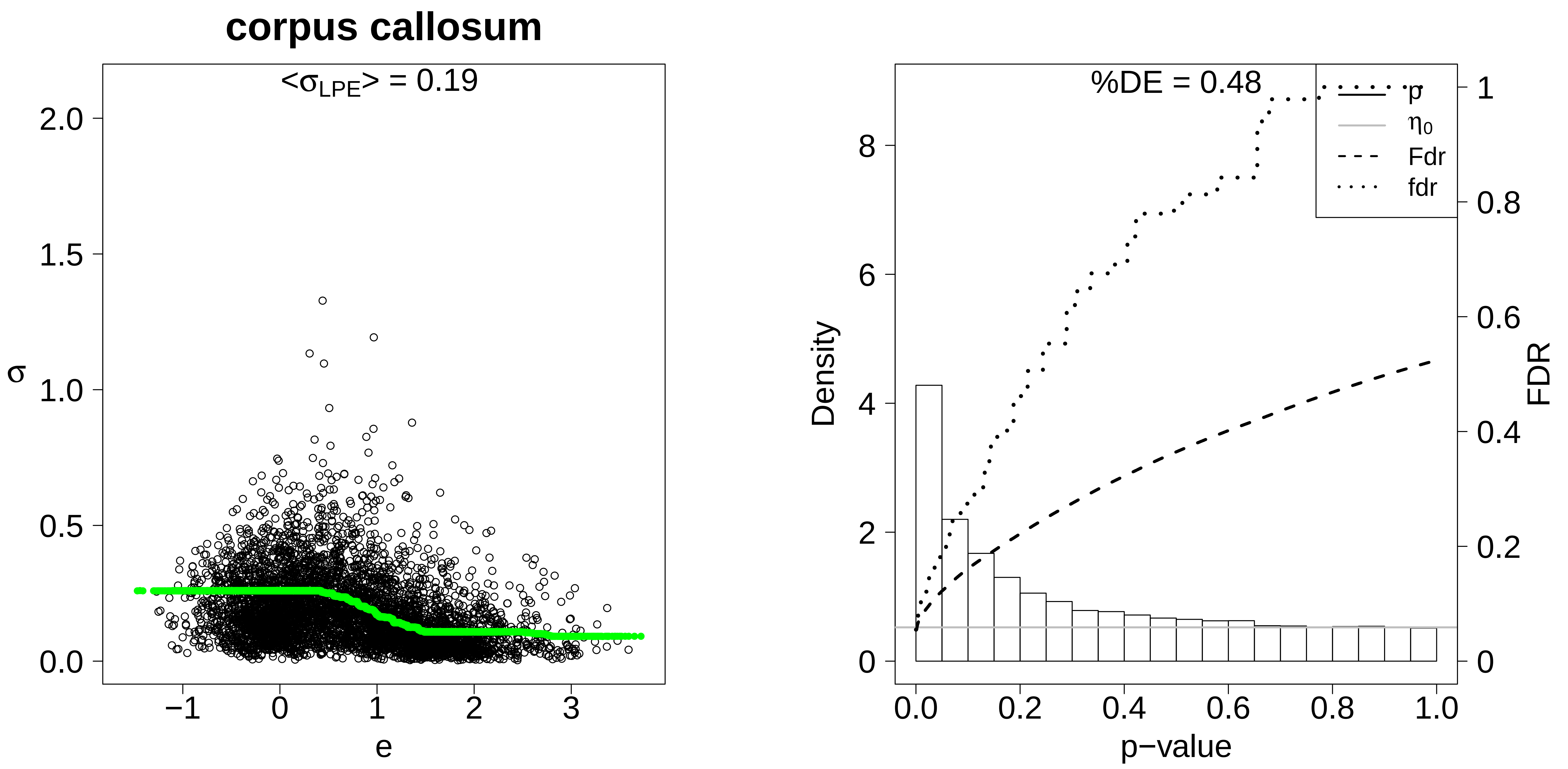

corpus callosum
<σLPE> = 0.19
σ
e
%DE = 0.48
p
η0
Fdr
fdr
Density
FDR
p−value

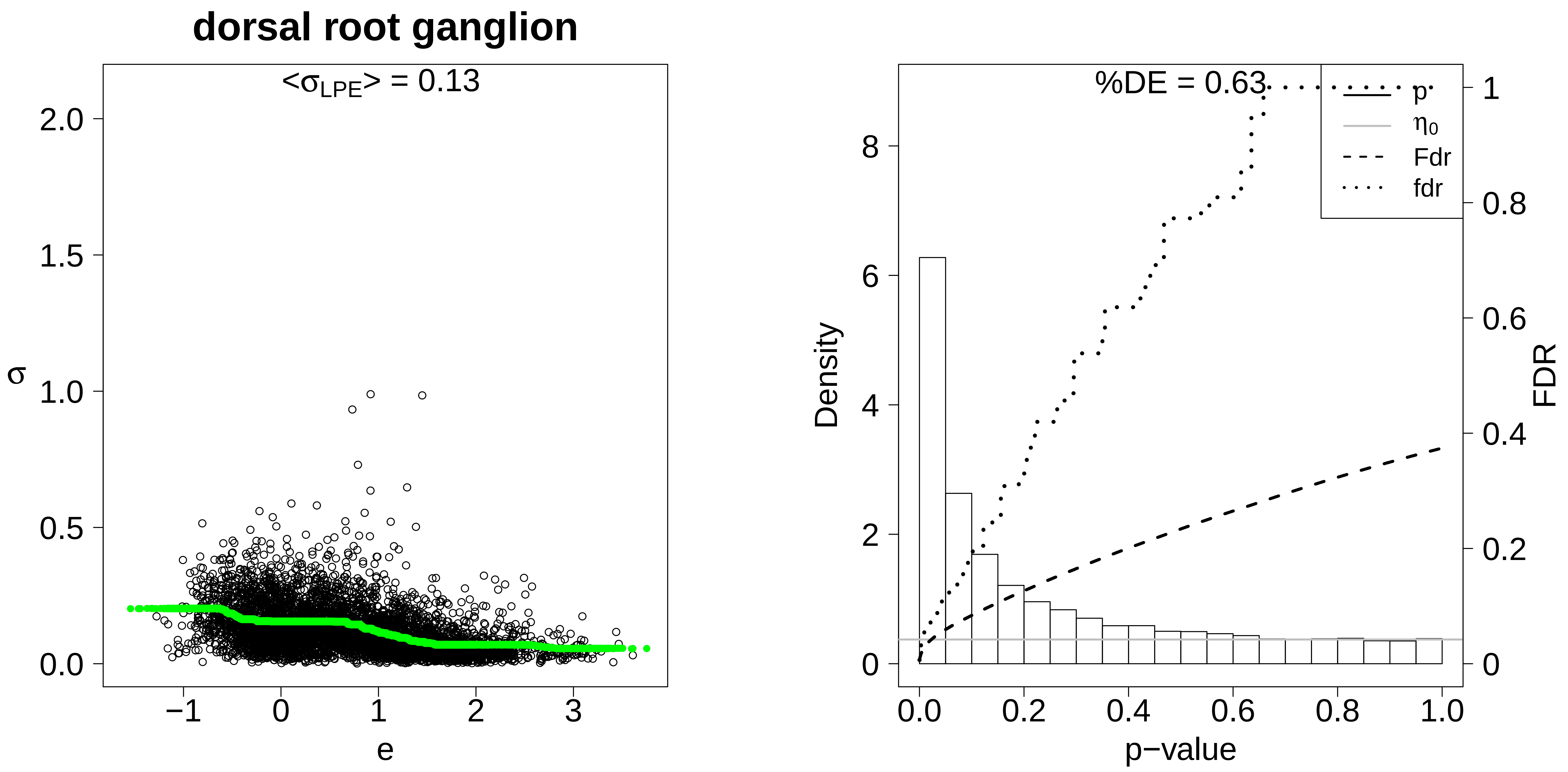

dorsal root ganglion
<σ_{LPE}> = 0.13
2.0
1.5
1.0
0.5
0.0
σ
−1
0
1
2
3
e
%DE = 0.63
p
η_0
Fdr
fdr
8
6
4
2
0
Density
0.0
0.2
0.4
0.6
0.8
1.0
p−value
1
0.8
0.6
0.4
0.2
0
FDR

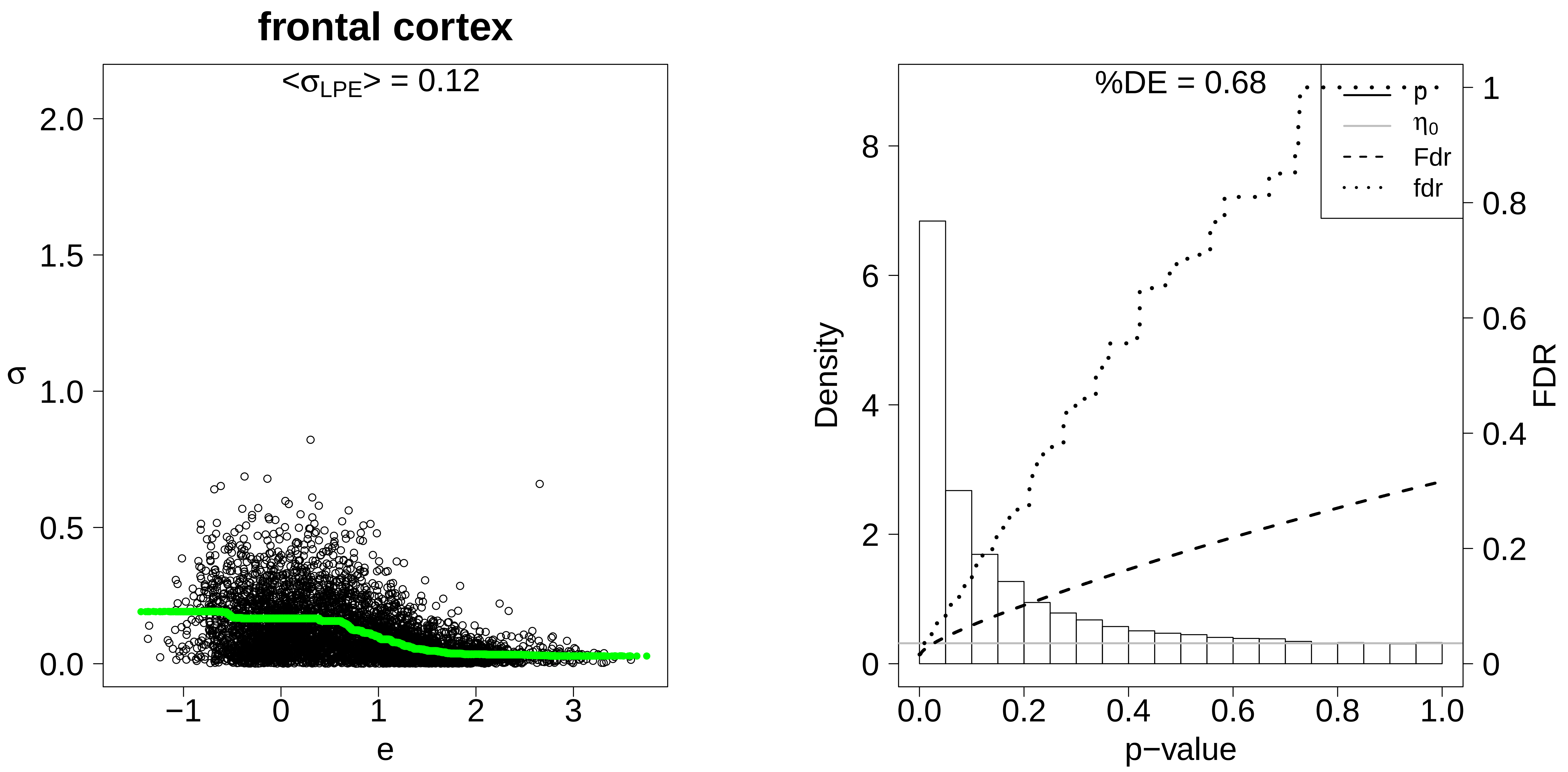

frontal cortex
<σLPE> = 0.12
σ
e
%DE = 0.68
p
η0
Fdr
fdr
Density
FDR
p−value

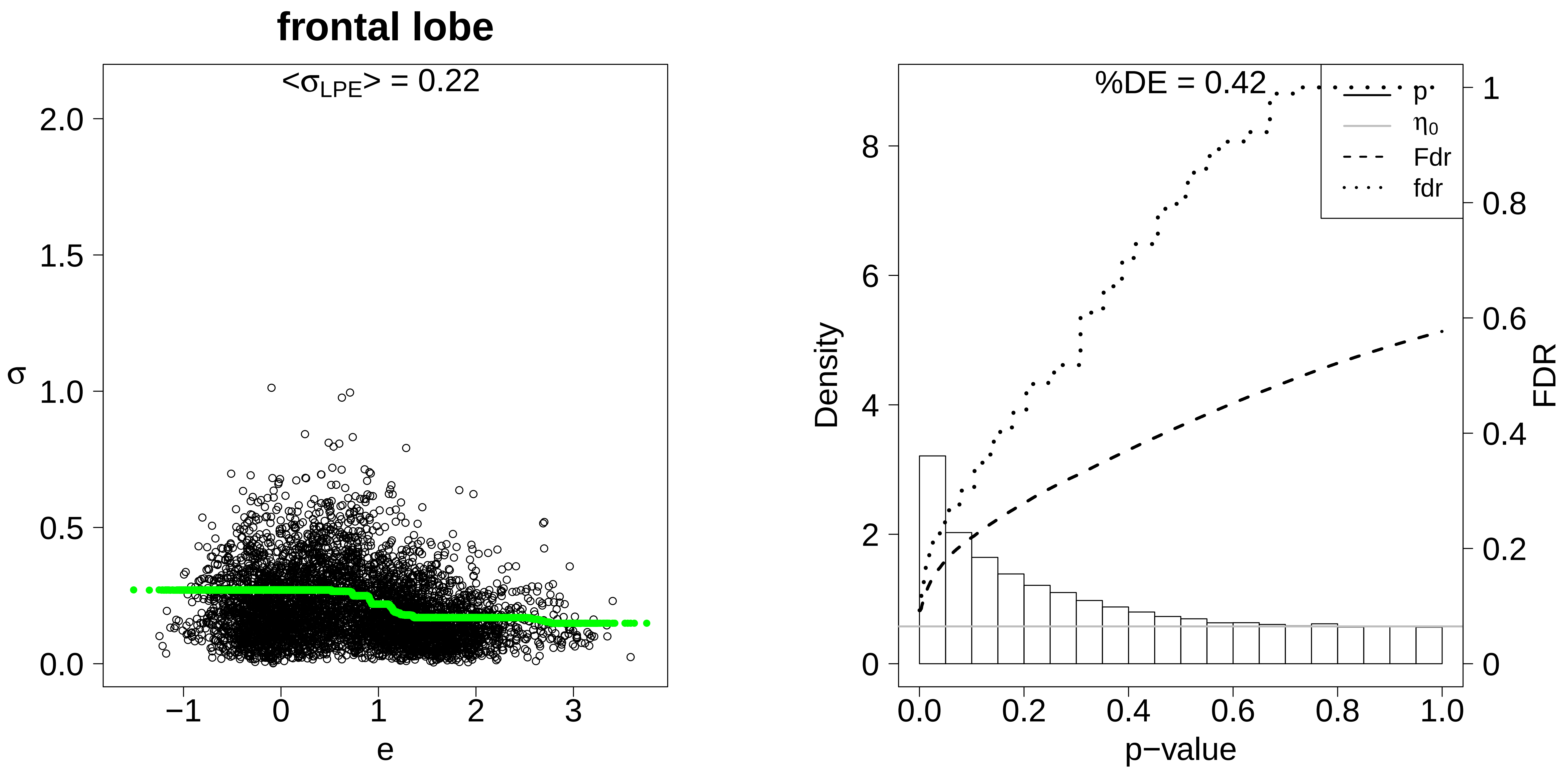

frontal lobe
<σLPE> = 0.22
σ
e
2.0
1.5
1.0
0.5
0.0
−1
0
1
2
3
%DE = 0.42
p
η0
Fdr
fdr
Density
FDR
p−value
8
6
4
2
0
0.0
0.2
0.4
0.6
0.8
1.0
1
0.8
0.6
0.4
0.2
0

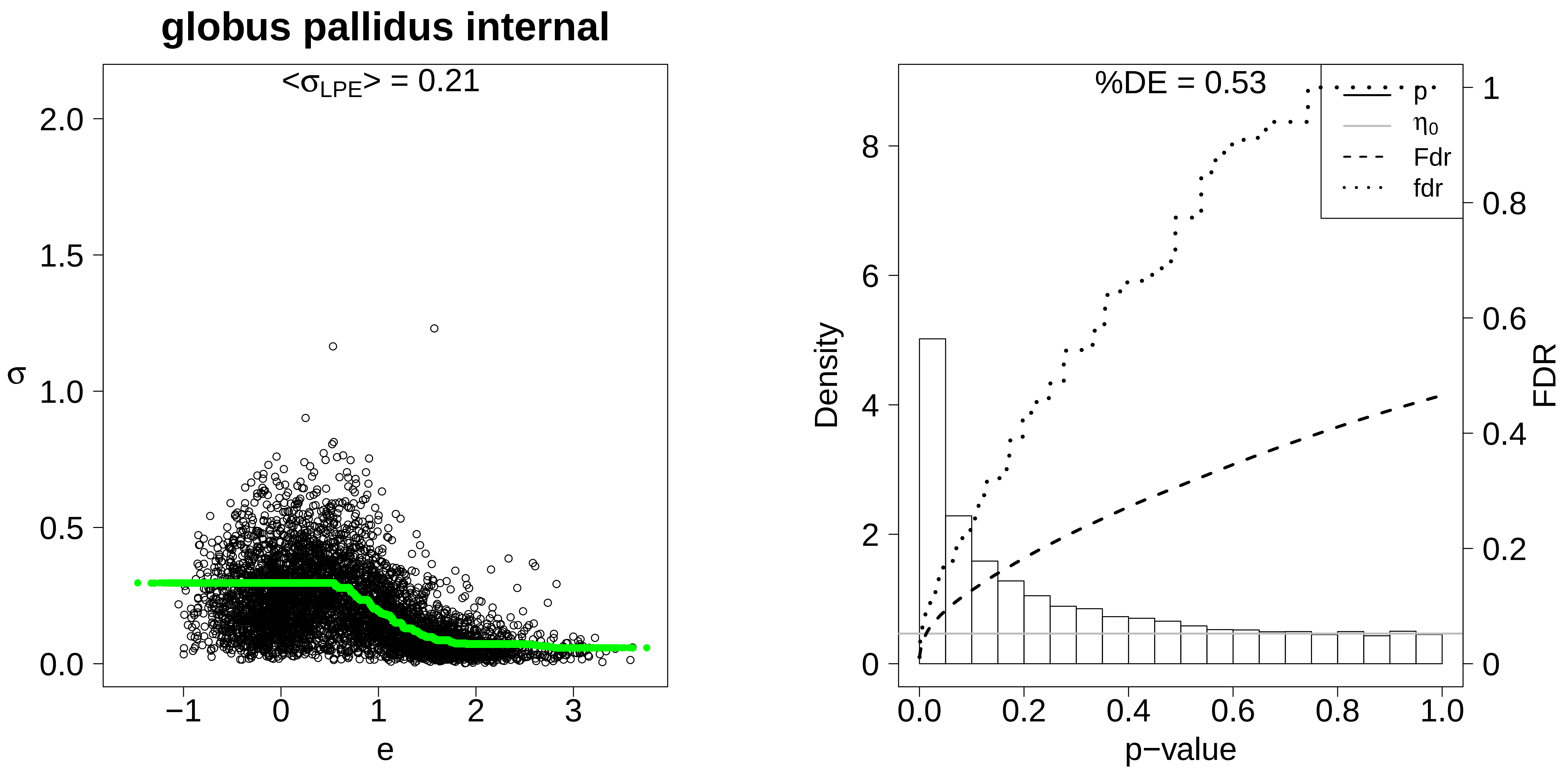

globus pallidus internal
<σLPE> = 0.21
σ
e
%DE = 0.53
Density
p−value
FDR
p
η0
Fdr
fdr

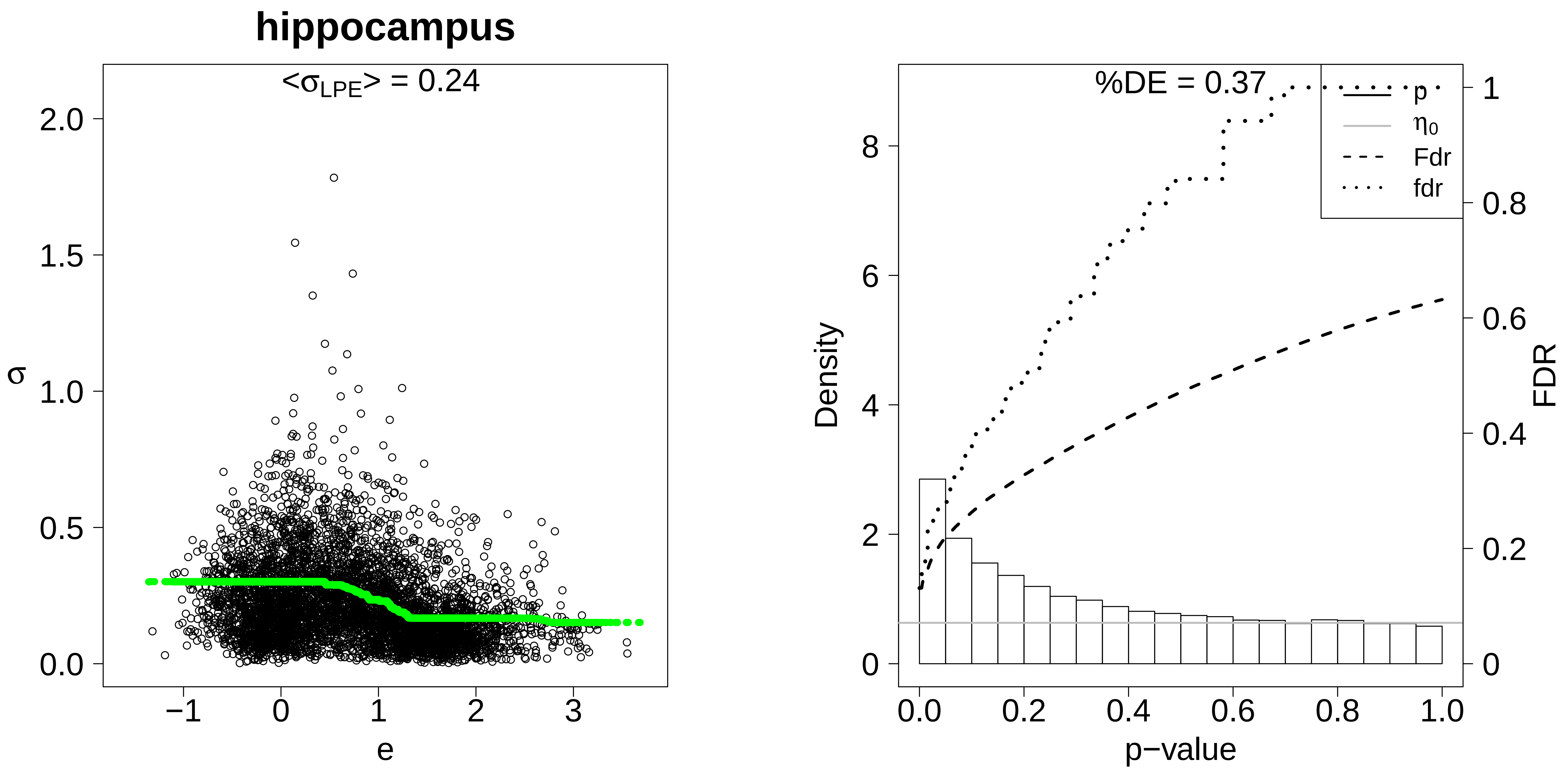

hippocampus
<σLPE> = 0.24
σ
2.0
1.5
1.0
0.5
0.0
−1
0
1
2
3
e
%DE = 0.37
p
η0
Fdr
fdr
Density
8
6
4
2
0
0.0
0.2
0.4
0.6
0.8
1.0
p−value
FDR
1
0.8
0.6
0.4
0.2
0

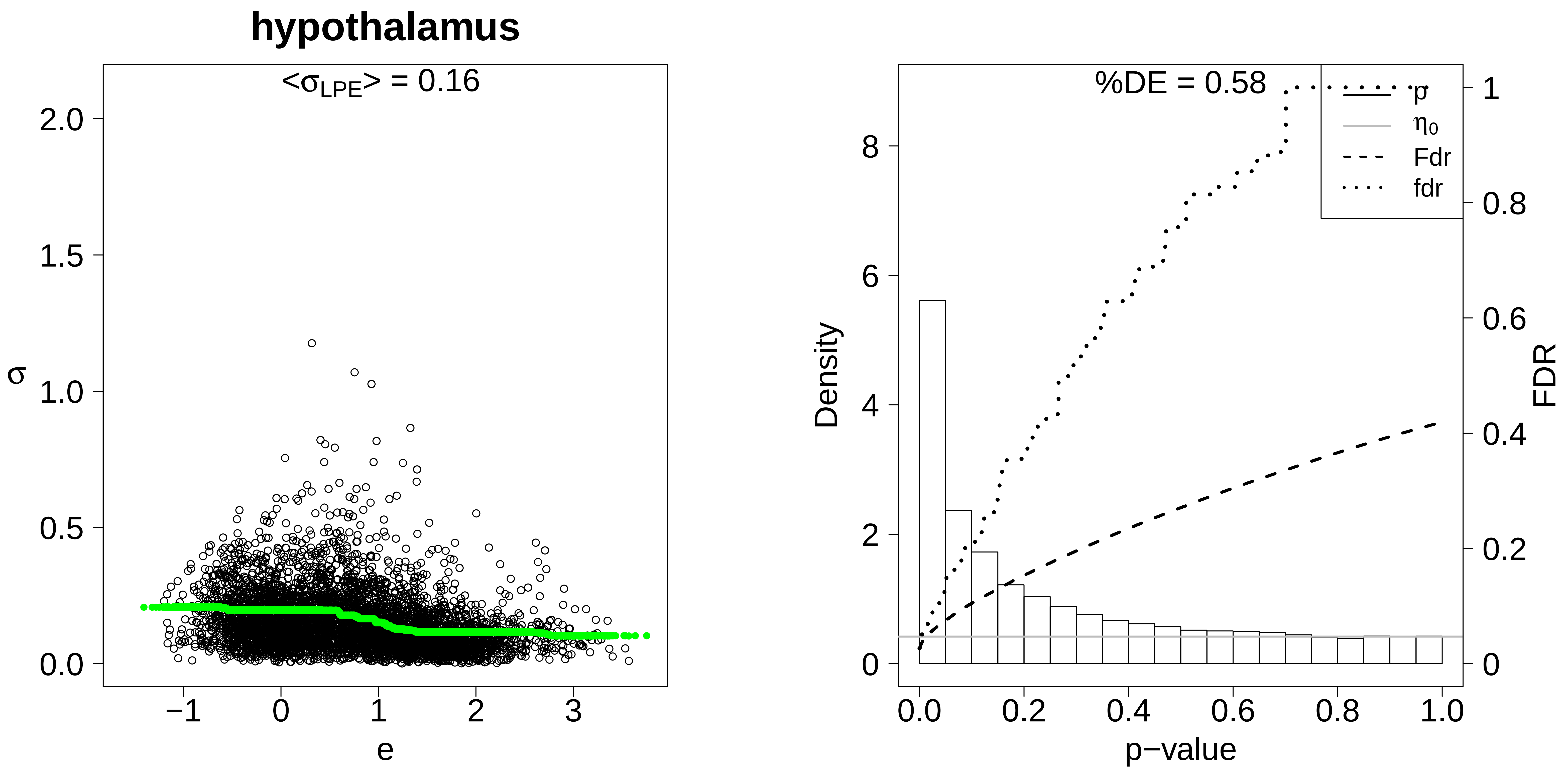

hypothalamus
<σLPE> = 0.16
σ
2.0
1.5
1.0
0.5
0.0
−1
0
1
2
3
e
%DE = 0.58
p
η0
Fdr
fdr
Density
8
6
4
2
0
0.0
0.2
0.4
0.6
0.8
1.0
p−value
FDR
1
0.8
0.6
0.4
0.2
0

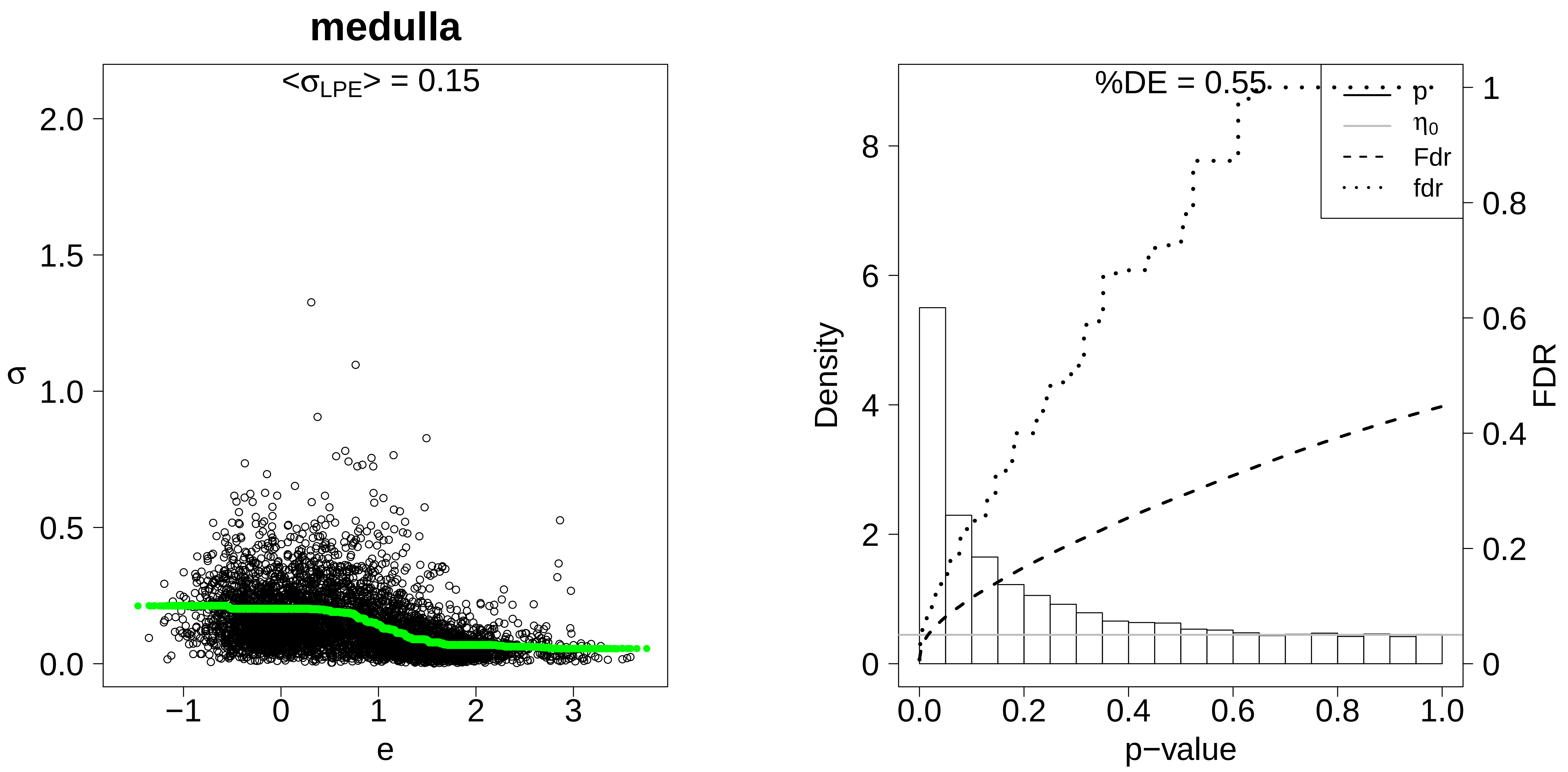

medulla
<σLPE> = 0.15
σ
e
2.0
1.5
1.0
0.5
0.0
−1
0
1
2
3
%DE = 0.55
p
η0
Fdr
fdr
Density
FDR
p−value
0.0
0.2
0.4
0.6
0.8
1.0
8
6
4
2
0
1
0.8
0.6
0.4
0.2
0

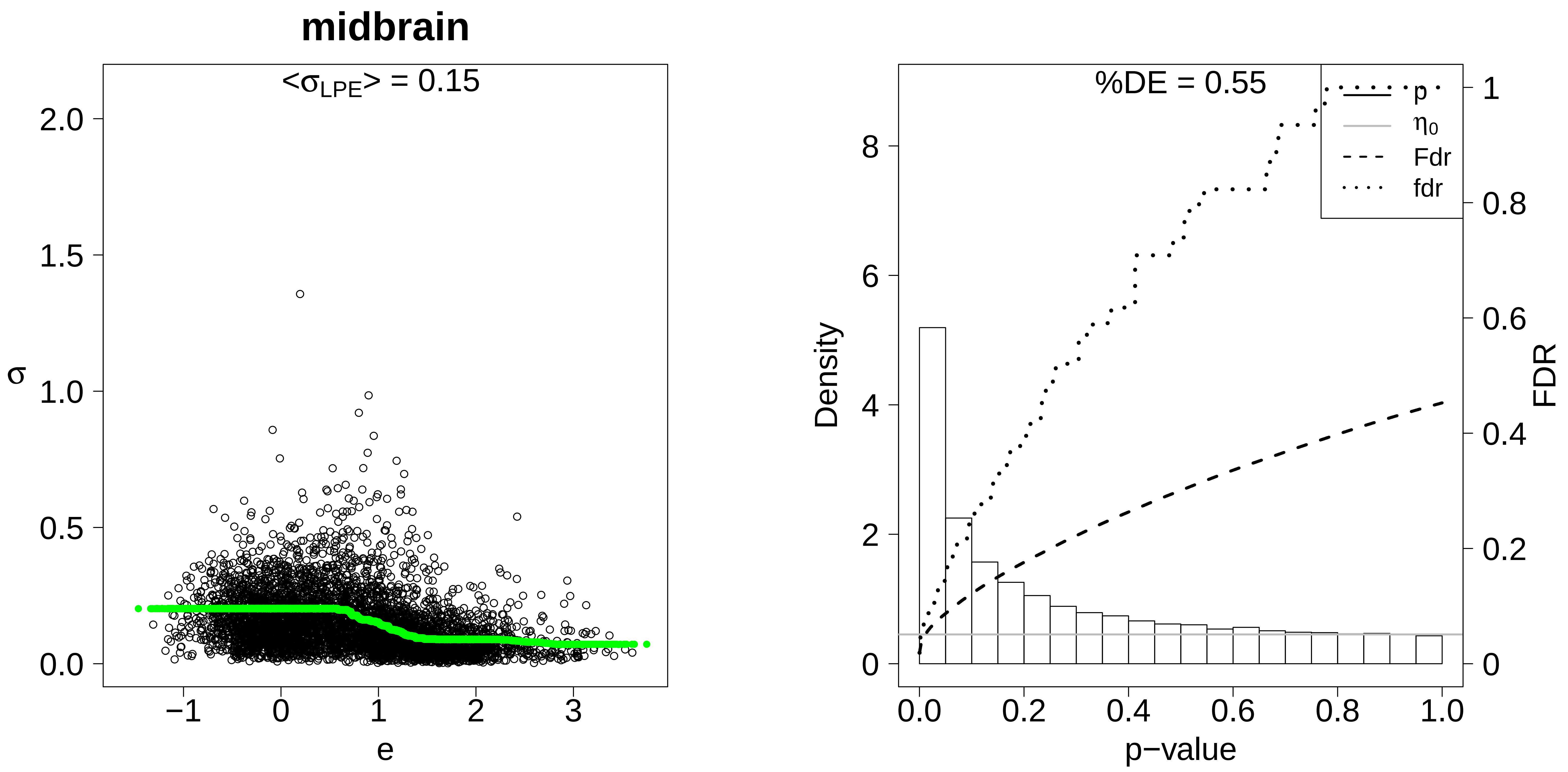

midbrain
<σLPE> = 0.15
σ
2.0
1.5
1.0
0.5
0.0
−1
0
1
2
3
e
%DE = 0.55
p
η0
Fdr
fdr
Density
8
6
4
2
0
0.0
0.2
0.4
0.6
0.8
1.0
p−value
1
0.8
0.6
0.4
0.2
0
FDR

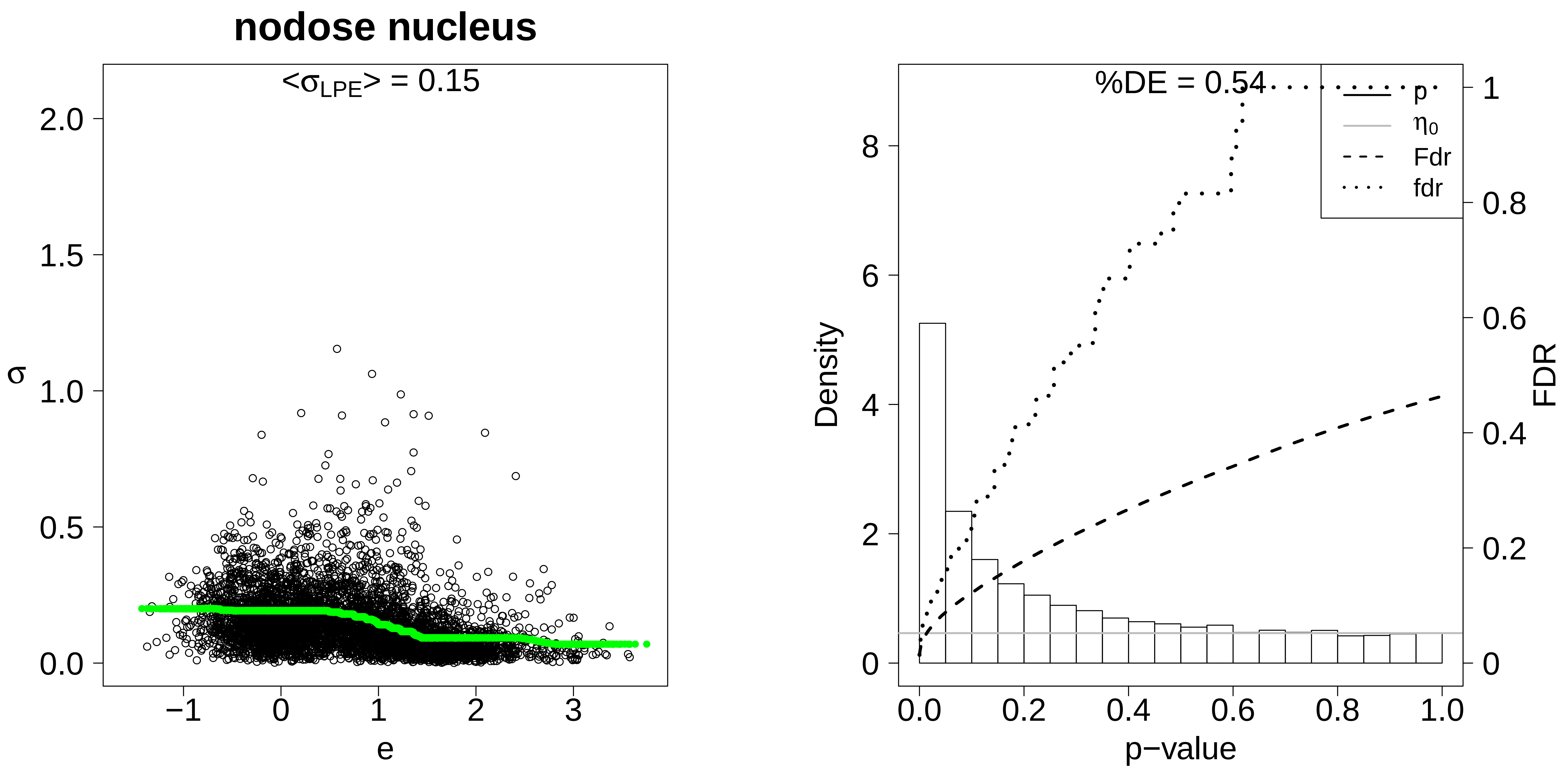

nodose nucleus
$<\sigma_{LPE}> = 0.15$
σ
e
%DE = 0.54
p
η_0
Fdr
fdr
Density
FDR
p−value

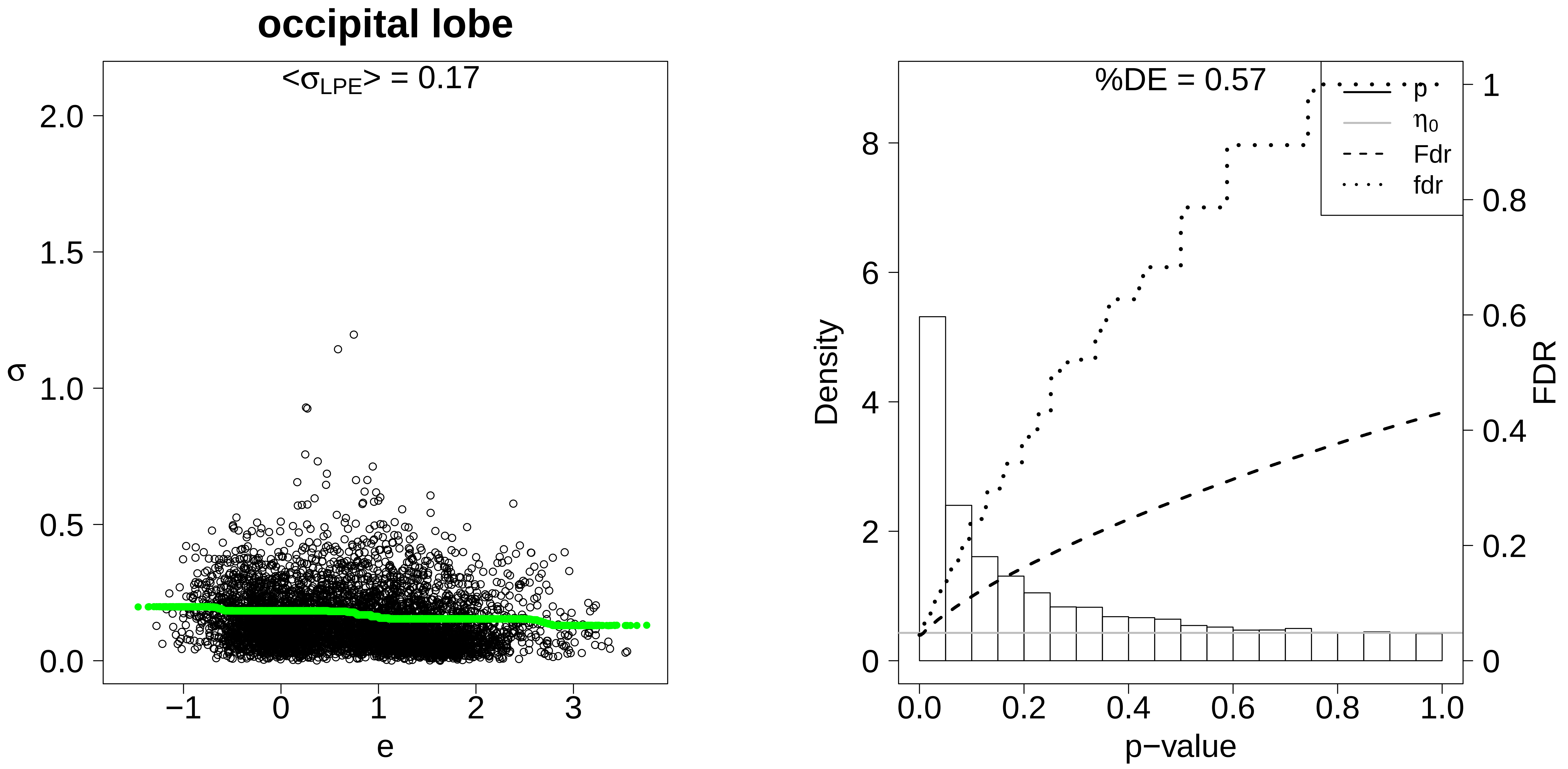

occipital lobe
<σ_LPE> = 0.17
σ
e
2.0
1.5
1.0
0.5
0.0
−1
0
1
2
3
%DE = 0.57
p
η0
Fdr
fdr
Density
FDR
p−value
0.0
0.2
0.4
0.6
0.8
1.0

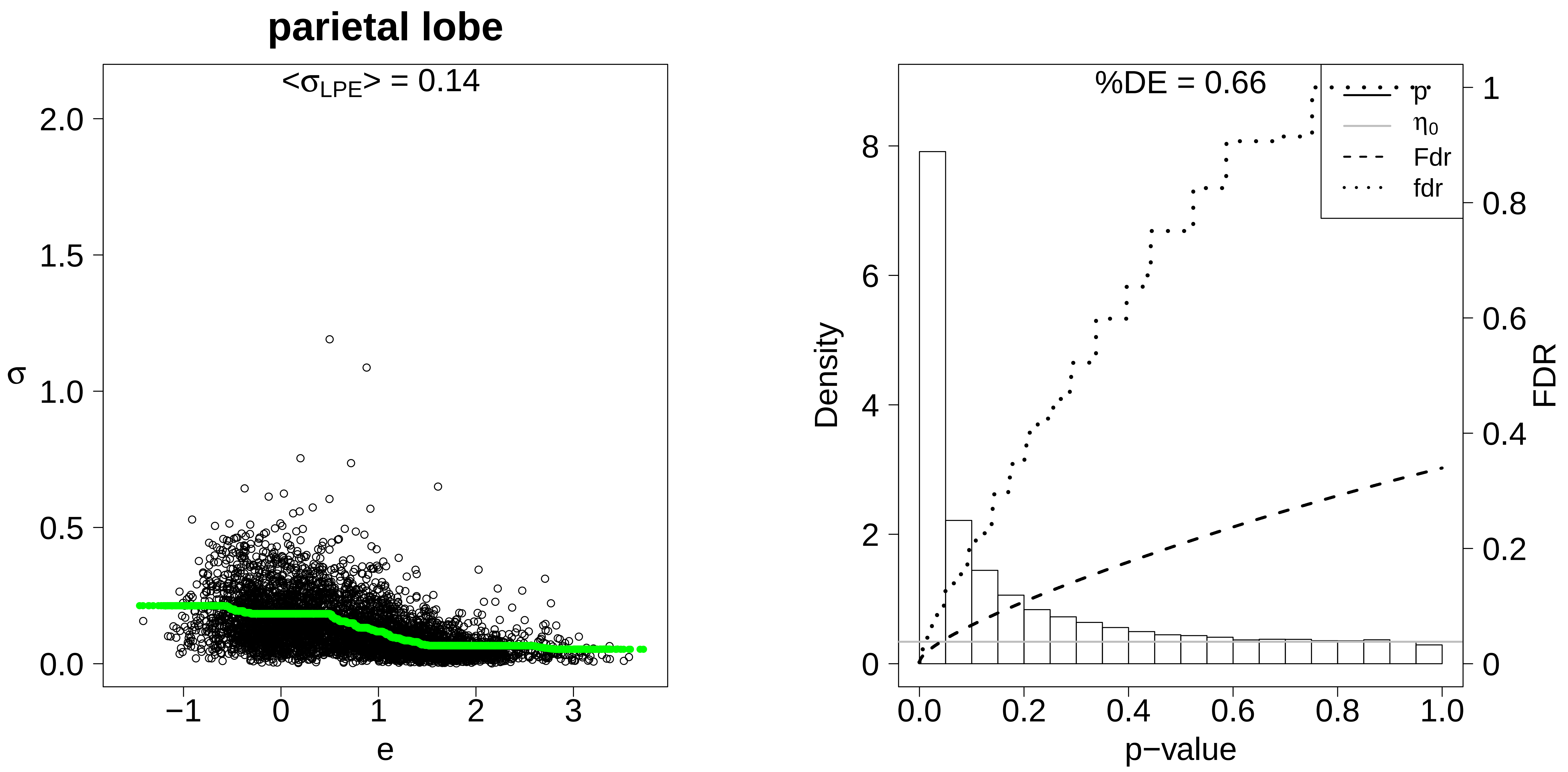

parietal lobe
<σ_LPE> = 0.14
σ
e
2.0
1.5
1.0
0.5
0.0
−1
0
1
2
3
%DE = 0.66
p
η0
Fdr
fdr
Density
FDR
p−value

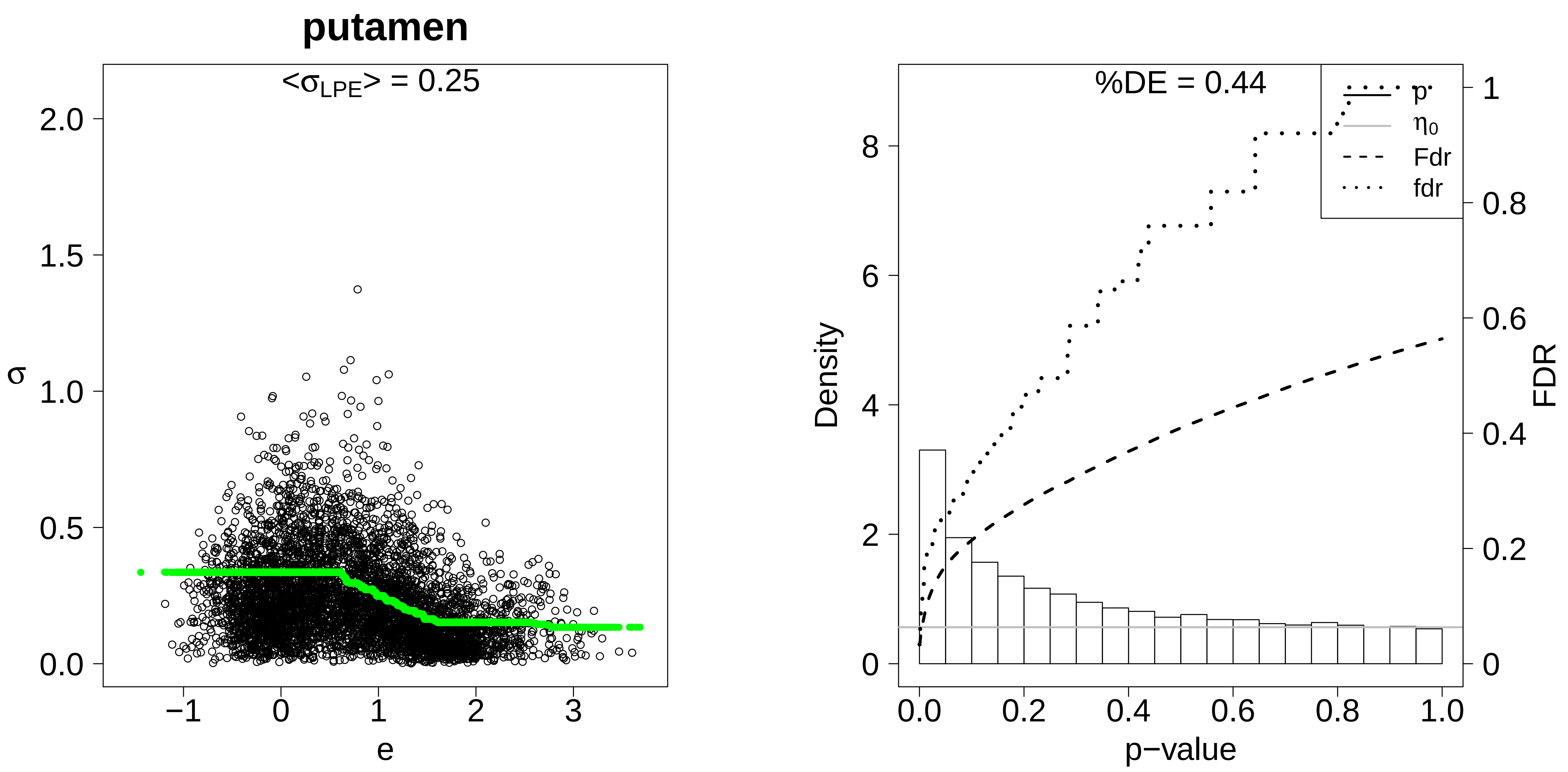

putamen
<σLPE> = 0.25
2.0
1.5
1.0
0.5
0.0
σ
−1
0
1
2
3
e
%DE = 0.44
p
η0
Fdr
fdr
8
6
4
2
0
Density
1
0.8
0.6
0.4
0.2
0
FDR
0.0
0.2
0.4
0.6
0.8
1.0
p−value

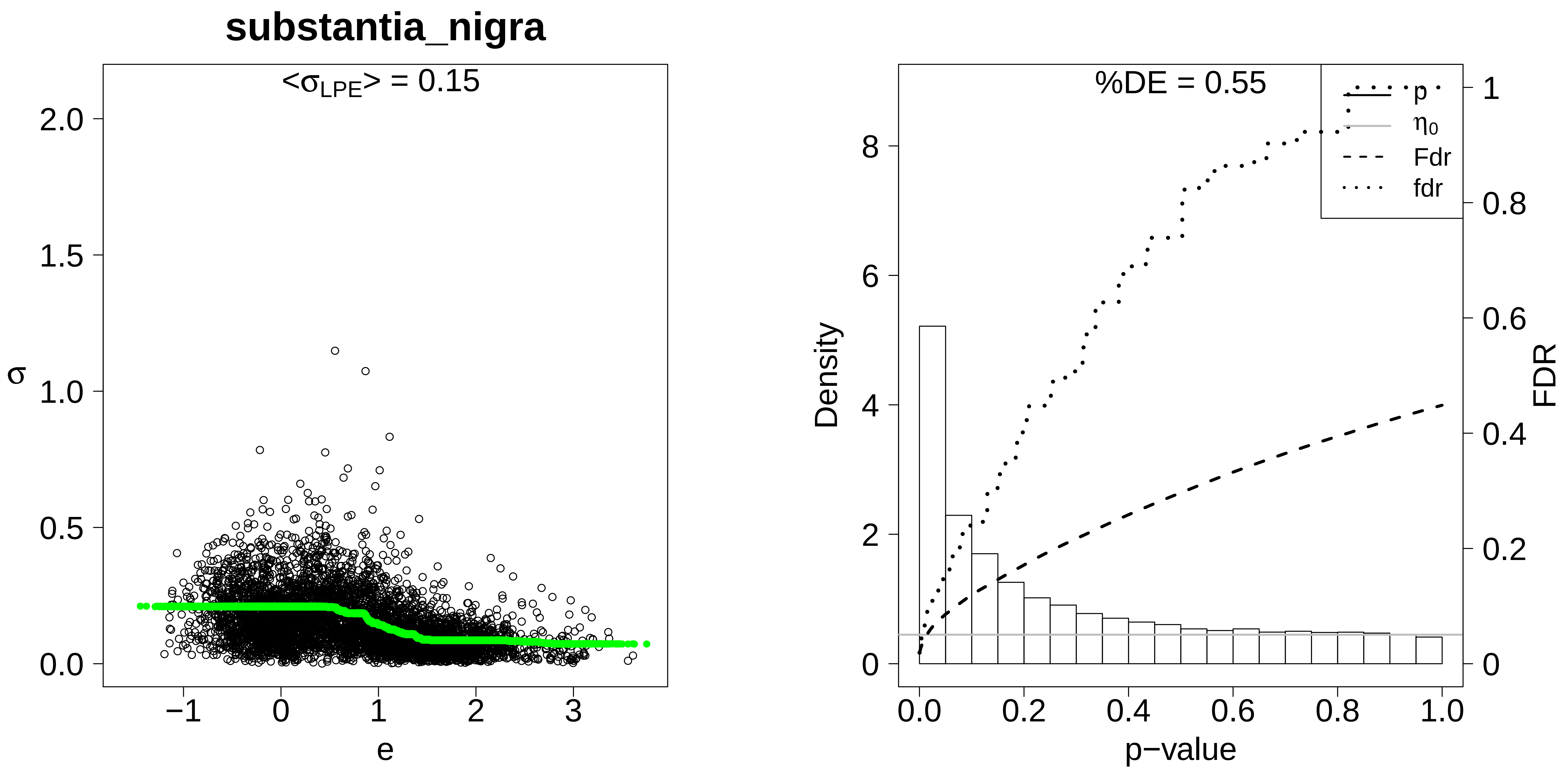
substantia_nigra
<σLPE> = 0.15
σ
e
%DE = 0.55
p
η0
Fdr
fdr
Density
FDR
p−value

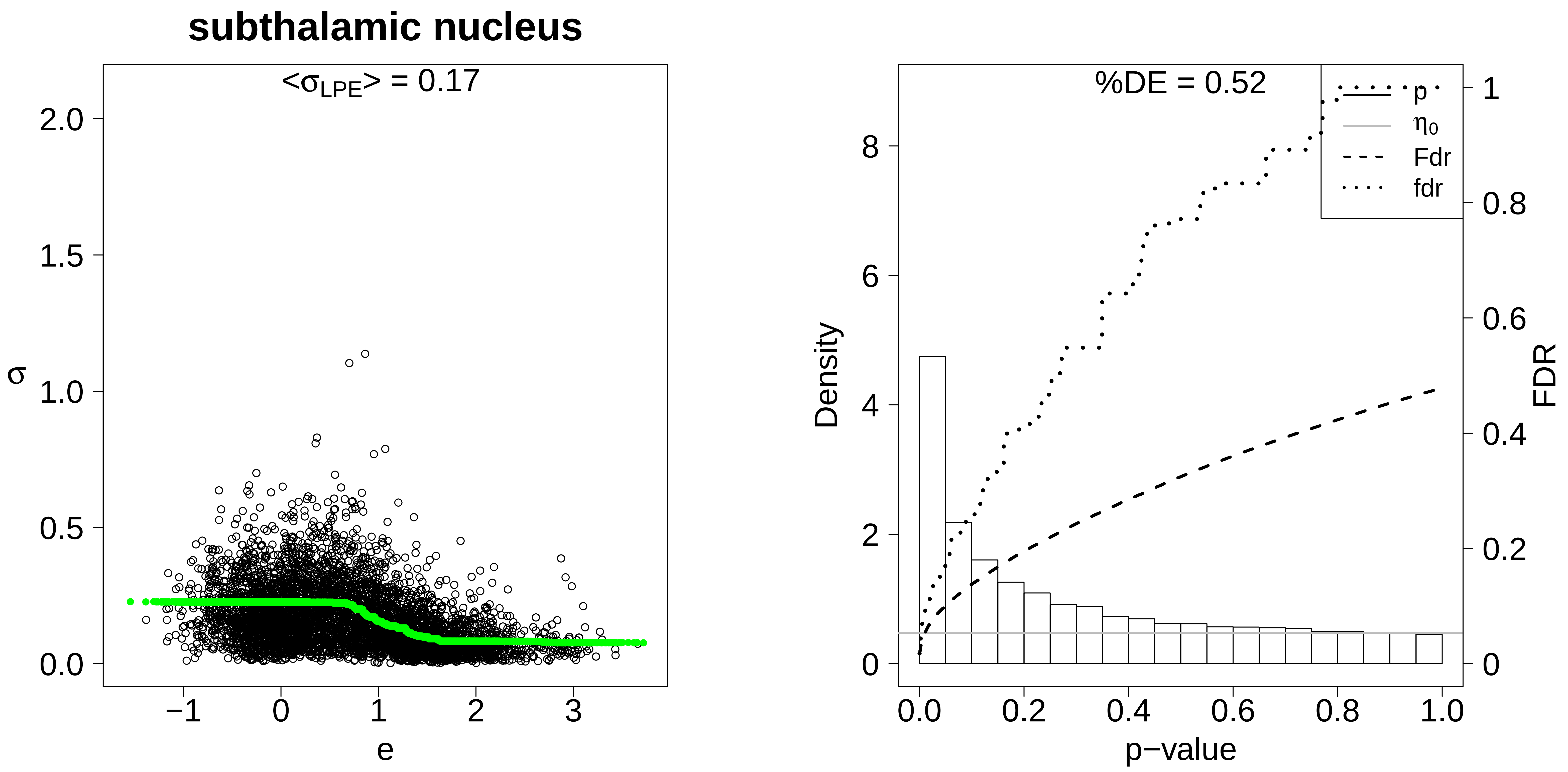

subthalamic nucleus
<σLPE> = 0.17
σ
e
%DE = 0.52
Density
p−value
FDR
p
η0
Fdr
fdr

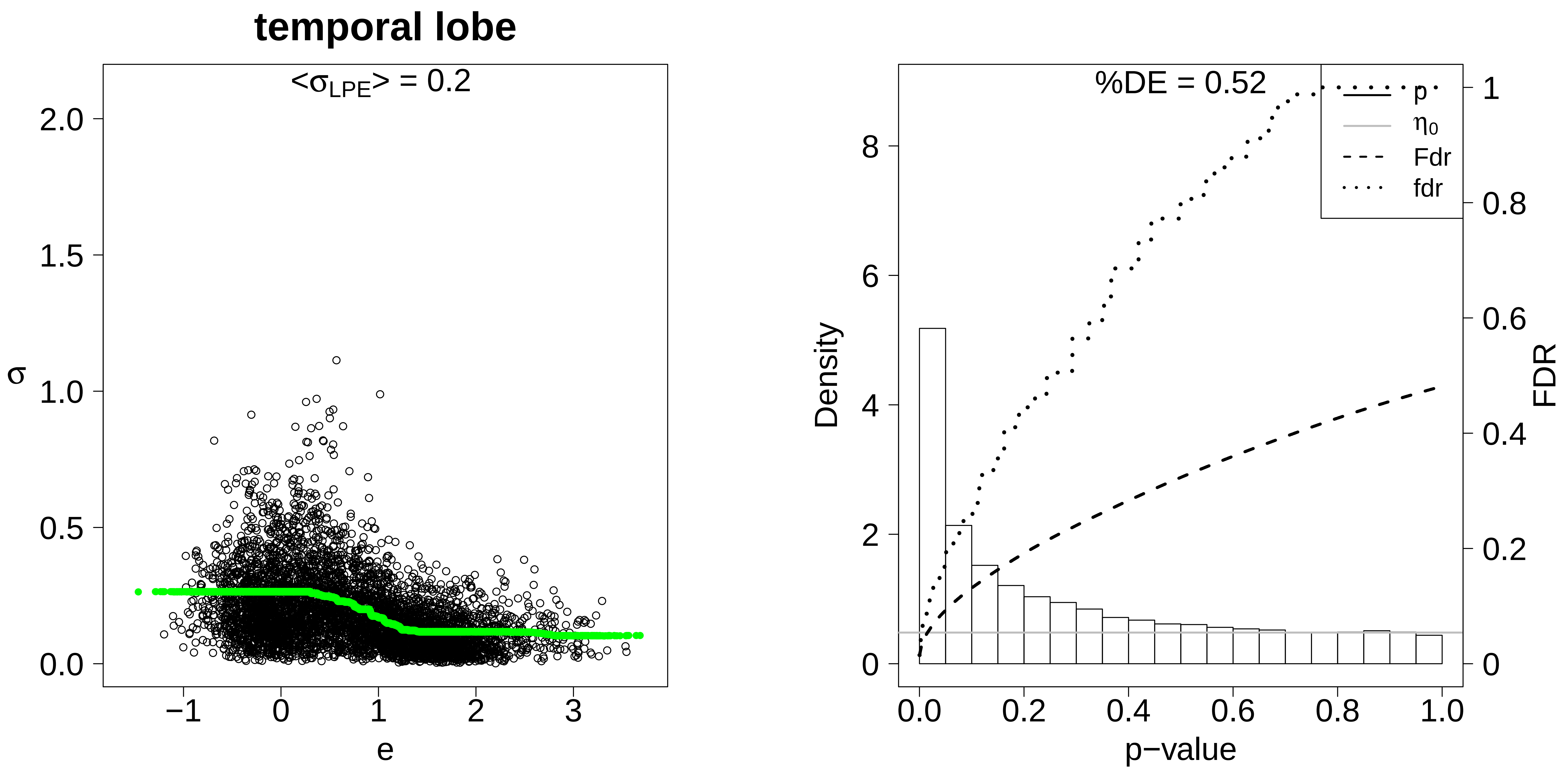

temporal lobe
<σLPE> = 0.2
2.0
1.5
1.0
0.5
0.0
σ
−1
0
1
2
3
e
%DE = 0.52
p
η0
Fdr
fdr
8
6
4
2
0
Density
1
0.8
0.6
0.4
0.2
0
FDR
0.0
0.2
0.4
0.6
0.8
1.0
p−value

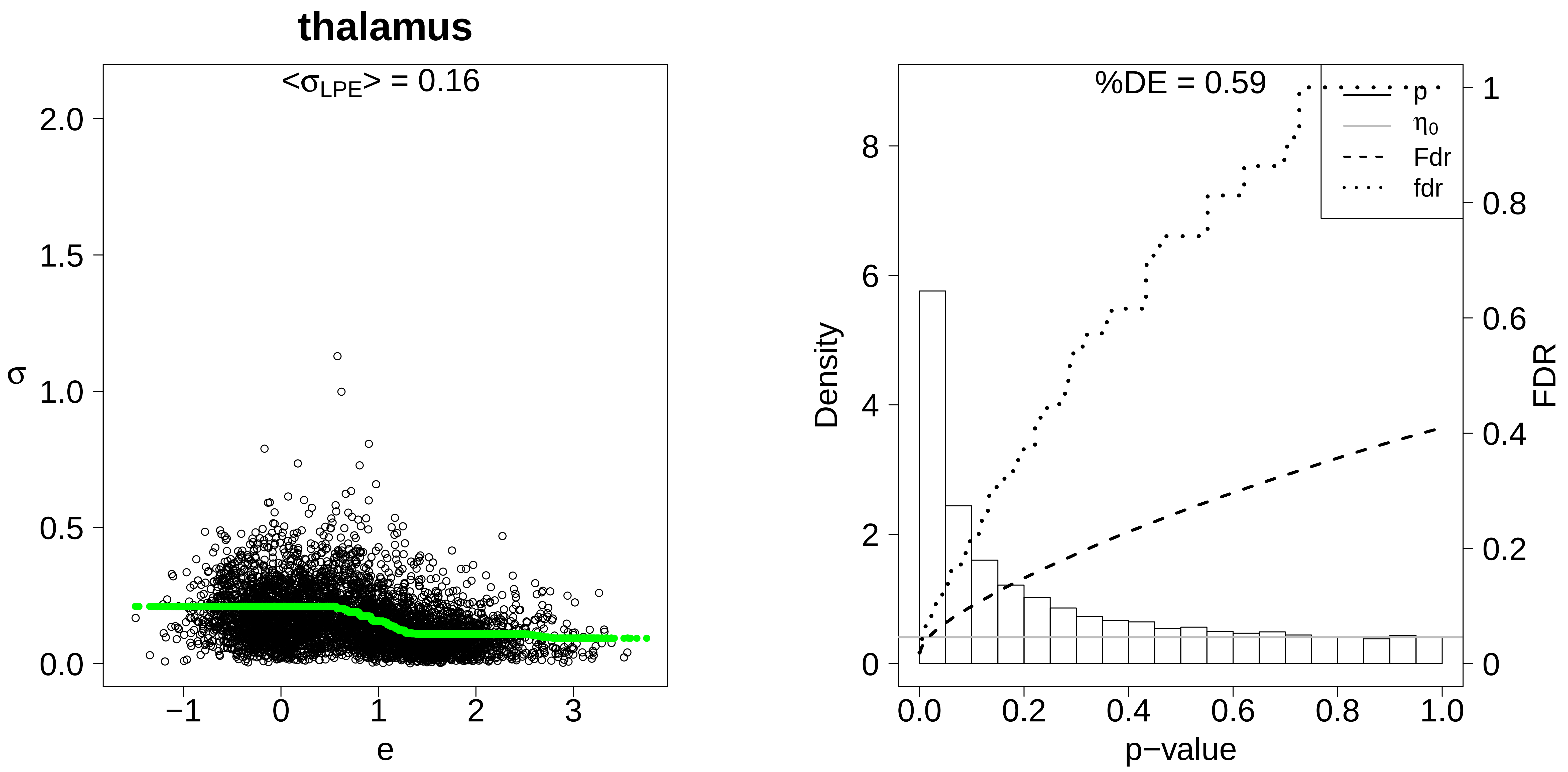

thalamus
<σLPE> = 0.16
σ
e
%DE = 0.59
p
η0
Fdr
fdr
Density
FDR
p−value

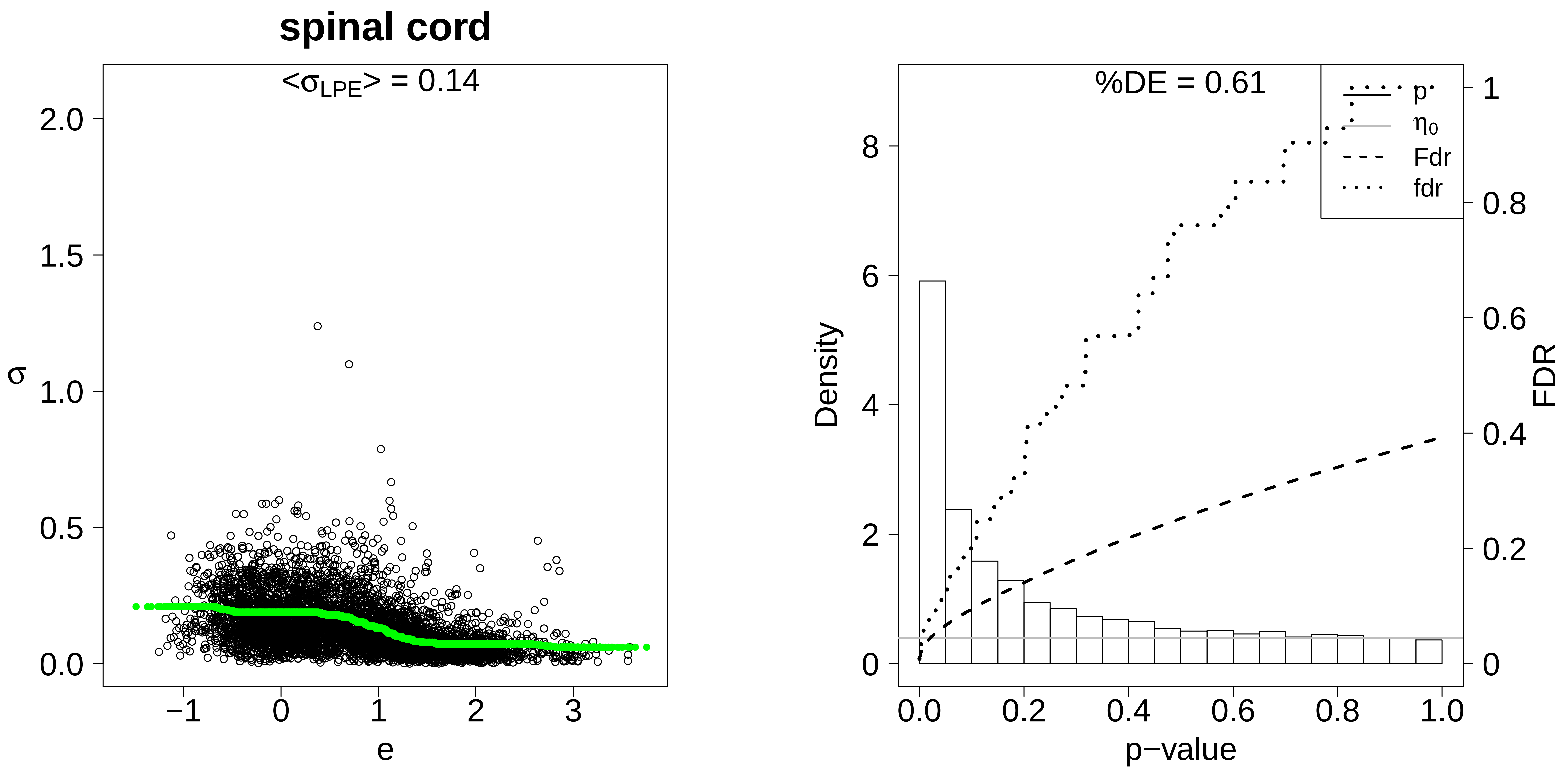

spinal cord
<σ_LPE> = 0.14
σ
2.0
1.5
1.0
0.5
0.0
−1
0
1
2
3
e
%DE = 0.61
p·
η0
Fdr
fdr
Density
8
6
4
2
0
0.0
0.2
0.4
0.6
0.8
1.0
p−value
FDR
1
0.8
0.6
0.4
0.2
0
